# Supplementary material for: Dalpiciclib combined with pyrotinib and endocrine therapy in women with ER-positive, HER2-positive advanced breast cancer: A prospective, multicenter, single-arm, phase 2 trial
Source: PLoS Med. 2025 Jul 31;22(7):e1004669. doi: 10.1371/journal.pmed.1004669 (PMC12312931; doi:10.1371/journal.pmed.1004669)
Supplement: S2 Fig — (DOCX) [file pmed.1004669.s002.docx]

**S2 Fig. Exploratory subgroup analyses of ORR by baseline factors.**


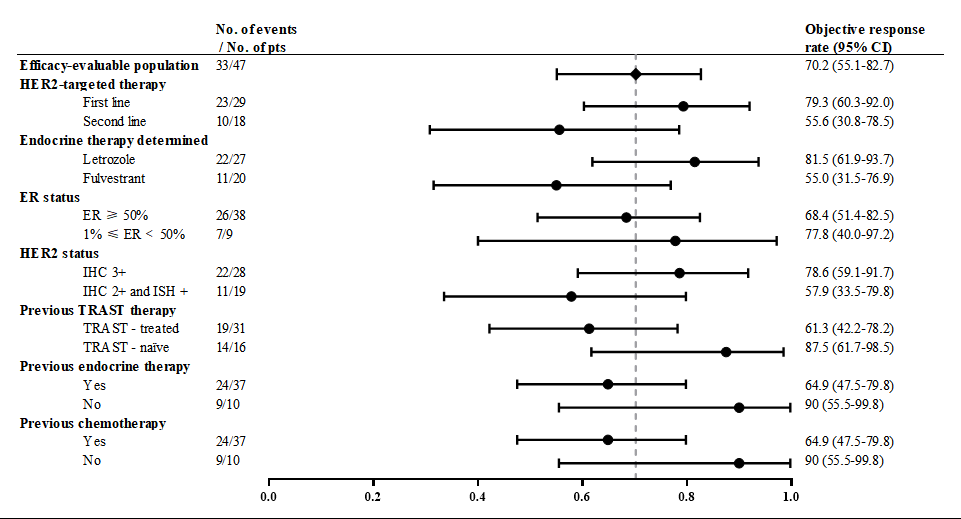


Data are presented as ORR (%) and 95% CI. The black dots indicate the ORR following stratifications. CI confidence interval, ER estrogen receptor, FISH fluorescence in situ hybridization, HER2 human epidermal growth factor receptor 2, IHC immunohistochemistry, ORR objective response rate, PR progesterone receptor, TRAST trastuzumab. The wide confidence intervals in these subgroup analyses reflect the high degree of uncertainty noted and should be interpreted with caution.
