# Supplementary material for: Dalpiciclib combined with pyrotinib and endocrine therapy in women with ER-positive, HER2-positive advanced breast cancer: A prospective, multicenter, single-arm, phase 2 trial
Source: PLoS Med. 2025 Jul 31;22(7):e1004669. doi: 10.1371/journal.pmed.1004669 (PMC12312931; doi:10.1371/journal.pmed.1004669)
Supplement: S4 Fig — (DOCX) [file pmed.1004669.s004.docx]

**S4 Fig. Progression-free survival in subgroups of different endocrine therapy.**


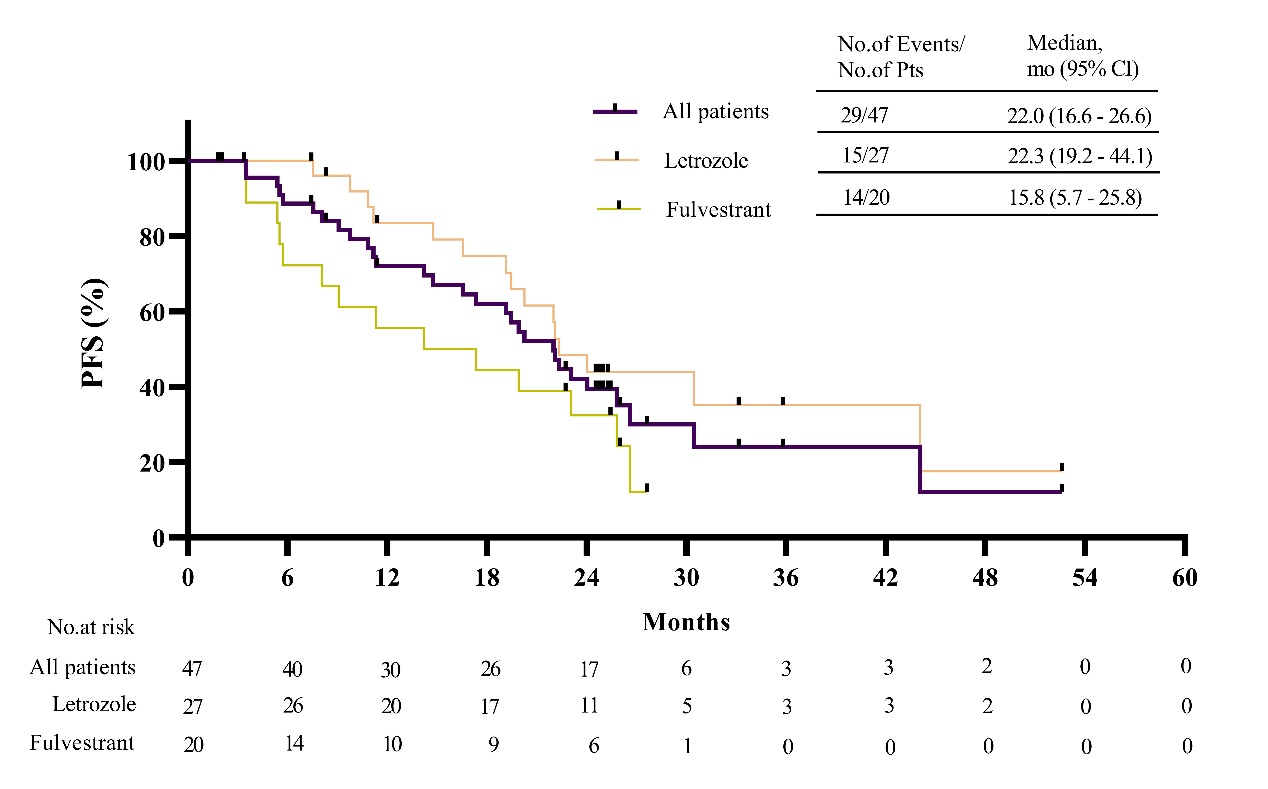
 Kaplan-Meier estimates of progression-free survival in overall efficacy-evaluable population and subgroups of different endocrine therapy. CI confidence interval, PFS progression-free survival.
