# Supplementary material for: Dalpiciclib combined with pyrotinib and endocrine therapy in women with ER-positive, HER2-positive advanced breast cancer: A prospective, multicenter, single-arm, phase 2 trial
Source: PLoS Med. 2025 Jul 31;22(7):e1004669. doi: 10.1371/journal.pmed.1004669 (PMC12312931; doi:10.1371/journal.pmed.1004669)
Supplement: S1 Table — (DOCX) [file pmed.1004669.s006.docx]

**S1 Table. Pharmacokinetic**

1. **PK parameters following multiple dosing of dalpiciclib, pyrotinib, and letrozole or fulvestrant.**

| **Dalpiciclib combined with pyrotinib and letrozole or fulvestrant (C1D21)** | **Cmax (ng/mL)** | **AUClast (h*ng/mL)** |
| --- | --- | --- |
| Pyrotinib | 93.6 | 1310 |
| Dalpiciclib | 133 | 2310 |
| Letrozole | 125 | 2410 |

1. **Steady-state valley concentration**

| **VISIT** | **Pyrotinib** | | | **Letrozole** | | | **Dalpiciclib** | | |
| --- | --- | --- | --- | --- | --- | --- | --- | --- | --- |
|  | n | Geometric Mean | Geometric CV% | n | Geometric Mean | Geometric CV% | n | Geometric Mean | Geometric CV% |
| C1D22 | 12 | 29.16 | 69.11 | 8 | 102.39 | 37.35 | 12 | 58.3 | 37.21 |
| C3D22 | 7 | 34.45 | 38.66 | 5 | 188.19 | 28.7 | 7 | 70.97 | 32.73 |
| C5D22 | 7 | 41.44 | 41.09 | 5 | 130.43 | 88.16 | 8 | 53.11 | 297.65 |
| C8D22 | 6 | 34.02 | 88.43 | 4 | 148.58 | 43.56 | 6 | 72.23 | 33.94 |
| C12D22 | 8 | 34.85 | 46.12 | 6 | 170.96 | 31.74 | 9 | 49.63 | 190.71 |

1. **Ratio of steady-state valley concentration to C1D22 valley concentration**

| **VISIT** | **Pyrotinib** | | | **Letrozole** | | | **Dalpiciclib** | | |
| --- | --- | --- | --- | --- | --- | --- | --- | --- | --- |
|  | n | Geometric Mean | Geometric CV% | n | Geometric Mean | Geometric CV% | n | Geometric Mean | Geometric CV% |
| C1D22 | 12 | 1 | 0 | 8 | 1 | 0 | 12 | 1 | 0 |
| C3D22 | 7 | 1.3 | 53.33 | 5 | 1.71 | 27.08 | 7 | 1.26 | 41.07 |
| C5D22 | 7 | 1.68 | 42.19 | 5 | 1.32 | 120.31 | 8 | 0.97 | 314.31 |
| C8D22 | 6 | 1.45 | 77.65 | 4 | 1.36 | 23.11 | 6 | 1.42 | 57.14 |
| C12D22 | 8 | 1.22 | 43.22 | 6 | 1.6 | 27.03 | 9 | 0.83 | 165.24 |

PK, pharmacokinetics; Cmax, maximum plasma concentration; AUClast, area under the curve to last timepoint; CV%, coefficient of variation.
