# Supplementary material for: Dalpiciclib combined with pyrotinib and endocrine therapy in women with ER-positive, HER2-positive advanced breast cancer: A prospective, multicenter, single-arm, phase 2 trial
Source: PLoS Med. 2025 Jul 31;22(7):e1004669. doi: 10.1371/journal.pmed.1004669 (PMC12312931; doi:10.1371/journal.pmed.1004669)
Supplement: S2 Table — (DOCX) [file pmed.1004669.s007.docx]

**S2 Table. Results of ^68^Ga-HER2 and 18F-FDG PET/CT.**

| **No.** | **RECIST- 1.1 best response (% change in SoD)** | **Confirmed response** | **Time on treatment in months** | **RECIST 1.1 target and non-target lesions on CT** | **Pre-protocol therapy (^68^Ga-HER2 SUV)** | **After 2 cycles of protocol therapy (^68^Ga-HER2 SUV)** | **Pre-protocol therapy (18F-FDG SUV)** | **After 2 cycles of protocol therapy (18F-FDG SUV)** |
| --- | --- | --- | --- | --- | --- | --- | --- | --- |
| 01018 | -42.9% | PR | 20.3 | Breast | none | Not done | none | Not done |
|  |  |  |  | Sternalis | 2.3 |  | 1.8 |  |
|  |  |  |  | Median SUV | 2.3 |  | 1.8 |  |
|  |  |  |  | Max SUV | 2.3 |  | 1.8 |  |
| 01020 | -57.8% | PR | 30.5 | Breast | none | none | 5.6 | 4.0 |
|  |  |  |  | Bone | 4.2 | 4.0 | 5.6 | 4.3 |
|  |  |  |  | Median SUV | 4.2 | 4.0 | 5.6 | 4.2 |
|  |  |  |  | Max SUV | 4.2 | 4.0 | 5.6 | 4.3 |
| 01021 | -43.5% | SD | 3.5 | Mediastinal lymph nodes | 4.7 | 9.0 | 12.3 | 7.9 |
|  |  |  |  | Hilar lymph nodes | 3.7 | 6.1 | 12.3 | 7.9 |
|  |  |  |  | Bone | 7.0 | 12.9 | 10.6 | 5.9 |
|  |  |  |  | Median SUV | 4.7 | 9.0 | 12.3 | 7.9 |
|  |  |  |  | Max SUV | 7 | 12.9 | 12.3 | 7.9 |
| 01022 | -45.5% | PR | 35.8 | Lung | none | Not done | 1.7 | 0 |
| 01023 | -83.0% | PR | 26.6 | Liver | NE | NE | 10.3 | 0 |
|  |  |  |  | chest wall | 2.3 | 0 | 5.3 | 0 |
|  |  |  |  | Sternalis | 5.3 | 0 | 8.7 | 0 |
|  |  |  |  | Lymph nodes | 2.4 | 0 | 4.2 | 0 |
|  |  |  |  | Pleura | 7.4 | 0 | 4.3 | 0 |
|  |  |  |  | Bone | 4.9 | 3.0 | 7.8 | 3.9 |
|  |  |  |  | Median SUV | 4.9 | 0 | 6.55 | 0 |
|  |  |  |  | Max SUV | 7.4 | 3.0 | 10.3 | 3.9 |
| 01024 | NE | NE | NE | Breast | 5.9 | Not done | 16.2 | Not done |
|  |  |  |  | Axillary lymph nodes | 6.4 |  | 9.1 |  |
|  |  |  |  | Median SUV | 6.15 |  | 12.65 |  |
|  |  |  |  | Max SUV | 6.4 |  | 16.2 |  |
| 01026 | -52.0% | PR | 9.8 | Left breast | 2.7 | Not done | 4.5 | Not done |
|  |  |  |  | Right breast | 5.6 |  | 19.1 |  |
|  |  |  |  | Clavicular lymph nodes | 3.5 |  | 18.1 |  |
|  |  |  |  | Paraaortic lymph nodes | 8.8 |  | 11.4 |  |
|  |  |  |  | Liver | 22.6 |  | 21.9 |  |
|  |  |  |  | Median SUV | 5.6 |  | 18.1 |  |
|  |  |  |  | Max SUV | 22.6 |  | 21.9 |  |
| 01027 | -41.8% | PR | 19.9 | Breast | 7.5 | 6.1 | 10.5 | 9.3 |
|  |  |  |  | Chest wall | 4.1 | 3.7 | 7.4 | 6.2 |
|  |  |  |  | Supraclavicular lymph nodes | 3.6 | 6.0 | 8.1 | 6.7 |
|  |  |  |  | other lymph nodes | 4.5 | 1.6 | 3.9 | 0 |
|  |  |  |  | Bone | 7.3 | 7.2 | 14.8 | 13.3 |
|  |  |  |  | Median SUV | 4.5 | 6 | 8.1 | 6.7 |
|  |  |  |  | Max SUV | 7.5 | 7.2 | 14.8 | 13.3 |
| 01028 | -80.7% | PR | 14.8 | Breast 1 | 3.0 | 0.9 | 5.8 | 0 |
|  |  |  |  | Breast 2 | 4.7 | 0 | 10.9 | 3.2 |
|  |  |  |  | Axillary lymph nodes | 5.1 | 0 | none | 1.5 |
|  |  |  |  | Bone | 10.5 | 3.9 | 10.2 | 4 |
|  |  |  |  | Median SUV | 4.9 | 0.5 | 10.2 | 2.4 |
|  |  |  |  | Max SUV | 10.5 | 3.9 | 10.9 | 4.0 |
| 01029 | -32.6% | SD | 5.4 | Liver | NE | Not done | 8.6 | Not done |
|  |  |  |  | Breast | 2.4 |  | 7 |  |
|  |  |  |  | Chest wall | 2.6 |  | 7.4 |  |
|  |  |  |  | Lymph nodes | 3.1 |  | 11.9 |  |
|  |  |  |  | Bone | none |  | 9.6 |  |
|  |  |  |  | Median SUV | 2.6 |  | 8 |  |
|  |  |  |  | Max SUV | 3.1 |  | 11.9 |  |
| 01030 | -47.0% | PR | 26.0 | Mediastinal lymph nodes | none | Not done | 14.4 | Not done |
| 01031 | -66.7% | PR | 16.6 | Mediastinal lymph nodes | none | none | 8 | 0 |
| 01032 | -36.4% | PR | 11.2 | Lung | none | none | 5.8 | 4.2 |
|  |  |  |  | Lymph nodes | none | none | 7.5 | 8.4 |
|  |  |  |  | Median SUV | none | none | 6.65 | 6.3 |
|  |  |  |  | Max SUV | none | none | 7.5 | 8.4 |
| 01033 | -76.0% | PR | 25.4 | Breast 1 | 5.0 | 3.0 | 26.7 | 6.7 |
|  |  |  |  | Breast 2 | 2.4 | 0.9 | 7.0 | 1.7 |
|  |  |  |  | Axillary lymph nodes | 3.9 | 0 | 1.5 | 1.7 |
|  |  |  |  | Supraclavicular lymph nodes | 13.3 | 0 | 13.3 | 2.5 |
|  |  |  |  | Median SUV | 4.5 | 0.5 | 10.2 | 2.1 |
|  |  |  |  | Max SUV | 13.3 | 3.0 | 26.7 | 6.7 |
| 01034 | -80.9% | PR | 24.8 | Breast | 6.2 | 6.9 | 4.7 | 3.0 |
|  |  |  |  | Lymph nodes | 7.5 | 7.4 | 6.1 | 0.0 |
|  |  |  |  | Median SUV | 6.9 | 7.2 | 5.4 | 1.5 |
|  |  |  |  | Max SUV | 7.5 | 7.4 | 6.1 | 3.0 |
| 01035 | -100.0% | PR | 24.5 | Breast | 34.9 | 0 | 8.4 | 0 |
|  |  |  |  | Lymph nodes | 16.3 | 0 | 6.4 | 0 |
|  |  |  |  | Bone | 27.1 | 0 | 7.8 | 0 |
|  |  |  |  | Median SUV | 27.1 | 0 | 7.8 | 0 |
|  |  |  |  | Max SUV | 34.9 | 0 | 8.4 | 0 |
| 01036 | -86.3% | PR | 19.5 | Breast | 5.2 | 3.3 | 14.6 | 3.9 |
|  |  |  |  | Prepericardial lymph nodes | 5.9 | 0 | 12.5 | 15.0 |
|  |  |  |  | Abdominal lymph nodes | 6.6 | 0 | 16.1 | 4.9 |
|  |  |  |  | Lung | 5.5 | 0 | 13.6 | 2.9 |
|  |  |  |  | Mesentery | 3.6 | 0 | 7.4 | 4.9 |
|  |  |  |  | Bone | 3.7 | 2.8 | 12.2 | 4.3 |
|  |  |  |  | Median SUV | 5.4 | 0.0 | 13.1 | 4.6 |
|  |  |  |  | Max SUV | 6.6 | 3.3 | 16.1 | 15 |
| 01037 | -9.8% | SD | 9.1 | ovary | 5.5 | 14.1 | 12.3 | 9.3 |
|  |  |  |  | Bone | 6.4 | 4.6 | 10 | 4.0 |
|  |  |  |  | Liver | none | 14.9 | 10.9 | 8.2 |
|  |  |  |  | Lymph nodes | none | none | 10.9 | 11.6 |
|  |  |  |  | Median SUV | 6.0 | 14.1 | 10.9 | 8.8 |
|  |  |  |  | Max SUV | 6.4 | 14.9 | 12.3 | 11.6 |
| 01038 | -66.2% | PR | 22.3 | Breast | 5.5 | 4.2 | 19.4 | 4.3 |
|  |  |  |  | Axillary lymph nodes | 4.1 | 0 | 14 | 3.3 |
|  |  |  |  | Bone | 8.4 | 3 | 15.4 | 4.2 |
|  |  |  |  | Median SUV | 5.5 | 3.0 | 15.4 | 4.2 |
|  |  |  |  | Max SUV | 8.4 | 4.2 | 19.4 | 4.3 |
| 01039 | -58.8% | PR | 19.2 | Breast | 11.9 | 0 | 22.1 | 2.0 |
|  |  |  |  | Chest wall | 13.5 | 0 | 12.1 | 0 |
|  |  |  |  | Lymph nodes 1 | 12.8 | 0 | 13.1 | 1.4 |
|  |  |  |  | Lymph nodes 2 | 13.2 | 0 | 12.1 | 0 |
|  |  |  |  | Adrenal gland | 29.9 | 0 | 12.3 | 0 |
|  |  |  |  | Spleen | 11.3 | 0 | 13.2 | 0 |
|  |  |  |  | Paracele | 3.8 | 0 | 20 | 0 |
|  |  |  |  | Bone | 15.2 | 2.6 | 8.5 | 0 |
|  |  |  |  | Median SUV | 13 | 0 | 12.7 | 0 |
|  |  |  |  | Max SUV | 29.9 | 2.6 | 22.1 | 2 |

Highlighted cells indicate patients with reduced ^68^Ga-HER2 affibody uptake to baseline SUV.

Highlighted cells indicate patients with decreased FDG metabolism alongside elevated ^68^Ga-HER2 affibody uptake exhibited stable disease.

PR partial response, CT computed tomography, FDG fluorodeoxyglucose, NE not evaluable, RECIST response evaluation criteria in solid tumors, SD stable disease, SoD sum of the diameters, SUV standardized uptake value.
