# Supplementary material for: Dalpiciclib combined with pyrotinib and endocrine therapy in women with ER-positive, HER2-positive advanced breast cancer: A prospective, multicenter, single-arm, phase 2 trial
Source: PLoS Med. 2025 Jul 31;22(7):e1004669. doi: 10.1371/journal.pmed.1004669 (PMC12312931; doi:10.1371/journal.pmed.1004669)
Supplement: S3 Table — (DOCX) [file pmed.1004669.s008.docx]

**S3 Table. Results of NGS in baseline.**

| **No.** | **Confirmed response** | **Time on treatment in months** | **ctDNA** | **Tissue** |
| --- | --- | --- | --- | --- |
| 01019 | SD | 7.6 | BRCA1 (somatic mutation,missense mutation)  BRCA2 (somatic mutation,nonsense mutation)  ATRX (somatic mutation,nonsense mutation) | Not done |
| 01020 | PR | 30.5 | No mutation detected | Not done |
| 01021 | SD | 3.5 | BRCA2 (germline mutation, frameshift deletion)  CACNA1C (somatic mutation, in-frame deletion)  ESR1 (somatic mutation,missense mutation)  MSH6 (somatic mutation,nonsense mutation)  TSC2 (somatic mutation,missense mutation)  MED12 (somatic mutation, splice site mutation) | Not done |
| 01023 | PR | 26.6 | ERBB2 (somatic mutation,copy number amplification)  MYC (somatic mutation,copy number amplification)  APC (somatic mutation,missense mutation)  JAK1 (somatic mutation,missense mutation)  MET (somatic mutation,missense mutation)  TP53 (somatic mutation,missense mutation) | ERBB2 (somatic mutation,copy number amplification)  FRK (somatic mutation,missense mutation)  NF1 (somatic mutation,missense mutation)  PTK2 (somatic mutation, frameshift deletion)  TP53 (somatic mutation,missense mutation) |
| 01024 | NE | NE | ERBB2 (somatic mutation, missense mutation)  TP53 (germline mutation, missense mutation) | ERBB2 (somatic mutation, missense mutation)  FGFR1 (somatic mutation,copy number amplification)  TP53 (germline mutation, missense mutation)  PRKCD (somatic mutation, missense mutation)  PTPRD (somatic mutation, missense mutation) |
| 01026 | PR | 9.8 | PIK3CA (somatic mutation, missense mutation)  ERBB2 (somatic mutation,copy number amplification)  TP53 (somatic mutation, in-frame deletion) | Not done |
| 01027 | PR | 19.9 | PIK3CA (somatic mutation, missense mutation)  CACNA1C (somatic mutation, missense mutation)  JAK2 (somatic mutation, splice site mutation)  PMS2 (somatic mutation, missense mutation)  TP53 (somatic mutation, frameshift deletion) | Not done |
| 01028 | PR | 14.8 | Not done | ERBB2 (somatic mutation,copy number amplification)  GATA3 (somatic mutation, frameshift insertion)  JUN (somatic mutation, missense mutation) |
| 01031 | PR | 16.6 | Not done | ERBB2 (somatic mutation,copy number amplification)  PIK3CA (somatic mutation, missense mutation)  FGFR1 (somatic mutation,copy number amplification)  TP53(somatic mutation, splicing mutation)  MYC (somatic mutation,copy number amplification)  EPHB4 (somatic mutation, missense mutation)  GATA3 (somatic mutation, missense mutation)  TRIM65 (somatic mutation, missense mutation)  AKT3 (somatic mutation,copy number amplification) |
| 01033 | PR | 25.4 | Not done | ERBB2 (somatic mutation,copy number amplification)  MYC (somatic mutation,copy number amplification)  TP53 (somatic mutation, frameshift deletion)  APC (somatic mutation,nonsense mutation)  ARID1A (somatic mutation,nonsense mutation)  ATM (somatic mutation, missense mutation)  ATN1 (somatic mutation, missense mutation)  BCOR (somatic mutation, missense mutation)  CBLB (somatic mutation, missense mutation)  CDC25B (somatic mutation, missense mutation)  CDH1 (somatic mutation, frameshift deletion)  CUX1 (somatic mutation, missense mutation)  CYP2C8 (somatic mutation, missense mutation)  ESR1 (somatic mutation, missense mutation)  FANCM (somatic mutation, missense mutation)  FOXA1 (somatic mutation, missense mutation)  IRS2 (somatic mutation, frameshift deletion)  KCNJ14 (somatic mutation, missense mutation)  MAP2K4 (somatic mutation, missense mutation)  MECOM (somatic mutation, missense mutation)  NFKB1 (somatic mutation, missense mutation)  NR2F2 (somatic mutation, in-frame deletion)  PREX2 (somatic mutation, missense mutation)  PTCH1 (somatic mutation, missense mutation)  ROCK2 (somatic mutation, missense mutation)  ROS1 (somatic mutation,nonsense mutation)  RPGR (somatic mutation, missense mutation)  TBL1XR1 (somatic mutation, missense mutation)  TET2 (somatic mutation, missense mutation)  USH2A (somatic mutation, missense mutation)  AKT3 (somatic mutation,copy number amplification) |
| 01038 | PR | 22.3 | Not done | ERBB2 (somatic mutation,copy number amplification)  FGFR1 (somatic mutation,copy number amplification)  PIK3CA (somatic mutation, missense mutation)  HIST1H2BC (somatic mutation, missense mutation)  MAP1A (somatic mutation,missense mutation)  MAP3K5 (somatic mutation,missense mutation)  NF1 (somatic mutation, frameshift deletion) |
| 01039 | PR | 19.2 | Not done | ERBB2 (somatic mutation,copy number amplification)  FGFR1 (somatic mutation,copy number amplification)  CCND1 (somatic mutation,copy number amplification)  FANCD2 (somatic mutation,missense mutation)  MECOM (somatic mutation, frameshift deletion)  RAD51D (somatic mutation,missense mutation)  TP53 (somatic mutation, in-frame deletion) |
| 06003 | PR | 27.6 | No mutation detected | ERBB2 (somatic mutation,copy number amplification)  GAB2 (somatic mutation,missense mutation)  NF1 (somatic mutation, frameshift deletion)  PRKCB (somatic mutation,nonsense mutation)  SOX9 (somatic mutation, frameshift deletion) |
| 16001 | PR | 33.2 | Not done | ERBB2 (somatic mutation,copy number amplification)  PIK3CA (somatic mutation, missense mutation)  TP53 (somatic mutation, missense mutation) |
| 16002 | PR | 7.4 | No mutation detected | ERBB2 (somatic mutation,copy number amplification)  ATRX (somatic mutation, missense mutation)  GATA3 (somatic mutation, frameshift deletion)  SPEN (somatic mutation, frameshift deletion)  TBX3 (somatic mutation, frameshift insertion)  PTEN (somatic mutation, deletion) |
| 12001 | PR | 25.8 | PIK3CA (somatic mutation, missense mutation) | Not done |

ctDNA circulating tumor DNA, PR partial response, NE not evaluable, NGS next-generation sequencing technology, SD stable disease.
