# Supplementary material for: Dalpiciclib combined with pyrotinib and endocrine therapy in women with ER-positive, HER2-positive advanced breast cancer: A prospective, multicenter, single-arm, phase 2 trial
Source: PLoS Med. 2025 Jul 31;22(7):e1004669. doi: 10.1371/journal.pmed.1004669 (PMC12312931; doi:10.1371/journal.pmed.1004669)
Supplement: S1 Protocol — (PDF) [file pmed.1004669.s009.pdf]

This file contains both the initial and final versions of the trial protocol in English and Chinese. The English translations are provided alongside the original Chinese versions to ensure transparency, as required by PLOS Medicine.

CLINICAL STUDY PROTOCAL

Pyrotinib, daLpiciclib (SHR6390) and Endocrine therApy in Subjects with dUal-Receptor positive (ER+/HER2+) Advanced Breast cancer: a muLti-center phasE Ib/II study

PLEASURABLE
(LORDSHIPS 3.0/YBCSG-20-01)

VERSION 3.0, 21 December 2022

Xichun Hu，Fudan University Shanghai Cancer Center

TABLE OF CONTENTS

PROTOCAL SYNOPSIS ... 4
SCHEDULE OF EVENTS ... 15
LIST OF ABBREVIATIONS ... 21
1. BACKGROUND ... 23
1.1. INVESTIGATIONAL PRODUCT ... 27
1.2. THE PHARMACOLOGICAL TYPE AND MECHANISM FOR SHR6390 ... 27
1.3. SHR6390 PHARMACODYNAMICS ... 27
1.4. SHR6390 TOXICOLOGY ... 27
1.5. SHR6390 PHARMACOKINETICS ... 28
1.6. IN VIVO ANTITUMOR ACTIVITY OF PYROTINIB AND SHR6390 IN ER+, HER2+ BREAST CANCER MODELS ... 28
2. CLINICAL STUDIES ... 28
2.1. PYROTINIB MALATE ... 28
2.2. SHR6390 ... 28
2.3. MARKETED CDK4/6 INHIBITORS ... 29
3. STUDY OBJECTIVES AND STUDY ENDPOINTS ... 30
3.1. PRIMARY STUDY OBJECTIVE ... 30
3.2. PRIMARY ENDPOINT ... 31
3.3. SECONDARY ENDPOINTS ... 31
4. INVESTIGATIONAL DRUGS ... 32
4.1. NAME AND SOURCE ... 32
4.2. DOSAGE FORM AND SPECIFICATIONS ... 32
4.3. STORAGE ... 32
4.4. ADMINISTRATION ... 32
4.5. MANAGEMENT, DISTRIBUTION, AND RETRIEVAL ... 33
5. OVERALL STUDY DESIGN ... 33
6. STUDY DESIGN ... 33
6.1. DOSAGE AND ADMINISTRATION ... 33
6.2. DOSE-LIMITING TOXICITY (DLT) ... 35
6.3. MAXIMUM TOLERATED DOSE (MTD) ... 36
6.4. SUBJECT REPLACEMENT ... 36
6.5. PLANNED SAMPLE SIZE ... 36
7. SAMPLE COLLECTION ... 36
8. PARTICIPANTS ... 37
8.1. SUBJECTS AND SAMPLE SIZE ... 37
8.2. INCLUSION CRITERIA ... 38
8.3. EXCLUSION CRITERIA ... 39
8.4. PARTICIPANT IDENTIFICATION ... 41

8.5. DROPOUT CRITERIA 41
8.6. TERMINATION CRITERIA FOR PARTICIPANT TREATMENT 41
8.7. STUDY TERMINATION CRITERIA 41
9. DOSE ADJUSTMENT AND CONCOMITANT MEDICATION 42
9.1. DLT OBSERVATION PERIOD 42
9.2. CONTINUOUS DOSING IN CYCLE 2 AND SUBSEQUENT CYCLES 42
9.3. PROHIBITED MEDICATIONS 43
9.4. PERMITTED MEDICATIONS 43
10. STUDY PROCEDURES 44
10.1. SCREENING PERIOD 44
10.2. TRIAL PERIOD 46
10.3. STUDY TERMINATION/WITHDRAWAL 47
10.4. FOLLOW-UP AFTER TREATMENT COMPLETION 47
11. SAFETY ASSESSMENT 48
11.1. ADVERSE EVENT (AE) 48
11.2. SERIOUS ADVERSE EVENTS (SAE) 51
12. SERIOUS ADVERSE EVENT REPORTING SYSTEM 52
13. SAE REPORTING PROCEDURES 53
14. EFFICACY EVALUATION 53
15. STUDY COMPLETION 53
16. SAFETY CONTROL MEASURES 54
17. RECOMMENDED PHASE II CLINICAL TRIAL DOSING REGIMEN 55
18. ETHICAL STANDARDS AND INFORMED CONSENT 55
18.1. ETHICAL STANDARDS 55
18.2. INFORMED CONSENT 55
19. QUALITY ASSURANCE IN CLINICAL TRIALS 56
20. DATA HANDLING 56
20.1. RESEARCHER DATA ENTRY REQUIREMENTS 56
20.2. DATA TRACEABILITY AND CRF COMPLETION 56
20.3. DATA SELECTION FOR ANALYSIS 57
20.4. UNDERGOING OTHER ANTINEOPLASTIC TREATMENTS 57
REFERENCES 59
APPENDIX 1: PERFORMANCE STATUS RATING SCALE (ECOG) 60
APPENDIX 2: CREATININE CLEARANCE RATE (CRCL) 61
APPENDIX 3: RESPONSE EVALUATION CRITERIA IN SOLID TUMORS 62

PROTOCAL SYNOPSIS

Study Title	Pyrotinib, dalpiciclib (SHR6390) and endocrine therapy in subjects with dual-receptor positive (ER+/HER2+) advanced breast cancer:<br>a multi-center phase Ib/II study
Protocal Number	PLEASURABLE (LORDSHIPS 3.0/YBCSG-20-01)
Version	3.0
Version Date	21 December 2022
Lead Institution	Fudan University Shanghai Cancer Center
Principal investigator	Professor Hu Xichun
Coordinating investigator	Professor Zhang Jian, Dr. Meng Yanchun, Dr. Tao Zhonghua
Indication Under Investigation	ER receptor-positive, HER2-positive advanced breast cancer
Study Objectives	Phase I (Ib stage):<br>Determine the safety and tolerability of the combination of a non-steroidal aromatase inhibitor (letrozole), pyrotinib maleate tablets, and the CDK4/6 inhibitor SHR6390 in the treatment of hormone receptor-positive, HER2-positive advanced breast cancer. Based on preliminary efficacy data, establish the recommended Phase II dosage for this combination therapy.<br>Phase II:<br>To evaluate the efficacy and safety of pyrotinib and dalpiciclib (SHR6390) combined endocrine therapy in double-receptor-positive (ER+, HER2+) advanced breast cancer.

Study Endpoints	Phase I<br><br>Primary Endpoint:<br>• For the combination regimen, determine the dose-limiting toxicity (DLT) and maximum tolerated dose (MTD) of SHR6390, establishing the recommended dosing regimen for the Phase II clinical study.<br>• Assess the incidence and severity of adverse events (AEs) and serious adverse events (SAEs) across different dose groups.<br><br>Secondary Efficacy Endpoints:<br>• Objective response rate (ORR), according to RECIST 1.1<br>• Progression-free survival (PFS)<br>• Disease Control Rate (DCR, The proportion of subjects with a best overall response of complete response, partial response, or stable disease).<br>• Clinical benefit rate (CBR, proportion of complete response, partial response, or stable disease for at least 24 weeks)<br>• Duration of response (DOR)<br>• Pharmacokinetics<br>• AEs and SAEs<br><br>Phase II:<br><br>Primary Endpoint:<br>• Objective response rate (ORR), according to RECIST 1.1<br><br>Secondary Efficacy Endpoints:<br>• Progression-free survival (PFS)<br>• Disease Control Rate (DCR, The proportion of subjects with a best overall response of complete response, partial response, or stable disease).<br>• Clinical benefit rate (CBR, proportion of complete response, partial response, or stable disease for at least 24 weeks)<br>• Duration of response (DOR)<br>• Exploratory analysis of the relationship between molecular markers and efficacy<br>• Pharmacokinetics<br>• AEs and SAEs
Planned Sample Size	Phase I (Ib stage): Each dose group will include 3－6 subjects, with a total of 6－12 subjects in Groups A and B. If additional backup dose groups C, D, E, or F are used, each of these groups will add 3－6 more subjects.

	Phase II: A total of 48 subjects will receive the combination of pyrotinib and SHR6390 with endocrine therapy (letrozole or fulvestrant).
Study Design	Phase I (Ib stage): A single-arm, open-label, dose-escalation Ib clinical study.<br><br>Phase II: A single-arm, open-label, multicenter Phase II clinical study.
Study Treatment	Phase I (Ib stage):<br>In this study, the dosing regimen includes letrozole at 2.5 mg/day or anastrozole at 1 mg/day, with pyrotinib at either 400 mg/day or 320 mg/day. SHR6390 is organized into three dose groups.

Dose groups	SHR6390	pyrotinib	Patients
A	125 mg/d	400 mg/d	3~6
B	150 mg/d	400 mg/d	3~6
C(backup group)	100 mg/d	400 mg/d	3~6
D(backup group)	125 mg/d	320 mg/d	3~6
E(backup group)	150 mg/d	320 mg/d	3~6
F(backup group)	100 mg/d	320 mg/d	3~6

In the regimen combining non-steroidal aromatase inhibitors (NSAIs), pyrotinib maleate tablets, and SHR6390, the starting dose for SHR6390 is set at 125 mg. Following a 3+3 dose-escalation design, subsequent doses will be adjusted by increments of 25 mg based on the occurrence of dose-limiting toxicity (DLT) at the starting dose group:

- If no DLT at the specified frequency is observed in the initial dose group (125 mg, Group A), the dose will be escalated to the 150 mg group. If the 150 mg group exhibits DLT at the specified frequency, then the 125 mg dose will be established as the maximum tolerated dose (MTD).
- If the initial dose group (125 mg, Group A) shows DLT at the specified frequency, investigators will initiate both backup groups C and D. If C is also intolerable, backup Group F will be initiated. Groups D and E will be introduced sequentially (using the same dosing escalation principles), and if Group D remains intolerable, backup Group F will be implemented. The research team will

analyze the data to determine the recommended Phase II dosing for the combination therapy.

- If Group F remains intolerable, the research team will analyze the trial data and decide whether to terminate the study.
- Once the recommended Phase II dose for the letrozole, pyrotinib maleate, and SHR6390 combination therapy has been established, a parallel group using anastrozole, pyrotinib maleate, and SHR6390 will be initiated. If intolerable toxicity is observed in this group, dose adjustments will be made after discussion among the researchers.

DLT assessments will be conducted at the end of one treatment cycle for each dose group.

The specific dosing regimens for the three drugs are as follows:

- Non-steroidal Aromatase Inhibitor (NSAI): Letrozole, taken orally, 2.5 mg (or 1 mg), once daily on an empty stomach, with a 28-day cycle.
- Pyrotinib: Taken orally at 400 mg or 320 mg once daily, within 30 minutes after breakfast, with a 28-day cycle.
- SHR6390: Taken orally once daily on an empty stomach (ensuring fasting for at least 1 hour before and after dosing). The drug is administered on a 28-day cycle, with continuous dosing during the first 3 weeks of each cycle (D1－21), concurrent with letrozole, followed by a week of rest (D22－28).

The initial two cycles are designated as the core trial phase. Subjects evaluated with CR, PR, or SD at the end of the second cycle may continue on the assigned combination regimen until disease progression, intolerable toxicity, or withdrawal of informed consent.

Based on the recommended phase 2 dose established in phase 1, proceed with the phase 2 dose expansion study using pyrotinib 320mg and dalpiciclib (SHR6390) 125mg in combination with endocrine therapy. Continue administration until the occurrence of disease progression, intolerable toxicity, or voluntary withdrawal of informed consent by the participant.

The specific regimen for the three drugs is as follows:

- Endocrine therapy:
    - ➢ For patients not proven to be resistant to aromatase inhibitors (AI) (primary and secondary): Letrozole, orally, 2.5 mg, once daily, taken on an empty stomach, administered continuously for 28 days as one cycle.
    - ➢ For aromatase inhibitor resistance (primary and secondary): Fulvestrant 500mg, D1 (additional administration on D15 of the first cycle), intramuscular injection, one cycle lasts 28 days.
- Pyrotinib: Orally, 320 mg, once daily, taken within 30 minutes after breakfast, continuously administered for 28 days as one cycle.
- Dalpiciclib (SHR6390): Administered orally at 125mg once daily on an empty stomach (fasting required for at least 1 hour before and 1 hour after taking the medication), continuously for 21 days, followed by a seven-day off period, with each cycle lasting 28 days.

Study Design Schema:

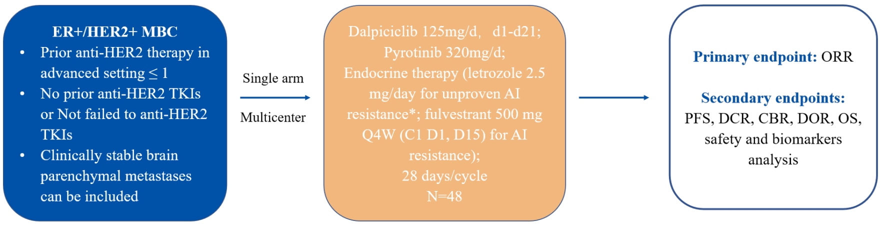

*Resistance includes both primary and secondary resistance.

Dose-limiting toxicity (DLT)

DLT is defined as any of the following drug-related or possibly drug-related adverse events occurring within Cycle 1 (based on CTC-AE v4.0.3 criteria):

1. Hematologic Toxicity:

- Grade 4 neutropenia lasting ≥5 days;
- Grade 4 thrombocytopenia or Grade 3 thrombocytopenia with clinically significant bleeding;
- Grade ≥3 neutropenia with fever (≥38.0° C lasting 1 hour or >38.3° C);

	• Grade ≥4 anemia.<br>2. Non-Hematologic Toxicity:<br>• Any Grade ≥3 non-hematologic toxicity, except for the following:<br>• Grade 3–4 nausea/vomiting and/or diarrhea and/or electrolyte imbalance that resolves to Grade ≤2 within 72 hours with optimal supportive treatment;<br>- Grade 3–4 elevations in alkaline phosphatase or gamma-glutamyl transferase clearly related to the tumor and unrelated to the drug.
Maximum tolerated dose (MTD)	Definition of Maximum Tolerated Dose (MTD):<br>During the dosing observation period in Cycle 1 (28 days), if DLTs are observed in ≥1/3 of the subjects in a given dose group, the previous dose group is determined as the MTD. If dose escalation reaches Group B in the Phase I trial without ≥1/3 of the subjects experiencing DLTs, the research team will discuss whether to initiate the backup dose group C and determine the recommended dose for the Phase II trial.
Inclusion Criteria	1. Participants voluntarily join the study, sign an informed consent form, and demonstrate good compliance.<br>2. Female patients aged ≥18 and ≤75, either postmenopausal or pre/perimenopausal, meeting one of the following:<br>a) Previous bilateral oophorectomy, or aged ≥60; or<br>b) Aged <60, naturally postmenopausal (defined as at least 12 consecutive months of amenorrhea without other pathological or physiological causes), with postmenopausal levels of E2 and FSH; or<br>c) Pre- or perimenopausal women also eligible but must be willing to undergo LHRH agonist treatment during the study.<br>3. Patients with histologically confirmed recurrent/metastatic breast cancer, ER-positive and HER2-positive:<br>- HER2 positivity defined as 3+ by standard immunohistochemistry (IHC) and/or positive by in situ hybridization (ISH).

- ER positivity defined as ≥1% ER-expressing cells.
- Local recurrence must be confirmed by the researcher as inoperable.

4. At least one measurable extracranial lesion according to RECIST 1.1 criteria.

5. Previous antitumor treatment guidelines:

- Maximum of one prior systemic therapy regimen for recurrent/metastatic breast cancer including anti-HER2 ADC:

  i. If not previously treated with a trastuzumab regimen in the advanced stage, or if recurrence occurs more than one year after adjuvant trastuzumab treatment, subsequent therapy is considered first-line anti-HER2 treatment.

  ii. If first-line treatment with a trastuzumab regimen fails, or if recurrence occurs during adjuvant trastuzumab treatment or within one year after its completion, subsequent therapy is considered second-line anti-HER2 treatment.

- No prior anti-HER2 TKI therapy or no proof of failure with such therapy.
- Previous endocrine therapy not proven resistant to aromatase inhibitors (Resistance defined as recurrence within one year after completing adjuvant aromatase inhibitor therapy or disease progression during the recurrent/metastatic phase after aromatase inhibitor treatment); subsequent endocrine therapy with letrozole. If aromatase inhibitor resistance is present, subsequent endocrine therapy with fulvestrant.

6. ECOG performance status of 0－1.

7. Expected survival of ≥12 weeks.

8. Adequate organ function (no use of any blood components and growth factors within 2 weeks prior to enrollment):

- Absolute neutrophil count ≥1.5×10^9/L;
- Platelets ≥90×10^9/L;
- Hemoglobin ≥90g/L;
- Total bilirubin ≤1.5 times upper limit of normal (ULN);
- ALT and AST ≤2.5 times ULN;

	- Urea/Blood Urea Nitrogen (BUN) and creatinine (Cr) ≤1.5×ULN;<br>- Left ventricular ejection fraction (LVEF) ≥50%;<br>- Fridericia-corrected QT interval (QTcF) < 470 ms;<br>- INR ≤1.5×ULN, APTT ≤1.5×ULN.
Exclusion Criteria	1. Brain meningeal metastasis or active brain parenchymal metastasis. Clinically stable brain parenchymal metastasis patients may be included, including asymptomatic brain metastasis without prior local treatment; or patients who have received CNS metastasis treatment (radiation or surgery) confirmed stable by imaging for at least 4 weeks, and who have ceased symptomatic treatment (including steroids and mannitol) for more than 2 weeks.<br>2. Prior treatment with any CDK4/6 inhibitor.<br>3. Presence of symptomatic ascites, pleural effusion, or pericardial effusion requiring drainage at baseline, or who have undergone serous cavity fluid drainage within 4 weeks prior to first drug administration.<br>4. Inability to swallow, intestinal obstruction, or other factors affecting drug intake and absorption.<br>5. Received chemotherapy, molecular targeted therapy, or other systemic treatments including clinical trial drugs within 4 weeks prior to enrollment; received endocrine therapy within 2 weeks prior to enrollment.<br>6. History of other malignancies within the past 5 years or concurrent malignancy, except for cured basal cell carcinoma of the skin and cervical carcinoma in situ.<br>7. Major surgical procedures or significant trauma within 4 weeks prior to the first drug administration, or anticipated need for major surgery during the study.<br>8. Pregnant or breastfeeding women, women of childbearing potential with a positive baseline pregnancy test, or unwilling to use effective contraception.<br>9. Known hypersensitivity to any component of the study drugs.

	10. Active HBV or HCV infection; stable hepatitis B (HBV viral copy number not exceeding the upper limit of normal) treated with medication and cured hepatitis C (HCV viral copy number below the detection limit of the assay) are excluded.<br>11. History of immunodeficiency, including HIV positivity, or any other acquired or congenital immunodeficiency diseases, or history of organ transplantation.<br>12. History of any cardiac disease, including: (1) angina; (2) arrhythmias requiring medication or clinically significant; (3) myocardial infarction; (4) heart failure; (5) any other heart disease deemed by the investigator as unsuitable for participation in the trial; severe cardiac or renal function abnormalities of grade ≥II found during the screening period.<br>13. According to the investigator's judgement, severe concomitant diseases that pose a risk to patient safety or affect the completion of the study (e.g., severe hypertension, diabetes, thyroid disorders).<br>14. History of definite neurologic or psychiatric disorders, including epilepsy or dementia.<br>15. Severe infection within 4 weeks prior to the first drug administration (e.g., requiring intravenous administration of antibiotics, antifungals, or antivirals according to clinical guidelines), or unexplained fever >38.3°C during the screening period or prior to the first administration.
Termination Criteria for Participant Treatment	If any of the following conditions occur, the participant must withdraw/terminate treatment:<br>1. The participant withdraws informed consent and requests to exit.<br>2. Imaging examination shows disease progression.<br>3. Inability to tolerate toxicity.<br>4. Serious protocol violation, as assessed by the investigator warranting treatment termination.<br>5. Participant loss to follow-up or occurrence of a pregnancy event.<br>6. Other circumstances deemed necessary for participant withdrawal from the study by the investigator.
Study	Study termination criteria include, but are not limited to:

Termination Criteria	1. Discovery of unexpected, significant, or unacceptable risks to participants;<br>2. The investigational drug/therapy is ineffective, or continuation of the trial is deemed futile;<br>3. The investigator decides to terminate the study due to reasons such as severe lag in participant enrollment or significant protocol violations.
Safety Assessment	Adverse events will be graded according to the CTCAE v4.0.3 standards. During the trial, adverse event logs must be accurately maintained, including the time of occurrence, severity, duration, measures taken, and outcomes of each event.
Efficacy Evaluation	Efficacy evaluations for enrolled participants involve imaging assessments every 2 cycles (±7 days) for the first 6 cycles and then every 3 cycles (±7 days) thereafter, continuing until disease progression or the initiation of new antitumor treatment. Tumor response will be assessed according to RECIST 1.1 criteria. Following disease progression or the start of new antitumor treatment, survival status will be followed up every 12 weeks.
Phase 2 Exploratory Study (Lead Institution or Conditional Participating Institutions Only)	HER2-PET and FDG-PET<br>Patients will undergo HER2-PET and FDG-PET assessments at baseline, after 2 cycles, and upon progression to further explore their clinical utility in evaluating antitumor efficacy.<br>2. [redacted]<br>3. Circulating tumor DNA (ctDNA)<br>ctDNA will be collected at baseline, at the end of cycle 2, and prior to disease progression or the initiation of new antitumor treatment to serve as a biomarker for assessing antitumor efficacy.
Statistical Analyses	The primary analysis of this trial will primarily utilize descriptive statistical methods. For continuous data, mean, standard deviation, median, maximum, and minimum values will be presented. For categorical and ordinal data, frequencies (relative frequencies), rates, and confidence intervals will be provided.<br>All statistical analyses will be conducted using SAS 9.2 or above

	statistical analysis software.<br>Safety Analysis:<br>Descriptive statistics will be used to analyze adverse events, serious adverse events, and adverse events related to the investigational drug in each dose group. Laboratory test results will describe the occurrence of abnormalities after treatment compared to normal values before the trial.<br>Efficacy Analysis:<br>Point estimates of objective response rate (ORR), disease control rate (DCR), clinical benefit rate (CBR), and other efficacy endpoints will be provided along with their 95% confidence intervals, representing the population. Survival outcomes will be assessed using Kaplan-Meier methods to estimate median progression-free survival, 12-month survival rates, and their 95% confidence intervals, with survival curves plotted. Descriptive analysis will be applied to other secondary efficacy endpoints.<br>Other Analyses:<br>The relationship between levels of molecular biomarkers that may affect efficacy and treatment outcomes will be explored.
Study Completion	The study will conclude 24 months after the last participant is enrolled or earlier if deemed necessary by the investigator.

Schedule of Events

<table>
<tr><th rowspan="3">Procedures</th><th colspan="2">Screening period</th><th colspan="4">Treatment and Follow-up Period 28d/cycle</th><th colspan="2" rowspan="2">Post-treatment</th><th rowspan="2">Survival Follow-up</th></tr>
<tr><th></th><th></th><th colspan="3">Cycle 1</th><th>≥ Cycle 2</th></tr>
<tr><th>Day-28 to Day-1</th><th>Day-7 to Day-1</th><th>Day15 (±3d)</th><th>Day21 (±3d)</th><th>Day28 (±3d)</th><th>Day28 (±3d)</th><th>End of treatment/withdrawal</th><th>4 weeks after the last dose</th><th>every 12 weeks (±7d)</th></tr>
<tr><td colspan="10">Baseline data</td></tr>
<tr><td>Signed informed consent</td><td>×</td><td></td><td></td><td></td><td></td><td></td><td></td><td></td><td></td></tr>
<tr><td>Demographic data</td><td>×</td><td></td><td></td><td></td><td></td><td></td><td></td><td></td><td></td></tr>
<tr><td>Cancer History/other medical history[1]</td><td>×</td><td colspan="8"></td></tr>
<tr><td>Concomitant medication[2]</td><td>×</td><td></td><td colspan="6">×</td><td></td></tr>
<tr><td colspan="10">Laboratory examination</td></tr>
<tr><td>Complete Blood Count (CBC)[3]</td><td></td><td>×</td><td>×</td><td></td><td>×</td><td>×</td><td>If not done within 7 days</td><td>In necessity</td><td></td></tr>
<tr><td>Urinalysis[4]</td><td></td><td>×</td><td colspan="4">Every 3 weeks</td><td>If not done within 7 days</td><td>In necessity</td><td></td></tr>
<tr><td>Fecal Routine[5]</td><td></td><td>×</td><td colspan="4">Every 3 weeks</td><td>If not done within 7</td><td>In necessity</td><td></td></tr>
</table>

Procedures	Screening period		Treatment and Follow-up Period 28d/cycle				Post-treatment		Survival Follow-up
	Day-28 to Day-1	Day-7 to Day-1	Cycle 1			⩾ Cycle 2			
			Day15 (±3d)	Day21 (±3d)	Day28 (±3d)	Day28 (±3d)	End of treatment/withdrawal	4 weeks after the last dose	every 12 weeks (±7d)
							days		
Blood Biochemistry[6]		×	×		×	×	If not done within 7 days	In necessity	
Hepatitis B, Hepatitis C, and HIV Testing[7]	×								
Pregnancy Test[8]		×						In necessity	
	Clinical evaluation and examination								
Adverse Events[9]	From the signing of the informed consent to 28 days after the last dose								
Vital Signs[10]		×	×		×	×	If not done within 7 days	×	
Physical Examination[11]		×	×		×	×	If not done within 7 days	×	
ECOG PS		×	×		×	×	If not done within 7 days	×	
12-Lead Electrocardiogram (ECG)[12]		×	×		×	×	If not done within 7 days	In necessity	
Echocardiogram[13]	×		Every 3 weeks				If not done within 4	In necessity	

Procedures	Screening period		Treatment and Follow-up Period 28d/cycle				Post-treatment		Survival Follow-up
	Day-28 to Day-1	Day-7 to Day-1	Cycle 1			≥ Cycle 2			
			Day15	Day21	Day28	Day28	End of treatment/withdrawal	4 weeks after the last dose	every 12 weeks
			(±3d)	(±3d)	(±3d)	(±3d)			(±7d)
							weeks		
	Research drugs								
Endocrine Therapy[14]									
Pyrotinib[15]			Take orally once daily within 30 minutes after meals						
SHR6390 (dalpiciclib)[16]			Take orally once daily on an empty stomach						
	Efficacy evaluation								
Imaging examination[17]	×		Imaging evaluation was performed at the end of every 2 cycles (±7 days) and at the end of every 3 cycles (±7 days) after 6 cycles until the disease progressed or a new anti-tumor therapy was initiated						
[redacted][18]	×		[redacted]						
HER2-PET[19]	×		After the second cycle of treatment (±7 days) and after disease progression						
FDG-PET[19]	×		After the second cycle of treatment (±7 days) and after disease progression						
	Follow-up after treatment								

Procedures	Screening period		Treatment and Follow-up Period 28d/cycle				Post-treatment		Survival Follow-up
	Day-28 to Day-1	Day-7 to Day-1	Cycle 1			≥ Cycle 2			
			Day15 (±3d)	Day21 (±3d)	Day28 (±3d)	Day28 (±3d)	End of treatment/withdrawal	4 weeks after the last dose	every 12 weeks (±7d)
Disease Progression Timing[20]							Until the disease progresses or a new anti-tumor therapy is initiated		
Survival Follow-Up[21]									×
	Blood collection and tumor sample collection/collection								
PK Collection[22]				×					
Biomarker Collection/Gathering[23]	×					×(Day 28 ±3 days of Cycle 2)	×(Before disease progression or start of new anti-tumor therapy)		

Notes:

[1] Cancer History/Other Medical History: Includes pathology results, ER/PR/HER2 testing reports; history of cancer surgery, chemotherapy, radiation therapy, and other disease treatments; history of cancers other than breast cancer.

[2] Concomitant Medication: Record medications and treatments used within 28 days prior to starting the study medication and during the study period. Once a participant discontinues trial treatment, only record concomitant medications and treatments used for new or unresolved adverse events related to the trial treatment。

[3] Complete Blood Count (CBC): Hemoglobin, red blood cells, white blood cells, neutrophils, lymphocytes, and platelet count.

[4] Urinalysis: Urine protein, glucose, occult blood (red cells, white cells). If semi-quantitative methods show protein 2+, a 24-hour urine protein quantitative test should be performed.

[5] Fecal Routine: Includes fecal occult blood.

[6] Blood Biochemistry: Total bilirubin, conjugated bilirubin, ALT, AST, AKP, γ-GT, LDH, total protein, albumin, urea/urea nitrogen, creatinine, uric acid, fasting glucose, triglycerides, cholesterol, potassium, sodium, chloride, calcium, phosphorus, magnesium; myocardial enzyme spectrum tests added as necessary.

[7] Hepatitis B, Hepatitis C, and HIV Testing: Hepatitis B five-marker test, and if results are abnormal, viral replication (HBV DNA) testing should be performed; Hepatitis C virus antibodies (anti-HCV), HIV antibodies.

[8] Pregnancy Test: Serum pregnancy test within one week before first medication use for women of childbearing age.

[9] Adverse Events: Record adverse events from the signing of the informed consent until at least 28 days after the last medication dose, and follow-up until adverse events have resolved or stabilized. If the participant begins new antitumor treatment, follow-up continues until the start of the tumor treatment.

[10] Vital Signs: Temperature, respiration, pulse, blood pressure.

[11] Physical Examination: Examination of major body systems (head, face, skin system, lymph nodes, eyes, ENT, oral cavity, respiratory system, cardiovascular system, abdomen, genitourinary system, musculoskeletal, nervous system, and mental status); comprehensive physical examination results recorded during screening and at the end of the study, with only abnormalities recorded during the trial.

[12] 12-Lead Electrocardiogram (ECG): If clinically significant abnormalities are found, the investigator may reconfirm if necessary.

[13] Echocardiogram: Follow-up on changes in LVEF values, additional unplanned checks if LVEF decreases to <50% and drops ≥10% from baseline, or if symptoms such as chest pain or palpitations occur.

[14] Endocrine Therapy: 1) For unproven aromatase inhibitor resistance (primary and secondary): Letrozole, oral, 2.5 mg, once daily, on an empty stomach, continuous administration for 28 days per cycle. 2) Aromatase inhibitor resistance (primary and secondary): Fulvestrant 500mg, D1 (additional dose on D15 of the first cycle), intramuscular injection, 28 days per cycle.

[15] Pyrotinib: Once daily, 320 mg, oral administration within 30 minutes after breakfast, continuous administration for 28 days per cycle.

[16] SHR6390 (dalpiciclib): Oral administration on an empty stomach, once daily, 125 mg, medication for 3 weeks (D1~21), off drug for 1 week (D22~28), 28 days per cycle. For SHR6390, take on an empty stomach in the morning with warm water; no eating 1 hour before and after taking the medication during the continuous administration period.

[17] Imaging examination: Imaging examination during the screening period include enhanced CT or MRI of the chest and abdomen, and CT/MRI of other suspected lesion sites (such as neck, pelvis, or brain). Tumor baseline assessment may be relaxed to within 4 weeks before the first medication dose, and CT/MRI results obtained before signing the informed consent can be used for tumor assessment during the screening period if they meet the requirements. Bone scans are necessary when clinical suspicion of bone metastasis exists. Imaging examination during the treatment period should be conducted under the same conditions as the baseline studies (scan slice thickness, contrast agent use, etc.), before the 6th cycle of medication, once every two cycles for lesions identified at baseline (bone scans conducted when bone progression is suspected or for CR confirmation), then every three cycles thereafter; if new lesions are suspected, timely checks are appropriate. Initial PR/CR should be confirmed 4-6 weeks later. The imaging examination schedule allows a window period of ±7 days. Unplanned imaging studies may be conducted if disease progression (e.g., worsening symptoms) is suspected.

[18] [redacted]

[19] HER2-PET and FDG-PET: Performed at baseline, end of cycle 2, and at disease progression (available at lead and participating sites with the capability).

[20] Disease Progression Timing: For participants ending trial treatment for reasons other than confirmed imaging progression, if imaging evaluation has not been performed within 4 weeks prior to the end of the trial, imaging should be conducted at the end of treatment, and tumor response follow-up should continue according to the protocol's specified frequency until documented disease progression or the start of a new antitumor treatment.

[21] Survival Follow-Up: After the end of trial treatment, survival status and subsequent antitumor treatment information may be collected every 3 months via clinical or telephone follow-up, until death.

[22] PK Collection: In Phase II, collect plasma within 0.5 hours before pyrotinib dosing on day 21 of the 1st cycle and 2 hours ±5 min, 4 hours ±10 min, 6 hours ±10 min, 12 hours ±10 min, 24 hours ±0.5 hour after dosing, and before dosing on day 22 of cycles 3, 5, 8, and 12, with medication times fixed relative to 3 days before PK blood collection.

[23] Biomarker Collection/Gathering: At baseline, end of cycle 2, and before disease progression/new antitumor treatment, collect [redacted] and ctDNA samples; collect existing paraffin-embedded tumor tissue samples or ≥10 slides, and attempt to obtain biopsy samples from metastatic lesions

LIST OF ABBREVIATIONS

ABBREVIATION	DEFINITION
ALT	alanine aminotransferase
AST	aspartate aminotransferase
Cr	creatinine
CR	complete response
CRF	case report form
CDK	cyclin-dependent kinase
CYP	cytochrome P450
bid	twice a day
BUN	blood urea nitrogen
dL	deciliter
EC	ethics committee
ECG	electrocardiogram
ECOG	Eastern Oncology Collaboration Group
EGFR	epidermal growth factor receptor
ER	estrogen receptor
g	gram
GCP	Good Clinical Practice
GGT	glutamyltransferase
h	hour
Hb	hemoglobin
HER2	human epidermal growth factor receptor-2
HR	hormone receptor
IB	Investigator's Brochure
ISH	immunohistochemistry
INR	International Normalized Ratio
IU	international unit
IV	intravenous(ly)

ABBREVIATION	DEFINITION
kg	kilogram
kPa	kilopascal
LDH	interstitial lung disease
m	meter
min	minutes
mg	milligram
mL	milliliter
mm	millimeter
MBC	metastatic breast cancer
MTD	maximum tolerated dose
NCI-CTC	National Cancer Institute General Toxicity Criteria
ORR	objective response rate
PDX	patient-derived xenografts
PFS	progression-free survival
PLT	platelet
PR	partial response
qd	once a day
RBC	red blood cell count
SAE	serious adverse event
SAP	statistical analysis plan
TBIL	total bilirubin
UNL	upper limit of normal
WBC	white blood cell count

1. Background

Breast cancer has become the most common malignant tumor among women worldwide, accounting for approximately 25% of all malignant tumors in women. Early-stage breast cancer can be cured, but the median overall survival (OS) of patients with Metastatic Breast Cancer (MBC) is only 2 to 3 years. Studies have shown that breast cancer is a highly heterogeneous disease at the molecular level, and there are significant differences in treatment efficacy and survival among different molecular subtypes of breast cancer. Breast cancer subtyping guides treatment selection and prognosis. The 12th St Gallen Conference expert panel classified breast cancer into four subtypes: Luminal A, Luminal B, HER2-positive, and Triple-negative. Among them, Luminal B can be further divided into Luminal B (HER2-negative) and Luminal B (HER2-positive) based on HER2 status. Research has shown that endocrine therapy combined with anti-HER2 targeted therapy is an effective option for treating Luminal B (HER2-positive) MBC patients.

The epidermal growth factor receptor (EGFR) is a family of transmembrane receptors with tyrosine kinase activity, which includes HER1 (erbB1, EGFR), HER2 (erbB2, NEU), HER3 (erbB3), and HER4 (erbB4). The HER2 gene is amplified/overexpressed in more than 30% of human tumors, including breast cancer, ovarian cancer, endometrial cancer, and others. In the clinical diagnosis and treatment of breast cancer, HER2 is an important prognostic factor distinct from tumor size, lymph nodes, and hormone receptors, and is also an independent prognostic factor for breast cancer recurrence and survival. 20% to 30% of primary invasive breast cancers have amplification/overexpression of the HER2 gene, and targeted HER2 molecular drugs significantly prolong the survival of HER2-positive patients. In 1998, Roche's trastuzumab (Herceptin) was launched in the United States, resulting in significant improvements in clinical response rate and survival for HER2-positive patients. Over a decade later, Roche developed pertuzumab (Perjeta) and T-DM1 (ado-trastuzumab emtansine), which were approved for marketing in the United States in 2012 and 2013 respectively, for second- and third-line treatment after trastuzumab resistance. While there have been rapid advances in the development of large molecular HER2 antibodies, lapatinib (TYKERB), the first small molecule inhibitor targeting HER2, developed by GlaxoSmithKline, was launched in the United States in 2007 and in China in 2013, and used in combination with capecitabine for second-line treatment of HER2-positive advanced breast cancer. Meanwhile, neratinib (Nerlynx), a small molecule inhibitor of EGFR/HER2 for breast

cancer treatment developed by Puma Biotechnology, was approved for marketing in 2017.
Pyrotinib maleate tablets, independently developed by Jiangsu Hengrui Medicine Co., Ltd., is an irreversible dual-target tyrosine kinase inhibitor against EGFR and HER2. Clinical trial data for stage II HER2-positive advanced or metastatic breast cancer patients who have failed anthracycline and taxane therapy and have received no more than 2 lines of chemotherapy, show that the combination of pyrotinib maleate tablets with capecitabine significantly improves the objective response rate (78.5% vs. 57.1%) and significantly prolongs progression-free survival (18.1 months vs. 7.0 months), reducing the risk of disease progression or death by 63.7% (HR=0.363), with good tolerability. Based on the efficacy and safety data obtained from current phase II clinical trials, the China National Medical Products Administration has agreed to accept Jiangsu Hengrui Medicine's application for conditional approval of pyrotinib maleate tablets for marketing.

Cyclin-dependent kinases (CDKs) are key enzymes in the regulation of the cell cycle, involved in physiological processes such as cell proliferation and survival. During cell proliferation, the complex formed by CDK4/6 and cellular cyclin D can phosphorylate the retinoblastoma protein (Rb). Once phosphorylated, Rb releases the transcription factor E2F, which, when bound in its unphosphorylated state, is tightly bound. The activation of E2F further promotes transcription that drives the cell cycle past the restriction point (R-point) from the growth phase (G1 phase) to the DNA replication phase (S phase), entering the cell proliferation phase. CDK4/6 inhibitors block cell proliferation at the G1 phase, thus achieving the purpose of inhibiting tumor proliferation. Currently, there are three CDK4/6 inhibitors on the global market: Palbociclib developed by Pfizer, Ribociclib by Novartis, and Abemaciclib by Eli Lilly, used for the treatment of hormone-receptor (HR) positive (ER+ and/or PR+), HER2-negative advanced or metastatic breast cancer patients.

The PALOMA-2 study, a phase III double-blind, randomized controlled trial, investigated the first-line treatment of advanced ER+/HER2-negative breast cancer with palbociclib in combination with letrozole. A total of 666 patients were enrolled. The study's primary endpoint showed that the combination of palbociclib and letrozole significantly prolonged the median progression-free survival (PFS) (24.8 months vs. 14.5 months) compared to letrozole alone, achieving a breakthrough of over two years in PFS for first-line treatment of advanced breast cancer. The most common adverse events of grade 3 or 4 were neutropenia, leukopenia, anemia, and fatigue.Similarly, the efficacy and safety of ribociclib combined with letrozole versus placebo combined with letrozole were assessed in a phase III randomized controlled trial for first-line treatment of HR-positive, HER2-negative recurrent or metastatic

postmenopausal breast cancer. The trial included 668 patients and demonstrated that ribociclib combined with letrozole significantly prolonged PFS (HR, 0.56; 95% CI, 0.43-0.72) and improved progression-free survival rates (63.0% vs. 42.2%) and overall response rates (52.7% vs. 37.1%, P <0.001). The most common adverse events of grade 3 or 4 reported in more than 10% of patients in both groups were neutropenia and leukopenia; the rates of discontinuation due to adverse events were 7.5% and 2.1%, respectively. The MONARCH-3 study, a double-blind, randomized phase III trial, investigated abemaciclib or placebo in combination with a non-steroidal aromatase inhibitor for first-line treatment of HR-positive, HER2-negative postmenopausal advanced breast cancer. A total of 493 patients were enrolled, with the abemaciclib group showing a significantly prolonged median PFS (not reached for the abemaciclib group vs. 14.7 months for the placebo group). The objective response rate was significantly higher in the abemaciclib group compared to the placebo group (59% vs. 44%). The most common adverse reaction in the abemaciclib group was diarrhea, and the most common grade 3 or 4 adverse events were neutropenia and leukopenia. Overall, the currently marketed CDK4/6 inhibitors (palbociclib, ribociclib, and abemaciclib) combined with non-steroidal aromatase inhibitors significantly improve progression-free survival and objective response rates, with a tolerable safety profile in patients with HR-positive, HER2-negative advanced breast cancer.

Dalpiciclib (SHR6390) is an orally administered, highly efficient, and selective small molecule CDK4/6 inhibitor developed by Jiangsu Hengrui Medicine Co., Ltd.[redacted]

Research by Shom Goel and colleagues indicates that CDK4/6 inhibitors can activate anti-tumor immune function by increasing the expression of tumor cell antigens and inhibiting the proliferation of immunosuppressive regulatory T cells. Another study suggests that CDK4/6 inhibitors not only block Rb phosphorylation but also reduce TSC2 phosphorylation, thereby partially weakening mTORC1 activity. This reduces inhibition of upstream EGFR family kinases, making tumors more sensitive to EGFR/HER2 inhibitors. Therefore, dual inhibition of EGFR/HER2 and CDK4/6 leads to more effective inhibition of TSC2 phosphorylation, thereby suppressing mTORC1/S6K/S6RP pathway activity. In various PDX models, CDK4/6 inhibitors sensitize HER2-targeted therapy and significantly delay tumor recurrence in HER2-positive breast cancer models.

Currently, researchers at Peking University Cancer Hospital have initiated a Phase I trial (NCT03480256) evaluating the combination of SHR6390 with pyrotinib for the treatment of HER2-positive advanced gastric cancer. Additionally, several studies are underway nationally and internationally focusing on CDK4/6 inhibitors in combination with anti-HER2 small

molecule inhibitors or antibodies for hormone receptor-positive, HER2-positive advanced breast cancer. A single-arm open-label Phase Ib/II clinical trial (NCT03054363) evaluating the safety and efficacy of tucatinib in combination with palbociclib and letrozole for the treatment of hormone receptor-positive and HER2-positive metastatic breast cancer. The NA-PHER2 study (NCT02530424), a Phase II trial investigating the combination of palbociclib with trastuzumab, pertuzumab, and fluorouracil/doxorubicin/cyclophosphamide as neoadjuvant therapy for ER-positive HER2-positive invasive breast cancer. The PATRICIA study (NCT02448420), a Phase II trial evaluating palbociclib in combination with trastuzumab, with or without letrozole, for the treatment of postmenopausal locally advanced or metastatic ER-positive HER2-positive breast cancer. The MonarcHER study (NCT02675231), a Phase II trial comparing abemaciclib in combination with trastuzumab and fluorouracil/doxorubicin/cyclophosphamide, and abemaciclib in combination with trastuzumab, versus trastuzumab in combination with standard chemotherapy for the treatment of ER-positive HER2-positive advanced breast cancer.

Based on the aforementioned clinical trials and theoretical foundations, CDK4/6 inhibitors can enhance the efficacy of endocrine therapy and augment the anti-tumor effects of HER2 targeting. Fudan University Affiliated Cancer Hospital has conducted a Phase Ib clinical study on the combination of letrozole, pyrotinib, and the CDK4/6 inhibitor SHR6390 for the treatment of hormone receptor-positive, HER2-positive advanced breast cancer. The tolerance data and preliminary efficacy data from the Phase Ib study are as follows:

1) Combination of letrozole 2.5 mg/day, pyrotinib 400 mg/day, and SHR6390 125 mg/day: 5 patients enrolled, 2 experienced dose-limiting toxicity (grade III oral mucositis), with a best response rate (ORR) of 60%.

2) Combination of letrozole 2.5 mg/day, pyrotinib 400 mg/day, and SHR6390 100 mg/day: 6 patients enrolled, 1 experienced dose-limiting toxicity (grade III oral mucositis), with a best response rate (ORR) of 50%.

3) Combination of letrozole 2.5 mg/day, pyrotinib 320 mg/day, and SHR6390 125 mg/day: 4 patients enrolled, no dose-limiting toxicity reported, with 4 patients achieving partial response and a best response rate (ORR) of 100%.

Based on the tolerability and safety data from the Phase Ib study, along with the preliminary efficacy data, the recommended dosages for the Phase II study are: pyrotinib 320 mg/day and SHR6390 125 mg/day. Subsequently, a multicenter Phase II clinical study is planned to be conducted on the combination of pyrotinib, CDK4/6 inhibitor dalcetrapib (SHR6390), and endocrine therapy for the treatment of estrogen receptor-positive, HER2-positive advanced

breast cancer.

1.1. Investigational Product

a) Name：Letrozole tablets

Letrozole tablets, a medication marketed by Jiangsu Hengrui Medicine Co., Ltd. For detailed information on its physicochemical properties and biological actions, it is advisable to refer directly to the drug's package insert.

b) Name：Fulvestrant Injection

Fulvestrant Injection, a medication marketed by Zhengda Tianqing Pharmaceutical Group. For detailed information on its physicochemical properties and biological actions, it is advisable to refer directly to the drug's package insert.

c) Name：Pyrotinib Malate

Hanyu Pinyin：Biluotini

English name：Pyrotinib

Chinese chemical name [redacted]

English chemical name [redacted]

d) Name：Dalpiciclib

Compound code: SHR6390

Hanyu Pinyin：Daerxili

English name：Dalpiciclib

Chinese chemical name [redacted]

Chemical structure[redacted]

1.2. The pharmacological type and mechanism for SHR6390

SHR6390 is a CDK4/6 kinase inhibitor and a [redacted] new drug developed by Jiangsu Hengrui Pharmaceuticals. Preclinical data indicate that SHR6390 selectively inhibits the activity of CDK4/6 kinases. This prevents the complex formed by these kinases with Cyclin D from phosphorylating the downstream Rb protein, thereby blocking the transition of cells from the G1 to the S phase and exerting an anti-proliferative and anti-tumor effect.

1.3. SHR6390 Pharmacodynamics

[redacted]

1.4. SHR6390 Toxicology

[redacted]

1.5. SHR6390 Pharmacokinetics

[redacted]

1.6. In Vivo Antitumor Activity of Pyrotinib and SHR6390 in ER+, HER2+ Breast Cancer Models

[redacted]

2. Clinical Studies

2.1. Pyrotinib Malate

Pyrotinib malate tablets, independently developed by Jiangsu Hengrui Pharmaceuticals, are an innovative drug and an irreversible dual-target tyrosine kinase inhibitor targeting EGFR and HER2. They are used for HER2-positive advanced or metastatic breast cancer. Phase II clinical trial data indicate that, for patients who failed treatment with anthracyclines and taxanes and received no more than two lines of chemotherapy after recurrence/metastasis, the combination of pyrotinib malate tablets with capecitabine (referred to as the "pyrotinib group") achieved an objective response rate of 78.5%, compared to 57.1% in the group combining lapatinib tablets with capecitabine (referred to as the "lapatinib group"). The median progression-free survival (PFS) was 18.1 months for the pyrotinib group and 7.0 months for the lapatinib group. The median PFS of the pyrotinib group was significantly longer than that of the lapatinib group, with statistical significance (P<0.0001). The risk of disease progression or death was reduced by 63.7% (HR=0.363) in the pyrotinib group compared to the lapatinib group, while maintaining good tolerability. Based on the efficacy and safety data obtained from the current Phase II clinical trials, the China National Medical Products Administration agreed to accept Hengrui Medicine's application for conditional approval of pyrotinib malate tablets. Following the publication of the large-scale Phase III studies PHENIX and PHOEBE, pyrotinib received full approval from the National Medical Products Administration in 2020 as a fully approved innovative anti-cancer drug.

2.2. SHR6390

SHR6390 has currently initiated Phase I clinical trials for advanced solid tumors, Phase I for advanced melanoma, and Phase Ib/II and Phase III clinical trials for advanced breast cancer.

SHR6390-I-101 is an ongoing Phase I clinical study in China that evaluates the tolerability and pharmacokinetics of SHR6390 in patients with advanced solid tumors. The primary objective of the study is to observe the dose-limiting toxicity (DLT) and maximum tolerated dose (MTD) of SHR6390 tablets after single and multiple oral administrations in patients with advanced solid tumors. The secondary objectives include observing the pharmacokinetic characteristics of SHR6390 tablets in these patients, as well as assessing their safety, tolerability, and the preliminary efficacy of SHR6390 tablets in the treatment of advanced solid tumors.[redacted]

The SHR6390-Ib/II-201 study is an ongoing Phase Ib/II clinical trial in China, investigating the combination of SHR6390 with letrozole, anastrozole, or fulvestrant in patients with hormone receptor-positive, HER2-negative advanced breast cancer.[redacted]

The SHR6390-III-301 study is an ongoing Phase III clinical trial in China, comparing SHR6390 combined with fulvestrant to placebo combined with fulvestrant in patients with hormone receptor-positive (HR+), HER2-negative locally advanced or metastatic breast cancer who have progressed after prior endocrine therapy. [redacted]On March 24, 2021, the SHR6390 tablets were included in the breakthrough therapy list by the Center for Drug Evaluation of the National Medical Products Administration. The proposed indication is for the use of SHR6390 tablets in combination with fulvestrant for the treatment of hormone receptor-positive, HER2-negative recurrent or metastatic breast cancer that has progressed following endocrine therapy.

The SHR6390-III-302 study is an ongoing Phase III clinical trial in China. It involves comparing the effectiveness of SHR6390 combined with either letrozole or anastrozole versus placebo combined with either letrozole or anastrozole. This trial is targeting patients with hormone receptor-positive (HR+), HER2-negative advanced breast cancer. The goal is to evaluate the efficacy and safety of SHR6390 in enhancing the benefits of aromatase inhibitors (letrozole or anastrozole) in this patient population.[redacted]

The SHR6390-III-303 study is an ongoing Phase III clinical trial being conducted in patients with early-stage hormone receptor-positive (HR+), HER2-negative breast cancer. This trial evaluates the efficacy and safety of SHR6390 combined with endocrine therapy compared to endocrine therapy alone in the adjuvant setting. The study aims to determine whether adding SHR6390 to standard endocrine treatment improves outcomes for patients with this type of breast cancer.

2.3. Marketed CDK4/6 inhibitors

The PALOMA-2 study, a Phase III double-blind, randomized controlled trial, assessed the efficacy of palbociclib combined with letrozole as a first-line treatment for advanced ER+/HER2- negative breast cancer. The study enrolled 666 patients, and the primary endpoint indicated that the median progression-free survival (PFS) reached 24.8 months with the combination, compared to 14.5 months for the letrozole alone group, marking a significant advancement in achieving over two years of PFS for first-line treatment in advanced breast cancer. The most common grade 3 or 4 adverse events included neutropenia (occurrence rate of 66.4% in the Palbociclib-letrozole group vs 1.4% in the placebo-letrozole group), leukopenia (24.8% vs. 0%), anemia (5.4% vs. 1.8%), and fatigue (1.8% vs. 0.5%).

Another Phase III randomized controlled trial investigated the efficacy and safety of ribociclib combined with letrozole versus placebo combined with letrozole in first-line treatment for HR-positive, HER2-negative postmenopausal patients with recurrent or metastatic breast cancer, including 668 patients. The ribociclib combination demonstrated significantly longer PFS compared to the placebo group (HR, 0.56; 95% CI, 0.43-0.72). After 18 months of follow-up, the ribociclib group had a PFS rate of 63.0% (95% CI, 54.6-70.3) versus 42.2% (95% CI, 34.8-49.5) in the placebo group. The overall response rates were 52.7% and 37.1%, respectively (P <0.001). Common grade 3 or 4 adverse events reported by more than 10% of patients included neutropenia (59.3% in the ribociclib group vs 0.9% in the placebo group) and leukopenia (21.0% & 0.6%); the rates of discontinuation due to adverse events were 7.5% and 2.1%, respectively.

The MONARCH-3 study, a double-blind, randomized Phase III trial, involved 493 patients with advanced breast cancer, assessing abemaciclib or placebo combined with a non-steroidal aromatase inhibitor for first-line treatment in HR-positive, HER2-negative postmenopausal advanced breast cancer. The median PFS was significantly extended in the abemaciclib group (HR=0.54; 95% CI, 0.41-0.72; P = .000021; median not reached for the abemaciclib group vs 14.7 months for the placebo group). The objective response rate was 59% in the abemaciclib group vs 44% in the placebo group (P = 0.004). In the abemaciclib group, the most common adverse reaction was diarrhea (81.3%, with grade 1 accounting for 44.6%). The most common grade 3 or 4 adverse events in the abemaciclib compared to the placebo group were neutropenia (21.1% vs 1.2%), diarrhea (9.5% vs 1.2%), and leukopenia (7.6% vs 0.6%).

3. Study Objectives and Study Endpoints

3.1. Primary Study Objective

Phase I (Ib stage):

Determine the safety and tolerability of the combination of a non-steroidal aromatase inhibitor (letrozole), pyrotinib maleate tablets, and the CDK4/6 inhibitor SHR6390 in the treatment of hormone receptor-positive, HER2-positive advanced breast cancer. Based on preliminary efficacy data, establish the recommended Phase II dosage for this combination therapy.

Phase II:

The primary research objective is to evaluate the effectiveness and safety of the combination of pyrotinib, dalpiciclib (SHR6390), and endocrine therapy in treating estrogen receptor-positive and HER2-positive advanced breast cancer. The study also aims to further explore the value of circulating tumor DNA (ctDNA) as a biomarker, as well as the clinical utility of FDG-PET and HER2-PET in assessing anti-tumor efficacy. [redacted]

3.2. Primary Endpoint

The primary endpoints of this trial include:

Phase I:

- Determination of dose-limiting toxicity (DLT) and maximum tolerated dose (MTD) of SHR6390 within the combination regimen to establish the recommended dosing regimen for the Phase II clinical study.

- Assessment of the incidence and severity of adverse events (AEs) and serious adverse events (SAEs) across different dose groups.

Phase II:

The objective response rate (ORR) of the recommended dosing regimen for the Phase II clinical study, evaluated based on RECIST 1.1 criteria.

3.3. Secondary Endpoints

Phase I

- Objective Response Rate (ORR), based on RECIST 1.1 criteria.

Phase II

- Incidence and severity of adverse events (AEs) and serious adverse events (SAEs).

Common Secondary Endpoints for Phase I/II Trial Include:

•Progression-free survival (PFS)

•Disease Control Rate (DCR, The proportion of subjects with a best overall response of complete response, partial response, or stable disease).

•Clinical benefit rate (CBR, proportion of complete response, partial response, or stable disease for at least 24 weeks)

•Duration of response (DOR)

•Exploratory analysis of the relationship between molecular markers and efficacy

4. Investigational Drugs

4.1. Name and Source

The investigational drugs for this project, letrozole, pyrotinib, and dalpiciclib (SHR6390 tablets), are all produced and supplied by Jiangsu Hengrui Pharmaceuticals Co., Ltd.

Fulvestrant is provided by Zhengda Tianqing Pharmaceutical Group Co., Ltd.

4.2. Dosage Form and Specifications

Letrozole tablets: 2.5 mg;

Pyrotinib tablets: 160 mg, 80 mg;

SHR6390 tablets: 25 mg, 125 mg;

Fulvestrant injection: 250 mg.

4.3. Storage

Storage Conditions: Store sealed at below 25°C.

Shelf Life: Tentatively 24 months.

4.4. Administration

- Letrozole: Administer orally, 2.5 mg daily, on an empty stomach. Administer continuously for 28 days as one cycle.
- Fulvestrant: Administer by intramuscular injection on Day 1 (with an additional dose on Day 15 of the first cycle); each cycle lasts 28 days.
- Pyrotinib: Administer orally, 320 mg daily, within 30 minutes after breakfast. Administer continuously for 28 days as one cycle.
- SHR6390: Administer orally, 125 mg daily, on an empty stomach (ensure fasting for at least 1 hour before and 1 hour after taking the medication). The medication is taken for 28 days as one cycle, with continuous dosing for the first three weeks (Day 1 to Day 21) and no dosing during the fourth week (Day 22 to Day 28).

4.5. Management, Distribution, and Retrieval

The management, distribution, and retrieval of drugs for clinical use in this trial are handled by designated personnel. Researchers must ensure that all investigational drugs are used only for participants in this clinical trial, and the dosage and administration should comply with the trial protocol. Any remaining drugs must be returned and must not be transferred to anyone not participating in the clinical trial. During drug distribution, a drug receipt form must be signed by two people, in duplicate. At the end of the study, any remaining drugs and empty boxes are to be collected, and a drug return form must be signed. Each instance of drug distribution and retrieval must be promptly recorded on a specific record form.

5. Overall Study Design

This study is a single-arm, open-label, dose-escalation Phase Ib clinical trial and a single-arm, open-label, multi-center Phase II clinical trial. In accordance with the "Regulations for Drug Registration," "Good Clinical Practice," and "Guidelines for Clinical Pharmacokinetics Studies of New Drugs (Chemical Drugs)," this trial is conducted among patients with estrogen receptor-positive, HER2-positive advanced breast cancer to evaluate the clinical efficacy and safety of the combination therapy consisting of pyrotinib, dalpiciclib (SHR6390), and endocrine therapy.

6. Study Design

6.1. Dosage and Administration

In patients with hormone receptor-positive, HER2-positive advanced breast cancer who have either not responded to standard treatments or lack standard treatment options, a dose-escalation study of the combination therapy will be conducted to observe tolerability and preliminarily assess efficacy. Once the last subject in each dose group completes 28 consecutive days of dosing and the DLT observation results for that dose group meet the criteria for dose escalation, the next higher dose group may begin the dose-escalation trial.

Phase I (Ib stage):

In this study, letrozole is administered at 2.5 mg/day, pyrotinib at either 400 mg/day or 320 mg/day, and SHR6390 is organized into three dose groups.

Dose groups	SHR6390	pyrotinib	Patients
A	125 mg/d	400 mg/d	3~6
B	150 mg/d	400 mg/d	3~6
C(backup group)	100 mg/d	400 mg/d	3~6
D(backup group)	125 mg/d	320 mg/d	3~6
E(backup group)	150 mg/d	320 mg/d	3~6
F(backup group)	100 mg/d	320 mg/d	3~6

The trial begins with Group A and follows a 3+3 dose-escalation design. The escalation sequence proceeds from Group A to Group B. If ≥2 cases of DLT are observed in Group A, Groups C and D will be initiated. If Group C remains intolerable, backup Group F will be initiated. If Group D is tolerable, escalation will proceed to Group E; if Group D is intolerable, then Group F will be initiated. If Group F remains intolerable, the research team will analyze the trial data to determine whether to terminate the study. DLT assessment will be conducted at the end of one cycle (28 days) for each dose group.

For specific dosing instructions of the three drugs, refer to Section 4.4. The initial two cycles are designated as the core study phase. Subjects evaluated as CR, PR, or SD at the end of Cycle 2 may continue on the assigned combination regimen until disease progression, intolerable toxicity, or voluntary withdrawal.

Phase II:

Based on the recommended phase 2 dose established in phase I, proceed with the phase 2 dose expansion study using pyrotinib 320mg and SHR6390 125mg in combination with endocrine therapy. Continue administration until the occurrence of disease progression, intolerable toxicity, or voluntary withdrawal of informed consent by the participant.

Study Design Schema：

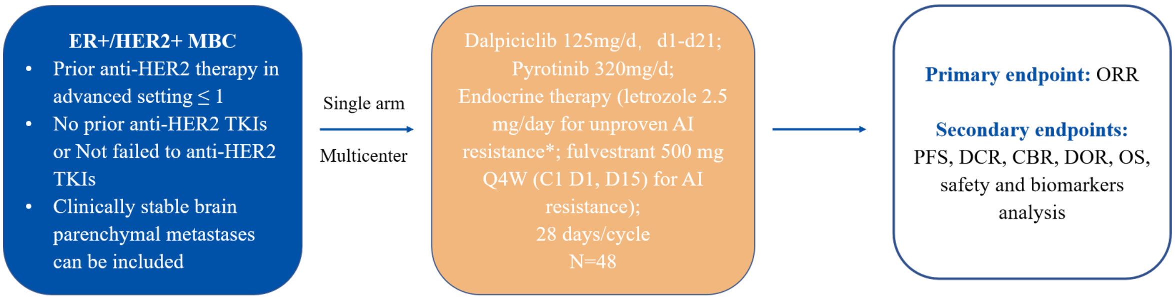

*Resistance includes both primary and secondary resistance.

6.2. Dose-limiting toxicity (DLT)

In this trial, DLT is defined as any of the following drug-related or possibly drug-related adverse events occurring within Cycle 1 (based on CTC-AE v4.0.3 criteria):

1. Hematologic Toxicity:

- Grade 4 neutropenia lasting ≥5 days;

- Grade 4 thrombocytopenia or Grade 3 thrombocytopenia with clinically significant bleeding;

- Grade ≥3 neutropenia with fever (≥38.0°C lasting 1 hour or >38.3°C);

- Grade ≥4 anemia.

2. Non-Hematologic Toxicity:

Any Grade ≥3 non-hematologic toxicity, except for the following:

- Grade 3–4 nausea/vomiting and/or diarrhea and/or electrolyte imbalance that resolves to Grade ≤2 within 72 hours with optimal supportive treatment;

- Grade 3–4 elevations in alkaline phosphatase or gamma-glutamyl transferase that are clearly related to the tumor and unrelated to the drug.

6.3. Maximum tolerated dose (MTD)

During the dosing observation period in Cycle 1 (28 days), if ≥1/3 of the subjects in a given dose group experience DLT, the previous dose group is designated as the MTD. If dose escalation reaches Group B in the Phase I trial without ≥1/3 of subjects experiencing DLT, the research team will discuss whether to initiate the backup dose group C and determine the recommended dose for the Phase II trial.

6.4. Subject replacement

If a subject needs to withdraw from the study due to a non-DLT event during the DLT observation period, an additional subject will be included as a replacement.

If a subject experiences an unplanned treatment interruption exceeding 4 days during the DLT observation period, they will be considered to have received insufficient treatment intensity. In this case, even if no DLT is observed, an additional subject will be included as a replacement.

6.5. Planned Sample Size

Based on the analysis of Phase Ib study data, the expected efficacy in the Phase II study is projected to be 60%, with a half-width of 0.15. Taking into account a dropout rate of 15%, the calculated sample size is 48 participants. Four participants from the Phase Ib RP2D (Recommended Phase 2 Dose) cohort can be included in the Phase II study analysis. The plan for Phase II is to enroll the remaining 44 participants.

7. Sample Collection

In Phase Ib, plasma samples will be collected at 1 hour, 3 hours, and 24 hours after dosing on Day 21 of the first cycle. In Phase II, blood plasma samples are collected from 8-10 participants on day 21 of the first cycle just within 0.5 hours before and at 2 hours ±5 minutes, 4 hours ±10 minutes, 6 hours ±10 minutes, 12 hours ±10 minutes, and 24 hours ±0.5 hours after administration of pyrotinib. On day 22 of the 3rd, 5th, 8th, and 12th cycles,

samples are taken within 0.5 hours before pyrotinib administration. The timing of dosing for the 3 days prior to PK blood collection is relatively fixed. Blood is drawn into two lithium heparin anticoagulant tubes, each extracting 3 mL of venous blood. The tubes are gently inverted 3-5 times to ensure adequate mixing of blood and anticoagulant and are then left at room temperature. The actual blood collection times are accurately recorded and detailed in the blood collection log. Within one hour of collection, the samples are transferred to a centrifuge and centrifuged at 2000g for 10 minutes at room temperature (15° C - 32° C) to separate the plasma. The plasma is then aliquoted into two cryogenic tubes: one for testing and one as a backup. The testing tube should contain no less than 0.5 ml of plasma, with the remaining plasma transferred to the backup tube. Care should be taken not to aspirate the bottom layer of blood cells, and appropriate labels should be affixed. The tubes are stored at -80±10° C in a low-temperature freezer for testing.The cryogenic tubes must be kept in the low-temperature freezer until shipment, and the freezer temperature should be recorded daily. When shipping samples, the testing tubes are sent first while the backup tubes remain stored at -80±10° C until the end of the experiment when they are shipped collectively.

Baseline blood samples are also collected at the end of cycle 2 and before disease progression or the start of new anti-tumor treatment. This involves [redacted] along with ctDNA blood samples. Existing paraffin-embedded tumor tissue samples or ⩾10 unstained slides are collected, with an effort made to obtain biopsy samples from metastatic sites.

8. Participants

8.1. Subjects and Sample Size

From an ethical and scientific standpoint, subjects must have received no more than one prior systemic treatment regimen containing trastuzumab for recurrent or metastatic breast cancer. Subjects should have no history of HER2 TKI treatment or, if previously treated, should not show evidence of treatment failure. Prior endocrine therapy must not indicate aromatase inhibitor resistance. Informed consent must be signed prior to the clinical trial.

In Phase I, 15 subjects were enrolled. Based on the Phase I results, Phase II is expected to enroll 48 subjects in total. Four subjects from the RP2D dose group in the Ib phase may be included in the Phase II analysis, with the remaining 44 subjects planned for additional enrollment in Phase II.

8.2. Inclusion Criteria

1. Participants voluntarily join the study, sign an informed consent form, and demonstrate good compliance.

2. Female patients aged ≥18 and ≤75, either postmenopausal or pre/perimenopausal, meeting one of the following:

a) Previous bilateral oophorectomy, or aged ≥60; or

b) Aged <60, naturally postmenopausal (defined as at least 12 consecutive months of amenorrhea without other pathological or physiological causes), with postmenopausal levels of E2 and FSH; or

c) Pre- or perimenopausal women also eligible but must be willing to undergo LHRH agonist treatment during the study.

3. Patients with histologically confirmed recurrent/metastatic breast cancer, ER-positive and HER2-positive:

- HER2 positivity defined as 3+ by standard immunohistochemistry (IHC) and/or positive by in situ hybridization (ISH).

- ER positivity defined as ≥1% ER-expressing cells.

- Local recurrence must be confirmed by the researcher as inoperable.

4. At least one measurable extracranial lesion according to RECIST 1.1 criteria.

5. Previous antitumor treatment guidelines:

- Maximum of one prior systemic therapy regimen for recurrent/metastatic breast cancer including anti-HER2 ADC:

i. If not previously treated with a trastuzumab regimen in the advanced stage, or if recurrence occurs more than one year after adjuvant trastuzumab treatment, subsequent therapy is considered first-line anti-HER2 treatment.

ii. If first-line treatment with a trastuzumab regimen fails, or if recurrence occurs during adjuvant trastuzumab treatment or within one year after its completion, subsequent therapy is considered second-line anti-HER2 treatment.

- No prior anti-HER2 TKI therapy or no proof of failure with such therapy.

- Previous endocrine therapy not proven resistant to aromatase inhibitors (Resistance defined as recurrence within one year after completing adjuvant aromatase inhibitor therapy or disease progression during the recurrent/metastatic phase after aromatase inhibitor treatment); subsequent endocrine therapy with letrozole. If aromatase inhibitor resistance is present, subsequent endocrine therapy with fulvestrant.

6. ECOG performance status of 0 – 1.

7. Expected survival of ≥12 weeks.

8. Adequate organ function (no use of any blood components and growth factors within 2 weeks prior to enrollment):

- Absolute neutrophil count ≥1.5×10^9/L;
- Platelets ≥90×10^9/L;
- Hemoglobin ≥90g/L;
- Total bilirubin ≤1.5 times upper limit of normal (ULN);
- ALT and AST ≤2.5 times ULN;
- Urea/Blood Urea Nitrogen (BUN) and creatinine (Cr) ≤1.5×ULN;
- Left ventricular ejection fraction (LVEF) ≥50%;
- Fridericia-corrected QT interval (QTcF) < 470 ms;
- INR ≤1.5×ULN, APTT ≤1.5×ULN.

8.3. Exclusion Criteria

Patients with any of the following criteria are not eligible to participate in this study:

1. Brain meningeal metastasis or active brain parenchymal metastasis. Clinically stable

brain parenchymal metastasis patients may be included, including asymptomatic brain metastasis without prior local treatment; or patients who have received CNS metastasis treatment (radiation or surgery) confirmed stable by imaging for at least 4 weeks, and who have ceased symptomatic treatment (including steroids and mannitol) for more than 2 weeks.

2. Prior treatment with any CDK4/6 inhibitor.

3. Presence of symptomatic ascites, pleural effusion, or pericardial effusion requiring drainage at baseline, or who have undergone serous cavity fluid drainage within 4 weeks prior to first drug administration.

4. Inability to swallow, intestinal obstruction, or other factors affecting drug intake and absorption.

5. Received chemotherapy, molecular targeted therapy, or other systemic treatments including clinical trial drugs within 4 weeks prior to enrollment; received endocrine therapy within 2 weeks prior to enrollment.

6. History of other malignancies within the past 5 years or concurrent malignancy, except for cured basal cell carcinoma of the skin and cervical carcinoma in situ.

7. Major surgical procedures or significant trauma within 4 weeks prior to the first drug administration, or anticipated need for major surgery during the study.

8. Pregnant or breastfeeding women, women of childbearing potential with a positive baseline pregnancy test, or unwilling to use effective contraception.

9. Known hypersensitivity to any component of the study drugs.

10. Active HBV or HCV infection; stable hepatitis B (HBV viral copy number not exceeding the upper limit of normal) treated with medication and cured hepatitis C (HCV viral copy number below the detection limit of the assay) are excluded.

11. History of immunodeficiency, including HIV positivity, or any other acquired or congenital immunodeficiency diseases, or history of organ transplantation.

12. History of any cardiac disease, including: (1) angina; (2) arrhythmias requiring medication or clinically significant; (3) myocardial infarction; (4) heart failure; (5) any other heart disease deemed by the investigator as unsuitable for participation in the trial; severe cardiac or renal function abnormalities of grade ≥II found during the screening period.

13. According to the investigator's judgement, severe concomitant diseases that pose a risk to patient safety or affect the completion of the study (e.g., severe hypertension, diabetes,

thyroid disorders).

14. History of definite neurologic or psychiatric disorders, including epilepsy or dementia.

15. Severe infection within 4 weeks prior to the first drug administration (e.g., requiring intravenous administration of antibiotics, antifungals, or antivirals according to clinical guidelines), or unexplained fever >38.3°C during the screening period or prior to the first administration.

8.4. Participant Identification

In this trial, all participants who have signed the informed consent form will receive a unique participant code, such as II-01-01. The coding rule is as follows: the first two digits represent the trial phase, such as II; the middle two digits are the center number, sequentially assigned as 01, 02, 03, etc.; the last two digits correspond to the order in which participants are screened at that center, continuing as 01, 02, 03, and so forth.

8.5. Dropout Criteria

All patients who have signed the written informed consent form and passed the screening to enter the trial have the right to withdraw from the clinical trial at any time. Regardless of the reason or timing of withdrawal, participants who have not completed the first cycle of multiple dosing and cannot undergo a safety evaluation will be considered as dropout cases.

8.6. Termination Criteria for Participant Treatment

If any of the following conditions occur, the participant must withdraw/terminate treatment:

1. The participant withdraws informed consent and requests to exit.
2. Imaging examination shows disease progression.
3. Inability to tolerate toxicity.
4. Serious protocol violation, as assessed by the investigator warranting treatment termination.
5. Participant loss to follow-up or occurrence of a pregnancy event.
6. Other circumstances deemed necessary for participant withdrawal from the study by the investigator.

8.7. Study Termination Criteria

Study termination criteria include, but are not limited to:

1. Discovery of unexpected, significant, or unacceptable risks to participants;

2. The investigational drug/therapy is ineffective, or continuation of the trial is deemed futile;

3. The investigator decides to terminate the study due to reasons such as severe lag in participant enrollment or significant protocol violation

9. Dose Adjustment and concomitant medication

9.1. DLT Observation Period

During the DLT observation period in Phase Ib, non-DLT adverse events generally should not receive medical intervention to allow for observation of potential adverse reactions from the investigational drug, including their severity and reversibility. However, if a DLT as defined in the protocol occurs, the investigational drug must be immediately discontinued, and appropriate medical treatment should be initiated. The medication used for management should be documented in the CRF.

9.2. Continuous Dosing in Cycle 2 and Subsequent Cycles

Dose Adjustment Criteria (to be applied for all dosing cycles in the Phase II study)

After the occurrence of toxic reactions, physicians can make judgments based on the situation and provide appropriate management. The specific principles of management are as follows:

Table 6

**Adverse Events**	**Severity**			
	**Grade I**	**Grade II**	**Grade III**	**Grade IV**
**Hematologic Toxicity**	Maintain dose	Maintain dose	Pause treatment, symptomatic management, resume treatment if reduced to Grade I or below, adjust dose as per investigator's judgment for this and subsequent cycles	Pause treatment, symptomatic management, dose reduction required if reduced to Grade I or below

**Non-Hematologic Toxicity**	Maintain dose	Maintain or pause treatment, symptomatic management, if reduced to Grade I or below, dose adjustment optional based on investigator’s	Pause treatment, symptomatic management, dose reduction required if reduced to Grade I or below	Permanently discontinue treatment, withdraw from study
**Febrile Neutropenia**	-	-	Pause treatment, symptomatic management, resume treatment if reduced to Grade I or below, dose	Permanently discontinue treatment, withdraw from study

When definite toxicity related to the study drug occurs, the investigator will handle it based on clinical manifestations. Administration may resume once recovery to ≤ Grade I is achieved (or if the investigator deems the adverse event tolerable at ≤ Grade II without significant safety risks). If the same adverse event recurs, the investigator will decide whether to pause and adjust the dosage, or require the participant to withdraw from the study; the investigator's priority will be to maximize participant safety.

If toxicity does not resolve within 2 weeks after pausing medication, the participant should, in principle, withdraw from the study. Any pauses in administration should be included in the dosing cycle.

9.3. Prohibited Medications

During the treatment period, the use of other antitumor drugs and tumor treatment-related adjuvant medications should be discontinued. This includes antitumor traditional Chinese medicines, antitumor hormonal therapy drugs, immunotherapies, or other antitumor therapeutic drugs.

9.4. Permitted Medications

If participants experience adverse reactions, they should be closely monitored, and symptomatic treatment should be administered as necessary. The treatment medications used

must be recorded and explained on the Case Report Form (CRF). Details such as the timing of use, name of the medication, and the method and dosage should be documented.

10. Study Procedures

10.1. Screening period

Before starting treatment with the study drug, the following screening steps should be completed within 28 days:

- Signing of Informed Consent Form: Patients must read and sign the informed consent form approved by the Ethics Committee.

- Medical History and Demographic Data Collection: Includes collecting patient's ID, address, contact details, a detailed inquiry of tumor history/other diseases history, pathology results, ER/PR/HER2 testing reports, history of tumor surgery, chemotherapy, radiation therapy, and other disease treatments; non-breast cancer tumor history.

- Tests for Hepatitis B, Hepatitis C, and HIV: Hepatitis B panel and, if abnormal, HBV DNA replication testing; Hepatitis C virus antibody (anti-HCV), and HIV antibody tests.

- Imaging Studies: Screening imaging includes enhanced CT or MRI of the chest, abdomen, and brain, and other suspected areas like the neck or pelvis. Tumor baseline assessment can be extended to within four weeks before the first administration, CT/MRI scans obtained before signing informed consent can be used for tumor evaluation if they meet the requirements; bone scans are necessary if clinical suspicion of bone metastases exists (FDG-PET, HER2-PET are performed at the lead unit and participating units with capabilities).

- [redacted]

- Biomarker Collection: [redacted] along with ctDNA samples; collect existing paraffin-embedded tumor tissue samples or ≥10 unstained slides, preferably obtaining biopsy specimens from metastatic sites.

- Echocardiogram: Monitor changes in LVEF value, additional unplanned checks may be performed if LVEF falls below 50% and drops by ≥10% from baseline, or if symptoms like

chest pain or palpitations occur.

- Evaluation of Current Medications and Treatments: Assess the medications and treatments currently being used by the patient.

- Collection of Adverse Events: Start collecting adverse events from the time of informed consent.

The following screening steps should be completed within 7 days before starting treatment with the study drug:

- Vital Signs Check: Temperature, respiration, pulse, blood pressure; smoking and coffee consumption are prohibited 30 minutes before measurement, and at least 10 minutes of rest in a seated position with the elbow at heart level is required for blood pressure measurement.

- Physical Examination: Examination of major body systems (head, face, skin system, lymph nodes, eyes, ENT, oral cavity, respiratory system, cardiovascular system, abdomen, reproductive urinary system, musculoskeletal, nervous system, and mental state).

- ECOG Performance Status.

- Complete Blood Count: Hemoglobin, red blood cells, white blood cells, neutrophils, lymphocytes, and platelet count.

- Urinalysis: Urine protein, urine sugar, urine occult blood (urine red cells, white cells). If the semi-quantitative method shows protein 2+, a 24-hour urine protein quantitative test is performed.

- Stool Routine: Including fecal occult blood.

- Blood Biochemistry: Total bilirubin, conjugated bilirubin, ALT, AST, ALP, γ-GT, LDH, total protein, albumin, urea/urea nitrogen, creatinine, uric acid, fasting blood glucose, triglycerides, cholesterol, potassium, sodium, chloride, calcium, phosphorus, magnesium; cardiac enzyme spectrum may be added if necessary.

- Pregnancy Test: Serum pregnancy testing within one week before the first medication use in women of childbearing age.

- 12-Lead ECG: If clinically significant abnormalities are found on the ECG, the investigator

may confirm it again if necessary.

10.2. Trial Period

During the study, the following tasks should be completed within the specified timeframes indicated in the trial schedule (note: the time window from the first cycle is ±3 days):

- ECOG Score, Vital Signs, Physical Examination, 12-Lead ECG: Day 15 and 28 of Cycle 1, Day 28 of Cycle 2 (can be either C1D28 or C2D1, same for subsequent cycles), thereafter on Day 28 of each cycle.
- Complete Blood Count, Blood Biochemistry: Day 15 and 28 of Cycle 1, Day 28 of Cycle 2 (can be either C1D28 or C2D1, same for subsequent cycles), thereafter on Day 28 of each cycle (if the participant is from out of town, a weekly complete blood count can be performed at a local hospital from Cycle 2 onwards and the results communicated to the researcher, who will decide if further investigation at the study center is necessary).
- Urinalysis, Stool Routine: Every three cycles, replaced by screening tests in Cycle 1, Day 1 of Cycle 2 (can be either C1D28 or C2D1, same for subsequent cycles), thereafter on Day 28 of each cycle.
- Echocardiogram: Every three cycles, replaced by screening tests in Cycle 1, Day 1 of Cycle 4 (can be either C3D28 or C4D1, same for subsequent cycles), thereafter every three cycles on Day 28; at the end of treatment or upon participant withdrawal (if not done in the previous four weeks).
- Biomarker Collection: Baseline, end of Cycle 2, before disease progression or starting a new anticancer treatment, collecting [redacted] along with ctDNA samples.
- Imaging Studies: The allowed imaging study window is ±7 days, and during treatment, imaging should be conducted under the same conditions as baseline (slice thickness, contrast use, etc.). Specific assessment times include:
- Every six cycles before medication, initial assessment at the end of Cycle 2, then every two cycles for six cycles, followed by every three cycles until disease progression, intolerable toxicity, or the start of new cancer treatment.
- For initial assessments showing CR or PR, a confirmation is recommended 4-6 weeks later; subsequent tumor evaluations should not alter the fixed two-cycle examination timeline.
- Unscheduled imaging studies may be performed if disease progression is suspected (e.g., worsening symptoms).
- Aside from confirmed disease progression through imaging, participants ending trial

treatment for other reasons should undergo imaging evaluation at the end of treatment if not done in the prior four weeks, and follow-up according to the protocol-specified frequency continues after trial end until documented disease progression or the start of a new cancer treatment.

-[redacted]

- Adverse Events: From signing the informed consent form until just before starting the medication, only serious adverse events are recorded, up to 28 days after the last medication, and follow-up continues until adverse events disappear, alleviate to baseline level or ≤ Grade 1, or stabilize.
- Recording Adverse Reactions: From the first day of study treatment, at least 28 days after the last treatment, or until all serious or drug-related toxicities recover to ≤ Grade 1 per NCI-CTC AE 4.0.3 standards. Additionally, various clinical manifestations during medication are observed and recorded.
- Recording Concomitant Medication or Treatment: From 28 days before study treatment until 28 days after the last treatment.

Note: Researchers may increase the frequency of participant visits (e.g., weekly) and the content of examinations as needed to maximize participant safety.

10.3. Study termination/withdrawal

Study termination: 24 months after the last subject is enrolled, or when the researcher deems it necessary to end the trial prematurely.

10.4. Follow-up after treatment completion

- Disease Progression: In addition to imaging-confirmed disease progression, subjects who end trial treatment for other reasons must undergo imaging evaluation at the end of treatment if no imaging has been done within the four weeks prior to the trial's conclusion. Furthermore, after the trial ends, continue to follow the tumor response according to the schedule specified in the protocol until documented disease progression or the start of a new cancer treatment.

- Survival Follow-Up: After the end of the trial treatment, survival status and subsequent anti-cancer treatment information can be collected every three months through clinical visits or telephone follow-ups until death.

10.4.1. Criteria for Severity of Adverse Events

Refer to version 4.0.3 of the NCI-CTC AE for grading standards of drug adverse reactions. For adverse reactions not listed in the table, refer to the following criteria:

- Grade I: Mild, no clinical symptoms or only minor clinical symptoms; only clinical or laboratory findings abnormal; no treatment required.
- Grade II: Moderate, requires minimal, local, or non-invasive treatment; activities of daily living (ADL) using tools are restricted, which includes activities like cooking, shopping, making phone calls, handling money, etc., consistent with age.
- Grade III: Severe or medically significant symptoms but not immediately life-threatening; causes hospitalization or prolongation of hospital stay; results in disability; self-care ADL is restricted. Self-care ADL includes bathing, dressing, undressing, eating, using the toilet, taking medication, etc., not bedridden.
- Grade IV: Life-threatening, requires urgent treatment.
- Grade V: Death due to adverse event.

11. Safety Assessment

11.1. Adverse Event (AE)

11.1.1. Adverse Event Definition

An adverse event is defined as any unfavorable medical occurrence in a clinical trial participant after the signing of the informed consent form. In this trial, any adverse medical events occurring from the start of the subject's treatment with the study drug until 28 days after the last use of the study drug are considered adverse events, regardless of causality with the study drug.

Researchers should record in detail any adverse events experienced by the participants, including: description of the adverse event and all related symptoms, time of occurrence, severity, duration, actions taken, and final outcome and resolution.

Concerned Adverse Events

After the occurrence of toxicity reactions, in principle, medication should not be stopped or the dosage reduced. Treatment may be given by the physician based on the situation, with specific treatment principles as follows:

- Hematological Support: Symptomatic treatment can be provided by researchers based on clinical presentations when hematological toxicity occurs. When hematological toxicity reaches Grade ≥III, medication should be paused until recovery to ≤Grade I, then resumed at the original dosage level. For Grade III or IV anemia, dosing may not be paused, and transfusion treatment may be carried out based on the researcher's judgment. Pausing medication does not affect the timing of tumor evaluation. If medication is paused for more than 14 days, the patient must withdraw from the study.
- Diarrhea: According to the "Medication Pause and Dose Adjustment" provisions in the trial protocol, symptomatic treatment should be given first, followed by close follow-up or observation (≤14 days). Clinical advice is to start oral montmorillonite powder (3 g/sachet, three times/day) or loperamide on the day of diarrhea. For unresolved Grade III diarrhea, medication should be paused; once the adverse event has recovered to within Grade I, resume the original dosage or reduce the dosage.
- Liver Function Abnormalities: Symptomatic treatment or observation (≤14 days) should be given by researchers based on the participant and adverse event conditions. If abnormalities persist after treatment or observation, close follow-up is required, and dosage adjustments and/or increased frequency of biochemical tests may be necessary. For participants with liver metastases, if ALT/AST levels exceed 1.5×UNL at enrollment, close monitoring of liver function is required, and a comprehensive decision should be made on whether the participant is suitable for inclusion in the study.
- Other Risks: Since the study drug is experimental, using it alone or in combination with other drugs may carry unknown risks. All drugs have potential risks of allergic reactions, which could be life-threatening if not treated promptly. Any severe allergic reaction symptoms, such as difficulty breathing after activity or swelling of the face, lips, gums, tongue, or neck, should receive immediate medical help and contact the study physician. Other allergic reactions may include rash, hives, or blisters. It is very important for patients to promptly report all symptoms and side effects, regardless of whether they believe these are caused by the study drug.

Discomfort resulting from this experiment may not be limited to the events listed above, but we will closely follow up and actively manage to ensure the safety and well-being of the

patients to the greatest extent possible, and all medications used in the treatment should be recorded on the CRF form.

11.1.2. Adverse Event-Drug Relationship Classification

Adverse events include all unexpected clinical manifestations occurring after the signing of the informed consent form, regardless of their relation to the study drug or even if the drug was administered. All adverse events must be reported clinically. Any discomfort reported by patients or any abnormal changes in objective laboratory tests during the treatment period must be accurately recorded, including the severity, duration, management, and outcome of the adverse events. Clinicians should also comprehensively determine the relationship between the adverse event and the study drug. The relationship between adverse events and the study drug is judged on a five-level scale: definitely related, possibly related, possibly unrelated, definitely unrelated, and indeterminate. The first two categories are considered adverse reactions, and the incidence of adverse reactions is statistically analyzed.

- Definitely related: The reaction occurs in a reasonable temporal sequence after medication use and is consistent with the known reaction type of the suspected drug; improvement after stopping the drug, and the reaction reappears upon re-administration.
- Possibly related: The reaction occurs in a reasonable temporal sequence after medication use and is consistent with the known reaction type of the suspected drug; the patient’s clinical status or other treatment modalities might also produce such a reaction.
- Possibly unrelated: The reaction does not quite fit the reasonable temporal sequence after medication use, and the reaction type does not quite match the known reactions of the suspected drug; the patient’s clinical status or other treatment modalities could possibly cause such a reaction.
- Definitely unrelated: The reaction does not fit the reasonable temporal sequence after medication use, and the reaction matches a known reaction type of a non-trial drug; the patient’s clinical status or other treatment modalities might produce the reaction, which disappears with the improvement of the disease state or cessation of other treatments, and reappears with the re-use of other treatment methods.
- Indeterminate: The reaction occurs without a clear temporal relationship to medication use, is similar to the known reactions of the drug, and other concurrently used drugs might also cause the same reaction.

11.2.Serious Adverse Events (SAE)

11.2.1. Serious Adverse Events Definition

Serious Adverse Events (SAE) are medical events that occur during a clinical trial requiring hospitalization or prolongation of hospital stay, result in disability, impair work ability, are life-threatening or result in death, or lead to congenital anomalies. This includes the following unexpected medical events:

- Events leading to death;
- Life-threatening events (defined as situations where the subject is at risk of death at the time of the event);
- Events requiring hospitalization or prolonging hospital stay;
- Events that may cause permanent or serious disability/impairment;
- Congenital anomalies or birth defects;
- Other significant medical events.

11.2.2. Pregnancy

During the clinical trial, if a female participant becomes pregnant, she will be withdrawn from the trial， and the pregnancy should be reported as a serious adverse event.

11.2.3. Disease Progression

Disease progression (including symptoms and signs of progression) should not be reported as a serious adverse event (SAE). However, if a death occurs due to disease progression during the trial or within the safety reporting period, it should be reported as an SAE. Hospitalization due to symptoms and signs of disease progression should not be reported as an SAE. If the ultimate outcome of the cancer is death within the trial or safety reporting period, the event leading to death must be reported as an SAE.

11.2.4. Undergoing Other Antineoplastic Treatments

Adverse event recording begins upon signing the informed consent and continues until 28 days after the last administration of the study drug. If a participant starts other antineoplastic treatments after these 28 days, there is no need to continue tracking non-fatal adverse events. However, if death occurs within the serious adverse event reporting period after the end of the study treatment, it must be reported, regardless of whether the patient is receiving other treatments.

11.2.5. Hospitalization

Adverse events that result in hospitalization or prolongation of hospital stay should be considered serious adverse events. Any initial admission to a medical facility, even if for less than 24 hours, meets this criterion.

Hospitalization does not include the following situations:

- Rehabilitation facilities
- Sanatoriums
- Routine emergency room admissions
- Same-day surgeries (such as outpatient/day/non-bedridden procedures) where the hospitalization or prolongation of stay is not related to a worsening of the adverse event. For example: Admission for an existing disease without the occurrence of a new adverse event or worsening of the existing disease (such as to investigate a laboratory abnormality that has persisted since before the trial);
- Hospitalizations for administrative reasons (such as annual routine physical examinations);
- Hospitalizations specified in the clinical trial protocol (such as procedures required by the trial protocol);
- Elective hospitalizations unrelated to a worsening of the adverse event (such as elective cosmetic surgery);
- Pre-scheduled treatments or surgeries should be documented in the entire trial protocol and/or participant's baseline information;
- Hospitalization solely for the use of blood products.

Diagnostic or therapeutic invasive (such as surgery) and non-invasive procedures should not be reported as adverse events. However, the condition leading to the procedure, if it meets the definition of an adverse event, should be reported. For example, acute appendicitis that occurs during the adverse event reporting period should be reported as an adverse event, and the resulting appendectomy should be recorded as a treatment for that adverse event.

12. Serious Adverse Event Reporting System

Reporting of serious adverse events should start from the moment a participant signs the informed consent form and continue until 28 calendar days (including the 28th day) after the last use of the study drug. During the trial, if a serious adverse event occurs, whether it is the initial report or a follow-up report, the investigator must immediately fill out the "Clinical Trial Serious Adverse Event (SAE) Report Form" for new drugs, sign it, and date it. Within

24 hours of becoming aware of the SAE, the investigator must notify the sponsor immediately and stop the participant's trial, taking measures to protect the participant. The investigator must track the SAE until it is resolved.

Serious adverse events should be meticulously documented, including symptoms, severity, time of occurrence, handling time, measures taken, follow-up timing and method, and outcome. If the investigator believes that a serious adverse event is not related to the study drug but potentially related to the study conditions (such as termination of the original treatment or complications during the trial), this relationship should be detailed in the narrative section of the medical record report form on the serious adverse event page.

13. SAE Reporting Procedures

Any serious adverse events that occur during the clinical study or within 28 days after discontinuation of the drug must be reported immediately in writing to the sponsor. After obtaining the primary efficacy endpoint results, any SAEs that occur during the period of observing secondary efficacy endpoint results must also be immediately reported to the sponsor. Additionally, the investigator must complete a Serious Adverse Event Report Form (SAE), detailing the time of occurrence, severity, relationship to the study drug, and measures taken, and sign the report.

14. Efficacy Evaluation

Efficacy evaluation is conducted using RECIST 1.1 criteria, including: (1) Evaluating the efficacy for each participant, which includes Complete Response (CR), Partial Response (PR), Stable Disease (SD), and Progressive Disease (PD). (2) Recording Progression-Free Survival (PFS), defined as the time from the start of study treatment to documented tumor progression or death from any cause, whichever comes first. Participants initially evaluated as CR or PR need confirmation after 4-6 weeks. Improvements or deteriorations in general condition are indicated by changes in ECOG scores before and after treatment.

15. Study Completion

The study concludes 24 months after the last participant is enrolled or earlier if the researcher deems it necessary to end the trial prematurely.

16. Safety Control Measures

1. Special Treatment

- Stop medication.

- Monitor vital signs: ECG, blood pressure, respiration, body temperature.

- Gastric lavage: 1%–2% sodium chloride solution or 1:5000 potassium permanganate solution.

- Laxative: 15–30 g sodium sulfate in 200 mL water for administration.

- Enema: 1% lukewarm soapy water (about 5000 mL) for high continuous cleansing.

2. Supportive Therapy

- Sedation and oxygen administration.

- Establish intravenous infusion access, open airways, and if necessary, provide tracheal intubation, external cardiac massage, and ventilatory support.

- Adequate fluid resuscitation to maintain circulating blood volume: Administer saline or glucose-sodium chloride solution intravenously, supplement with colloids according to the clinical condition to ensure osmotic pressure.

- Cardiotonics and vasopressors to maintain stable blood pressure and ensure blood supply to vital organs: Initially administer 20–60 mg corticosteroids with 50–250 mL of 5% glucose solution intravenously, followed by dopamine infusion to maintain stability.

- Diuretics according to urine output, administer appropriate doses of furosemide and sodium bicarbonate to alkalinize the urine.

- Maintain electrolyte and acid-base balance.

- Treat arrhythmias.

- Symptomatic treatment to maintain nitrogen balance.

3. Anti-Allergy

- Administer 10 mg of chlorpheniramine or 25–50 mg of promethazine; ensure the airway is

clear while the patient is lying down and breathing oxygen.

- 0.1% adrenaline 0.1–0.2 mL added to 5% glucose for intravenous drip.

- Use corticosteroids such as dexamethasone.

4. Preventive Use of Gastric Acid Suppressants

5. Correct Respiratory and Circulatory Failure

- Provide oxygen or artificial respiration, administer 0.375 g of phentolamine and 3–6 mg of lobeline alternately by injection every 15–30 minutes as needed, and 1–2 times by IV if necessary, along with cardiotonic drugs like digoxin preparations.

17. Recommended Phase II Clinical Trial Dosing Regimen

Based on the tolerance, preliminary efficacy, and drug exposure results from the continuous dosing study, a comprehensive analysis has determined the optimal dosing regimen to be pyrotinib at 320 mg/day and SHR6390 at 125 mg/day in combination with endocrine therapy, progressing to a Phase II clinical trial.

18. Ethical Standards and Informed Consent

18.1. Ethical Standards

This clinical trial must adhere to the Helsinki Declaration (1996 version), the Good Clinical Practice (GCP) guidelines issued by the CFDA, and relevant regulations.

18.2. Informed Consent

Participants must provide informed consent before receiving treatment in this trial, to protect their legal rights. Researchers must fully and comprehensively inform participants or their designated representatives about the study's purpose, the drug's effects, potential toxic side effects, and possible risks. It is essential that participants are made aware of their rights, the risks they are taking, and the benefits involved. The discussion is a crucial part of the informed consent process. If the participant and their legal representative are illiterate, the informed consent process must involve a witness. After the participant or their legal representative gives verbal consent, they should sign the informed consent form on the same

day as the witness. The informed consent form should specify the version and the date it was created or modified.

19. Quality Assurance in Clinical Trials

To ensure that this trial is conducted strictly according to the clinical research protocol, throughout the entire clinical trial process, both clinical researchers and sponsors must strictly adhere to the Good Clinical Practice (GCP) guidelines. It is essential to ensure that trial procedures are standardized, trial data is accurate, and research conclusions are reliable.

20. Data Handling

20.1. Researcher Data Entry Requirements

- For all patients who have signed the informed consent form and are screened as eligible to participate in the trial, every item in the case report form must be recorded carefully and in detail, with no blank or missing items (use a line to indicate any space that should not be left empty).

- All data in the case report forms must be verified against the participants' medical records to ensure accuracy.

- The case report forms serve as original data; any corrections must be made by drawing a line through the error, annotating the corrected data next to it, and including the researcher's signature with the date.

- Copies of lab reports should be affixed in the designated section for lab reports in the case report forms.

- Any data that are significantly high or outside the clinically acceptable range must be verified, with necessary explanations provided by the researcher, as per the instructions for filling out the case report forms.

20.2. Data Traceability and CRF Completion

The original records consist of the research medical records for proper preservation. The case report forms (CRFs) are derived from these research medical records and must be completed by the researcher for each selected case.

20.3.Data Selection for Analysis

1. Full Analysis Set (FAS)

According to the intention-to-treat (ITT) principle, efficacy analysis is conducted on all enrolled cases who have received the drug at least once. For cases where the full treatment process was not observed, the last observation carried forward (LOCF) method is used to impute the final trial outcomes.

2. Per-protocol Set (PPS)

Includes all cases that adhered to the trial protocol, demonstrated good compliance, did not use any prohibited medication during the trial, and completed the case report forms as required. No imputation is made for missing data. Efficacy analysis of the trial drug is conducted on the PPS.

3. Safety Analysis Set (SAS)

Comprises all enrolled patients who have used the trial medication at least once and have post-medication safety records. This dataset is used for safety analysis.

20.4.Undergoing Other Antineoplastic Treatments

The results of this trial will primarily use descriptive statistical methods. For quantitative data, means, standard deviations, medians, maximum and minimum values will be listed. For count data and ordinal data, frequencies (proportions), rates, and confidence intervals will be presented.

All statistical analyses will be conducted using SAS 9.2 statistical analysis software. All statistical tests will be two-sided, with a P-value of less than or equal to 0.05 considered statistically significant, and confidence intervals will be set at 95% confidence level.

1. Patient Baseline Characteristics

Calculate means, standard deviations, medians, maximums, and minimums for quantitative data such as age, height, and weight. Qualitative data like gender and ECOG scores will be listed with frequencies and percentages.

2. Tolerability Evaluation

Predominantly descriptive statistical analysis will be used, tabulating adverse events and reactions (where adverse reactions are defined as "adverse events related to the study drug as

'definitely related/very likely related/possibly related'") that occur in each dosage group in this trial. Laboratory test results will describe conditions that were normal before the trial but abnormal after treatment and the relationship of these changes to the study drug. Calculate means, standard deviations, medians, minimums, and maximums for vital signs (blood pressure, heart rate, body temperature, respiratory rate) and laboratory indicators before and after medication for each single-dose group, using paired t-tests for pre-post comparisons when necessary; trend tests will be performed for changes in vital signs and laboratory indicators between different dosage groups. For multiple-dose groups, comparisons will be made between each post-medication time point and before medication.

3. Efficacy Analysis

Tabular description of the clinical efficacy observed in this trial.

References

1. Siegel, R.L., Miller, K.D. & Jemal, A. Cancer statistics, 2018. *CA Cancer J Clin* **68**, 7-30 (2018).
2. Cardoso, F., *et al.* 1st International consensus guidelines for advanced breast cancer (ABC 1). *Breast* **21**, 242-252 (2012).
3. Goldhirsch, A., *et al.* Strategies for subtypes--dealing with the diversity of breast cancer: highlights of the St. Gallen International Expert Consensus on the Primary Therapy of Early Breast Cancer 2011. *Ann Oncol* **22**, 1736-1747 (2011).
4. Slamon, D.J., *et al.* Studies of the HER-2/neu proto-oncogene in human breast and ovarian cancer. *Science* **244**, 707-712 (1989).
5. Slamon, D.J., *et al.* Human breast cancer: correlation of relapse and survival with amplification of the HER-2/neu oncogene. *Science* **235**, 177-182 (1987).
6. Ponde, N., Brandao, M., El-Hachem, G., Werbrouck, E. & Piccart, M. Treatment of advanced HER2-positive breast cancer: 2018 and beyond. *Cancer Treat Rev* **67**, 10-20 (2018).
7. Velasco-Velazquez, M.A., *et al.* Examining the role of cyclin D1 in breast cancer. *Future Oncol* **7**, 753-765 (2011).
8. Casimiro, M.C., Velasco-Velazquez, M., Aguirre-Alvarado, C. & Pestell, R.G. Overview of cyclins D1 function in cancer and the CDK inhibitor landscape: past and present. *Expert Opin Investig Drugs* **23**, 295-304 (2014).
9. Finn, R.S., *et al.* Palbociclib and Letrozole in Advanced Breast Cancer. *N Engl J Med* **375**, 1925-1936 (2016).
10. Hortobagyi, G.N., *et al.* Ribociclib as First-Line Therapy for HR-Positive, Advanced Breast Cancer. *N Engl J Med* **375**, 1738-1748 (2016).
11. Goetz, M.P., *et al.* MONARCH 3: Abemaciclib As Initial Therapy for Advanced Breast Cancer. *J Clin Oncol* **35**, 3638-3646 (2017).
12. Goel, S., *et al.* CDK4/6 inhibition triggers anti-tumour immunity. *Nature* **548**, 471-475 (2017).
13. Goel, S., *et al.* Overcoming Therapeutic Resistance in HER2-Positive Breast Cancers with CDK4/6 Inhibitors. *Cancer Cell* **29**, 255-269 (2016).

Appendix 1: Performance Status Rating Scale (ECOG)

(Eastern Cooperative Oncology Group)

ECOG Score	Description
0	Fully active, able to carry on all pre-disease performance without restriction.
1	Restricted in physically strenuous activity but ambulatory and able to carry out work of a light or sedentary nature, e.g., light house work, office work.
2	Ambulatory and capable of all self-care but unable to carry out any work activities. Up and about more than 50% of waking hours.
3	Capable of only limited self-care, confined to bed or chair more than 50% of waking hours.
4	Completely disabled. Cannot carry on any self-care. Totally confined to bed or chair.
5	Dead.

Appendix 2: Creatinine clearance rate (CrCl)

Cockcroft-Gault Equation:

Serum Creatinine: in milligrams per deciliter (mg/dL):

$$\text{Males Creatinine Clearance (CrCl) (mL/min)} = \frac{(140-\text{age})\times\text{weight (kg)}^{a}}{72\times\text{serum creatinine (mg/dL)}}$$

$$\text{Females Creatinine Clearance (CrCl) (mL/min)} = \frac{0.85\times(140-\text{age})\times\text{weight (kg)}^{a}}{72\times\text{serum creatinine (mg/dL)}}$$

Serum Creatinine: in micromoles per liter (µmol/L):

$$\text{Males Creatinine Clearance (CrCl) (mL/min)} = \frac{(140-\text{age})\times\text{weight (kg)}^{a}}{0.81\times\text{serum creatinine (}\mu\text{mol/L)}}$$

$$\text{Females Creatinine Clearance (CrCl) (mL/min)} = \frac{0.85\times(140-\text{age})\times\text{weight (kg)}^{a}}{0.81\times\text{serum creatinine (}\mu\text{mol/L)}}$$

a Age is measured in years, and weight is in kilograms.

Appendix 3: Response evaluation criteria in solid tumors

Solid Tumor Response Evaluation Criteria Version 1.1 (Excerpt)

(New Response Evaluation Criteria in Solid Tumors：Revised RECIST Version 1.1)

Note: This attachment is an internal translation document, provided for reference only. Please refer to the English version for actual operations.

1 Background

Omitted

2 Objectives

Omitted

3 Tumor Measurability at Baseline

3.1 Definitions

At baseline, tumor lesions/lymph nodes will be categorized measurable or non measurable as follows:

3.1.1 Measurable

Tumor Lesions: At least one measurable diameter (recorded as the maximum diameter) with a minimum length as follows:

- CT scan: 10 mm (CT scan slice thickness not exceeding 5mm)
- Clinical routine examination instruments: 10 mm (lesions that cannot be accurately measured with a caliper should be recorded as non-measurable)
- Chest X-ray: 20 mm
- Malignant lymph nodes: Pathologically enlarged and measurable, with a single lymph node CT scan short axis ≥15 mm (CT scan slice thickness recommended not to exceed 5 mm). Only the short axis is measured and followed up at baseline and during follow-up.

3.1.2 Non-measurable

All other lesions, including small lesions (longest diameter <10 mm or pathological lymph nodes with ≥10 to <15 mm short axis), as well as truly non-measurable lesions are considered non-measurable. Lesions considered truly non-measurable include: leptomeningeal disease, ascites, pleural or pericardial effusion, inflammatory breast disease, lymphangitic involvement of skin or lung, and abdominal masses/abdominal organomegaly

identified by physical examination that is not measurable by reproducible imaging techniques.

3.1.3 Special Considerations Regarding Lesion Measurability

Bone lesions, cystic lesions, and lesions previously treated with local therapy require particular comment:

Bone lesions

- Bone scan, positron emission tomography scan or plain films are not considered adequate imaging techniques to measure bone lesions. However, these techniques can be used to confirm the presence or disappearance of bone lesions.
- Lytic bone lesions or mixed lytic-blastic lesions, with identifiable soft tissue components, that can be evaluated by cross sectional imaging techniques such as CT or MRI can be considered as measurable lesions if the soft tissue component meets the definition of measurability described above.
- Blastic bone lesions are non-measurable.

Cystic lesions

Lesions that meet the criteria for radiographically defined simple cysts should not be considered as malignant lesions (neither measurable nor non-measurable) since they are, by definition, simple cysts.

'Cystic lesions' thought to represent cystic metastases can be considered as measurable lesions, if they meet the definition of measurability described above. However, if noncystic lesions are present in the same subject, these are preferred for selection as target lesions.

Lesions with prior local treatment

Tumor lesions situated in a previously irradiated area, or in an area subjected to other loco-regional therapy, are not considered measurable unless there has been demonstrated progression in the lesion since the therapy.

3.2 Specifications by Methods of Measurements

3.2.1 Measurement of Lesions

All measurements should be recorded in metric notation. All baseline evaluations should be performed as close as possible to the treatment start and NEVER more than 4 weeks before the beginning of the treatment.

3.2.2 Method of Assessment

The evaluation of lesions at baseline and subsequent measurements should employ the same techniques and methods. Except for lesions that cannot be evaluated by imaging studies and are only assessable by clinical examination, all lesions must be evaluated using imaging studies.

Clinical Lesions: Clinical lesions can be considered measurable lesions only if they are superficial and have a diameter of ≥10 mm when measured (e.g., skin nodules). For patients with skin lesions, it is recommended to archive colored photographs with a ruler for measuring lesion size. Whenever possible, imaging evaluations should be prioritized over clinical assessments, as imaging is more objective and allows for reproducible review at the end of the study.

Chest X-Ray: When tumor progression is a significant study endpoint, priority should be given to chest CT scans, as CT is more sensitive than X-rays, especially for detecting new lesions. Chest X-rays are applicable only when the measured lesions have clear boundaries and good lung ventilation.

CT, MRI: CT is currently the preferred and repeatable method for evaluating therapeutic efficacy. The definition of measurability is based on CT scan slice thickness ≤5 mm. If the CT slice thickness exceeds 5 mm, the minimum measurable lesion size should be twice the slice thickness. MRI is also acceptable in some cases (e.g., whole-body scans).

Ultrasound: Ultrasound should not be used as a method for measuring lesion size. Due to its operator-dependent nature and lack of repeatability after measurement, ultrasound cannot ensure technical and measurement consistency between different assessments. If new lesions are detected by ultrasound during the trial, confirmation should be obtained using CT or MRI. MRI can be used as an alternative to CT considering the radiation exposure associated with CT.

Endoscopy, Laparoscopic Examination: These techniques are not recommended for the objective assessment of tumors. However, they can be used to confirm complete response (CR) when obtaining biopsy specimens, and can be used to confirm recurrence after CR in trials where recurrence or surgical resection is an endpoint.

Tumor Markers: Tumor markers cannot be used alone to evaluate objective tumor responses. However, if the marker levels exceed the upper limit of normal at baseline, they must return

to normal levels to assess complete response. Since tumor markers vary by disease, this factor should be considered when drafting measurement criteria in the protocol. Specific criteria for CA-125 relief (recurrent ovarian cancer) and PSA (recurrent prostate cancer) relief have been published. The International Gynecologic Cancer Society has also developed CA-125 progression criteria, which are likely to be included in the objective tumor assessment criteria for first-line ovarian cancer treatment protocols.

Cytology/Histology Techniques: In specified circumstances as outlined in the protocol, these techniques can be used to identify PR and CR (e.g., residual benign tumor tissue commonly exists in lesions of germ cell tumors). When exudate may be a potential side effect of therapy (e.g., treatment with taxanes or anti-angiogenic agents), and measurable tumors meet the criteria for relief or disease stabilization, the appearance or exacerbation of tumor-related exudate during treatment can be diagnosed through cytological techniques to differentiate between relief (or disease stabilization) and disease progression.

4 Tumor Response Evaluation

4.1 Assessment of Overall Tumor Burden and Measurable Disease

To evaluate objective relief or potential progression, it is necessary to perform a baseline assessment of the total tumor burden of all tumor lesions, as a reference for subsequent measurements. In clinical protocols where objective relief is the primary treatment endpoint, only patients with measurable lesions at baseline are eligible for inclusion. Measurable lesions are defined as having at least one lesion that can be measured. For trials where disease progression (time to progression or degree of progression at a fixed date) is the primary treatment endpoint, the inclusion criteria in the protocol must clearly specify whether eligibility is limited to patients with measurable lesions only or if patients without measurable lesions can also be included.

4.2 Baseline Documentation of ‘Target’ and ‘Nontarget’ Lesions

When more than 1 measurable lesion is present at baseline all lesions up to a total of 2 lesions per organ and a maximum of 5 lesions total (representative of all involved organs, with a maximum of 2 per organ) should be identified as target lesions and will be recorded and measured at baseline (this means in instances where subjects have only 1 or 2 organ sites involved a maximum of 2 and 4 lesions respectively will be recorded).

Target lesions should be selected on the basis of their size (lesions with the longest diameter), be representative of all involved organs, but in addition should be those that lend themselves to reproducible repeated measurements. It may be the case that, on occasion, the largest lesion does not lend itself to reproducible measurement in which circumstance the next largest lesion that can be measured reproducibly should be selected.

Lymph nodes merit special mention since they are normal anatomical structures that may be visible by imaging even if not involved by tumor. As noted above, pathological nodes that are defined as measurable and may be identified as target lesions must meet the criterion of a short axis of ≥15 mm by CT scan. Only the short axis of these nodes will contribute to the baseline sum of lesion diameters. The short axis of the node is the diameter normally used by radiologists to judge if a node is involved by solid tumor. Nodal size is normally reported as 2 dimensions in the plane in which the image is obtained (for CT scan this is almost always the axial plane; for MRI the plane of acquisition may be axial, sagittal, or coronal). The smaller of these measures is the short axis. For example, an abdominal node that is reported as being 20 mm × 30 mm has a short axis of 20 mm and qualifies as a malignant, measurable node. In this example, 20 mm should be recorded as the node measurement. Up to 2 nodal target lesions can be recorded. All other pathological nodes (those with short axis ≥10 mm but <15 mm) should be considered non-target lesions. Nodes that have a short axis <10 mm are considered non-pathological and should not be recorded.

A sum of the diameters (longest diameter for non-nodal lesions, short-axis diameter for nodal lesions) for all target lesions will be calculated and reported as the baseline sum of diameters. If lymph nodes are to be included in the sum, then as noted above, only the short axis is added into the sum. The baseline sum of diameters will be used as reference to further characterize any objective tumor regression in the measurable dimension of the disease.

All other lesions (or sites of disease) including pathological lymph nodes should be identified as non-target lesions and should also be recorded at baseline. Measurements are not required and these lesions should be followed as “present,” “absent,” or in rare cases “unequivocal progression.” In addition, it is possible to record multiple non-target lesions involving the same organ as a single item on the case report form (eg, ‘multiple enlarged

pelvic lymph nodes’ or ‘multiple liver metastases’).

4.3 Response Criteria

4.3.1 Evaluation of Target Lesions

Complete Response (CR): Disappearance of all target lesions. Any pathological lymph nodes (whether target or non-target) must have reduction in short axis to <10 mm.

Partial Response (PR): At least a 30% decrease in the sum of diameters of target lesions, taking as reference the baseline sum diameters.

Progressive Disease (PD): At least a 20% increase in the sum of diameters of target lesions, taking as reference the smallest sum on study (this includes the baseline sum if that is the smallest on study). In addition to the relative increase of 20%, the sum of diameters must also demonstrate an absolute increase of at least 5 mm. (Note: The appearance of one or more new lesions is also considered progression.)

Stable Disease (SD): Neither sufficient shrinkage to qualify for PR (taking as reference the sum of diameters at baseline) nor sufficient increase to qualify for PD (taking as reference the smallest sum of diameters while on study).

4.3.2 Special Notes on the Assessment of Target Lesions

Lymph nodes: Lymph nodes identified as target lesions should always have the actual short axis measurement recorded (measured in the same anatomical plane as the baseline examination), even if the nodes regress to below 10 mm on study. This means that when lymph nodes are included as target lesions, the ‘sum’ of lesions may not be zero even if CR criteria are met, since a normal lymph node is defined as having a short axis of <10 mm. For PR, SD, and PD, the actual short axis measurement of the nodes is to be included in the sum of target lesions.

Target lesions that become ‘too small to measure’: While on study, all lesions (nodal and non-nodal) recorded at baseline should have their actual measurements recorded at each subsequent evaluation, even when very small (eg, 2 mm). However, sometimes lesions or lymph nodes that are recorded as target lesions at baseline become so faint on CT scan that the radiologist may not feel comfortable assigning an exact measure and may report them as being ‘too small to measure.’ When this occurs, it is important that a value be recorded on the eCRF. If it is the opinion of the radiologist that the lesion has likely disappeared, the

measurement should be recorded as 0 mm. If the lesion is believed to be present and is faintly seen but too small to measure, a default value of 5 mm should be assigned (Note: It is less unlikely that this rule will be used for lymph nodes since they usually have a definable size when normal and are frequently surrounded by fat such as in the retro-peritoneum; however, if a lymph node is believed to be present and is faintly seen but too small to measure, a default value of 5 mm should be assigned in this circumstance as well. This default value is derived from the 5 mm CT slice thickness (but should not be changed with varying CT slice thickness.) The measurement of these lesions is potentially non-reproducible; therefore providing this default value will prevent false responses or progressions based upon measurement error. To reiterate, however, if the radiologist is able to provide an actual measure, that should be recorded, even if it is below 5 mm.

Lesions that split or coalesce on treatment: When non-nodal lesions “fragment,” the longest diameters of the fragmented portions should be added together to calculate the target lesion sum. Similarly, as lesions coalesce, a plane between them may be maintained that would aid in obtaining maximal diameter measurements of each individual lesion. If the lesions have truly coalesced such that they are no longer separable, the vector of the longest diameter in this instance should be the maximal longest diameter for the “coalesced lesion.”

4.3.3 Evaluation of Non-target Lesions

This section provides the definitions of the criteria used to determine the tumor response for the group of non-target lesions. While some non-target lesions may actually be measurable, they need not be measured and instead should be assessed only qualitatively at the time points specified in the protocol.

Complete Response: Disappearance of all non-target lesions. All lymph nodes must be non-pathological in size (<10 mm short axis).

Progressive Disease: Unequivocal progression (see comments below) of existing nontarget lesions. (Note: The appearance of one or more new lesions is also considered progression.)

Non-CR/Non-PD: Persistence of one or more non-target lesion(s).

4.3.4 Special Notes on Assessment of Progression of Non-target Disease

The concept of progression of non-target disease requires additional explanation as follows:

When the subject also has measurable disease: In this setting, to achieve ‘unequivocal progression’ on the basis of the non-target disease, there must be an overall level of substantial worsening in non-target disease such that, even in presence of SD or PR in target disease, the overall tumor burden has increased sufficiently to merit discontinuation of therapy. A modest “increase” in the size of 1 or more non-target lesions is usually not sufficient to qualify for unequivocal progression status. The designation of overall progression solely on the basis of change in non-target disease in the face of SD or PR of target disease will therefore be rare.

When the subject has only non-measurable disease: The same general concepts apply here as noted above; however, in this instance there is no measurable disease assessment to factor into the interpretation of an increase in non-measurable disease burden. Because worsening in non-target disease cannot be easily quantified (by definition: if all lesions are truly non-measurable) a useful test that can be applied when assessing subjects for unequivocal progression is to consider if the increase in overall disease burden based on the change in non-measurable disease is comparable in magnitude to the increase that would be required to declare PD for measurable disease (ie, an increase in tumor burden representing an additional 73% increase in “volume” [which is equivalent to a 20% increase diameter in a measurable lesion]). If ‘unequivocal progression’ is seen, the subject should be considered to have had overall PD at that time point. While it would be ideal to have objective criteria to apply to non-measurable disease, the very nature of that disease makes it impossible to do so; therefore, the increase must be substantial.

4.3.5 New Lesions

The emergence of new malignant lesions indicates disease progression; therefore, evaluation of new lesions is crucial. Currently, there are no specific criteria for the detection of new lesions by imaging; however, the discovery of a new lesion should be clear. For instance, progression cannot be attributed to differences in imaging techniques, changes in imaging morphology, or other non-tumor-related conditions (e.g., some so-called new bone lesions are simply the healing or recurrence of the original lesion). This is particularly important when partial or complete response of baseline lesions occurs; for example, necrosis of a liver lesion may be reported as a new cystic lesion on a CT scan report, when in fact it is not.

Lesions detected during follow-up that were not identified at baseline examinations will be considered new lesions and indicative of disease progression. For example, if a patient with visceral lesions detected at baseline examinations is found to have metastases during a CT or MRI examination of the head, the intracranial metastatic lesions in this patient will be considered evidence of disease progression, even if head examinations were not performed at baseline.

If a new lesion is ambiguous, such as due to its small size, further treatment and follow-up assessments are required to confirm whether it is indeed a new lesion. If repeated examinations confirm it to be a new lesion, then the time of disease progression should be calculated from its initial discovery.

Assessment of lesions using FDG-PET generally requires additional testing for confirmation. Combining FDG-PET and supplementary CT scan results to evaluate disease progression, especially for new suspicious lesions, is reasonable. New lesions can be clarified by FDG-PET examination according to the following procedure:

- If the baseline FDG-PET examination result is negative and subsequent follow-up FDG-PET examinations are positive, it indicates disease progression.
- If no baseline FDG-PET examination was conducted, and subsequent FDG-PET examinations are positive:
- If the new lesion identified in the follow-up FDG-PET examination corresponds to the CT scan results, it confirms disease progression.
- If the new lesion identified in the follow-up FDG-PET examination is not confirmed by CT scan results, confirmation by CT scan is required (if confirmed, disease progression time is calculated from the time of abnormality detected in the previous FDG-PET examination).
- If the new lesion identified in the follow-up FDG-PET examination corresponds to an existing lesion identified by CT scan and there is no progression on imaging, then there is no disease progression.

4.4 Evaluation of Best Overall Response

The best overall treatment response is the best efficacy record from the start to the end of the trial, taking into account any necessary conditions for confirmation. Sometimes, efficacy responses occur after the end of treatment, so the protocol should specify whether

post-treatment efficacy evaluations are considered in the best overall treatment response. The protocol must specify how any new treatments before progression affect the best treatment response. The best treatment response of patients mainly depends on the results of target lesions, non-target lesions, and the manifestation of new lesions. Additionally, it relies on the nature of the trial, protocol requirements, and outcome measurement criteria. Specifically, in non-randomized trials, the efficacy response situation is the primary objective, and confirmation of PR or CR efficacy is necessary to determine which is the best overall treatment response.

4.4.1 **Time Point Response**

Assuming efficacy responses occur at specific time points in each protocol, Table 1 will provide a summary of the overall treatment responses at each time point for the population of patients with measurable disease at baseline.

Table 1 Time Point Response: Subjects with Target Disease

Target Lesions	Non-target Lesions	New Lesions	Time Point Response
CR	CR	No	CR
CR	Non-CR/non-PD	No	PR
CR	NE	No	PR
PR	Non-PD or NE	No	PR
SD	Non-PD or NE	No	SD
NE	Non-PD	No	NE
PD	Any	Yes or No	PD
Any	PD	Yes or No	PD
Any	Any	Yes	PD

CR = complete response; PR = partial response; SD = stable disease; PD = progressive disease; NE = not evaluable

If patients have no measurable lesions (no target lesions), the assessment can refer to Table 2.

Table 2 Time Point Response: Subjects with non-target Disease

Non-target Lesions	New Lesions	Time Point Response
CR	No	CR
Non-CR/non-PD	No	Non-CR/non-PD [a]
NE	No	NE
Uncertain PD	Yes or No	PD
Any	Yes	PD

a：For non-target lesions, "non-CR/non-PD" refers to a response better than stable disease (SD). As SD is increasingly used as an endpoint to assess efficacy, defining non-CR/non-PD responses is necessary for

cases where no measurable lesions are specified.

4.4.2 Missing Assessments and Non-evaluable Designation

When no imaging/measurement is done at all at a particular time point, the subject is not evaluable (NE) at that time point. If only a subset of lesion measurements are made at an assessment, usually the case is also considered NE at that time point, unless a convincing argument can be made that the contribution of the individual missing lesion(s) would not change the assigned time point response. This would be most likely to happen in the case of PD. For example, if a subject had a baseline sum of 50 mm with 3 measured lesions and at follow-up only 2 lesions were assessed, but those gave a sum of 80 mm, the subject will have achieved PD status, regardless of the contribution of the missing lesion

4.4.3 Best Overall Response: All Time Points

Once all the patient data is available, their best overall response can be determined.

Assessment of best overall response when confirmation of complete or partial response is not required in the study:

The best response at all time points during the trial is considered (e.g., a patient may have stable disease (SD) at the first cycle assessment, partial response (PR) at the second cycle assessment, and progressive disease (PD) at the last cycle assessment, but their best overall response is PR). However, if the best overall response is SD, it must meet the minimum duration criteria specified in the protocol from baseline. If the criteria for the minimum duration are not met, even if the best overall response is SD, it will not be accepted, and the patient's best overall response will be determined by subsequent assessments. For example, a patient with SD at the first cycle assessment, followed by PD at the second cycle, but who did not meet the minimum duration requirement for SD, their best overall response will be PD. Similarly, a patient who is lost to follow-up after being assessed with SD at the first cycle will be considered non-evaluable.

Assessment of best overall response when confirmation of complete or partial response is required in the study:

Only when each subject meets the trial-specified criteria for complete or partial response and is specifically mentioned in the protocol to have the response confirmed at subsequent time

points (generally four weeks later) can it be declared as complete or partial response. In this case, refer to the explanation in Table 3 for the best overall response.

Table 3 **Best Overall Response Requiring Confirmation of CR and PR Efficacy**

Initial Time Point Overall Response	Subsequent Time Point Overall Response	Best Overall Response
CR	CR	CR
CR	PR	SD，PD or PR[a]
CR	SD	If the duration of SD is sufficient, it is classified as SD; otherwise, it should be classified as PD.
CR	PD	If the duration of SD is sufficient, it is classified as SD; otherwise, it should be classified as PD.
CR	NE	If the duration of SD is sufficient, it is classified as SD; otherwise, it should be classified as NE.
PR	CR	PR
PR	PR	PR
PR	SD	SD
PR	PD	If the duration of SD is sufficient, it is classified as SD; otherwise, it should be classified as PD.
PR	NE	If the duration of SD is sufficient, it is classified as SD; otherwise, it should be classified as NE.
NE	NE	NE

CR = complete response; PR = partial response; SD = stable disease; PD = progressive disease; NE = not evaluable

a: If a complete response (CR) genuinely occurs at the first time point, any disease that appears at subsequent time points means that even if the subject's efficacy reaches the partial response (PR) criteria relative to baseline, their efficacy evaluation at later time points will still be progressive disease (PD) (because the disease reappears after CR). The best remission depends on whether stable disease (SD) occurs within the shortest treatment interval. However, sometimes the first evaluation is CR, but subsequent time point scans suggest that small lesions still appear, so in fact, the subject's efficacy should have been PR rather than CR at the first time point. In this case, the initial CR judgment should be revised to PR, and the best response is PR.

4.4.4 Special Notes on Response Assessment

When nodular lesions are included in the total assessment of target lesions, and their size reduces to "normal" (<10 mm), they will still have a lesion size report on the scan. To prevent overestimation based on increases in nodule size, measurements will be recorded even when nodules are considered normal. As previously mentioned, this means that for subjects achieving complete remission (CR), the case report forms (CRFs) will not record a zero size.

If confirmation of response is required during the trial, repeated "non-measurable" time points can complicate the assessment of best response. The trial's analytical plan must specify

how these missing data/assessments can be clearly interpreted when determining response efficacy. For example, in most trials, a subject's response pattern of PR-NE-PR can be considered as confirmed efficacy.

When a subject exhibits overall health deterioration that necessitates cessation of drug administration without objective evidence, it should be reported as symptomatic progression. Objective progression should be assessed as much as possible even after treatment discontinuation. Symptomatic worsening is not a description of an objective response; it is a reason for stopping treatment. Such subjects' objective responses will be assessed based on target and non-target lesion conditions as shown in Tables 1 to 3.

Situations defined as early progression, early death, and unassessable should be explicitly described in each protocol, depending on the treatment intervals and cycles.

In some cases, it can be difficult to distinguish local lesions from normal tissue. When a complete remission assessment is based on such definitions, it is recommended to perform a biopsy before assessing complete remission of local lesions. When imaging results of some subjects' local lesions are considered indicative of lesion fibrosis or scar formation, FDG-PET is regarded as a similar standard to biopsy for confirming efficacy in complete remission. In such cases, the use of FDG-PET should be prospectively described in the protocol, supported by reports from the relevant medical literature. However, it is important to be aware of the limitations of both FDG-PET and biopsy (including their resolution and sensitivity), which may lead to false positive results in assessing complete remission.

Treatment may continue until the next assessment for unclear progression findings (such as very small, indeterminate new lesions; cystic changes or necrosis of existing lesions). If disease progression is confirmed at the next assessment, the progression date should be backdated to the date when suspected progression was first noted.

4.5 Frequency of Tumor Re-evaluation

The frequency of tumor re-evaluation during treatment is determined by the treatment plan and should align with the type and schedule of therapy. However, in Phase II trials where the benefit of treatment is unclear, it is reasonable to conduct follow-ups every 6-8 weeks (timed at the end of a cycle), with adjustments possible in special protocols or circumstances. The protocol should specifically indicate which anatomical sites require baseline assessment

(usually those most likely associated with metastasis of the tumor type under study) and the frequency of repeated evaluations. Normally, both target and non-target lesions should be assessed at each evaluation. In some optional scenarios, the frequency of non-target lesion evaluation may be reduced, for example, when the efficacy evaluation of the target disease confirms a complete response (CR) or when a bone scan is repeated only if there is suspected progression of osseous lesions.

After the treatment ends, re-evaluation of the tumor depends on whether response rate or time to an event (progression/death) is used as the clinical trial endpoint. For time to an event (e.g., TTP/DFS/PFS), routine repeat evaluations as specified in the protocol are required. Particularly in randomized comparative trials, scheduled evaluations should be listed in the timeline (e.g., 6-8 weeks during treatment, or 3-4 months post-treatment) and should not be affected by other factors such as treatment delays, dosing intervals, or any other events that could cause imbalance in treatment arms regarding the timing of disease evaluation.

4.6 Assessment of Efficacy/Confirmation of Remission

4.6.1 Confirmation

For non-randomized clinical studies where efficacy is the primary endpoint, confirmation of Partial Response (PR) and Complete Response (CR) must be obtained to ensure that the efficacy is not the result of assessment error. This also allows for a reasonable interpretation of the results in the context of historical data, but the efficacy in these historical data should also have been confirmed. However, in all other cases, such as randomized trials (Phase II or III) or studies where the primary endpoints are disease stabilization or disease progression, confirmation of efficacy is no longer required, as it does not add value to the interpretation of the trial results. Nevertheless, the removal of the requirement for efficacy confirmation makes the role of central review to prevent bias more important, especially in open-label studies.

In the case of Stable Disease (SD), at least one measurement meeting the SD criteria specified in the protocol should occur within the shortest interval after the start of the trial, generally not less than 6-8 weeks.

4.6.2 Duration of Overall Remission

Duration of Overall Remission is measured from the time the criteria for Complete Response (CR) or Partial Response (PR) (whichever is measured first) are met, to the time the disease

is first officially recorded as recurring or progressing (using the smallest measurement recorded during the trial as a reference for disease progression). Duration of Complete Remission is measured from the time the criteria for CR are first met, to the time the disease is first officially recorded as recurring or progressing.

4.6.3 Duration of Stable Disease

Duration of Stable Disease is defined as the time from the start of treatment to the time of disease progression (from the time of randomization in randomized trials), using the smallest total sum as a reference (if the baseline sum is the smallest, it is used as the reference for calculating Progressive Disease (PD)). The clinical relevance of the duration of stable disease varies between different studies and diseases. If a trial specifically uses the proportion of patients maintaining stable disease for a minimum duration as a study endpoint, the protocol should explicitly specify the minimum interval between two measurements for defining Stable Disease (SD).

Note: The durations of remission, stable disease, and Progression-Free Survival (PFS) are influenced by the frequency of follow-up assessments after baseline evaluation. Defining standard follow-up frequencies is beyond the scope of these guidelines. Follow-up frequency should consider various factors, such as disease type and stage, treatment cycles, and standard protocols. However, the limitations of the accuracy of these measurement endpoints should be considered if comparisons between trials are necessary.

4.7 PFS/TTP

4.7.1 Phase II Clinical Trials

This guideline primarily focuses on the use of objective response as a study endpoint in Phase II clinical trials. In some cases, response rates may not be the optimal choice for evaluating the potential anti-cancer activity of a new drug/new regimen. In these instances, progression-free survival (PFS) or progression-free probability (PPF) at a defined time point can be considered suitable alternative indicators for providing initial signals of the new drug's biological activity. However, it is clear that in a non-controlled trial, these assessments can be questioned because seemingly valuable observations may be related to biological factors such as patient selection, rather than the effect of the drug intervention. Therefore, Phase II clinical trials using these as study endpoints are best designed as randomized controlled trials.

However, in certain tumors with consistently poor clinical outcomes, non-randomized trials are also reasonable. In these cases, due to the absence of a positive control, care must be taken in documenting efficacy evidence when assessing expected PFS or PPF.

CLINICAL STUDY PROTOCAL

Pyrotinib, LEtrozole And SHR6390 in subjects with dUal-Receptor positive(ER+/HER2+) Advanced Breast cancer: a muLti-center phasE Ib/II study

PLEASURABLE
(LORDSHIPS 3.0/YBCSG-20-01)

VERSION 1.1, February 5, 2021

Xichun Hu，Fudan University Shanghai Cancer Center

TABLE OF CONTENTS

PROTOCAL SYNOPSIS 4

SCHEDULE OF EVENTS 14

LIST OF ABBREVIATIONS 20

1. BACKGROUND 22
- 1.1. INVESTIGATIONAL PRODUCT 26
- 1.2. THE PHARMACOLOGICAL TYPE AND MECHANISM FOR SHR6390 26
- 1.3. SHR6390 PHARMACODYNAMICS 26
- 1.4. SHR6390 TOXICOLOGY 26
- 1.5. SHR6390 PHARMACOKINETICS 26
- 1.6. IN VIVO ANTITUMOR ACTIVITY OF PYROTINIB AND SHR6390 IN ER+, HER2+ BREAST CANCER MODELS 27

2. CLINICAL STUDIES 27
- 2.1. PYROTINIB MALATE 27
- 2.2. SHR6390 27
- 2.3. MARKETED CDK4/6 INHIBITORS 28

3. STUDY OBJECTIVES AND STUDY ENDPOINTS 29
- 3.1. PRIMARY STUDY OBJECTIVE 29
- 3.2. PRIMARY ENDPOINT 29
- 3.3. SECONDARY ENDPOINTS 30

4. INVESTIGATIONAL DRUGS 30
- 4.1. NAME AND SOURCE 30
- 4.2. DOSAGE FORM AND SPECIFICATIONS 30
- 4.3. STORAGE 30
- 4.4. ADMINISTRATION 31
- 4.5. MANAGEMENT, DISTRIBUTION, AND RETRIEVAL 31

5. OVERALL STUDY DESIGN 31

6. STUDY DESIGN 31
- 6.1. DOSAGE AND ADMINISTRATION 31
- 6.2. DOSE-LIMITING TOXICITY (DLT) 33
- 6.3. MAXIMUM TOLERATED DOSE (MTD) 33
- 6.4. SUBJECT REPLACEMENT 33
- 6.5. PLANNED SAMPLE SIZE 34

7. SAMPLE COLLECTION 34

8. PARTICIPANTS 35
- 8.1. SUBJECTS AND SAMPLE SIZE 35
- 8.2. INCLUSION CRITERIA 35
- 8.3. EXCLUSION CRITERIA 37
- 8.4. PARTICIPANT IDENTIFICATION 38

8.5. Dropout Criteria.. 38
8.6. Termination Criteria for Participant Treatment... 38
8.7. Study Termination Criteria.. 39
9. DOSE ADJUSTMENT AND CONCOMITANT MEDICATION.. 39
9.1. DLT Observation Period 39
9.2. Continuous Dosing in Cycle 2 and Subsequent Cycles.. 39
9.3. Prohibited Medications 40
9.4. Permitted Medications.. 41
10. STUDY PROCEDURES 41
10.1. Screening period... 41
10.2. Trial Period 43
10.3. Study termination/withdrawal 44
10.4. Follow-up after treatment completion... 45
11. SAFETY ASSESSMENT 45
11.1. Adverse Event (AE).. 45
11.2. Serious Adverse Events (SAE) 48
12. SERIOUS ADVERSE EVENT REPORTING SYSTEM... 50
13. SAE REPORTING PROCEDURES.. 50
14. EFFICACY EVALUATION 51
15. STUDY COMPLETION 51
16. SAFETY CONTROL MEASURES.. 51
17. RECOMMENDED PHASE II CLINICAL TRIAL DOSING REGIMEN........................... 52
18. ETHICAL STANDARDS AND INFORMED CONSENT 52
18.1. Ethical Standards 52
18.2. Informed Consent 53
19. QUALITY ASSURANCE IN CLINICAL TRIALS... 53
20. DATA HANDLING.. 53
20.1. Researcher Data Entry Requirements 53
20.2. Data Traceability and CRF Completion... 54
20.3. Data Selection for Analysis 54
20.4. Undergoing Other Antineoplastic Treatments 54
REFERENCES.. 56
APPENDIX 1: PERFORMANCE STATUS RATING SCALE (ECOG)................................. 57
APPENDIX 2: CREATININE CLEARANCE RATE (CRCL) 58
APPENDIX 3: RESPONSE EVALUATION CRITERIA IN SOLID TUMORS...................... 59

PROTOCAL SYNOPSIS

Study Title	A multicenter Phase Ib/II clinical study of pyrotinib and letrozole combined with the CDK4/6 inhibitor SHR6390 for the treatment of dual receptor-positive (ER+/HER2+) advanced breast cancer.
Protocal Number	PLEASURABLE (LORDSHIPS 3.0/YBCSG-20-01)
Version	1.1
Version Date	February 5, 2021
Lead Institution	Fudan University Shanghai Cancer Center
Principal investigator	Professor Hu Xichun
Coordinating investigator	Professor Zhang Jian, Dr. Meng Yanchun, Dr. Tao Zhonghua
Indication Under Investigation	ER receptor-positive, HER2-positive advanced breast cancer
Study Objectives	Phase I (Ib stage):<br>Determine the safety and tolerability of the combination of a non-steroidal aromatase inhibitor (letrozole), pyrotinib maleate tablets, and the CDK4/6 inhibitor SHR6390 in the treatment of hormone receptor-positive, HER2-positive advanced breast cancer. Based on preliminary efficacy data, establish the recommended Phase II dosage for this combination therapy.<br>Phase II:<br>To evaluate the efficacy and safety of the combination of pyrotinib, letrozole, and the CDK4/6 inhibitor SHR6390 in the treatment of dual receptor-positive (ER+, HER2+) advanced breast cancer.

Study Endpoints	Phase I<br><br>Primary Endpoint:<br>• For the combination regimen, determine the dose-limiting toxicity (DLT) and maximum tolerated dose (MTD) of SHR6390, establishing the recommended dosing regimen for the Phase II clinical study.<br>• Assess the incidence and severity of adverse events (AEs) and serious adverse events (SAEs) across different dose groups.<br><br>Secondary Efficacy Endpoints:<br>• Objective response rate (ORR), according to RECIST 1.1<br>• Progression-free survival (PFS)<br>• Disease Control Rate (DCR, The proportion of subjects with a best overall response of complete response, partial response, or stable disease).<br>• Clinical benefit rate (CBR, proportion of complete response, partial response, or stable disease for at least 24 weeks)<br>• Duration of response (DOR)<br>• Pharmacokinetics<br>• AEs and SAEs<br><br>Phase II:<br><br>Primary Endpoint:<br>• Objective response rate (ORR), according to RECIST 1.1<br><br>Secondary Efficacy Endpoints:<br>• Progression-free survival (PFS)<br>• Disease Control Rate (DCR, The proportion of subjects with a best overall response of complete response, partial response, or stable disease).<br>• Clinical benefit rate (CBR, proportion of complete response, partial response, or stable disease for at least 24 weeks)<br>• Duration of response (DOR)<br>• Exploratory analysis of the relationship between molecular markers and efficacy<br>• Pharmacokinetics<br>• AEs and SAEs
Planned Sample Size	Phase I (Ib stage): Each dose group will include 3－6 subjects, with a total of 6－12 subjects in Groups A and B. If additional backup dose groups C, D, E, or F are used, each of these groups will add 3－6 more subjects.

<table>
<tr><td></td><td>Phase II: First-line anti-HER2 treatment: 39 subjects; Second-line anti-HER2 treatment: 28 subjects.</td></tr>
<tr><td>Study Design</td><td>Phase I (Ib stage): A single-arm, open-label, dose-escalation Ib clinical study.
Phase II: A single-arm, open-label, multicenter Phase II clinical study.</td></tr>
<tr><td>Study Treatment</td><td>Phase I (Ib stage):
In this study, the dosing regimen includes letrozole at 2.5 mg/day or anastrozole at 1 mg/day, with pyrotinib at either 400 mg/day or 320 mg/day. SHR6390 is organized into three dose groups.
<table>
<tr><th>Dose groups</th><th>SHR6390</th><th>pyrotinib</th><th>Patients</th></tr>
<tr><td>A</td><td>125 mg/d</td><td>400 mg/d</td><td>3~6</td></tr>
<tr><td>B</td><td>150 mg/d</td><td>400 mg/d</td><td>3~6</td></tr>
<tr><td>C(backup group)</td><td>100 mg/d</td><td>400 mg/d</td><td>3~6</td></tr>
<tr><td>D(backup group)</td><td>125 mg/d</td><td>320 mg/d</td><td>3~6</td></tr>
<tr><td>E(backup group)</td><td>150 mg/d</td><td>320 mg/d</td><td>3~6</td></tr>
<tr><td>F(backup group)</td><td>100 mg/d</td><td>320 mg/d</td><td>3~6</td></tr>
</table>
In the regimen combining non-steroidal aromatase inhibitors (NSAIs), pyrotinib maleate tablets, and SHR6390, the starting dose for SHR6390 is set at 125 mg. Following a 3+3 dose-escalation design, subsequent doses will be adjusted by increments of 25 mg based on the occurrence of dose-limiting toxicity (DLT) at the starting dose group:
➢ If no DLT at the specified frequency is observed in the initial dose group (125 mg, Group A), the dose will be escalated to the 150 mg group. If the 150 mg group exhibits DLT at the specified frequency, then the 125 mg dose will be established as the maximum tolerated dose (MTD).
➢ If the initial dose group (125 mg, Group A) shows DLT at the specified frequency, investigators will initiate both backup groups C and D. If C is also intolerable, backup Group F will be initiated. Groups D and E will be introduced sequentially (using the same dosing escalation principles), and if Group D remains intolerable, backup Group F will be implemented. The research team will analyze the data to determine the recommended Phase II dosing for</td></tr>
</table>

the combination therapy.

- If Group F remains intolerable, the research team will analyze the trial data and decide whether to terminate the study.
- Once the recommended Phase II dose for the letrozole, pyrotinib maleate, and SHR6390 combination therapy has been established, a parallel group using anastrozole, pyrotinib maleate, and SHR6390 will be initiated. If intolerable toxicity is observed in this group, dose adjustments will be made after discussion among the researchers.

DLT assessments will be conducted at the end of one treatment cycle for each dose group.

The specific dosing regimens for the three drugs are as follows:

- Non-steroidal Aromatase Inhibitor (NSAI): Letrozole, taken orally, 2.5 mg (or 1 mg), once daily on an empty stomach, with a 28-day cycle.
- Pyrotinib: Taken orally at 400 mg or 320 mg once daily, within 30 minutes after breakfast, with a 28-day cycle.
- SHR6390: Taken orally once daily on an empty stomach (ensuring fasting for at least 1 hour before and after dosing). The drug is administered on a 28-day cycle, with continuous dosing during the first 3 weeks of each cycle (D1－21), concurrent with letrozole, followed by a week of rest (D22－28).

The initial two cycles are designated as the core trial phase. Subjects evaluated with CR, PR, or SD at the end of the second cycle may continue on the assigned combination regimen until disease progression, intolerable toxicity, or withdrawal of informed consent.

Based on the recommended Phase II dosing established in Phase I, the expansion study in Phase II will administer pyrotinib at 320 mg, letrozole at 2.5 mg, and SHR6390 at 125 mg. Treatment will continue until disease progression, intolerable toxicity, or voluntary withdrawal of informed consent by the subject.

The specific regimen for the three drugs is as follows:

- Non-steroidal aromatase inhibitor: letrozole, administered orally at 2.5 mg once daily on an empty stomach, with continuous dosing for

	28 days per cycle.<br>• Pyrotinib: Orally, 320 mg, once daily, taken within 30 minutes after breakfast, continuously administered for 28 days as one cycle.<br>• SHR6390: Administered orally at 125mg once daily on an empty stomach (fasting required for at least 1 hour before and 1 hour after taking the medication), continuously for 21 days, followed by a seven-day off period, with each cycle lasting 28 days.
Dose-limiting toxicity (DLT)	DLT is defined as any of the following drug-related or possibly drug-related adverse events occurring within Cycle 1 (based on CTC-AE v4.0.3 criteria):<br>1. Hematologic Toxicity:<br>• Grade 4 neutropenia lasting ≥5 days;<br>• Grade 4 thrombocytopenia or Grade 3 thrombocytopenia with clinically significant bleeding;<br>• Grade ≥3 neutropenia with fever (≥38.0° C lasting 1 hour or >38.3° C);<br>• Grade ≥4 anemia.<br>2. Non-Hematologic Toxicity:<br>• Any Grade ≥3 non-hematologic toxicity, except for the following:<br>• Grade 3 – 4 nausea/vomiting and/or diarrhea and/or electrolyte imbalance that resolves to Grade ≤2 within 72 hours with optimal supportive treatment;<br>- Grade 3 – 4 elevations in alkaline phosphatase or gamma-glutamyl transferase clearly related to the tumor and unrelated to the drug.
Maximum tolerated dose (MTD)	Definition of Maximum Tolerated Dose (MTD):<br>During the dosing observation period in Cycle 1 (28 days), if DLTs are observed in ≥1/3 of the subjects in a given dose group, the previous dose group is determined as the MTD. If dose escalation reaches Group B in the Phase I trial without ≥1/3 of the subjects experiencing DLTs, the research team will discuss whether to initiate

	the backup dose group C and determine the recommended dose for the Phase II trial.
Inclusion Criteria	1. Participants voluntarily join the study, sign an informed consent form, and demonstrate good compliance.<br>2. Female patients aged 18 to 75 years (inclusive).<br>3. Patients with histologically confirmed recurrent/metastatic breast cancer, ER-positive and HER2-positive:<br>- HER2 positivity defined as 3+ by standard immunohistochemistry (IHC) and/or positive by in situ hybridization (ISH).<br>- ER positivity defined as ≥1% ER-expressing cells.<br>- Local recurrence must be confirmed by the researcher as inoperable.<br>4. At least one measurable extracranial lesion according to RECIST 1.1 criteria.<br>5. Postmenopausal women, or women who have undergone bilateral oophorectomy or are receiving ovarian function suppression.<br>6. Previous antitumor treatment guidelines:<br>- Maximum of one prior systemic therapy regimen for recurrent/metastatic breast cancer including anti-HER2 ADC:<br>i. If not previously treated with a trastuzumab regimen in the advanced stage, or if recurrence occurs more than one year after adjuvant trastuzumab treatment, subsequent therapy is considered first-line anti-HER2 treatment.<br>ii. If first-line treatment with a trastuzumab regimen fails, or if recurrence occurs during adjuvant trastuzumab treatment or within one year after its completion, subsequent therapy is considered second-line anti-HER2 treatment.<br>- No prior anti-HER2 TKI therapy or no proof of failure with such therapy.<br>- No prior evidence of resistance to aromatase inhibitors in endocrine therapy (resistance defined as recurrence during adjuvant aromatase inhibitor therapy or within one year after its completion, or disease progression after receiving aromatase inhibitor therapy during the

	recurrent/metastatic phase).<br>7. ECOG performance status of 0 – 1.<br>8. Expected survival of ≥12 weeks.<br>9. Adequate organ function (no use of any blood components and growth factors within 2 weeks prior to enrollment):<br>- Absolute neutrophil count ≥1.5×10^9/L;<br>- Platelets ≥90×10^9/L;<br>- Hemoglobin ≥90g/L;<br>- Total bilirubin ≤1.5 times upper limit of normal (ULN);<br>- ALT and AST ≤2.5 times ULN;<br>- Urea/Blood Urea Nitrogen (BUN) and creatinine (Cr) ≤1.5× ULN;<br>- Left ventricular ejection fraction (LVEF) ≥50%;<br>- Fridericia-corrected QT interval (QTcF) < 470 ms;<br>- INR ≤1.5×ULN, APTT ≤1.5×ULN.
Exclusion Criteria	1. Subjects with untreated central nervous system metastases.<br>2. Patients with a history of systemic or definitive treatment for brain or meningeal metastases (radiotherapy or surgery) may be included if imaging confirms stable disease for at least 4 weeks, they have discontinued systemic steroids therapy for more than 4 weeks, and they show no clinical symptoms.<br>3. Prior treatment with any CDK4/6 inhibitor.<br>4. Presence of symptomatic ascites, pleural effusion, or pericardial effusion requiring drainage at baseline, or who have undergone serous cavity fluid drainage within 4 weeks prior to first drug administration.<br>5. Inability to swallow, intestinal obstruction, or other factors affecting drug intake and absorption.<br>6. Received chemotherapy, molecular targeted therapy, or other systemic treatments including clinical trial drugs within 4 weeks prior to enrollment; received endocrine therapy within 2 weeks prior to enrollment.<br>7. History of other malignancies within the past 5 years or concurrent

	malignancy, except for cured basal cell carcinoma of the skin and cervical carcinoma in situ.<br>8. Major surgical procedures or significant trauma within 4 weeks prior to the first drug administration, or anticipated need for major surgery during the study.<br>9. Pregnant or breastfeeding women, women of childbearing potential with a positive baseline pregnancy test, or unwilling to use effective contraception.<br>10. Known hypersensitivity to any component of the study drugs.<br>11. Active HBV or HCV infection; stable hepatitis B (HBV viral copy number not exceeding the upper limit of normal) treated with medication and cured hepatitis C (HCV viral copy number below the detection limit of the assay) are excluded.<br>12. History of immunodeficiency, including HIV positivity, or any other acquired or congenital immunodeficiency diseases, or history of organ transplantation.<br>13. History of any cardiac disease, including: (1) angina; (2) arrhythmias requiring medication or clinically significant; (3) myocardial infarction; (4) heart failure; (5) any other heart disease deemed by the investigator as unsuitable for participation in the trial; severe cardiac or renal function abnormalities of grade ≥II found during the screening period.<br>14. According to the investigator's judgement, severe concomitant diseases that pose a risk to patient safety or affect the completion of the study (e.g., severe hypertension, diabetes, thyroid disorders).<br>15. History of definite neurologic or psychiatric disorders, including epilepsy or dementia.<br>16. Severe infection within 4 weeks prior to the first drug administration (e.g., requiring intravenous administration of antibiotics, antifungals, or antivirals according to clinical guidelines), or unexplained fever >38.3°C during the screening period or prior to the first administration.
Termination Criteria for	If any of the following conditions occur, the participant must withdraw/terminate treatment:

Participant Treatment	1. The participant withdraws informed consent and requests to exit.<br>2. Imaging examination shows disease progression.<br>3. Inability to tolerate toxicity.<br>4. Serious protocol violation, as assessed by the investigator warranting treatment termination.<br>5. Participant loss to follow-up or occurrence of a pregnancy event.<br>6. Other circumstances deemed necessary for participant withdrawal from the study by the investigator.
Study Termination Criteria	Study termination criteria include, but are not limited to:<br>1. Discovery of unexpected, significant, or unacceptable risks to participants;<br>2. The investigational drug/therapy is ineffective, or continuation of the trial is deemed futile;<br>3. The investigator decides to terminate the study due to reasons such as severe lag in participant enrollment or significant protocol violations.
Safety Assessment	Adverse events will be graded according to the CTCAE v4.0.3 standards. During the trial, adverse event logs must be accurately maintained, including the time of occurrence, severity, duration, measures taken, and outcomes of each event.
Efficacy Evaluation	Efficacy evaluations for enrolled participants involve imaging assessments every 2 cycles (±7 days) for the first 6 cycles and then every 3 cycles (±7 days) thereafter, continuing until disease progression or the initiation of new antitumor treatment. Tumor response will be assessed according to RECIST 1.1 criteria. Following disease progression or the start of new antitumor treatment, survival status will be followed up every 12 weeks.
Phase 2 Exploratory Study (Lead Institution or Conditional Participating	HER2-PET and FDG-PET<br>Patients will undergo HER2-PET and FDG-PET assessments at baseline, after 2 cycles, and upon progression to further explore their clinical utility in evaluating antitumor efficacy.<br>2. [redacted]<br>3. Circulating tumor DNA (ctDNA)

Institutions Only)	ctDNA will be collected at baseline, at the end of cycle 2, and prior to disease progression or the initiation of new antitumor treatment to serve as a biomarker for assessing antitumor efficacy.
Statistical Analyses	The primary analysis of this trial will primarily utilize descriptive statistical methods. For continuous data, mean, standard deviation, median, maximum, and minimum values will be presented. For categorical and ordinal data, frequencies (relative frequencies), rates, and confidence intervals will be provided.<br>All statistical analyses will be conducted using SAS 9.2 or above statistical analysis software.<br>Safety Analysis:<br>Descriptive statistics will be used to analyze adverse events, serious adverse events, and adverse events related to the investigational drug in each dose group. Laboratory test results will describe the occurrence of abnormalities after treatment compared to normal values before the trial.<br>Efficacy Analysis:<br>Point estimates of objective response rate (ORR), disease control rate (DCR), clinical benefit rate (CBR), and other efficacy endpoints will be provided along with their 95% confidence intervals, representing the population. Survival outcomes will be assessed using Kaplan-Meier methods to estimate median progression-free survival, 12-month survival rates, and their 95% confidence intervals, with survival curves plotted. Descriptive analysis will be applied to other secondary efficacy endpoints.<br>Other Analyses:<br>The relationship between levels of molecular biomarkers that may affect efficacy and treatment outcomes will be explored.
Study Completion	The study will conclude 24 months after the last participant is enrolled or earlier if deemed necessary by the investigator.
Study duration	Estimated duration: November 2018 to November 2022

Schedule of Events

Procedures	Screening period		Treatment and Follow-up Period 28d/cycle				Post-treatment		Survival Follow-up
			Cycle 1			≥ Cycle 2			
	Day-28 to Day-1	Day-7 to Day-1	Day15 (±3d)	Day21 (±3d)	Day28 (±3d)	Day28 (±3d)	End of treatment/withdrawal	4 weeks after the last dose	every 12 weeks (±7d)
Baseline data									
Signed informed consent	×								
Demographic data	×								
Cancer History/other medical history[1]	×								
Concomitant medication[2]	×					×			
Laboratory examination									
Complete Blood Count (CBC)[3]		×	×		×	×	If not done within 7 days	In necessity	
Urinalysis[4]		×	Every 3 weeks				If not done within 7 days	In necessity	
Fecal Routine[5]		×	Every 3 weeks				If not done within 7	In necessity	

**Procedures**	**Screening period**		**Treatment and Follow-up Period 28d/cycle**				**Post-treatment**		**Survival Follow-up**
	**Day-28 to Day-1**	**Day-7 to Day-1**	**Cycle 1**			**⩾ Cycle 2**			
			**Day15** (±3d)	**Day21** (±3d)	**Day28** (±3d)	**Day28** (±3d)	**End of treatment/withdrawal**	**4 weeks after the last dose**	**every 12 weeks** (±7d)
							days		
Blood Biochemistry[6]		×	×		×	×	If not done within 7 days	In necessity	
Hepatitis B, Hepatitis C, and HIV Testing[7]	×								
Pregnancy Test[8]		×						In necessity	
Clinical evaluation and examination									
Adverse Events[9]	From the signing of the informed consent to 28 days after the last dose								
Vital Signs[10]		×	×		×	×	If not done within 7 days	×	
Physical Examination[11]		×	×		×	×	If not done within 7 days	×	
ECOG PS		×	×		×	×	If not done within 7 days	×	
12-Lead Electrocardiogram (ECG)[12]		×	×		×	×	If not done within 7 days	In necessity	
Echocardiogram[13]	×		Every 3 weeks				If not done within 4	In necessity	

Procedures	Screening period		Treatment and Follow-up Period 28d/cycle				Post-treatment		Survival Follow-up
			Cycle 1			≥ Cycle 2			
	Day-28 to Day-1	Day-7 to Day-1	Day15 (±3d)	Day21 (±3d)	Day28 (±3d)	Day28 (±3d)	End of treatment/withdrawal	4 weeks after the last dose	every 12 weeks (±7d)
							weeks		
	Research drugs								
Letrozole[14]			Take orally once daily on an empty stomach						
Pyrotinib[15]			Take orally once daily within 30 minutes after meals						
SHR6390 (dalpiciclib)[16]			Take orally once daily on an empty stomach						
	Efficacy evaluation								
Imaging examination[17]	×		Imaging evaluation was performed at the end of every 2 cycles (±7 days) and at the end of every 3 cycles (±7 days) after 6 cycles until the disease progressed or a new anti-tumor therapy was initiated						
[redacted][18]	×		[redacted]						
HER2-PET[19]	×		After the second cycle of treatment (±7 days) and after disease progression						
FDG-PET[19]	×		After the second cycle of treatment (±7 days) and after disease progression						
	Follow-up after treatment								

**Procedures**	**Screening period**		**Treatment and Follow-up Period 28d/cycle**				**Post-treatment**		**Survival Follow-up**
			**Cycle 1**			**⩾ Cycle 2**			
	**Day-28 to Day-1**	**Day-7 to Day-1**	**Day15** （±3d）	**Day21** （±3d）	**Day28** （±3d）	**Day28** （±3d）	**End of treatment/withdrawal**	**4 weeks after the last dose**	**every 12 weeks** （±7d）
Disease Progression Timing[20]							Until the disease progresses or a new anti-tumor therapy is initiated		
Survival Follow-Up[21]									×
	Blood collection and tumor sample collection/collection								
PK Collection[22]				×					
Biomarker Collection/Gathering[23]	×					×(Day 28 ±3 days of Cycle 2)	×(Before disease progression or start of new anti-tumor therapy)		

Notes:

[1] Cancer History/Other Medical History: Includes pathology results, ER/PR/HER2 testing reports; history of cancer surgery, chemotherapy, radiation therapy, and other disease treatments; history of cancers other than breast cancer.

[2] Concomitant Medication: Record medications and treatments used within 28 days prior to starting the study medication and during the study period. Once a participant discontinues trial treatment, only record concomitant medications and treatments used for new or unresolved adverse events related to the trial treatment。

[3] Complete Blood Count (CBC): Hemoglobin, red blood cells, white blood cells, neutrophils, lymphocytes, and platelet count.

[4] Urinalysis: Urine protein, glucose, occult blood (red cells, white cells). If semi-quantitative methods show protein 2+, a 24-hour urine protein quantitative test should be performed.

[5] Fecal Routine: Includes fecal occult blood.

[6] Blood Biochemistry: Total bilirubin, conjugated bilirubin, ALT, AST, AKP, γ-GT, LDH, total protein, albumin, urea/urea nitrogen, creatinine, uric acid, fasting glucose, triglycerides, cholesterol, potassium, sodium, chloride, calcium, phosphorus, magnesium; myocardial enzyme spectrum tests added as necessary.

[7] Hepatitis B, Hepatitis C, and HIV Testing: Hepatitis B five-marker test, and if results are abnormal, viral replication (HBV DNA) testing should be performed; Hepatitis C virus antibodies (anti-HCV), HIV antibodies.

[8] Pregnancy Test: Serum pregnancy test within one week before first medication use for women of childbearing age.

[9] Adverse Events: Record adverse events from the signing of the informed consent until at least 28 days after the last medication dose, and follow-up until adverse events have resolved or stabilized. If the participant begins new antitumor treatment, follow-up continues until the start of the tumor treatment.

[10] Vital Signs: Temperature, respiration, pulse, blood pressure.

[11] Physical Examination: Examination of major body systems (head, face, skin system, lymph nodes, eyes, ENT, oral cavity, respiratory system, cardiovascular system, abdomen, genitourinary system, musculoskeletal, nervous system, and mental status); comprehensive physical examination results recorded during screening and at the end of the study, with only abnormalities recorded during the trial.

[12] 12-Lead Electrocardiogram (ECG): If clinically significant abnormalities are found, the investigator may reconfirm if necessary.

[13] Echocardiogram: Follow-up on changes in LVEF values, additional unplanned checks if LVEF decreases to <50% and drops ≥10% from baseline, or if symptoms such as chest pain or palpitations occur.

[14] Non-steroidal aromatase inhibitor: letrozole, administered orally at 2.5 mg once daily, with continuous dosing for 28 days per cycle.

[15] Pyrotinib: Once daily, 320 mg, oral administration within 30 minutes after breakfast, continuous administration for 28 days per cycle.

[16] SHR6390 (dalpiciclib): Oral administration on an empty stomach, once daily, 125 mg, medication for 3 weeks (D1~21), off drug for 1 week (D22~28), 28 days per cycle. For SHR6390, take on an empty stomach in the morning with warm water; no eating 1 hour before and after taking the medication during the continuous administration period.

[17] Imaging examination: Imaging examination during the screening period include enhanced CT or MRI of the chest and abdomen, and CT/MRI of other suspected lesion sites (such as neck, pelvis, or brain). Tumor baseline assessment may be relaxed to within 4 weeks before the first medication dose, and CT/MRI results obtained before signing the informed consent can be used for tumor assessment during the screening period if they meet the requirements. Bone scans are necessary when clinical suspicion of bone metastasis exists. Imaging examination during the treatment period should be conducted under the same conditions as the baseline studies (scan slice thickness, contrast agent use, etc.), before the 6th cycle of medication, once every two cycles for lesions identified at baseline (bone scans conducted when bone progression is suspected or for CR confirmation), then every three cycles thereafter; if new lesions are suspected, timely checks are appropriate. Initial PR/CR should be confirmed 4-6 weeks later. The imaging examination schedule allows a window period of ±7 days. Unplanned imaging studies may be conducted if disease progression (e.g., worsening symptoms) is suspected.

[18] [redacted]

[19] HER2-PET and FDG-PET: Performed at baseline, end of cycle 2, and at disease progression (available at lead and participating sites with the capability).

[20] Disease Progression Timing: For participants ending trial treatment for reasons other than confirmed imaging progression, if imaging evaluation has not been performed within

4 weeks prior to the end of the trial, imaging should be conducted at the end of treatment, and tumor response follow-up should continue according to the protocol's specified frequency until documented disease progression or the start of a new antitumor treatment.

[21] Survival Follow-Up: After the end of trial treatment, survival status and subsequent antitumor treatment information may be collected every 3 months via clinical or telephone follow-up, until death.

[22] PK Collection: In Phase II, collect plasma within 0.5 hours before pyrotinib dosing on day 21 of the 1st cycle and 2 hours ±5 min, 4 hours ±10 min, 6 hours ±10 min, 12 hours ±10 min, 24 hours ±0.5 hour after dosing, and before dosing on day 22 of cycles 3, 5, 8, and 12, with medication times fixed relative to 3 days before PK blood collection.

[23] **Biomarker Collection/Gathering: At baseline, end of cycle 2, and before disease progression/new antitumor treatment, collect [redacted] and ctDNA samples; collect existing paraffin-embedded tumor tissue samples or ≥10 slides, and attempt to obtain biopsy samples from metastatic lesion.**

LIST OF ABBREVIATIONS

ABBREVIATION	DEFINITION
ALT	alanine aminotransferase
AST	aspartate aminotransferase
Cr	creatinine
CR	complete response
CRF	case report form
CDK	cyclin-dependent kinase
CYP	cytochrome P450
bid	twice a day
BUN	blood urea nitrogen
dL	deciliter
EC	ethics committee
ECG	electrocardiogram
ECOG	Eastern Oncology Collaboration Group
EGFR	epidermal growth factor receptor
ER	estrogen receptor
g	gram
GCP	Good Clinical Practice
GGT	glutamyltransferase
h	hour
Hb	hemoglobin
HER2	human epidermal growth factor receptor-2
HR	hormone receptor
IB	Investigator's Brochure
ISH	immunohistochemistry
INR	International Normalized Ratio
IU	international unit
IV	intravenous(ly)

ABBREVIATION	DEFINITION
kg	kilogram
kPa	kilopascal
LDH	interstitial lung disease
m	meter
min	minutes
mg	milligram
mL	milliliter
mm	millimeter
MBC	metastatic breast cancer
MTD	maximum tolerated dose
NCI-CTC	National Cancer Institute General Toxicity Criteria
ORR	objective response rate
PDX	patient-derived xenografts
PFS	progression-free survival
PLT	platelet
PR	partial response
qd	once a day
RBC	red blood cell count
SAE	serious adverse event
SAP	statistical analysis plan
TBIL	total bilirubin
UNL	upper limit of normal
WBC	white blood cell count

1. Background

Breast cancer has become the most common malignant tumor among women worldwide, accounting for approximately 25% of all malignant tumors in women. Early-stage breast cancer can be cured, but the median overall survival (OS) of patients with Metastatic Breast Cancer (MBC) is only 2 to 3 years. Studies have shown that breast cancer is a highly heterogeneous disease at the molecular level, and there are significant differences in treatment efficacy and survival among different molecular subtypes of breast cancer. Breast cancer subtyping guides treatment selection and prognosis. The 12th St Gallen Conference expert panel classified breast cancer into four subtypes: Luminal A, Luminal B, HER2-positive, and Triple-negative. Among them, Luminal B can be further divided into Luminal B (HER2-negative) and Luminal B (HER2-positive) based on HER2 status. Research has shown that endocrine therapy combined with anti-HER2 targeted therapy is an effective option for treating Luminal B (HER2-positive) MBC patients.

The epidermal growth factor receptor (EGFR) is a family of transmembrane receptors with tyrosine kinase activity, which includes HER1 (erbB1, EGFR), HER2 (erbB2, NEU), HER3 (erbB3), and HER4 (erbB4). The HER2 gene is amplified/overexpressed in more than 30% of human tumors, including breast cancer, ovarian cancer, endometrial cancer, and others. In the clinical diagnosis and treatment of breast cancer, HER2 is an important prognostic factor distinct from tumor size, lymph nodes, and hormone receptors, and is also an independent prognostic factor for breast cancer recurrence and survival. 20% to 30% of primary invasive breast cancers have amplification/overexpression of the HER2 gene, and targeted HER2 molecular drugs significantly prolong the survival of HER2-positive patients. In 1998, Roche's trastuzumab (Herceptin) was launched in the United States, resulting in significant improvements in clinical response rate and survival for HER2-positive patients. Over a decade later, Roche developed pertuzumab (Perjeta) and T-DM1 (ado-trastuzumab emtansine), which were approved for marketing in the United States in 2012 and 2013 respectively, for second- and third-line treatment after trastuzumab resistance. While there have been rapid advances in the development of large molecular HER2 antibodies, lapatinib (TYKERB), the first small molecule inhibitor targeting HER2, developed by GlaxoSmithKline, was launched in the United States in 2007 and in China in 2013, and used in combination with capecitabine for second-line treatment of HER2-positive advanced breast cancer. Meanwhile, neratinib (Nerlynx), a small molecule inhibitor of EGFR/HER2 for breast

cancer treatment developed by Puma Biotechnology, was approved for marketing in 2017. Pyrotinib maleate tablets, independently developed by Jiangsu Hengrui Medicine Co., Ltd., is an irreversible dual-target tyrosine kinase inhibitor against EGFR and HER2. Clinical trial data for stage II HER2-positive advanced or metastatic breast cancer patients who have failed anthracycline and taxane therapy and have received no more than 2 lines of chemotherapy, show that the combination of pyrotinib maleate tablets with capecitabine significantly improves the objective response rate (78.5% vs. 57.1%) and significantly prolongs progression-free survival (18.1 months vs. 7.0 months), reducing the risk of disease progression or death by 63.7% (HR=0.363), with good tolerability. Based on the efficacy and safety data obtained from current phase II clinical trials, the China National Medical Products Administration has agreed to accept Jiangsu Hengrui Medicine's application for conditional approval of pyrotinib maleate tablets for marketing.

Cyclin-dependent kinases (CDKs) are key enzymes in the regulation of the cell cycle, involved in physiological processes such as cell proliferation and survival. During cell proliferation, the complex formed by CDK4/6 and cellular cyclin D can phosphorylate the retinoblastoma protein (Rb). Once phosphorylated, Rb releases the transcription factor E2F, which, when bound in its unphosphorylated state, is tightly bound. The activation of E2F further promotes transcription that drives the cell cycle past the restriction point (R-point) from the growth phase (G1 phase) to the DNA replication phase (S phase), entering the cell proliferation phase. CDK4/6 inhibitors block cell proliferation at the G1 phase, thus achieving the purpose of inhibiting tumor proliferation. Currently, there are three CDK4/6 inhibitors on the global market: Palbociclib developed by Pfizer, Ribociclib by Novartis, and Abemaciclib by Eli Lilly, used for the treatment of hormone-receptor (HR) positive (ER+ and/or PR+), HER2-negative advanced or metastatic breast cancer patients.

The PALOMA-2 study, a phase III double-blind, randomized controlled trial, investigated the first-line treatment of advanced ER+/HER2-negative breast cancer with palbociclib in combination with letrozole. A total of 666 patients were enrolled. The study's primary endpoint showed that the combination of palbociclib and letrozole significantly prolonged the median progression-free survival (PFS) (24.8 months vs. 14.5 months) compared to letrozole alone, achieving a breakthrough of over two years in PFS for first-line treatment of advanced breast cancer. The most common adverse events of grade 3 or 4 were neutropenia, leukopenia, anemia, and fatigue.Similarly, the efficacy and safety of ribociclib combined with letrozole versus placebo combined with letrozole were assessed in a phase III randomized controlled trial for first-line treatment of HR-positive, HER2-negative recurrent or metastatic

postmenopausal breast cancer. The trial included 668 patients and demonstrated that ribociclib combined with letrozole significantly prolonged PFS (HR, 0.56; 95% CI, 0.43-0.72) and improved progression-free survival rates (63.0% vs. 42.2%) and overall response rates (52.7% vs. 37.1%, P <0.001). The most common adverse events of grade 3 or 4 reported in more than 10% of patients in both groups were neutropenia and leukopenia; the rates of discontinuation due to adverse events were 7.5% and 2.1%, respectively. The MONARCH-3 study, a double-blind, randomized phase III trial, investigated abemaciclib or placebo in combination with a non-steroidal aromatase inhibitor for first-line treatment of HR-positive, HER2-negative postmenopausal advanced breast cancer. A total of 493 patients were enrolled, with the abemaciclib group showing a significantly prolonged median PFS (not reached for the abemaciclib group vs. 14.7 months for the placebo group). The objective response rate was significantly higher in the abemaciclib group compared to the placebo group (59% vs. 44%). The most common adverse reaction in the abemaciclib group was diarrhea, and the most common grade 3 or 4 adverse events were neutropenia and leukopenia. Overall, the currently marketed CDK4/6 inhibitors (palbociclib, ribociclib, and abemaciclib) combined with non-steroidal aromatase inhibitors significantly improve progression-free survival and objective response rates, with a tolerable safety profile in patients with HR-positive, HER2-negative advanced breast cancer.

SHR6390 is an orally administered, highly efficient, and selective small molecule CDK4/6 inhibitor developed by Jiangsu Hengrui Medicine Co., Ltd.[redacted]

Research by Shom Goel and colleagues indicates that CDK4/6 inhibitors can activate anti-tumor immune function by increasing the expression of tumor cell antigens and inhibiting the proliferation of immunosuppressive regulatory T cells. Another study suggests that CDK4/6 inhibitors not only block Rb phosphorylation but also reduce TSC2 phosphorylation, thereby partially weakening mTORC1 activity. This reduces inhibition of upstream EGFR family kinases, making tumors more sensitive to EGFR/HER2 inhibitors. Therefore, dual inhibition of EGFR/HER2 and CDK4/6 leads to more effective inhibition of TSC2 phosphorylation, thereby suppressing mTORC1/S6K/S6RP pathway activity. In various PDX models, CDK4/6 inhibitors sensitize HER2-targeted therapy and significantly delay tumor recurrence in HER2-positive breast cancer models.

Currently, researchers at Peking University Cancer Hospital have initiated a Phase I trial (NCT03480256) evaluating the combination of SHR6390 with pyrotinib for the treatment of HER2-positive advanced gastric cancer. Additionally, several studies are underway nationally and internationally focusing on CDK4/6 inhibitors in combination with anti-HER2 small

molecule inhibitors or antibodies for hormone receptor-positive, HER2-positive advanced breast cancer. A single-arm open-label Phase Ib/II clinical trial (NCT03054363) evaluating the safety and efficacy of tucatinib in combination with palbociclib and letrozole for the treatment of hormone receptor-positive and HER2-positive metastatic breast cancer. The NA-PHER2 study (NCT02530424), a Phase II trial investigating the combination of palbociclib with trastuzumab, pertuzumab, and fluorouracil/doxorubicin/cyclophosphamide as neoadjuvant therapy for ER-positive HER2-positive invasive breast cancer. The PATRICIA study (NCT02448420), a Phase II trial evaluating palbociclib in combination with trastuzumab, with or without letrozole, for the treatment of postmenopausal locally advanced or metastatic ER-positive HER2-positive breast cancer. The MonarcHER study (NCT02675231), a Phase II trial comparing abemaciclib in combination with trastuzumab and fluorouracil/doxorubicin/cyclophosphamide, and abemaciclib in combination with trastuzumab, versus trastuzumab in combination with standard chemotherapy for the treatment of ER-positive HER2-positive advanced breast cancer.

Based on the aforementioned clinical trials and theoretical foundations, CDK4/6 inhibitors can enhance the efficacy of endocrine therapy and augment the anti-tumor effects of HER2 targeting. Fudan University Affiliated Cancer Hospital has conducted a Phase Ib clinical study on the combination of letrozole, pyrotinib, and the CDK4/6 inhibitor SHR6390 for the treatment of hormone receptor-positive, HER2-positive advanced breast cancer. The tolerance data and preliminary efficacy data from the Phase Ib study are as follows:

1) Combination of letrozole 2.5 mg/day, pyrotinib 400 mg/day, and SHR6390 125 mg/day: 5 patients enrolled, 2 experienced dose-limiting toxicity (grade III oral mucositis), with a best response rate (ORR) of 60%.

2) Combination of letrozole 2.5 mg/day, pyrotinib 400 mg/day, and SHR6390 100 mg/day: 6 patients enrolled, 1 experienced dose-limiting toxicity (grade III oral mucositis), with a best response rate (ORR) of 50%.

3) Combination of letrozole 2.5 mg/day, pyrotinib 320 mg/day, and SHR6390 125 mg/day: 3 patients enrolled, no dose-limiting toxicity reported, with 4 patients achieving partial response and a best response rate (ORR) of 75%.

Based on the safety and tolerability data from the Ib phase study, along with preliminary efficacy data, the recommended dosing for the II phase study is as follows: letrozole 2.5 mg/d, pyrotinib 320 mg/d, and SHR6390 125 mg/d. Subsequently, a multicenter II phase clinical study will be conducted to investigate the combination of letrozole, pyrotinib, and the CDK4/6 inhibitor SHR6390 in the treatment of estrogen receptor-positive and HER2-positive

advanced breast cancer.

1.1. Investigational Product

a) Name：Letrozole tablets

Letrozole tablets, a medication marketed by Jiangsu Hengrui Medicine Co., Ltd. For detailed information on its physicochemical properties and biological actions, it is advisable to refer directly to the drug's package insert.

b) Name：Pyrotinib Malate

Hanyu Pinyin：Biluotini

English name：Pyrotinib

Chinese chemical name [redacted]

English chemical name [redacted]

c) Name：None

Compound code: SHR6390

Hanyu Pinyin：SHR6390 Pian

English name：SHR6390 Tablets

Chinese chemical name [redacted]

Chemical structure[redacted]

1.2. The pharmacological type and mechanism for SHR6390

SHR6390 is a CDK4/6 kinase inhibitor and a [redacted] new drug developed by Jiangsu Hengrui Pharmaceuticals. Preclinical data indicate that SHR6390 selectively inhibits the activity of CDK4/6 kinases. This prevents the complex formed by these kinases with Cyclin D from phosphorylating the downstream Rb protein, thereby blocking the transition of cells from the G1 to the S phase and exerting an anti-proliferative and anti-tumor effect.

1.3. SHR6390 Pharmacodynamics

[redacted]

1.4. SHR6390 Toxicology

[redacted]

1.5. SHR6390 Pharmacokinetics

[redacted]

1.6. In Vivo Antitumor Activity of Pyrotinib and SHR6390 in ER+, HER2+ Breast Cancer Models

[redacted]

2. Clinical Studies

2.1. Pyrotinib Malate

Pyrotinib malate tablets, independently developed by Jiangsu Hengrui Pharmaceuticals, are an innovative drug and an irreversible dual-target tyrosine kinase inhibitor targeting EGFR and HER2. They are used for HER2-positive advanced or metastatic breast cancer. Phase II clinical trial data indicate that, for patients who failed treatment with anthracyclines and taxanes and received no more than two lines of chemotherapy after recurrence/metastasis, the combination of pyrotinib malate tablets with capecitabine (referred to as the "pyrotinib group") achieved an objective response rate of 78.5%, compared to 57.1% in the group combining lapatinib tablets with capecitabine (referred to as the "lapatinib group"). The median progression-free survival (PFS) was 18.1 months for the pyrotinib group and 7.0 months for the lapatinib group. The median PFS of the pyrotinib group was significantly longer than that of the lapatinib group, with statistical significance (P<0.0001). The risk of disease progression or death was reduced by 63.7% (HR=0.363) in the pyrotinib group compared to the lapatinib group, while maintaining good tolerability. Based on the efficacy and safety data obtained from the current Phase II clinical trials, the China National Medical Products Administration agreed to accept Hengrui Medicine's application for conditional approval of pyrotinib malate tablets. Following the publication of the large-scale Phase III studies PHENIX and PHOEBE, pyrotinib received full approval from the National Medical Products Administration in 2020 as a fully approved innovative anti-cancer drug.

2.2. SHR6390

SHR6390 has currently initiated Phase I clinical trials for advanced solid tumors, Phase I for advanced melanoma, and Phase Ib/II and Phase III clinical trials for advanced breast cancer.

SHR6390-I-101 is an ongoing Phase I clinical study in China that evaluates the tolerability and pharmacokinetics of SHR6390 in patients with advanced solid tumors. The primary objective of the study is to observe the dose-limiting toxicity (DLT) and maximum tolerated dose (MTD) of SHR6390 tablets after single and multiple oral administrations in patients

with advanced solid tumors. The secondary objectives include observing the pharmacokinetic characteristics of SHR6390 tablets in these patients, as well as assessing their safety, tolerability, and the preliminary efficacy of SHR6390 tablets in the treatment of advanced solid tumors.[redacted]

2.3. Marketed CDK4/6 inhibitors

The PALOMA-2 study, a Phase III double-blind, randomized controlled trial, assessed the efficacy of palbociclib combined with letrozole as a first-line treatment for advanced ER+/HER2- negative breast cancer. The study enrolled 666 patients, and the primary endpoint indicated that the median progression-free survival (PFS) reached 24.8 months with the combination, compared to 14.5 months for the letrozole alone group, marking a significant advancement in achieving over two years of PFS for first-line treatment in advanced breast cancer. The most common grade 3 or 4 adverse events included neutropenia (occurrence rate of 66.4% in the Palbociclib-letrozole group vs 1.4% in the placebo-letrozole group), leukopenia (24.8% vs. 0%), anemia (5.4% vs. 1.8%), and fatigue (1.8% vs. 0.5%).

Another Phase III randomized controlled trial investigated the efficacy and safety of ribociclib combined with letrozole versus placebo combined with letrozole in first-line treatment for HR-positive, HER2-negative postmenopausal patients with recurrent or metastatic breast cancer, including 668 patients. The ribociclib combination demonstrated significantly longer PFS compared to the placebo group (HR, 0.56; 95% CI, 0.43-0.72). After 18 months of follow-up, the ribociclib group had a PFS rate of 63.0% (95% CI, 54.6-70.3) versus 42.2% (95% CI, 34.8-49.5) in the placebo group. The overall response rates were 52.7% and 37.1%, respectively (P <0.001). Common grade 3 or 4 adverse events reported by more than 10% of patients included neutropenia (59.3% in the ribociclib group vs 0.9% in the placebo group) and leukopenia (21.0% & 0.6%); the rates of discontinuation due to adverse events were 7.5% and 2.1%, respectively.

The MONARCH-3 study, a double-blind, randomized Phase III trial, involved 493 patients with advanced breast cancer, assessing abemaciclib or placebo combined with a non-steroidal aromatase inhibitor for first-line treatment in HR-positive, HER2-negative postmenopausal advanced breast cancer. The median PFS was significantly extended in the abemaciclib group (HR=0.54; 95% CI, 0.41-0.72; P = .000021; median not reached for the abemaciclib group vs 14.7 months for the placebo group). The objective response rate was 59% in the abemaciclib group vs 44% in the placebo group (P = 0.004). In the abemaciclib group, the most common adverse reaction was diarrhea (81.3%, with grade 1 accounting for 44.6%). The most

common grade 3 or 4 adverse events in the abemaciclib compared to the placebo group were neutropenia (21.1% vs 1.2%), diarrhea (9.5% vs 1.2%), and leukopenia (7.6% vs 0.6%).

3. Study Objectives and Study Endpoints

3.1. Primary Study Objective

Phase I (Ib stage):

Determine the safety and tolerability of the combination of a non-steroidal aromatase inhibitor (letrozole), pyrotinib maleate tablets, and the CDK4/6 inhibitor SHR6390 in the treatment of hormone receptor-positive, HER2-positive advanced breast cancer. Based on preliminary efficacy data, establish the recommended Phase II dosage for this combination therapy.

Phase II:

To evaluate the efficacy and safety of the combination of letrozole, pyrotinib, and the CDK4/6 inhibitor SHR6390 in the treatment of estrogen receptor-positive and HER2-positive advanced breast cancer. The study also aims to further explore the value of circulating tumor DNA (ctDNA) as a biomarker, as well as the clinical utility of FDG-PET and HER2-PET in assessing anti-tumor efficacy. [redacted]

3.2. Primary Endpoint

The primary endpoints of this trial include:

Phase I:

- Determination of dose-limiting toxicity (DLT) and maximum tolerated dose (MTD) of SHR6390 within the combination regimen to establish the recommended dosing regimen for the Phase II clinical study.

- Assessment of the incidence and severity of adverse events (AEs) and serious adverse events (SAEs) across different dose groups.

Phase II:

The objective response rate (ORR) of the recommended dosing regimen for the Phase II clinical study, evaluated based on RECIST 1.1 criteria.

3.3. Secondary Endpoints

Phase I

- Objective Response Rate (ORR), based on RECIST 1.1 criteria.

Phase II

- Incidence and severity of adverse events (AEs) and serious adverse events (SAEs).

Common Secondary Endpoints for Phase I/II Trial Include:

•Progression-free survival (PFS)

•Disease Control Rate (DCR, The proportion of subjects with a best overall response of complete response, partial response, or stable disease).

•Clinical benefit rate (CBR, proportion of complete response, partial response, or stable disease for at least 24 weeks)

•Duration of response (DOR)

•Exploratory analysis of the relationship between molecular markers and efficacy

4. Investigational Drugs

4.1. Name and Source

The investigational drugs for this project—letrozole, pyrotinib, and SHR6390 tablets—are all manufactured and provided by Jiangsu Hengrui Pharmaceuticals Co., Ltd.

4.2. Dosage Form and Specifications

Letrozole tablets: 2.5 mg;

Pyrotinib tablets: 160 mg, 80 mg;

SHR6390 tablets: 25 mg, 125 mg;

4.3. Storage

Storage Conditions: Store sealed at below 25°C.

Shelf Life: Tentatively 24 months.

4.4. Administration

- Non-steroidal aromatase inhibitor: Letrozole: Administer orally, 2.5 mg daily, on an empty stomach. Administer continuously for 28 days as one cycle.
- Pyrotinib: Administer orally, 320 mg daily, within 30 minutes after breakfast. Administer continuously for 28 days as one cycle.
- SHR6390: Administer orally, 125 mg daily, on an empty stomach (ensure fasting for at least 1 hour before and 1 hour after taking the medication). The medication is taken for 28 days as one cycle, with continuous dosing for the first three weeks (Day 1 to Day 21) and no dosing during the fourth week (Day 22 to Day 28).

4.5. Management, Distribution, and Retrieval

The management, distribution, and retrieval of drugs for clinical use in this trial are handled by designated personnel. Researchers must ensure that all investigational drugs are used only for participants in this clinical trial, and the dosage and administration should comply with the trial protocol. Any remaining drugs must be returned and must not be transferred to anyone not participating in the clinical trial. During drug distribution, a drug receipt form must be signed by two people, in duplicate. At the end of the study, any remaining drugs and empty boxes are to be collected, and a drug return form must be signed. Each instance of drug distribution and retrieval must be promptly recorded on a specific record form.

5. Overall Study Design

This study is a single-arm, open-label, dose-escalation Phase Ib clinical trial and a single-arm, open-label, multi-center Phase II clinical trial. In accordance with the "Regulations for Drug Registration," "Good Clinical Practice," and "Guidelines for Clinical Pharmacokinetics Studies of New Drugs (Chemical Drugs)," This trial aims to evaluate the efficacy and safety of the combination of letrozole, pyrotinib, and the CDK4/6 inhibitor SHR6390 in patients with estrogen receptor-positive, HER2-positive advanced breast cancer who have not responded to standard treatments or lack standard treatment options.

6. Study Design

6.1. Dosage and Administration

In patients with hormone receptor-positive, HER2-positive advanced breast cancer who have either not responded to standard treatments or lack standard treatment options, a dose-escalation study of the combination therapy will be conducted to observe tolerability and preliminarily assess efficacy. Once the last subject in each dose group completes 28 consecutive days of dosing and the DLT observation results for that dose group meet the criteria for dose escalation, the next higher dose group may begin the dose-escalation trial.

Phase I (Ib stage):

In this study, letrozole is administered at 2.5 mg/day, pyrotinib at either 400 mg/day or 320 mg/day, and SHR6390 is organized into three dose groups.

Dose groups	SHR6390	pyrotinib	Patients
A	125 mg/d	400 mg/d	3~6
B	150 mg/d	400 mg/d	3~6
C(backup group)	100 mg/d	400 mg/d	3~6
D(backup group)	125 mg/d	320 mg/d	3~6
E(backup group)	150 mg/d	320 mg/d	3~6
F(backup group)	100 mg/d	320 mg/d	3~6

The trial begins with Group A and follows a 3+3 dose-escalation design. The escalation sequence proceeds from Group A to Group B. If ≥2 cases of DLT are observed in Group A, Groups C and D will be initiated. If Group C remains intolerable, backup Group F will be initiated. If Group D is tolerable, escalation will proceed to Group E; if Group D is intolerable, then Group F will be initiated. If Group F remains intolerable, the research team will analyze the trial data to determine whether to terminate the study. DLT assessment will be conducted at the end of one cycle (28 days) for each dose group.

For specific dosing instructions of the three drugs, refer to Section 4.4. The initial two cycles are designated as the core study phase. Subjects evaluated as CR, PR, or SD at the end of Cycle 2 may continue on the assigned combination regimen until disease progression, intolerable toxicity, or voluntary withdrawal.

Phase II:

Based on the recommended Phase II dosing established in Phase I, the expansion study in Phase II will administer pyrotinib at 320 mg/day, SHR6390 at 125 mg/day, and letrozole at 2.5 mg/day. Treatment will continue until disease progression, intolerable toxicity, or voluntary withdrawal by the patient.

6.2. Dose-limiting toxicity (DLT)

In this trial, DLT is defined as any of the following drug-related or possibly drug-related adverse events occurring within Cycle 1 (based on CTC-AE v4.0.3 criteria):

1. Hematologic Toxicity:

- Grade 4 neutropenia lasting ≥5 days;

- Grade 4 thrombocytopenia or Grade 3 thrombocytopenia with clinically significant bleeding;

- Grade ≥3 neutropenia with fever (≥38.0°C lasting 1 hour or >38.3°C);

- Grade ≥4 anemia.

2. Non-Hematologic Toxicity:

Any Grade ≥3 non-hematologic toxicity, except for the following:

- Grade 3–4 nausea/vomiting and/or diarrhea and/or electrolyte imbalance that resolves to Grade ≤2 within 72 hours with optimal supportive treatment;

- Grade 3–4 elevations in alkaline phosphatase or gamma-glutamyl transferase that are clearly related to the tumor and unrelated to the drug.

6.3. Maximum tolerated dose (MTD)

During the dosing observation period in Cycle 1 (28 days), if ≥1/3 of the subjects in a given dose group experience DLT, the previous dose group is designated as the MTD. If dose escalation reaches Group B in the Phase I trial without ≥1/3 of subjects experiencing DLT, the research team will discuss whether to initiate the backup dose group C and determine the recommended dose for the Phase II trial.

6.4. Subject replacement

If a subject needs to withdraw from the study due to a non-DLT event during the DLT observation period, an additional subject will be included as a replacement.

If a subject experiences an unplanned treatment interruption exceeding 4 days during the DLT observation period, they will be considered to have received insufficient treatment intensity. In this case, even if no DLT is observed, an additional subject will be included as a replacement.

6.5. Planned Sample Size

Based on data analysis from the first subject in the Phase Ib and initial Phase II studies, the anticipated ORR for HER2 first-line treatment subjects in the Phase II study is 50%, while for second-line treatment subjects, it is 43%.

Using a historical control ORR of 28%, based on prior studies, the sample size for first-line subjects is calculated to be 35, assuming a significance level (α) of 0.05 and a power of 80%. Adjusting for a 10% loss to follow-up, the final sample size is 39 subjects. Historical control basis: In the EGF30008 study, the ORR for HER2+ HR+ MBC patients receiving first-line lapatinib combined with letrozole was 28%.

Using a historical control ORR of 19%, the sample size for second-line subjects is calculated to be 25 under the same parameters (α = 0.05, power = 80%). After accounting for a 10% loss to follow-up, the sample size is adjusted to 28 subjects. Historical control basis: In the ALTERNATIVE study, the ORR for HER2+ HR+ MBC patients receiving first- to second-line lapatinib combined with letrozole was 18.6%.

7. Sample Collection

In Phase Ib, plasma samples will be collected at 1 hour, 3 hours, and 24 hours after dosing on Day 21 of the first cycle.In Phase II, blood plasma samples are collected from 8-10 participants on day 21 of the first cycle just within 0.5 hours before and at 2 hours ±5 minutes, 4 hours ±10 minutes, 6 hours ±10 minutes, 12 hours ±10 minutes, and 24 hours ±0.5 hours after administration of pyrotinib. On day 22 of the 3rd, 5th, 8th, and 12th cycles, samples are taken within 0.5 hours before pyrotinib administration. The timing of dosing for the 3 days prior to PK blood collection is relatively fixed. Blood is drawn into two lithium

heparin anticoagulant tubes, each extracting 3 mL of venous blood. The tubes are gently inverted 3-5 times to ensure adequate mixing of blood and anticoagulant and are then left at room temperature. The actual blood collection times are accurately recorded and detailed in the blood collection log. Within one hour of collection, the samples are transferred to a centrifuge and centrifuged at 2000g for 10 minutes at room temperature (15° C - 32° C) to separate the plasma. The plasma is then aliquoted into two cryogenic tubes: one for testing and one as a backup. The testing tube should contain no less than 0.5 ml of plasma, with the remaining plasma transferred to the backup tube. Care should be taken not to aspirate the bottom layer of blood cells, and appropriate labels should be affixed. The tubes are stored at -80±10° C in a low-temperature freezer for testing.The cryogenic tubes must be kept in the low-temperature freezer until shipment, and the freezer temperature should be recorded daily. When shipping samples, the testing tubes are sent first while the backup tubes remain stored at -80±10° C until the end of the experiment when they are shipped collectively.

Baseline blood samples are also collected at the end of cycle 2 and before disease progression or the start of new anti-tumor treatment. This involves [redacted] along with ctDNA blood samples. Existing paraffin-embedded tumor tissue samples or ≥10 unstained slides are collected, with an effort made to obtain biopsy samples from metastatic sites.

8. Participants

8.1. Subjects and Sample Size

From an ethical and scientific standpoint, subjects must have received no more than one prior systemic treatment regimen containing trastuzumab for recurrent or metastatic breast cancer. Subjects should have no history of HER2 TKI treatment or, if previously treated, should have no evidence of treatment failure. Prior endocrine therapy must not demonstrate aromatase inhibitor resistance. Informed consent must be signed before entering the clinical trial.

Phase I has already enrolled 15 subjects. Based on the results of Phase I, Phase II is expected to enroll 39 subjects for first-line HER2 treatment and 28 subjects for second-line HER2 treatment.

8.2. Inclusion Criteria

1. Participants voluntarily join the study, sign an informed consent form, and demonstrate good compliance.

2. Female patients aged 18 to 75 years (inclusive).
3. Patients with histologically confirmed recurrent/metastatic breast cancer, ER-positive and HER2-positive:

- HER2 positivity defined as 3+ by standard immunohistochemistry (IHC) and/or positive by in situ hybridization (ISH).
- ER positivity defined as ≥1% ER-expressing cells.
- Local recurrence must be confirmed by the researcher as inoperable.

4. At least one measurable extracranial lesion according to RECIST 1.1 criteria.
5. Postmenopausal women, or women who have undergone bilateral oophorectomy or are receiving ovarian function suppression.
6. Previous antitumor treatment guidelines:

- Maximum of one prior systemic therapy regimen for recurrent/metastatic breast cancer including anti-HER2 ADC:

  i. If not previously treated with a trastuzumab regimen in the advanced stage, or if recurrence occurs more than one year after adjuvant trastuzumab treatment, subsequent therapy is considered first-line anti-HER2 treatment.

  ii. If first-line treatment with a trastuzumab regimen fails, or if recurrence occurs during adjuvant trastuzumab treatment or within one year after its completion, subsequent therapy is considered second-line anti-HER2 treatment.

- No prior anti-HER2 TKI therapy or no proof of failure with such therapy.
- No prior evidence of resistance to aromatase inhibitors in endocrine therapy (resistance defined as recurrence during adjuvant aromatase inhibitor therapy or within one year after its completion, or disease progression after receiving aromatase inhibitor therapy during the recurrent/metastatic phase).

7. ECOG performance status of 0 – 1.
8. Expected survival of ≥12 weeks.
9. Adequate organ function (no use of any blood components and growth factors within 2 weeks prior to enrollment):

- Absolute neutrophil count ≥1.5×10^9/L;
- Platelets ≥90×10^9/L;
- Hemoglobin ≥90g/L;
- Total bilirubin ≤1.5 times upper limit of normal (ULN);
- ALT and AST ≤2.5 times ULN;

- Urea/Blood Urea Nitrogen (BUN) and creatinine (Cr) ≤1.5×ULN;
- Left ventricular ejection fraction (LVEF) ≥50%;
- Fridericia-corrected QT interval (QTcF) < 470 ms;
- INR ≤1.5×ULN, APTT ≤1.5×ULN.

8.3. Exclusion Criteria

Patients with any of the following criteria are not eligible to participate in this study:

1. Subjects with untreated central nervous system metastases.
2. Patients with a history of systemic or definitive treatment for brain or meningeal metastases (radiotherapy or surgery) may be included if imaging confirms stable disease for at least 4 weeks, they have discontinued systemic steroids therapy for more than 4 weeks, and they show no clinical symptoms.
3. Prior treatment with any CDK4/6 inhibitor.
4. Presence of symptomatic ascites, pleural effusion, or pericardial effusion requiring drainage at baseline, or who have undergone serous cavity fluid drainage within 4 weeks prior to first drug administration.
5. Inability to swallow, intestinal obstruction, or other factors affecting drug intake and absorption.
6. Received chemotherapy, molecular targeted therapy, or other systemic treatments including clinical trial drugs within 4 weeks prior to enrollment; received endocrine therapy within 2 weeks prior to enrollment.
7. History of other malignancies within the past 5 years or concurrent malignancy, except for cured basal cell carcinoma of the skin and cervical carcinoma in situ.
8. Major surgical procedures or significant trauma within 4 weeks prior to the first drug administration, or anticipated need for major surgery during the study.
9. Pregnant or breastfeeding women, women of childbearing potential with a positive baseline pregnancy test, or unwilling to use effective contraception.
10. Known hypersensitivity to any component of the study drugs.
11. Active HBV or HCV infection; stable hepatitis B (HBV viral copy number not exceeding the upper limit of normal) treated with medication and cured hepatitis C (HCV viral copy number below the detection limit of the assay) are excluded.
12. History of immunodeficiency, including HIV positivity, or any other acquired or

congenital immunodeficiency diseases, or history of organ transplantation.

13. History of any cardiac disease, including: (1) angina; (2) arrhythmias requiring medication or clinically significant; (3) myocardial infarction; (4) heart failure; (5) any other heart disease deemed by the investigator as unsuitable for participation in the trial; severe cardiac or renal function abnormalities of grade ≥II found during the screening period.

14. According to the investigator's judgement, severe concomitant diseases that pose a risk to patient safety or affect the completion of the study (e.g., severe hypertension, diabetes, thyroid disorders).

15. History of definite neurologic or psychiatric disorders, including epilepsy or dementia.

16. Severe infection within 4 weeks prior to the first drug administration (e.g., requiring intravenous administration of antibiotics, antifungals, or antivirals according to clinical guidelines), or unexplained fever >38.3°C during the screening period or prior to the first administration.

8.4. Participant Identification

In this trial, all participants who have signed the informed consent form will receive a unique participant code, such as II-01-01. The coding rule is as follows: the first two digits represent the trial phase, such as II; the middle two digits are the center number, sequentially assigned as 01, 02, 03, etc.; the last two digits correspond to the order in which participants are screened at that center, continuing as 01, 02, 03, and so forth.

8.5. Dropout Criteria

All patients who have signed the written informed consent form and passed the screening to enter the trial have the right to withdraw from the clinical trial at any time. Regardless of the reason or timing of withdrawal, participants who have not completed the first cycle of multiple dosing and cannot undergo a safety evaluation will be considered as dropout cases.

8.6. Termination Criteria for Participant Treatment

If any of the following conditions occur, the participant must withdraw/terminate treatment:

1. The participant withdraws informed consent and requests to exit.
2. Imaging examination shows disease progression.
3. Inability to tolerate toxicity.

4. Serious protocol violation, as assessed by the investigator warranting treatment termination.

5. Participant loss to follow-up or occurrence of a pregnancy event.

6. Other circumstances deemed necessary for participant withdrawal from the study by the investigator.

8.7. Study Termination Criteria

Study termination criteria include, but are not limited to:

1. Discovery of unexpected, significant, or unacceptable risks to participants;

2. The investigational drug/therapy is ineffective, or continuation of the trial is deemed futile;

3. The investigator decides to terminate the study due to reasons such as severe lag in participant enrollment or significant protocol violation

9. Dose Adjustment and concomitant medication

9.1. DLT Observation Period

During the DLT observation period in Phase Ib, non-DLT adverse events generally should not receive medical intervention to allow for observation of potential adverse reactions from the investigational drug, including their severity and reversibility. However, if a DLT as defined in the protocol occurs, the investigational drug must be immediately discontinued, and appropriate medical treatment should be initiated. The medication used for management should be documented in the CRF.

9.2. Continuous Dosing in Cycle 2 and Subsequent Cycles

Dose Adjustment Criteria (to be applied for all dosing cycles in the Phase II study)

After the occurrence of toxic reactions, physicians can make judgments based on the situation and provide appropriate management. The specific principles of management are as follows:

Table 6

Adverse Events	Severity			
	Grade I	Grade II	Grade III	Grade IV

**Hematologic Toxicity**	Maintain dose	Maintain dose	Pause treatment, symptomatic management, resume treatment if reduced to Grade I or below, adjust dose as per investigator's judgment for this and subsequent cycles	Pause treatment, symptomatic management, dose reduction required if reduced to Grade I or below
**Non-Hematologic Toxicity**	Maintain dose	Maintain or pause treatment, symptomatic management, if reduced to Grade I or below, dose adjustment optional based on investigator's	Pause treatment, symptomatic management, dose reduction required if reduced to Grade I or below	Permanently discontinue treatment, withdraw from study
**Febrile Neutropenia**	-	-	Pause treatment, symptomatic management, resume treatment if reduced to Grade I or below, dose	Permanently discontinue treatment, withdraw from study

When definite toxicity related to the study drug occurs, the investigator will handle it based on clinical manifestations. Administration may resume once recovery to ≤ Grade I is achieved (or if the investigator deems the adverse event tolerable at ≤ Grade II without significant safety risks). If the same adverse event recurs, the investigator will decide whether to pause and adjust the dosage, or require the participant to withdraw from the study; the investigator's priority will be to maximize participant safety.

If toxicity does not resolve within 2 weeks after pausing medication, the participant should, in principle, withdraw from the study. Any pauses in administration should be included in the dosing cycle.

9.3. Prohibited Medications

During the treatment period, the use of other antitumor drugs and tumor treatment-related adjuvant medications should be discontinued. This includes antitumor traditional Chinese medicines, antitumor hormonal therapy drugs, immunotherapies, or other antitumor therapeutic drugs.

9.4. Permitted Medications

If participants experience adverse reactions, they should be closely monitored, and symptomatic treatment should be administered as necessary. The treatment medications used must be recorded and explained on the Case Report Form (CRF). Details such as the timing of use, name of the medication, and the method and dosage should be documented.

10. Study Procedures

Before starting the study, patients must read and sign the currently Ethics Committee (EC)-approved informed consent form. All study procedures must be conducted within the time windows specified in the study schedule.

10.1. Screening period

Before starting treatment with the study drug, the following screening steps should be completed within 28 days:

- Signing of Informed Consent Form: Patients must read and sign the informed consent form approved by the Ethics Committee.

- Medical History and Demographic Data Collection: Includes collecting patient's ID, address, contact details, a detailed inquiry of tumor history/other diseases history, pathology results, ER/PR/HER2 testing reports, history of tumor surgery, chemotherapy, radiation therapy, and other disease treatments; non-breast cancer tumor history.

- Tests for Hepatitis B, Hepatitis C, and HIV: Hepatitis B panel and, if abnormal, HBV DNA replication testing; Hepatitis C virus antibody (anti-HCV), and HIV antibody tests.

- Imaging Studies: Screening imaging includes enhanced CT or MRI of the chest, abdomen, and brain, and other suspected areas like the neck or pelvis. Tumor baseline assessment can be extended to within four weeks before the first administration, CT/MRI scans obtained

before signing informed consent can be used for tumor evaluation if they meet the requirements; bone scans are necessary if clinical suspicion of bone metastases exists (FDG-PET, HER2-PET are performed at the lead unit and participating units with capabilities).

-[redacted]

- Biomarker Collection: [redacted] along with ctDNA samples; collect existing paraffin-embedded tumor tissue samples or ≥10 unstained slides, preferably obtaining biopsy specimens from metastatic sites.

- Echocardiogram: Monitor changes in LVEF value, additional unplanned checks may be performed if LVEF falls below 50% and drops by ≥10% from baseline, or if symptoms like chest pain or palpitations occur.

- Evaluation of Current Medications and Treatments: Assess the medications and treatments currently being used by the patient.

- Collection of Adverse Events: Start collecting adverse events from the time of informed consent.

The following screening steps should be completed within 7 days before starting treatment with the study drug:

- Vital Signs Check: Temperature, respiration, pulse, blood pressure; smoking and coffee consumption are prohibited 30 minutes before measurement, and at least 10 minutes of rest in a seated position with the elbow at heart level is required for blood pressure measurement.

- Physical Examination: Examination of major body systems (head, face, skin system, lymph nodes, eyes, ENT, oral cavity, respiratory system, cardiovascular system, abdomen, reproductive urinary system, musculoskeletal, nervous system, and mental state).

- ECOG Performance Status.

- Complete Blood Count: Hemoglobin, red blood cells, white blood cells, neutrophils, lymphocytes, and platelet count.

- Urinalysis: Urine protein, urine sugar, urine occult blood (urine red cells, white cells). If the

semi-quantitative method shows protein 2+, a 24-hour urine protein quantitative test is performed.

- Stool Routine: Including fecal occult blood.

- Blood Biochemistry: Total bilirubin, conjugated bilirubin, ALT, AST, ALP, γ-GT, LDH, total protein, albumin, urea/urea nitrogen, creatinine, uric acid, fasting blood glucose, triglycerides, cholesterol, potassium, sodium, chloride, calcium, phosphorus, magnesium; cardiac enzyme spectrum may be added if necessary.

- Pregnancy Test: Serum pregnancy testing within one week before the first medication use in women of childbearing age.

- 12-Lead ECG: If clinically significant abnormalities are found on the ECG, the investigator may confirm it again if necessary.

10.2. Trial Period

During the study, the following tasks should be completed within the specified timeframes indicated in the trial schedule (note: the time window from the first cycle is ±3 days):

- ECOG Score, Vital Signs, Physical Examination, 12-Lead ECG: Day 15 and 28 of Cycle 1, Day 28 of Cycle 2 (can be either C1D28 or C2D1, same for subsequent cycles), thereafter on Day 28 of each cycle.
- Complete Blood Count, Blood Biochemistry: Day 15 and 28 of Cycle 1, Day 28 of Cycle 2 (can be either C1D28 or C2D1, same for subsequent cycles), thereafter on Day 28 of each cycle (if the participant is from out of town, a weekly complete blood count can be performed at a local hospital from Cycle 2 onwards and the results communicated to the researcher, who will decide if further investigation at the study center is necessary).
- Urinalysis, Stool Routine: Every three cycles, replaced by screening tests in Cycle 1, Day 1 of Cycle 2 (can be either C1D28 or C2D1, same for subsequent cycles), thereafter on Day 28 of each cycle.
- Echocardiogram: Every three cycles, replaced by screening tests in Cycle 1, Day 1 of Cycle 4 (can be either C3D28 or C4D1, same for subsequent cycles), thereafter every three cycles on Day 28; at the end of treatment or upon participant withdrawal (if not done in the previous four weeks).
- Biomarker Collection: Baseline, end of Cycle 2, before disease progression or starting a

new anticancer treatment, collecting [redacted] along with ctDNA samples.

- Imaging Studies: The allowed imaging study window is ±7 days, and during treatment, imaging should be conducted under the same conditions as baseline (slice thickness, contrast use, etc.). Specific assessment times include:
- Every six cycles before medication, initial assessment at the end of Cycle 2, then every two cycles for six cycles, followed by every three cycles until disease progression, intolerable toxicity, or the start of new cancer treatment.
- For initial assessments showing CR or PR, a confirmation is recommended 4-6 weeks later; subsequent tumor evaluations should not alter the fixed two-cycle examination timeline.
- Unscheduled imaging studies may be performed if disease progression is suspected (e.g., worsening symptoms).
- Aside from confirmed disease progression through imaging, participants ending trial treatment for other reasons should undergo imaging evaluation at the end of treatment if not done in the prior four weeks, and follow-up according to the protocol-specified frequency continues after trial end until documented disease progression or the start of a new cancer treatment.
- [redacted]
- Adverse Events: From signing the informed consent form until just before starting the medication, only serious adverse events are recorded, up to 28 days after the last medication, and follow-up continues until adverse events disappear, alleviate to baseline level or ≤ Grade 1, or stabilize.
- Recording Adverse Reactions: From the first day of study treatment, at least 28 days after the last treatment, or until all serious or drug-related toxicities recover to ≤ Grade 1 per NCI-CTC AE 4.0.3 standards. Additionally, various clinical manifestations during medication are observed and recorded.
- Recording Concomitant Medication or Treatment: From 28 days before study treatment until 28 days after the last treatment.

Note: Researchers may increase the frequency of participant visits (e.g., weekly) and the content of examinations as needed to maximize participant safety.

10.3.Study termination/withdrawal

Study termination: 24 months after the last subject is enrolled, or when the researcher deems it necessary to end the trial prematurely.

10.4. Follow-up after treatment completion

- Disease Progression: In addition to imaging-confirmed disease progression, subjects who end trial treatment for other reasons must undergo imaging evaluation at the end of treatment if no imaging has been done within the four weeks prior to the trial's conclusion. Furthermore, after the trial ends, continue to follow the tumor response according to the schedule specified in the protocol until documented disease progression or the start of a new cancer treatment.
- Survival Follow-Up: After the end of the trial treatment, survival status and subsequent anti-cancer treatment information can be collected every three months through clinical visits or telephone follow-ups until death.

10.4.1. Criteria for Severity of Adverse Events

Refer to version 4.0.3 of the NCI-CTC AE for grading standards of drug adverse reactions. For adverse reactions not listed in the table, refer to the following criteria:

- Grade I: Mild, no clinical symptoms or only minor clinical symptoms; only clinical or laboratory findings abnormal; no treatment required.
- Grade II: Moderate, requires minimal, local, or non-invasive treatment; activities of daily living (ADL) using tools are restricted, which includes activities like cooking, shopping, making phone calls, handling money, etc., consistent with age.
- Grade III: Severe or medically significant symptoms but not immediately life-threatening; causes hospitalization or prolongation of hospital stay; results in disability; self-care ADL is restricted. Self-care ADL includes bathing, dressing, undressing, eating, using the toilet, taking medication, etc., not bedridden.
- Grade IV: Life-threatening, requires urgent treatment.
- Grade V: Death due to adverse event.

11. Safety Assessment

11.1. Adverse Event (AE)

11.1.1. Adverse Event Definition

An adverse event is defined as any unfavorable medical occurrence in a clinical trial participant after the signing of the informed consent form. In this trial, any adverse medical events occurring from the start of the subject's treatment with the study drug until 28 days after the last use of the study drug are considered adverse events, regardless of causality with the study drug.

Researchers should record in detail any adverse events experienced by the participants, including: description of the adverse event and all related symptoms, time of occurrence, severity, duration, actions taken, and final outcome and resolution.

Concerned Adverse Events

After the occurrence of toxicity reactions, in principle, medication should not be stopped or the dosage reduced. Treatment may be given by the physician based on the situation, with specific treatment principles as follows:

- Hematological Support: Symptomatic treatment can be provided by researchers based on clinical presentations when hematological toxicity occurs. When hematological toxicity reaches Grade ≥III, medication should be paused until recovery to ≤Grade I, then resumed at the original dosage level. For Grade III or IV anemia, dosing may not be paused, and transfusion treatment may be carried out based on the researcher's judgment. Pausing medication does not affect the timing of tumor evaluation. If medication is paused for more than 14 days, the patient must withdraw from the study.
- Diarrhea: According to the "Medication Pause and Dose Adjustment" provisions in the trial protocol, symptomatic treatment should be given first, followed by close follow-up or observation (≤14 days). Clinical advice is to start oral montmorillonite powder (3 g/sachet, three times/day) or loperamide on the day of diarrhea. For unresolved Grade III diarrhea, medication should be paused; once the adverse event has recovered to within Grade I, resume the original dosage or reduce the dosage.
- Liver Function Abnormalities: Symptomatic treatment or observation (≤14 days) should be given by researchers based on the participant and adverse event conditions. If abnormalities persist after treatment or observation, close follow-up is required, and dosage adjustments and/or increased frequency of biochemical tests may be necessary. For participants with liver metastases, if ALT/AST levels exceed 1.5×UNL at enrollment, close monitoring of liver

function is required, and a comprehensive decision should be made on whether the participant is suitable for inclusion in the study.

- Other Risks: Since the study drug is experimental, using it alone or in combination with other drugs may carry unknown risks. All drugs have potential risks of allergic reactions, which could be life-threatening if not treated promptly. Any severe allergic reaction symptoms, such as difficulty breathing after activity or swelling of the face, lips, gums, tongue, or neck, should receive immediate medical help and contact the study physician. Other allergic reactions may include rash, hives, or blisters. It is very important for patients to promptly report all symptoms and side effects, regardless of whether they believe these are caused by the study drug.

Discomfort resulting from this experiment may not be limited to the events listed above, but we will closely follow up and actively manage to ensure the safety and well-being of the patients to the greatest extent possible, and all medications used in the treatment should be recorded on the CRF form.

11.1.2. Adverse Event-Drug Relationship Classification

Adverse events include all unexpected clinical manifestations occurring after the signing of the informed consent form, regardless of their relation to the study drug or even if the drug was administered. All adverse events must be reported clinically. Any discomfort reported by patients or any abnormal changes in objective laboratory tests during the treatment period must be accurately recorded, including the severity, duration, management, and outcome of the adverse events. Clinicians should also comprehensively determine the relationship between the adverse event and the study drug. The relationship between adverse events and the study drug is judged on a five-level scale: definitely related, possibly related, possibly unrelated, definitely unrelated, and indeterminate. The first two categories are considered adverse reactions, and the incidence of adverse reactions is statistically analyzed.

- Definitely related: The reaction occurs in a reasonable temporal sequence after medication use and is consistent with the known reaction type of the suspected drug; improvement after stopping the drug, and the reaction reappears upon re-administration.
- Possibly related: The reaction occurs in a reasonable temporal sequence after medication use and is consistent with the known reaction type of the suspected drug; the patient’ s clinical status or other treatment modalities might also produce such a reaction.

- Possibly unrelated: The reaction does not quite fit the reasonable temporal sequence after medication use, and the reaction type does not quite match the known reactions of the suspected drug; the patient’ s clinical status or other treatment modalities could possibly cause such a reaction.
- Definitely unrelated: The reaction does not fit the reasonable temporal sequence after medication use, and the reaction matches a known reaction type of a non-trial drug; the patient’ s clinical status or other treatment modalities might produce the reaction, which disappears with the improvement of the disease state or cessation of other treatments, and reappears with the re-use of other treatment methods.
- Indeterminate: The reaction occurs without a clear temporal relationship to medication use, is similar to the known reactions of the drug, and other concurrently used drugs might also cause the same reaction.

11.2.Serious Adverse Events (SAE)

11.2.1. Serious Adverse Events Definition

Serious Adverse Events (SAE) are medical events that occur during a clinical trial requiring hospitalization or prolongation of hospital stay, result in disability, impair work ability, are life-threatening or result in death, or lead to congenital anomalies. This includes the following unexpected medical events:

- Events leading to death;
- Life-threatening events (defined as situations where the subject is at risk of death at the time of the event);
- Events requiring hospitalization or prolonging hospital stay;
- Events that may cause permanent or serious disability/impairment;
- Congenital anomalies or birth defects;
- Other significant medical events.

11.2.2. Pregnancy

During the clinical trial, if a female participant becomes pregnant, she will be withdrawn from the trial， and the pregnancy should be reported as a serious adverse event.

11.2.3. Disease Progression

Disease progression (including symptoms and signs of progression) should not be reported as a serious adverse event (SAE). However, if a death occurs due to disease progression during

the trial or within the safety reporting period, it should be reported as an SAE. Hospitalization due to symptoms and signs of disease progression should not be reported as an SAE. If the ultimate outcome of the cancer is death within the trial or safety reporting period, the event leading to death must be reported as an SAE.

11.2.4. Undergoing Other Antineoplastic Treatments

Adverse event recording begins upon signing the informed consent and continues until 28 days after the last administration of the study drug. If a participant starts other antineoplastic treatments after these 28 days, there is no need to continue tracking non-fatal adverse events. However, if death occurs within the serious adverse event reporting period after the end of the study treatment, it must be reported, regardless of whether the patient is receiving other treatments.

11.2.5. Hospitalization

Adverse events that result in hospitalization or prolongation of hospital stay should be considered serious adverse events. Any initial admission to a medical facility, even if for less than 24 hours, meets this criterion.

Hospitalization does not include the following situations:

- Rehabilitation facilities
- Sanatoriums
- Routine emergency room admissions
- Same-day surgeries (such as outpatient/day/non-bedridden procedures) where the hospitalization or prolongation of stay is not related to a worsening of the adverse event. For example: Admission for an existing disease without the occurrence of a new adverse event or worsening of the existing disease (such as to investigate a laboratory abnormality that has persisted since before the trial);
- Hospitalizations for administrative reasons (such as annual routine physical examinations);
- Hospitalizations specified in the clinical trial protocol (such as procedures required by the trial protocol);
- Elective hospitalizations unrelated to a worsening of the adverse event (such as elective cosmetic surgery);
- Pre-scheduled treatments or surgeries should be documented in the entire trial protocol and/or participant's baseline information;
- Hospitalization solely for the use of blood products.

Diagnostic or therapeutic invasive (such as surgery) and non-invasive procedures should not be reported as adverse events. However, the condition leading to the procedure, if it meets the definition of an adverse event, should be reported. For example, acute appendicitis that occurs during the adverse event reporting period should be reported as an adverse event, and the resulting appendectomy should be recorded as a treatment for that adverse event.

12. Serious Adverse Event Reporting System

Reporting of serious adverse events should start from the moment a participant signs the informed consent form and continue until 28 calendar days (including the 28th day) after the last use of the study drug. During the trial, if a serious adverse event occurs, whether it is the initial report or a follow-up report, the investigator must immediately fill out the "Clinical Trial Serious Adverse Event (SAE) Report Form" for new drugs, sign it, and date it. Within 24 hours of becoming aware of the SAE, the investigator must notify the sponsor immediately and stop the participant's trial, taking measures to protect the participant. The investigator must track the SAE until it is resolved.

Serious adverse events should be meticulously documented, including symptoms, severity, time of occurrence, handling time, measures taken, follow-up timing and method, and outcome. If the investigator believes that a serious adverse event is not related to the study drug but potentially related to the study conditions (such as termination of the original treatment or complications during the trial), this relationship should be detailed in the narrative section of the medical record report form on the serious adverse event page.

13. SAE Reporting Procedures

Any serious adverse events that occur during the clinical study or within 28 days after discontinuation of the drug must be reported immediately in writing to the sponsor. After obtaining the primary efficacy endpoint results, any SAEs that occur during the period of observing secondary efficacy endpoint results must also be immediately reported to the sponsor. Additionally, the investigator must complete a Serious Adverse Event Report Form (SAE), detailing the time of occurrence, severity, relationship to the study drug, and measures taken, and sign the report.

14. Efficacy Evaluation

Efficacy evaluation is conducted using RECIST 1.1 criteria, including: (1) Evaluating the efficacy for each participant, which includes Complete Response (CR), Partial Response (PR), Stable Disease (SD), and Progressive Disease (PD). (2) Recording Progression-Free Survival (PFS), defined as the time from the start of study treatment to documented tumor progression or death from any cause, whichever comes first. Participants initially evaluated as CR or PR need confirmation after 4-6 weeks. Improvements or deteriorations in general condition are indicated by changes in ECOG scores before and after treatment.

15. Study Completion

The study concludes 24 months after the last participant is enrolled or earlier if the researcher deems it necessary to end the trial prematurely.

16. Safety Control Measures

1. Special Treatment

- Stop medication.

- Monitor vital signs: ECG, blood pressure, respiration, body temperature.

- Gastric lavage: 1%–2% sodium chloride solution or 1:5000 potassium permanganate solution.

- Laxative: 15–30 g sodium sulfate in 200 mL water for administration.

- Enema: 1% lukewarm soapy water (about 5000 mL) for high continuous cleansing.

2. Supportive Therapy

- Sedation and oxygen administration.

- Establish intravenous infusion access, open airways, and if necessary, provide tracheal intubation, external cardiac massage, and ventilatory support.

- Adequate fluid resuscitation to maintain circulating blood volume: Administer saline or glucose-sodium chloride solution intravenously, supplement with colloids according to the clinical condition to ensure osmotic pressure.

- Cardiotonics and vasopressors to maintain stable blood pressure and ensure blood supply to vital organs: Initially administer 20–60 mg corticosteroids with 50–250 mL of 5% glucose solution intravenously, followed by dopamine infusion to maintain stability.

- Diuretics according to urine output, administer appropriate doses of furosemide and sodium bicarbonate to alkalinize the urine.

- Maintain electrolyte and acid-base balance.

- Treat arrhythmias.

- Symptomatic treatment to maintain nitrogen balance.

3. Anti-Allergy

- Administer 10 mg of chlorpheniramine or 25–50 mg of promethazine; ensure the airway is clear while the patient is lying down and breathing oxygen.

- 0.1% adrenaline 0.1–0.2 mL added to 5% glucose for intravenous drip.

- Use corticosteroids such as dexamethasone.

4. Preventive Use of Gastric Acid Suppressants

5. Correct Respiratory and Circulatory Failure

- Provide oxygen or artificial respiration, administer 0.375 g of phentolamine and 3–6 mg of lobeline alternately by injection every 15–30 minutes as needed, and 1–2 times by IV if necessary, along with cardiotonic drugs like digoxin preparations.

17. Recommended Phase II Clinical Trial Dosing Regimen

Based on the tolerance, preliminary efficacy, and drug exposure results from the continuous dosing study, a comprehensive analysis has determined the optimal dosing regimen to be pyrotinib at 320 mg/day and SHR6390 at 125 mg/day in combination with endocrine therapy, progressing to a Phase II clinical trial.

18. Ethical Standards and Informed Consent

18.1. Ethical Standards

This clinical trial must adhere to the Helsinki Declaration (1996 version), the Good Clinical

Practice (GCP) guidelines issued by the CFDA, and relevant regulations.

18.2. Informed Consent

Participants must provide informed consent before receiving treatment in this trial, to protect their legal rights. Researchers must fully and comprehensively inform participants or their designated representatives about the study's purpose, the drug's effects, potential toxic side effects, and possible risks. It is essential that participants are made aware of their rights, the risks they are taking, and the benefits involved. The discussion is a crucial part of the informed consent process. If the participant and their legal representative are illiterate, the informed consent process must involve a witness. After the participant or their legal representative gives verbal consent, they should sign the informed consent form on the same day as the witness. The informed consent form should specify the version and the date it was created or modified.

19. Quality Assurance in Clinical Trials

To ensure that this trial is conducted strictly according to the clinical research protocol, throughout the entire clinical trial process, both clinical researchers and sponsors must strictly adhere to the Good Clinical Practice (GCP) guidelines. It is essential to ensure that trial procedures are standardized, trial data is accurate, and research conclusions are reliable.

20. Data Handling

20.1. Researcher Data Entry Requirements

- For all patients who have signed the informed consent form and are screened as eligible to participate in the trial, every item in the case report form must be recorded carefully and in detail, with no blank or missing items (use a line to indicate any space that should not be left empty).

- All data in the case report forms must be verified against the participants' medical records to ensure accuracy.

- The case report forms serve as original data; any corrections must be made by drawing a line through the error, annotating the corrected data next to it, and including the researcher's signature with the date.

- Copies of lab reports should be affixed in the designated section for lab reports in the case report forms.

- Any data that are significantly high or outside the clinically acceptable range must be verified, with necessary explanations provided by the researcher, as per the instructions for filling out the case report forms.

20.2. Data Traceability and CRF Completion

The original records consist of the research medical records for proper preservation. The case report forms (CRFs) are derived from these research medical records and must be completed by the researcher for each selected case.

20.3. Data Selection for Analysis

1. Full Analysis Set (FAS)

According to the intention-to-treat (ITT) principle, efficacy analysis is conducted on all enrolled cases who have received the drug at least once. For cases where the full treatment process was not observed, the last observation carried forward (LOCF) method is used to impute the final trial outcomes.

2. Per-protocol Set (PPS)

Includes all cases that adhered to the trial protocol, demonstrated good compliance, did not use any prohibited medication during the trial, and completed the case report forms as required. No imputation is made for missing data. Efficacy analysis of the trial drug is conducted on the PPS.

3. Safety Analysis Set (SAS)

Comprises all enrolled patients who have used the trial medication at least once and have post-medication safety records. This dataset is used for safety analysis.

20.4. Undergoing Other Antineoplastic Treatments

The results of this trial will primarily use descriptive statistical methods. For quantitative data, means, standard deviations, medians, maximum and minimum values will be listed. For count

data and ordinal data, frequencies (proportions), rates, and confidence intervals will be presented.

All statistical analyses will be conducted using SAS 9.2 statistical analysis software. All statistical tests will be two-sided, with a P-value of less than or equal to 0.05 considered statistically significant, and confidence intervals will be set at 95% confidence level.

1. Patient Baseline Characteristics

Calculate means, standard deviations, medians, maximums, and minimums for quantitative data such as age, height, and weight. Qualitative data like gender and ECOG scores will be listed with frequencies and percentages.

2. Tolerability Evaluation

Predominantly descriptive statistical analysis will be used, tabulating adverse events and reactions (where adverse reactions are defined as "adverse events related to the study drug as 'definitely related/very likely related/possibly related'") that occur in each dosage group in this trial. Laboratory test results will describe conditions that were normal before the trial but abnormal after treatment and the relationship of these changes to the study drug. Calculate means, standard deviations, medians, minimums, and maximums for vital signs (blood pressure, heart rate, body temperature, respiratory rate) and laboratory indicators before and after medication for each single-dose group, using paired t-tests for pre-post comparisons when necessary; trend tests will be performed for changes in vital signs and laboratory indicators between different dosage groups. For multiple-dose groups, comparisons will be made between each post-medication time point and before medication.

3. Efficacy Analysis

Tabular description of the clinical efficacy observed in this trial.

References

1. Siegel, R.L., Miller, K.D. & Jemal, A. Cancer statistics, 2018. *CA Cancer J Clin* **68**, 7-30 (2018).
2. Cardoso, F., *et al.* 1st International consensus guidelines for advanced breast cancer (ABC 1). *Breast* **21**, 242-252 (2012).
3. Goldhirsch, A., *et al.* Strategies for subtypes--dealing with the diversity of breast cancer: highlights of the St. Gallen International Expert Consensus on the Primary Therapy of Early Breast Cancer 2011. *Ann Oncol* **22**, 1736-1747 (2011).
4. Slamon, D.J., *et al.* Studies of the HER-2/neu proto-oncogene in human breast and ovarian cancer. *Science* **244**, 707-712 (1989).
5. Slamon, D.J., *et al.* Human breast cancer: correlation of relapse and survival with amplification of the HER-2/neu oncogene. *Science* **235**, 177-182 (1987).
6. Ponde, N., Brandao, M., El-Hachem, G., Werbrouck, E. & Piccart, M. Treatment of advanced HER2-positive breast cancer: 2018 and beyond. *Cancer Treat Rev* **67**, 10-20 (2018).
7. Velasco-Velazquez, M.A., *et al.* Examining the role of cyclin D1 in breast cancer. *Future Oncol* **7**, 753-765 (2011).
8. Casimiro, M.C., Velasco-Velazquez, M., Aguirre-Alvarado, C. & Pestell, R.G. Overview of cyclins D1 function in cancer and the CDK inhibitor landscape: past and present. *Expert Opin Investig Drugs* **23**, 295-304 (2014).
9. Finn, R.S., *et al.* Palbociclib and Letrozole in Advanced Breast Cancer. *N Engl J Med* **375**, 1925-1936 (2016).
10. Hortobagyi, G.N., *et al.* Ribociclib as First-Line Therapy for HR-Positive, Advanced Breast Cancer. *N Engl J Med* **375**, 1738-1748 (2016).
11. Goetz, M.P., *et al.* MONARCH 3: Abemaciclib As Initial Therapy for Advanced Breast Cancer. *J Clin Oncol* **35**, 3638-3646 (2017).
12. Goel, S., *et al.* CDK4/6 inhibition triggers anti-tumour immunity. *Nature* **548**, 471-475 (2017).
13. Goel, S., *et al.* Overcoming Therapeutic Resistance in HER2-Positive Breast Cancers with CDK4/6 Inhibitors. *Cancer Cell* **29**, 255-269 (2016).

Appendix 1: Performance Status Rating Scale (ECOG)

(Eastern Cooperative Oncology Group)

ECOG Score	Description
0	Fully active, able to carry on all pre-disease performance without restriction.
1	Restricted in physically strenuous activity but ambulatory and able to carry out work of a light or sedentary nature, e.g., light house work, office work.
2	Ambulatory and capable of all self-care but unable to carry out any work activities. Up and about more than 50% of waking hours.
3	Capable of only limited self-care, confined to bed or chair more than 50% of waking hours.
4	Completely disabled. Cannot carry on any self-care. Totally confined to bed or chair.
5	Dead.

Appendix 2: Creatinine clearance rate (CrCl)

Cockcroft-Gault Equation:

Serum Creatinine: in milligrams per deciliter (mg/dL):

$$\text{Males Creatinine Clearance (CrCl) (mL/min)} = \frac{(140-\text{age})\times\text{weight (kg)}^{a}}{72\times\text{serum creatinine (mg/dL)}}$$

$$\text{Females Creatinine Clearance (CrCl) (mL/min)} = \frac{0.85\times(140-\text{age})\times\text{weight (kg)}^{a}}{72\times\text{serum creatinine (mg/dL)}}$$

Serum Creatinine: in micromoles per liter (µmol/L):

$$\text{Males Creatinine Clearance (CrCl) (mL/min)} = \frac{(140-\text{age})\times\text{weight (kg)}^{a}}{0.81\times\text{serum creatinine }(\mu\text{mol/L})}$$

$$\text{Females Creatinine Clearance (CrCl) (mL/min)} = \frac{0.85\times(140-\text{age})\times\text{weight (kg)}^{a}}{0.81\times\text{serum creatinine }(\mu\text{mol/L})}$$

a Age is measured in years, and weight is in kilograms.

Appendix 3: Response evaluation criteria in solid tumors

Solid Tumor Response Evaluation Criteria Version 1.1 (Excerpt)

(New Response Evaluation Criteria in Solid Tumors：Revised RECIST Version 1.1)

Note: This attachment is an internal translation document, provided for reference only. Please refer to the English version for actual operations.

1 Background

Omitted

2 Objectives

Omitted

3 Tumor Measurability at Baseline

3.1 Definitions

At baseline, tumor lesions/lymph nodes will be categorized measurable or non measurable as follows:

3.1.1 Measurable

Tumor Lesions: At least one measurable diameter (recorded as the maximum diameter) with a minimum length as follows:

- CT scan: 10 mm (CT scan slice thickness not exceeding 5mm)
- Clinical routine examination instruments: 10 mm (lesions that cannot be accurately measured with a caliper should be recorded as non-measurable)
- Chest X-ray: 20 mm
- Malignant lymph nodes: Pathologically enlarged and measurable, with a single lymph node CT scan short axis ≥15 mm (CT scan slice thickness recommended not to exceed 5 mm). Only the short axis is measured and followed up at baseline and during follow-up.

3.1.2 Non-measurable

All other lesions, including small lesions (longest diameter <10 mm or pathological lymph nodes with ≥10 to <15 mm short axis), as well as truly non-measurable lesions are considered non-measurable. Lesions considered truly non-measurable include: leptomeningeal disease, ascites, pleural or pericardial effusion, inflammatory breast disease, lymphangitic involvement of skin or lung, and abdominal masses/abdominal organomegaly

identified by physical examination that is not measurable by reproducible imaging techniques.

3.1.3 Special Considerations Regarding Lesion Measurability

Bone lesions, cystic lesions, and lesions previously treated with local therapy require particular comment:

Bone lesions

- Bone scan, positron emission tomography scan or plain films are not considered adequate imaging techniques to measure bone lesions. However, these techniques can be used to confirm the presence or disappearance of bone lesions.
- Lytic bone lesions or mixed lytic-blastic lesions, with identifiable soft tissue components, that can be evaluated by cross sectional imaging techniques such as CT or MRI can be considered as measurable lesions if the soft tissue component meets the definition of measurability described above.
- Blastic bone lesions are non-measurable.

Cystic lesions

Lesions that meet the criteria for radiographically defined simple cysts should not be considered as malignant lesions (neither measurable nor non-measurable) since they are, by definition, simple cysts.

'Cystic lesions' thought to represent cystic metastases can be considered as measurable lesions, if they meet the definition of measurability described above. However, if noncystic lesions are present in the same subject, these are preferred for selection as target lesions.

Lesions with prior local treatment

Tumor lesions situated in a previously irradiated area, or in an area subjected to other loco-regional therapy, are not considered measurable unless there has been demonstrated progression in the lesion since the therapy.

3.2 Specifications by Methods of Measurements

3.2.1 Measurement of Lesions

All measurements should be recorded in metric notation. All baseline evaluations should be performed as close as possible to the treatment start and NEVER more than 4 weeks before the beginning of the treatment.

3.2.2 Method of Assessment

The evaluation of lesions at baseline and subsequent measurements should employ the same techniques and methods. Except for lesions that cannot be evaluated by imaging studies and are only assessable by clinical examination, all lesions must be evaluated using imaging studies.

Clinical Lesions: Clinical lesions can be considered measurable lesions only if they are superficial and have a diameter of ≥10 mm when measured (e.g., skin nodules). For patients with skin lesions, it is recommended to archive colored photographs with a ruler for measuring lesion size. Whenever possible, imaging evaluations should be prioritized over clinical assessments, as imaging is more objective and allows for reproducible review at the end of the study.

Chest X-Ray: When tumor progression is a significant study endpoint, priority should be given to chest CT scans, as CT is more sensitive than X-rays, especially for detecting new lesions. Chest X-rays are applicable only when the measured lesions have clear boundaries and good lung ventilation.

CT, MRI: CT is currently the preferred and repeatable method for evaluating therapeutic efficacy. The definition of measurability is based on CT scan slice thickness ≤5 mm. If the CT slice thickness exceeds 5 mm, the minimum measurable lesion size should be twice the slice thickness. MRI is also acceptable in some cases (e.g., whole-body scans).

Ultrasound: Ultrasound should not be used as a method for measuring lesion size. Due to its operator-dependent nature and lack of repeatability after measurement, ultrasound cannot ensure technical and measurement consistency between different assessments. If new lesions are detected by ultrasound during the trial, confirmation should be obtained using CT or MRI. MRI can be used as an alternative to CT considering the radiation exposure associated with CT.

Endoscopy, Laparoscopic Examination: These techniques are not recommended for the objective assessment of tumors. However, they can be used to confirm complete response (CR) when obtaining biopsy specimens, and can be used to confirm recurrence after CR in trials where recurrence or surgical resection is an endpoint.

Tumor Markers: Tumor markers cannot be used alone to evaluate objective tumor responses. However, if the marker levels exceed the upper limit of normal at baseline, they must return

to normal levels to assess complete response. Since tumor markers vary by disease, this factor should be considered when drafting measurement criteria in the protocol. Specific criteria for CA-125 relief (recurrent ovarian cancer) and PSA (recurrent prostate cancer) relief have been published. The International Gynecologic Cancer Society has also developed CA-125 progression criteria, which are likely to be included in the objective tumor assessment criteria for first-line ovarian cancer treatment protocols.

Cytology/Histology Techniques: In specified circumstances as outlined in the protocol, these techniques can be used to identify PR and CR (e.g., residual benign tumor tissue commonly exists in lesions of germ cell tumors). When exudate may be a potential side effect of therapy (e.g., treatment with taxanes or anti-angiogenic agents), and measurable tumors meet the criteria for relief or disease stabilization, the appearance or exacerbation of tumor-related exudate during treatment can be diagnosed through cytological techniques to differentiate between relief (or disease stabilization) and disease progression.

4 Tumor Response Evaluation

4.1 Assessment of Overall Tumor Burden and Measurable Disease

To evaluate objective relief or potential progression, it is necessary to perform a baseline assessment of the total tumor burden of all tumor lesions, as a reference for subsequent measurements. In clinical protocols where objective relief is the primary treatment endpoint, only patients with measurable lesions at baseline are eligible for inclusion. Measurable lesions are defined as having at least one lesion that can be measured. For trials where disease progression (time to progression or degree of progression at a fixed date) is the primary treatment endpoint, the inclusion criteria in the protocol must clearly specify whether eligibility is limited to patients with measurable lesions only or if patients without measurable lesions can also be included.

4.2 Baseline Documentation of ‘Target’ and ‘Nontarget’ Lesions

When more than 1 measurable lesion is present at baseline all lesions up to a total of 2 lesions per organ and a maximum of 5 lesions total (representative of all involved organs, with a maximum of 2 per organ) should be identified as target lesions and will be recorded and measured at baseline (this means in instances where subjects have only 1 or 2 organ sites involved a maximum of 2 and 4 lesions respectively will be recorded).

Target lesions should be selected on the basis of their size (lesions with the longest diameter), be representative of all involved organs, but in addition should be those that lend themselves to reproducible repeated measurements. It may be the case that, on occasion, the largest lesion does not lend itself to reproducible measurement in which circumstance the next largest lesion that can be measured reproducibly should be selected.

Lymph nodes merit special mention since they are normal anatomical structures that may be visible by imaging even if not involved by tumor. As noted above, pathological nodes that are defined as measurable and may be identified as target lesions must meet the criterion of a short axis of ≥15 mm by CT scan. Only the short axis of these nodes will contribute to the baseline sum of lesion diameters. The short axis of the node is the diameter normally used by radiologists to judge if a node is involved by solid tumor. Nodal size is normally reported as 2 dimensions in the plane in which the image is obtained (for CT scan this is almost always the axial plane; for MRI the plane of acquisition may be axial, sagittal, or coronal). The smaller of these measures is the short axis. For example, an abdominal node that is reported as being 20 mm × 30 mm has a short axis of 20 mm and qualifies as a malignant, measurable node. In this example, 20 mm should be recorded as the node measurement. Up to 2 nodal target lesions can be recorded. All other pathological nodes (those with short axis ≥10 mm but <15 mm) should be considered non-target lesions. Nodes that have a short axis <10 mm are considered non-pathological and should not be recorded.

A sum of the diameters (longest diameter for non-nodal lesions, short-axis diameter for nodal lesions) for all target lesions will be calculated and reported as the baseline sum of diameters. If lymph nodes are to be included in the sum, then as noted above, only the short axis is added into the sum. The baseline sum of diameters will be used as reference to further characterize any objective tumor regression in the measurable dimension of the disease.

All other lesions (or sites of disease) including pathological lymph nodes should be identified as non-target lesions and should also be recorded at baseline. Measurements are not required and these lesions should be followed as “present,” “absent,” or in rare cases “unequivocal progression.” In addition, it is possible to record multiple non-target lesions involving the same organ as a single item on the case report form (eg, ‘multiple enlarged

pelvic lymph nodes’ or ‘multiple liver metastases’).

4.3 Response Criteria

4.3.1 Evaluation of Target Lesions

Complete Response (CR): Disappearance of all target lesions. Any pathological lymph nodes (whether target or non-target) must have reduction in short axis to <10 mm.

Partial Response (PR): At least a 30% decrease in the sum of diameters of target lesions, taking as reference the baseline sum diameters.

Progressive Disease (PD): At least a 20% increase in the sum of diameters of target lesions, taking as reference the smallest sum on study (this includes the baseline sum if that is the smallest on study). In addition to the relative increase of 20%, the sum of diameters must also demonstrate an absolute increase of at least 5 mm. (Note: The appearance of one or more new lesions is also considered progression.)

Stable Disease (SD): Neither sufficient shrinkage to qualify for PR (taking as reference the sum of diameters at baseline) nor sufficient increase to qualify for PD (taking as reference the smallest sum of diameters while on study).

4.3.2 Special Notes on the Assessment of Target Lesions

Lymph nodes: Lymph nodes identified as target lesions should always have the actual short axis measurement recorded (measured in the same anatomical plane as the baseline examination), even if the nodes regress to below 10 mm on study. This means that when lymph nodes are included as target lesions, the ‘sum’ of lesions may not be zero even if CR criteria are met, since a normal lymph node is defined as having a short axis of <10 mm. For PR, SD, and PD, the actual short axis measurement of the nodes is to be included in the sum of target lesions.

Target lesions that become ‘too small to measure’: While on study, all lesions (nodal and non-nodal) recorded at baseline should have their actual measurements recorded at each subsequent evaluation, even when very small (eg, 2 mm). However, sometimes lesions or lymph nodes that are recorded as target lesions at baseline become so faint on CT scan that the radiologist may not feel comfortable assigning an exact measure and may report them as being ‘too small to measure.’ When this occurs, it is important that a value be recorded on the eCRF. If it is the opinion of the radiologist that the lesion has likely disappeared, the

measurement should be recorded as 0 mm. If the lesion is believed to be present and is faintly seen but too small to measure, a default value of 5 mm should be assigned (Note: It is less unlikely that this rule will be used for lymph nodes since they usually have a definable size when normal and are frequently surrounded by fat such as in the retro-peritoneum; however, if a lymph node is believed to be present and is faintly seen but too small to measure, a default value of 5 mm should be assigned in this circumstance as well. This default value is derived from the 5 mm CT slice thickness (but should not be changed with varying CT slice thickness.) The measurement of these lesions is potentially non-reproducible; therefore providing this default value will prevent false responses or progressions based upon measurement error. To reiterate, however, if the radiologist is able to provide an actual measure, that should be recorded, even if it is below 5 mm.

Lesions that split or coalesce on treatment: When non-nodal lesions “fragment,” the longest diameters of the fragmented portions should be added together to calculate the target lesion sum. Similarly, as lesions coalesce, a plane between them may be maintained that would aid in obtaining maximal diameter measurements of each individual lesion. If the lesions have truly coalesced such that they are no longer separable, the vector of the longest diameter in this instance should be the maximal longest diameter for the “coalesced lesion.”

4.3.3 Evaluation of Non-target Lesions

This section provides the definitions of the criteria used to determine the tumor response for the group of non-target lesions. While some non-target lesions may actually be measurable, they need not be measured and instead should be assessed only qualitatively at the time points specified in the protocol.

Complete Response: Disappearance of all non-target lesions. All lymph nodes must be non-pathological in size (<10 mm short axis).

Progressive Disease: Unequivocal progression (see comments below) of existing nontarget lesions. (Note: The appearance of one or more new lesions is also considered progression.)

Non-CR/Non-PD: Persistence of one or more non-target lesion(s).

4.3.4 Special Notes on Assessment of Progression of Non-target Disease

The concept of progression of non-target disease requires additional explanation as follows:

When the subject also has measurable disease: In this setting, to achieve ‘unequivocal progression’ on the basis of the non-target disease, there must be an overall level of substantial worsening in non-target disease such that, even in presence of SD or PR in target disease, the overall tumor burden has increased sufficiently to merit discontinuation of therapy. A modest “increase” in the size of 1 or more non-target lesions is usually not sufficient to qualify for unequivocal progression status. The designation of overall progression solely on the basis of change in non-target disease in the face of SD or PR of target disease will therefore be rare.

When the subject has only non-measurable disease: The same general concepts apply here as noted above; however, in this instance there is no measurable disease assessment to factor into the interpretation of an increase in non-measurable disease burden. Because worsening in non-target disease cannot be easily quantified (by definition: if all lesions are truly non-measurable) a useful test that can be applied when assessing subjects for unequivocal progression is to consider if the increase in overall disease burden based on the change in non-measurable disease is comparable in magnitude to the increase that would be required to declare PD for measurable disease (ie, an increase in tumor burden representing an additional 73% increase in “volume” [which is equivalent to a 20% increase diameter in a measurable lesion]). If ‘unequivocal progression’ is seen, the subject should be considered to have had overall PD at that time point. While it would be ideal to have objective criteria to apply to non-measurable disease, the very nature of that disease makes it impossible to do so; therefore, the increase must be substantial.

4.3.5 New Lesions

The emergence of new malignant lesions indicates disease progression; therefore, evaluation of new lesions is crucial. Currently, there are no specific criteria for the detection of new lesions by imaging; however, the discovery of a new lesion should be clear. For instance, progression cannot be attributed to differences in imaging techniques, changes in imaging morphology, or other non-tumor-related conditions (e.g., some so-called new bone lesions are simply the healing or recurrence of the original lesion). This is particularly important when partial or complete response of baseline lesions occurs; for example, necrosis of a liver lesion may be reported as a new cystic lesion on a CT scan report, when in fact it is not.

Lesions detected during follow-up that were not identified at baseline examinations will be considered new lesions and indicative of disease progression. For example, if a patient with visceral lesions detected at baseline examinations is found to have metastases during a CT or MRI examination of the head, the intracranial metastatic lesions in this patient will be considered evidence of disease progression, even if head examinations were not performed at baseline.

If a new lesion is ambiguous, such as due to its small size, further treatment and follow-up assessments are required to confirm whether it is indeed a new lesion. If repeated examinations confirm it to be a new lesion, then the time of disease progression should be calculated from its initial discovery.

Assessment of lesions using FDG-PET generally requires additional testing for confirmation. Combining FDG-PET and supplementary CT scan results to evaluate disease progression, especially for new suspicious lesions, is reasonable. New lesions can be clarified by FDG-PET examination according to the following procedure:

- If the baseline FDG-PET examination result is negative and subsequent follow-up FDG-PET examinations are positive, it indicates disease progression.
- If no baseline FDG-PET examination was conducted, and subsequent FDG-PET examinations are positive:
- If the new lesion identified in the follow-up FDG-PET examination corresponds to the CT scan results, it confirms disease progression.
- If the new lesion identified in the follow-up FDG-PET examination is not confirmed by CT scan results, confirmation by CT scan is required (if confirmed, disease progression time is calculated from the time of abnormality detected in the previous FDG-PET examination).
- If the new lesion identified in the follow-up FDG-PET examination corresponds to an existing lesion identified by CT scan and there is no progression on imaging, then there is no disease progression.

4.4 Evaluation of Best Overall Response

The best overall treatment response is the best efficacy record from the start to the end of the trial, taking into account any necessary conditions for confirmation. Sometimes, efficacy responses occur after the end of treatment, so the protocol should specify whether

post-treatment efficacy evaluations are considered in the best overall treatment response. The protocol must specify how any new treatments before progression affect the best treatment response. The best treatment response of patients mainly depends on the results of target lesions, non-target lesions, and the manifestation of new lesions. Additionally, it relies on the nature of the trial, protocol requirements, and outcome measurement criteria. Specifically, in non-randomized trials, the efficacy response situation is the primary objective, and confirmation of PR or CR efficacy is necessary to determine which is the best overall treatment response.

4.4.1 **Time Point Response**

Assuming efficacy responses occur at specific time points in each protocol, Table 1 will provide a summary of the overall treatment responses at each time point for the population of patients with measurable disease at baseline.

Table 1 Time Point Response: Subjects with Target Disease

Target Lesions	Non-target Lesions	New Lesions	Time Point Response
CR	CR	No	CR
CR	Non-CR/non-PD	No	PR
CR	NE	No	PR
PR	Non-PD or NE	No	PR
SD	Non-PD or NE	No	SD
NE	Non-PD	No	NE
PD	Any	Yes or No	PD
Any	PD	Yes or No	PD
Any	Any	Yes	PD

CR = complete response; PR = partial response; SD = stable disease; PD = progressive disease; NE = not evaluable

If patients have no measurable lesions (no target lesions), the assessment can refer to Table 2.

Table 2 Time Point Response: Subjects with non-target Disease

Non-target Lesions	New Lesions	Time Point Response
CR	No	CR
Non-CR/non-PD	No	Non-CR/non-PD [a]
NE	No	NE
Uncertain PD	Yes or No	PD
Any	Yes	PD

a：For non-target lesions, "non-CR/non-PD" refers to a response better than stable disease (SD). As SD is increasingly used as an endpoint to assess efficacy, defining non-CR/non-PD responses is necessary for

cases where no measurable lesions are specified.

4.4.2 Missing Assessments and Non-evaluable Designation

When no imaging/measurement is done at all at a particular time point, the subject is not evaluable (NE) at that time point. If only a subset of lesion measurements are made at an assessment, usually the case is also considered NE at that time point, unless a convincing argument can be made that the contribution of the individual missing lesion(s) would not change the assigned time point response. This would be most likely to happen in the case of PD. For example, if a subject had a baseline sum of 50 mm with 3 measured lesions and at follow-up only 2 lesions were assessed, but those gave a sum of 80 mm, the subject will have achieved PD status, regardless of the contribution of the missing lesion

4.4.3 Best Overall Response: All Time Points

Once all the patient data is available, their best overall response can be determined.

Assessment of best overall response when confirmation of complete or partial response is not required in the study:

The best response at all time points during the trial is considered (e.g., a patient may have stable disease (SD) at the first cycle assessment, partial response (PR) at the second cycle assessment, and progressive disease (PD) at the last cycle assessment, but their best overall response is PR). However, if the best overall response is SD, it must meet the minimum duration criteria specified in the protocol from baseline. If the criteria for the minimum duration are not met, even if the best overall response is SD, it will not be accepted, and the patient's best overall response will be determined by subsequent assessments. For example, a patient with SD at the first cycle assessment, followed by PD at the second cycle, but who did not meet the minimum duration requirement for SD, their best overall response will be PD. Similarly, a patient who is lost to follow-up after being assessed with SD at the first cycle will be considered non-evaluable.

Assessment of best overall response when confirmation of complete or partial response is required in the study:

Only when each subject meets the trial-specified criteria for complete or partial response and is specifically mentioned in the protocol to have the response confirmed at subsequent time

points (generally four weeks later) can it be declared as complete or partial response. In this case, refer to the explanation in Table 3 for the best overall response.

Table 3 **Best Overall Response Requiring Confirmation of CR and PR Efficacy**

Initial Time Point Overall Response	Subsequent Time Point Overall Response	Best Overall Response
CR	CR	CR
CR	PR	SD，PD or PR[a]
CR	SD	If the duration of SD is sufficient, it is classified as SD; otherwise, it should be classified as PD.
CR	PD	If the duration of SD is sufficient, it is classified as SD; otherwise, it should be classified as PD.
CR	NE	If the duration of SD is sufficient, it is classified as SD; otherwise, it should be classified as NE.
PR	CR	PR
PR	PR	PR
PR	SD	SD
PR	PD	If the duration of SD is sufficient, it is classified as SD; otherwise, it should be classified as PD.
PR	NE	If the duration of SD is sufficient, it is classified as SD; otherwise, it should be classified as NE.
NE	NE	NE

CR = complete response; PR = partial response; SD = stable disease; PD = progressive disease; NE = not evaluable

a: If a complete response (CR) genuinely occurs at the first time point, any disease that appears at subsequent time points means that even if the subject's efficacy reaches the partial response (PR) criteria relative to baseline, their efficacy evaluation at later time points will still be progressive disease (PD) (because the disease reappears after CR). The best remission depends on whether stable disease (SD) occurs within the shortest treatment interval. However, sometimes the first evaluation is CR, but subsequent time point scans suggest that small lesions still appear, so in fact, the subject's efficacy should have been PR rather than CR at the first time point. In this case, the initial CR judgment should be revised to PR, and the best response is PR.

4.4.4 Special Notes on Response Assessment

When nodular lesions are included in the total assessment of target lesions, and their size reduces to "normal" (<10 mm), they will still have a lesion size report on the scan. To prevent overestimation based on increases in nodule size, measurements will be recorded even when nodules are considered normal. As previously mentioned, this means that for subjects achieving complete remission (CR), the case report forms (CRFs) will not record a zero size.

If confirmation of response is required during the trial, repeated "non-measurable" time points can complicate the assessment of best response. The trial's analytical plan must specify

how these missing data/assessments can be clearly interpreted when determining response efficacy. For example, in most trials, a subject's response pattern of PR-NE-PR can be considered as confirmed efficacy.

When a subject exhibits overall health deterioration that necessitates cessation of drug administration without objective evidence, it should be reported as symptomatic progression. Objective progression should be assessed as much as possible even after treatment discontinuation. Symptomatic worsening is not a description of an objective response; it is a reason for stopping treatment. Such subjects' objective responses will be assessed based on target and non-target lesion conditions as shown in Tables 1 to 3.

Situations defined as early progression, early death, and unassessable should be explicitly described in each protocol, depending on the treatment intervals and cycles.

In some cases, it can be difficult to distinguish local lesions from normal tissue. When a complete remission assessment is based on such definitions, it is recommended to perform a biopsy before assessing complete remission of local lesions. When imaging results of some subjects' local lesions are considered indicative of lesion fibrosis or scar formation, FDG-PET is regarded as a similar standard to biopsy for confirming efficacy in complete remission. In such cases, the use of FDG-PET should be prospectively described in the protocol, supported by reports from the relevant medical literature. However, it is important to be aware of the limitations of both FDG-PET and biopsy (including their resolution and sensitivity), which may lead to false positive results in assessing complete remission.

Treatment may continue until the next assessment for unclear progression findings (such as very small, indeterminate new lesions; cystic changes or necrosis of existing lesions). If disease progression is confirmed at the next assessment, the progression date should be backdated to the date when suspected progression was first noted.

4.5 Frequency of Tumor Re-evaluation

The frequency of tumor re-evaluation during treatment is determined by the treatment plan and should align with the type and schedule of therapy. However, in Phase II trials where the benefit of treatment is unclear, it is reasonable to conduct follow-ups every 6-8 weeks (timed at the end of a cycle), with adjustments possible in special protocols or circumstances. The protocol should specifically indicate which anatomical sites require baseline assessment

(usually those most likely associated with metastasis of the tumor type under study) and the frequency of repeated evaluations. Normally, both target and non-target lesions should be assessed at each evaluation. In some optional scenarios, the frequency of non-target lesion evaluation may be reduced, for example, when the efficacy evaluation of the target disease confirms a complete response (CR) or when a bone scan is repeated only if there is suspected progression of osseous lesions.

After the treatment ends, re-evaluation of the tumor depends on whether response rate or time to an event (progression/death) is used as the clinical trial endpoint. For time to an event (e.g., TTP/DFS/PFS), routine repeat evaluations as specified in the protocol are required. Particularly in randomized comparative trials, scheduled evaluations should be listed in the timeline (e.g., 6-8 weeks during treatment, or 3-4 months post-treatment) and should not be affected by other factors such as treatment delays, dosing intervals, or any other events that could cause imbalance in treatment arms regarding the timing of disease evaluation.

4.6 Assessment of Efficacy/Confirmation of Remission

4.6.1 Confirmation

For non-randomized clinical studies where efficacy is the primary endpoint, confirmation of Partial Response (PR) and Complete Response (CR) must be obtained to ensure that the efficacy is not the result of assessment error. This also allows for a reasonable interpretation of the results in the context of historical data, but the efficacy in these historical data should also have been confirmed. However, in all other cases, such as randomized trials (Phase II or III) or studies where the primary endpoints are disease stabilization or disease progression, confirmation of efficacy is no longer required, as it does not add value to the interpretation of the trial results. Nevertheless, the removal of the requirement for efficacy confirmation makes the role of central review to prevent bias more important, especially in open-label studies.

In the case of Stable Disease (SD), at least one measurement meeting the SD criteria specified in the protocol should occur within the shortest interval after the start of the trial, generally not less than 6-8 weeks.

4.6.2 Duration of Overall Remission

Duration of Overall Remission is measured from the time the criteria for Complete Response (CR) or Partial Response (PR) (whichever is measured first) are met, to the time the disease

is first officially recorded as recurring or progressing (using the smallest measurement recorded during the trial as a reference for disease progression). Duration of Complete Remission is measured from the time the criteria for CR are first met, to the time the disease is first officially recorded as recurring or progressing.

4.6.3 Duration of Stable Disease

Duration of Stable Disease is defined as the time from the start of treatment to the time of disease progression (from the time of randomization in randomized trials), using the smallest total sum as a reference (if the baseline sum is the smallest, it is used as the reference for calculating Progressive Disease (PD)). The clinical relevance of the duration of stable disease varies between different studies and diseases. If a trial specifically uses the proportion of patients maintaining stable disease for a minimum duration as a study endpoint, the protocol should explicitly specify the minimum interval between two measurements for defining Stable Disease (SD).

Note: The durations of remission, stable disease, and Progression-Free Survival (PFS) are influenced by the frequency of follow-up assessments after baseline evaluation. Defining standard follow-up frequencies is beyond the scope of these guidelines. Follow-up frequency should consider various factors, such as disease type and stage, treatment cycles, and standard protocols. However, the limitations of the accuracy of these measurement endpoints should be considered if comparisons between trials are necessary.

4.7 PFS/TTP

4.7.1 Phase II Clinical Trials

This guideline primarily focuses on the use of objective response as a study endpoint in Phase II clinical trials. In some cases, response rates may not be the optimal choice for evaluating the potential anti-cancer activity of a new drug/new regimen. In these instances, progression-free survival (PFS) or progression-free probability (PPF) at a defined time point can be considered suitable alternative indicators for providing initial signals of the new drug's biological activity. However, it is clear that in a non-controlled trial, these assessments can be questioned because seemingly valuable observations may be related to biological factors such as patient selection, rather than the effect of the drug intervention. Therefore, Phase II clinical trials using these as study endpoints are best designed as randomized controlled trials.

However, in certain tumors with consistently poor clinical outcomes, non-randomized trials are also reasonable. In these cases, due to the absence of a positive control, care must be taken in documenting efficacy evidence when assessing expected PFS or PPF.

吡咯替尼、达尔西利（SHR6390）联合内分泌治疗在双受体阳性（ER+/HER2+）晚期乳腺癌的多中心 Ib/II 期临床研究

Pyrotinib, daLpiciclib(SHR6390) and Endocrine therApy in Subjects with dUal-Receptor positive(ER+/HER2+) Advanced Breast cancer: a muLti-center phasE Ib/II study

研究方案

研究方案名称：	PLEASURABLE (LORDSHIPS 3.0/YBCSG-20-01)
研究方案版本号：	3.0
研究方案版本日期：	2022 年 12 月 21 日
临床研究负责人：	胡夕春教授
临床研究协调人：	张剑教授、孟艳春医生、陶中华医生
临床研究组长单位：	复旦大学附属肿瘤医院

目　录

方案摘要......4

研究流程图......13

缩略语表......18

1 研究背景......20

1.1 药品名称......23

1.2 SHR6390 的药理类型和作用机制......23

1.3 SHR6390 的药效学研究......24

1.4 SHR6390 的毒理学研究......24

1.5 SHR6390 的药代动力学研究......24

1.6 吡咯替尼联合 SHR6390 在 ER+、HER2+ 乳腺癌肿瘤模型体内抑瘤作用......24

2 临床研究......24

2.1 马来酸吡咯替尼临床研究......24

2.2 SHR6390 临床研究......24

2.3 目前已上市 CDK4/6 抑制剂临床研究......25

3 研究目的与研究终点......26

3.1 主要研究目的......26

3.2 主要研究终点......26

3.3 次要研究终点......27

4 试验药品......27

4.1 名称和来源......27

4.2 药品剂型和规格......27

4.3 保存条件......27

4.4 使用方法......27

4.5 试验药物的管理、发放与回收......28

5 研究总体设计......28

6 实验设计......28

6.1 药品的剂量和给药方案......28

6.2 剂量限制性毒性（DLT）......29

6.3 最大耐受剂量(MTD)......30

6.4 受试者替换......30

6.5 样本量计算......30

7 标本收集......30

8 受试者的选择......31

8.1 受试者及研究例数......31

8.2 入选标准......31

8.3 排除标准......32

8.4 受试者识别......33

8.5 脱落标准......34

8.6 受试者治疗终止标准 34
8.7 研究终止标准 34
9 剂量调整与伴随用药 34
9.1 DLT 观察期 34
9.2 连续给药第 2 周期及后续周期 34
9.3 研究期间不可使用的药物 35
9.4 研究期间可酌情使用的药物 35
10 研究步骤 36
10.1 筛选期 36
10.2 不良事件的收集/试验期 37
10.3 研究治疗结束/退出研究 38
10.4 治疗结束后随访 38
11 安全性评价 39
11.1 不良事件(AE) 39
11.2 严重不良事件（SAE） 41
12 严重不良事件的报告制度 42
13 SAE 的报告程序 43
14 疗效评价 43
15 研究结束 43
16 安全控制措施 43
17 推荐Ⅱ期临床试验给药方案 44
18 伦理规范及知情同意 44
18.1 伦理规范 44
18.2 知情同意 44
19 临床试验的质量保证 45
20 数据处理 45
20.1 研究者填写数据要求 45
20.2 数据的可溯源性、病例报告表（CRF）的填写 45
20.3 统计分析数据的选择 45
20.4 统计分析计划 46
附件一 身体状况评分标准（ECOG） 48
附件二 肌酐清除率计算 49
附件三 实体肿瘤的疗效评价标准 50

方案摘要

研究题目	吡咯替尼、达尔西利（SHR6390）联合内分泌治疗在双受体阳性（ER+/HER2+）晚期乳腺癌的多中心 Ib/II 期临床研究
研究方案名称	PLEASURABLE (LORDSHIPS 3.0/YBCSG-20-01))
版本号	3.0
版本日期	2022 年 12 月 21 日
组长单位	复旦大学附属肿瘤医院
主要研究者	胡夕春教授
协调研究者	张剑教授、孟艳春医生、陶中华医生
研究对象	ER受体阳性、HER2阳性晚期乳腺癌
研究目的	**第一阶段（Ib 期）：**<br>确定非甾体类芳香化酶抑制剂（来曲唑）、马来酸吡咯替尼片联合 CDK4/6 抑制剂 SHR6390 治疗激素受体阳性、HER2 阳性晚期乳腺癌的安全性和耐受性，结合初步的有效性数据，明确此联合方案的 II 期推荐给药剂量。<br>**第二阶段（II 期）：**<br>评估吡咯替尼、达尔西利（SHR6390）联合内分泌治疗在双受体阳性（ER+、HER2+）晚期乳腺癌的有效性和安全性。

<table>
<tr><td>终点指标</td><td>
<table>
<tr><td></td><td>第一阶段（I 期）</td><td>第二阶段（II 期）</td></tr>
<tr><td>主要终点指标</td><td>• 联合方案中 SHR6390 的剂量限制性毒性（DLT）和最大耐受剂量（MTD），确定 II 期临床研究推荐给药方案；
• 各剂量组的不良事件（AE）及严重不良事件（SAE）的发生率及严重程度。</td><td>II 期临床研究推荐给药方案的客观有效率（ORR），基于 RECIST1.1</td></tr>
<tr><td rowspan="2">次要终点指标
•</td><td>ORR，基于 RECIST1.1</td><td>AE 和 SAE 的发生率及严重程度</td></tr>
<tr><td colspan="2">• 无进展生存期（PFS）；
• 疾病控制率（DCR）：疗效评价为 CR/PR/SD 的受试者比例；
• 临床获益率（CBR）：研究过程中出现 CR、PR 及 SD≥24 周的受试者比例；
• 缓解持续时间（DoR）；
• 药代动力学（PK）
• 探索性分析分子标志物与疗效之间的关系。</td></tr>
</table>
</td></tr>
<tr><td>样本量</td><td>第一阶段（Ib 期）：每个剂量组 3~6 例受试者。A、B 组共 6~12 例受试者。如使用备用剂量 C、D、 E、 F 组，每组将增加 3~6 例受试者。
第二阶段（II 期）：吡咯替尼、SHR6390 联合内分泌治疗（来曲唑或氟维司群）：48 例受试者。</td></tr>
<tr><td>研究设计</td><td>第一阶段（Ib 期）：单臂、开放、剂量爬坡的 Ib 期临床研究
第二阶段（II 期）：单臂、开放、多中心 II 期临床研究</td></tr>
<tr><td>给药方案</td><td>第一阶段（Ib 期）：
本研究来曲唑给药剂量 2.5 mg/d 或阿那曲唑给药剂量 1 mg/d，吡咯替尼给药剂量为 400 mg/d 或 320 mg/d，SHR6390 设计 3 个剂量组：
<table>
<tr><td>剂量组</td><td>SHR6390</td><td>吡咯替尼片</td><td>病例数</td></tr>
<tr><td>A</td><td>125 mg/d</td><td>400 mg/d</td><td>3~6</td></tr>
<tr><td>B</td><td>150 mg/d</td><td>400 mg/d</td><td>3~6</td></tr>
</table>
</td></tr>
</table>

C（备用组）	100 mg/d	400 mg/d	3~6
D（备用组）	125 mg/d	320 mg/d	3~6
E（备用组）	150 mg/d	320 mg/d	3~6
F（备用组）	100 mg/d	320 mg/d	3~6

非甾体类芳香化酶抑制剂(NSAIs)、马来酸吡咯替尼片联合 SHR6390 方案中，SHR6390 的起始剂量为 125 mg。遵循 3+3 原则，根据起始剂量组是否出现规定频率的 DLT，以 25 mg 为剂量单位进行后续

- 如果起始剂量 A 组 125mg 没有观察到规定频率的 DLT，则剂量 递增至 150mg 组，若该组出现规定频率的 DLT，则 125mg 剂量 被确定为 MTD。
- 如果起始剂量 A 组 125mg 组出现规定频率的 DLT，由研究者讨论同时启动备选 C 组及 D 组，如 C 组仍不能耐受，则启动备 用 F 组。并同时依次启用 D 组→E 组（SHR6390 剂量调整原则 同上），如 D 组仍不能耐受，启动备用 F 组。最后由研究者团 队共同分析试验数据确定联合给药 II 期推荐剂量。
- 若 F 组仍不能耐受则由研究者团队共同分析试验数据决定是否终止试验。
- 根据来曲唑、马来酸吡咯替尼片联合 SHR6390 联合用药组推荐 II 期给药剂量，启用阿那曲唑、马来酸吡咯替尼片联合 SHR6390 联合用药组，若出现不可耐受毒性需要调整用药，由研究者讨论后决定否行药物剂量调整。

每个剂量组受试者给药 1 周期结束后对 DLT 进行评估。

三药的具体服用方式为：

- 非甾体类芳香化酶抑制剂：来曲唑，口服，2.5 mg，口服，1mg，每天 1 次，空腹给药，连续给药 28 天为 1 个周期。
- 吡咯替尼：口服，400 mg 或 320 mg，每天 1 次，早餐后 30 分钟内口服给药，连续给药 28 天为 1 个周期。
- SHR6390：口服，每日 1 次，空腹给药（给药期间应至少保证服

药前 1 小时和服药后 1 小时禁食）。服药以 28 天为一个给药周期，每周期前 3 周（D1~21）连续服药，与来曲唑同时服用，第 4 周（D22~28）停药。

前 2 周期为核心试验阶段，第 2 周期末疗效评估为 CR/PR/SD 的受试者，可接受该剂量组联合方案持续给药至受试者出现疾病进展、不能耐受毒性或主动撤回知情同意书等情况为止。

第二阶段（II 期）：

根据第一阶段确定的联合给药 II 期推荐剂量，进行第二阶段扩组研究，吡咯替尼 320mg 及 SHR6390 125mg，联合内分泌治疗连续给药至受试者出现疾病进展、不能耐受毒性或主动撤回知情同意书等情况为止。

三药的具体服用方式为：

- 内分泌治疗：
  - ➢ 未证明芳香化酶抑制剂耐药（原发及继发）：来曲唑，口服，2.5 mg，每天 1 次，空腹给药，连续给药 28 天为 1 个周期。
  - ➢ 芳香化酶抑制剂耐药（原发及继发）：氟维司群 500mg，D1（第一周期 D15 增加一次给药），肌肉注射，28 天为一周期。
- 吡咯替尼：口服，320 mg，每天 1 次，早餐后 30 分钟内口服给药，连续给药 28 天为 1 个周期。
- SHR6390：口服，125mg，每日 1 次，空腹给药（给药期间应至少保证服药前 1 小时和服药后 1 小时禁食）。服药以 28 天为一个给药周期，每周期前 3 周（D1~21）连续服药，第 4 周（D22~28）停药。

研究设计图：

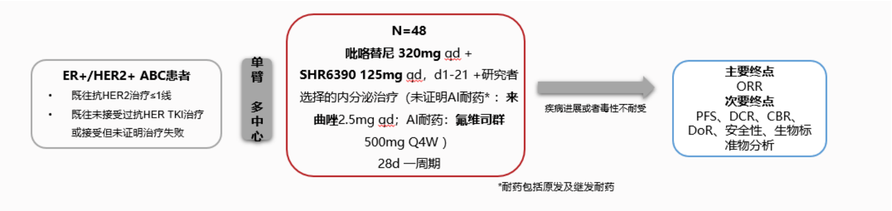

剂量限制性毒性（DLT）	**DLT 定义为在第 1 周期内出现的与药物相关或可能相关的以下不良事件（CTC-AE v4.0.3 标准）：**<br>1. 血液学毒性：<br>● 4 级中性粒细胞减少持续≥5 天；<br>● 4 级血小板减少，或 3 级血小板减少伴临床显著出血；<br>● ≥3 级中性粒细胞减少伴发热（≥38.0 摄氏度持续 1 小时或者>38.3 摄氏度）；<br>● ≥4 级贫血。<br>2. 非血液学毒性：<br>任何≥3 级非血液学毒性，以下情况除外：<br>● 3-4 级恶心/呕吐和/或腹泻和/或电解质紊乱，经最佳支持治疗后 72 小时内恢复至≤2 级者；<br>明确与肿瘤相关、与药物无关的 3-4 级的碱性磷酸酶、谷氨酰转肽酶升高。
最大耐受剂量（MTD）	**MTD 定义：**<br>在第 1 周期（28 天）的给药观察期内，若某一剂量组最终有≥1/3 的受试者出现 DLT，则前一剂量组为 MTD。如爬坡至 I 期临床试验 B 剂量组仍未能有 1/3 受试者出现 MTD，则由研究者团队讨论确定是否启用备用的 C 剂量组，并确定 II 期试验的推荐剂量。
入组标准	1. 受试者自愿加入本研究，签署知情同意书，有良好的依从性；<br>2. 年龄≥18 岁且≤75 岁的绝经后或绝经前/围绝经期女性患者，满足以下一条：<br>a) 既往进行过双侧卵巢切除术，或年龄≥60 岁；或<br>b) 年龄<60，自然绝经后状态（定义为连续至少 12 个月规律月经自发性停止且无其他病理或生理原因），E2 和 FSH 在绝经后水平；或<br>c) 绝经前或围绝经期女性患者也可以入选，但在研究期间必须愿意接受 LHRH 激动剂治疗；<br>3. 具有经组织病理确认的复发/转移性乳腺癌患者，且 ER 表达阳性，

	HER2 表达阳性；<br>•HER2 表达阳性指标准免疫组化染色（IHC）检测显示 HER2 为 3+和/或原位杂交技术（ISH）阳性；<br>•ER 阳性定义为 ER 表达阳性的细胞百分比≥1%；<br>•局部复发者需经研究者确认无法进行根治性手术切除；<br>4. 至少具有 1 个符合 RECIST 1.1 标准的颅外可测量病灶；<br>5. 既往抗肿瘤治疗的规定：<br>• 既往至多接受过 1 个针对复发转移性乳腺癌的含曲妥珠单抗方案的系统性治疗【包括抗 HER2 ADC，后续含义相同】<br>i. 晚期阶段未经含曲妥珠单抗方案治疗，或含曲妥珠单抗方案辅助治疗结束后 1 年以上复发的，后续治疗作为抗 HER2 一线治疗入组；<br>ii. 一线经过含曲妥珠单抗方案治疗失败，或含曲妥珠单抗方案辅助治疗期间复发或辅助治疗结束后 1 年以内复发的，后续治疗作为抗 HER2 二线治疗入组；<br>• 既往未接受过抗 HER2 TKI 治疗或接受但未证明治疗失败；<br>• 既往内分泌治疗未证明芳香化酶抑制剂耐药（耐药定义：辅助芳香化酶抑制剂治疗过程中或结束后 1 年以内复发，复发转移阶段接受过芳香化酶抑制剂并疾病进展），后续内分泌治疗选来曲唑。既往内分泌治疗存在芳香化酶抑制剂耐药，后续内分泌治疗选氟维司群。<br>6. ECOG 评分：0～1；<br>7. 预期生存期≥12 周；<br>8. 重要器官的功能符合下列要求（在入组前 2 周内未使用过任何血液成分及细胞生长因子）：<br>•中性粒细胞绝对计数≥1.5×10^9/L；<br>•血小板≥90×10^9/L；<br>•血红蛋白≥90g/L；<br>•总胆红素 TBIL≤1.5 倍 ULN；

	•ALT 和 AST ≤2.5 倍 ULN；<br>•尿素/尿素氮（BUN）和肌酐（Cr）≤1.5×ULN；<br>•左室射血分数（LVEF）≥50%；<br>•Fridericia 法校正的 QT 间期（QTcF）< 470 毫秒。<br>•INR≤1.5×ULN，APTT≤1.5×ULN。
排除标准	1. 脑膜转移或活动性脑实质转移。临床稳定脑实质转移患者可以入组，包括未接受过局部治疗的无症状的脑转移；或既往接受过中枢神经系统转移治疗（放疗或手术）的患者，如影像学证实稳定已维持至少 4 周、且已停止对症治疗（含激素和甘露醇等）大于 2 周才可以入组；<br>2. 既往接受过任何 CDK4/6 抑制剂治疗；<br>3. 基线期存在伴有临床症状的腹水、胸腔积液、心包积液，需要引流者，或首次用药前 4 周内进行过浆膜腔积液引流者；<br>4. 无法吞咽、肠梗阻或存在影响药物服用和吸收的其他因素；<br>5. 入组前 4 周内接受过化疗、分子靶向治疗或其他临床试验药物等系统治疗；入组前 2 周内接受过内分泌治疗；<br>6. 受试者既往 5 年内或同时患有其它恶性肿瘤（已治愈的皮肤基底细胞癌和宫颈原位癌除外）；<br>7. 在首次用药前 4 周内接受过重大手术操作或明显的创伤，或预计患者将要接受重大手术治疗；<br>8. 妊娠期、哺乳期女性受试者，或有生育能力女性的基线妊娠试验检测阳性，或不愿意采取有效避孕措施的育龄期受试者；<br>9. 已知对本方案药物组分有过敏史者；<br>10. 有活动性 HBV、HCV 感染者；经药物治疗后稳定的乙肝（HBV 病毒拷贝数不高于参考值上限）及已治愈的丙肝患者（HCV 病毒拷贝数低于测定法的检测下限）除外；<br>11. 有免疫缺陷病史，包括 HIV 检测阳性，或患有其他获得性、先天性免疫缺陷疾病，或有器官移植史；<br>12. 曾患有任何心脏疾病，包括：（1）心绞痛；（2）需药物治疗的

	或有临床意义的心率失常；（3）心肌梗死；（4）心力衰竭；（5）任何被研究者判断为不适于参加本试验的其他心脏疾病等；筛选期检查发现心脏功能或肾功能异常严重程度≥II 度；<br>13. 根据研究者的判断，有严重的危害患者安全、或影响患者完成研究的伴随疾病（如：严重的高血压、糖尿病、甲状腺疾病等）；<br>14. 既往有明确的神经或精神障碍史，包括癫痫或痴呆；<br>15. 首次用药前 4 周内并发重度感染（如：根据临床诊疗规范需要静脉滴注抗生素、抗真菌或抗病毒药物），或在筛选期间/首次给药前出现不明原因的发热>38.3°C。
受试者治疗终止标准	如出现以下一种及以上情况，该受试者须退出/终止治疗<br>1. 受试者撤回知情同意，要求退出；<br>2. 经影像学检查显示病情进展；<br>3. 无法耐受毒性者；<br>4. 严重违背试验方案，研究者评估认为应该终止治疗者；<br>5. 受试者失访或发生妊娠事件；<br>6. 研究者认为其他有必要退出研究的情况。
研究终止标准	本研究终止标准，包括但不限于：<br>1. 发现对受试者有非预期的、意义重大的或不可接受的风险；<br>2. 研究药物/试验治疗无效，或继续试验是无意义的；<br>3. 由于诸如受试者入选严重滞后或重大违背方案等原因，研究者决定终止研究。
安全性评价指标	依据 CTCAE v4.0.3 标准判断不良事件严重程度。试验期间应如实填写不良事件记录表，包括不良事件的发生时间、严重程度、持续时间、采取的措施和转归等。
疗效评价	入组受试者前 6 周期每 2 周期末（±7 天）进行 1 次影像学评价，6 周期之后每 3 周期末（±7 天）进行 1 次影像学评价，直至疾病进展或开始新的抗肿瘤治疗。根据 RECIST 1.1 标准进行肿瘤疗效评估。疾病进展或开始新的抗肿瘤治疗后每 12 周随访一次生存状态。

II 期探索性研究<br>（仅组长单位或有条件的参与单位）	1.患者基线、2 周期、进展后加做 FDG-PET、HER2-PET 检测，进一步探索其评估抗肿瘤疗效的临床应用价值<br>2.[redacted]<br>3. Circulating tumor DNA (ctDNA)<br>ctDNA 将在基线、周期 2 结束时、疾病进展或开始新的抗肿瘤治疗之前收集，作为评估抗肿瘤疗效的生物标志物。
统计方法	本试验结果主要采用统计描述方法。计量资料列出均数、标准差、中位数、最大值、最小值，计数资料和等级资料列出频数（构成比）、率、可信区间。<br>所有统计分析将采用 SAS 9.2 或以上版本统计分析软件编程计算。<br>**安全性分析：**<br>以描述性统计分析为主，对各剂量组所发生的不良事件、严重不良事件及与研究药物相关的不良事件等进行分析。实验室检验结果描述试验前正常但治疗后异常的情况。<br>**有效性分析：**<br>对客观缓解率（ORR）、疾病控制率（DCR）、临床获益率（CBR）等疗效终点进行点估计并提供其代表总体的 95%置信区间。生存率采用 Kaplan-Meier 法评估中位无进展生存时间、12 个月生存率以及其代表总体的 95%置信区间，并绘制生存图。对其它次要疗效指标均采用描述性分析。<br>**其他分析：**<br>对于可能影响疗效的分子标志物的水平与疗效之间的关系。
研究结束	末例受试者入组后 24 个月或研究者认为需提前结束试验时。

研究流程图

项目	筛选期		治疗期（28 天为一个治疗周期）				治疗后		生存随访
			第 1 周期			≥2 周期			
	第-28 天至第-1 天	第-7 天至第-1 天	第 15 天（±3d）	第 21 天（±3d）	第 28 天（±3d）	第 28 天（±3d）	治疗结束/退出	治疗结束后访视末次给药后 4 周	每 12 周（±7d）
基线资料									
签署知情同意	×								
人口学资料	×								
肿瘤病史/其他病史[1]	×								
伴随用药[2]	×		×						
实验室检查									
血常规[3]		×	×		×	×	如 7 天内未做	必要时	
尿常规[4]		×	每 3 周期一次				如 7 天内未做	必要时	
大便常规[5]		×	每 3 周期一次				如 7 天内未做	必要时	
血生化[6]		×	×		×	×	如 7 天内未做	必要时	
乙肝、丙肝及 HIV 检查[7]	×								
妊娠试验[8]		×						必要时	
临床评估、检查									
不良事件[9]	**自签署知情同意书至末次用药后 28 天**								
生命体征[10]		×	×		×	×	如 7 天内未做	×	

项目	筛选期		治疗期（28 天为一个治疗周期）				治疗后		生存随访
			第 1 周期			≥2 周期			
	第-28 天至第-1 天	第-7 天至第-1 天	第 15 天（±3d）	第 21 天（±3d）	第 28 天（±3d）	第 28 天（±3d）	治疗结束/退出	治疗结束后访视末次给药后 4 周	每 12 周（±7d）
体格检查[11]		×	×		×	×	如 7 天内未做	×	
ECOG 评分		×	×		×	×	如 7 天内未做	×	
心电图[12]		×	×		×	×	如 7 天内未做	必要时	
超声心动图[13]	×		每 3 周期一次				如 4 周内未做	必要时	
研究药物									
内分泌治疗给药[14]									
吡咯替尼给药[15]			**每日一次，餐后 30 分钟内口服**						
SHR6390 给药[16]			**每日一次，空腹口服**						
疗效评估									
影像学检查[17]	×		**每 2 周期末（±7 天）进行 1 次影像学评价，6 周期后每 3 周期末（±7 天）进行 1 次影像学评价，直至疾病进展或开始新的抗肿瘤治疗**						
[redacted][18]	×		[redacted]						
HER2-PET[19]	×		**第 2 周期治疗后（±7 天）及疾病进展后**						
FDG-PET[19]	×		**第 2 周期治疗后（±7 天）及疾病进展后**						
治疗结束后随访									
疾病进展时间[20]							**直至疾病进展或开始新的抗肿瘤治疗**		
生存随访[21]									×
血样采集及肿瘤样本采集/收集									
PK 采集[22]				×					

项目	筛选期		治疗期（28 天为一个治疗周期）				治疗后		生存随访
			第 1 周期			≥2 周期			
	第-28 天至第-1 天	第-7 天至第-1 天	第 15 天（±3d）	第 21 天（±3d）	第 28 天（±3d）	第 28 天（±3d）	治疗结束/退出	治疗结束后访视 末次给药后 4 周	每 12 周（±7d）
生物标志物采集/收集[23]	×					×（第 2 周期第 28 天±3 天）	×（疾病进展或开始新的抗肿瘤治疗前）		

备注：

[1] 肿瘤病史/其他疾病史：病理结果、ER/PR/HER2检测报告；肿瘤手术、化疗、放疗史以及其他疾病治疗史；除乳腺癌以外肿瘤病史。

[2] 记录开始用药前28天内以及研究期间的伴随用药和伴随治疗情况。一旦受试者中断试验治疗，仅应记录试验治疗相关的新发或未解决的不良事件所用的伴随用药和伴随治疗

[3] 血常规：血红蛋白、红细胞、白细胞、中性粒细胞计数、淋巴细胞计数和血小板计数。

[4] 尿常规：尿蛋白、尿糖、尿潜血（尿红细胞、白细胞）。如果半定量方法显示蛋白2+，则进行24小时尿蛋白定量检查。

[5] 大便常规：包括大便潜血。

[6] 血生化：总胆红素、结合胆红素、ALT、AST、AKP、γ-GT、LDH、总蛋白、白蛋白、尿素/尿素氮、肌酐、尿酸、空腹血糖、甘油三酯、胆固醇、钾、钠、氯、钙、磷、镁； 必要时增加心肌酶谱检查。

[7] 乙肝、丙肝及HIV检查：乙肝五项检查，若检查结果异常，应进行病毒复制（HBV DNA）检测；丙肝病毒抗体（抗HCV）、HIV抗体检查。

[8] 妊娠检查：育龄期女性首次用药前1周内进行血清妊娠检测。

[9] 不良事件：从签署知情同意开始记录不良事件，直至末次用药后至少28天，并且随访直至不良事件缓解或稳定，如受试者开始新的抗肿瘤治疗，随访至开始肿瘤治疗。

[10] 体征检查：体温、呼吸、脉搏、血压。

[11] 体格检查及体重测量：主要身体系统的检查（头面部、皮肤系统、淋巴结、眼部、耳鼻喉部、口腔、呼吸系统、心血管系统、腹部、生殖泌尿系统、肌肉骨骼、神经系统和精神状态），筛选期及研究结束时记录全面体格检查结果，试验期间仅须记录异常情况。

[12] 12导联心电图：若发现心电图异常有临床意义，研究者认为必要时可再次确认。

[13] 超声心动图：随访LVEF值变化，如出现LVEF下降至＜50%且较基线下降≥10%时，或出现胸痛、心悸等症状时，可增加计划外检查。

[14] 内分泌治疗：1）未证明芳香化酶抑制剂耐药（原发及继发）：来曲唑，口服，2.5 mg，每天1次，空腹给药，连续给药28天为1个周期。2）芳香化酶抑制剂耐药（原发及继发）：氟维司群500mg，D1（第一周期D15增加一次给药），肌肉注射，28天为一周期。

[15] 吡咯替尼：每天1次，320 mg，早餐后30分钟内口服给药，连续给药28天为1个周期。

[16] SHR6390（达尔西利）空腹口服，每日1次，125mg，服药3周（D1~21），停药1周（D22~28），28天为1个周期。对于SHR6390空腹服药的要求，早晨空腹口服，温水送服，连续给药期间服药前1小时和服药后1小时禁食。

[17] 影像学检查：筛选期影像学检查包括胸部、腹部的增强CT或MRI，以及研究者怀疑存在病灶的其他部位（如颈部、盆腔或颅脑）CT/MRI。肿瘤基线评估可放宽至首次给药前4周内，在签署知情同意前获得CT/MRI扫描结果只要符合要求可以用于筛选期肿瘤评估；临床怀疑有骨转移时需进行骨扫描检查。治疗期影像学检查应该在与基线检查相同的条件（扫描的层厚、造影剂的使用等）下，用药前6周期，每2周期对基线时发现的病灶检查一次（骨扫描在怀疑有骨进展或进行CR确认时进行）之后每3周期进行一次；如怀疑新发病灶可适时检查。首次PR/CR，须在4~6周后进行确认。影像学检查时间表允许的窗口期为±7天。当怀疑疾病进展（如症状恶化）可进行计划外的影像学检查。

[18] [redacted]

[19] 基线期、2周期末、疾病进展时进行HER2-PET和FDG-PET检查（组长单位及有条件的参与单位）。

[20] 除了由于影像学证实的疾病进展外，因其它原因结束试验治疗的受试者，若试验结束前4周内未进行影像学评价，须在治疗结束时进行影像学评价，同时，在结束试验后继续按照方案规定的随访频率随访肿瘤疗效，直至有记录证实疾病进展或开始新的肿瘤治疗。

[21] 生存随访：试验治疗终止后，每3个月可通过临床随访或者电话随访收集生存状态和后续抗肿瘤治疗情况，直至死亡。

[22] PK采集：Ib期收集第1周期第21天给药后1小时、3小时及24小时的血浆。II期收集8-10人第1周期第21天吡咯替尼给药前0.5h内、给药后2h±5min、4h±10min、6h±10min、12h±10min、24h±0.5h血浆，第3、第5、第8、第12周期第22天吡咯替尼给药前0.5h内，PK采血前3天服药时间相对固定。

[23] 生物标志物采集/收集：基线期、2周期末、疾病进展/开始新的抗肿瘤治疗前，采集[redacted]及ctDNA血样；收集已有的石蜡包埋肿瘤组织样本或白片≥10张，尽量获取转移灶穿刺标本。

缩略语表

缩略语	中文
ALT	丙氨酸氨基转移酶
AST	天门冬氨酸氨基转移酶
Cr	肌酐
CR	完全缓解
CRF	病例报告表
CDK	细胞周期蛋白依赖性激酶
CYP	细胞色素
bid	每日两次
BUN	尿素氮
dL	分升
EC	伦理委员会
ECG	心电图
ECOG	东部肿瘤协作组
EGFR	表皮生长因子受体
ER	雌激素受体
g	克/离心力
GCP	临床试验规范
GGT	谷氨酰转移酶
h	小时
Hb	血红蛋白
HER2	人表皮生长因子受体-2
HR	激素受体
IB	研究者手册
ISH	原位杂交技术
INR	国际标准化值
IU	国际单位
IV	静脉注射（滴注）
kg	千克

缩略语	中文
kPa	千帕
LDH	乳酸脱氢酶
m	米
min	分钟
mg	毫克
mL	毫升
mm	毫米
MBC	转移性乳腺癌
MTD	最大耐受药物剂量
NCI-CTC	国立癌症研究所通用毒性标准
ORR	客观有效率
PDX	人源肿瘤异种移植模型
PFS	无进展生存期
PLT	血小板
PR	部分缓解
qd	每日一次
RBC	红细胞计数
SAE	严重不良事件
SAP	统计分析计划
TBIL	总胆红素
UNL	正常值上限
WBC	白细胞计数

1 研究背景

在世界范围内，乳腺癌成为女性最常见的恶性肿瘤，其约占全部女性恶性肿瘤发病的 25%[1]。早期乳腺癌可治愈，但转移性乳腺癌（Metastatic Breast Cance，MBC）患者的中位总生存期（overall survival, OS）仅为 2 至 3 年[2]。研究表明，乳腺癌是一类分子水平上具有高度异质性的疾病，不同分子亚型乳腺癌的治疗疗效和生存存在显著差异[3]。乳腺癌分型对治疗方案的选择及预后起指导作用。第 12 届 St Gallen 会议专家组将乳腺癌分为 Luminal A 型、Luminal B 型、HER2 型、三阴性四种亚型[3]。 其中 Luminal B 型根据 HER2 是否阳性又可分为 Luminal B (HER2 阴性)及 Luminal B (HER2 阳性)型。研究表明内分泌联合抗 HER2 的靶向治疗是治疗 Luminal B (HER2 阳性型)MBC 患者的一种有效选择[3]。

表皮生长因子受体（epithelial growth factor receptor，EGFR）是一类具有酪氨酸激酶活性的跨膜受体，该家族包括 HER1（erbB1，EGFR）、HER2（erbB2，NEU）、HER3（erbB3）及 HER4（erbB4）。其中 HER2 基因在 30%以上的人类肿瘤中有扩增/过表达，包括乳腺癌、卵巢癌、子宫内膜癌等等[4]。在乳腺癌的临床诊治过程中 HER2 是有别于肿瘤大小、淋巴结及激素受体外的重要预后因子，也是乳腺癌复发和生存期的独立预后因子[5]。20%~30%的原发性浸润性乳腺癌有 HER2 基因的扩增/过表达，靶向 HER2 分子的药物显著延长 HER2 阳性患者的生存期[5]。1998 年，罗氏公司曲妥珠单抗（Trastuzumab，Herceptin）在美国上市，HER2 阳性患者的临床缓解率及生存期都有了较大的改善[6]；十数年后罗氏公司开发的帕妥珠单抗（Pertuzumab，Perjeta）及 T -DM1（ado-trastuzumab emtansine）分别于 2012 年和 2013 年在美国被批准上市，用于曲妥珠单抗耐药后的二三线治疗。在 HER2 抗体大分子的研发快速进展的同时，葛兰素史克（GlaxoSmithKline）公司研发的拉帕替尼（Lapatinib，TYKERB），作为靶向 HER2 的首个小分子抑制剂，于 2007 年在美国上市，并于 2013 年在中国上市，与卡培他滨（Capecitabine）联用于 HER2 阳性晚期乳腺癌的二线治疗[6]。同时，由 Puma 生物科技公司研发的用于乳腺癌治疗的 EGFR/HER2 小分子抑制剂 Neratinib（Nerlynx），2017 年被批准上市。

马来酸吡咯替尼片（Pyrotinib）是由江苏恒瑞医药股份有限公司自主研发药物，是一个不可逆性针对 EGFR、HER2 双靶点酪氨酸激酶抑制剂。一项对于 HER2 阳性的晚期或转移性乳腺癌 II 期临床试验数据显示，对于经蒽环类和紫杉类药物治疗失败复发/转移后且化疗不超过 2 线的患者，马来酸吡咯替尼片联合卡培他滨片组的较甲苯磺酸

拉帕替尼片联合卡培他滨片组显著提高客观缓解率（78.5% & 57.1%），显著延长无进展生存期（18.1 月 & 7.0 月），使得患者的疾病进展或死亡的风险下降了 63.7%（HR=0.363），且耐受性良好。基于目前 II 期临床试验获得的疗效和安全性数据，国家食品药品监督管理总局同意受理恒瑞医药递交的马来酸吡咯替尼片有条件上市的申请。

细胞周期蛋白依赖性激酶（cyclin denpendent kinase，CDK）为细胞周期调节的关键激酶，参与细胞增殖、存活等生理过程。在细胞增殖过程中，CDK4/6 与细胞 cyclin D 形成的复合物能够磷酸化视网膜母细胞瘤蛋白（Rb）[7]。Rb 磷酸化后，可释放其在未被磷酸化状态下紧密结合的转录因子 E2F，E2F 的激活进一步转录推动细胞周期通过 R 点并由生长期（G1 期）向 DNA 复制期（S1 期）转变，进入了细胞增殖期。CDK4/6 抑制剂可将细胞增殖阻滞于 G1 期，从而达到抑制肿瘤增殖的目的 [8]。目前全球已经有三个 CDK4/6 抑制剂上市，辉瑞公司研发的 Palbociclib、诺华公司研发的 Ribociclib，和礼来公司研发的 Abemaciclib，用于治疗 HR（hormone-receptor [HR]）阳性（ER+和/或 PR+），HER2 阴性的晚期或者转移性乳腺癌患者。

Palbociclib 联合来曲唑一线治疗晚期 ER+/HER2 阴性乳腺癌的双盲、随机对照的 III 期 PALOMA-2 研究，共纳入 666 例患者，主要研究终点表明 Palbociclib 联合来曲唑组较来曲唑对照组显著延长中位无进展生存时间(progression-free survival,PFS)（24.8 月 & 14.5 月），实现了晚期乳腺癌一线治疗 PFS 超过 2 年的突破。最常见的 3 级或 4 级不良事件是中性粒细胞减少，白细胞减少，贫血和疲劳 [9]。Ribociclib 联合来曲唑对比安慰剂联合来曲唑用于一线治疗 HR 阳性、HER2 阴性复发或转移性的绝经后乳腺癌患者的疗效和安全性 III 期随机对照试验，共纳入 668 例患者， Ribociclib 联合来曲唑较安慰剂组显著延长 PFS（HR，0.56; 95％CI，0.43-0.72）、无进展生存率（63.0％ & 42.2％），总体反应率分别为 52.7％和 37.1％（P <0.001）。两组中超过 10％的患者报告的常见 3 级或 4 级不良事件为中性粒细胞减少症和白细胞减少；由于不良事件导致的停药率分别为 7.5％和 2.1％[10]。Abemaciclib 或安慰剂联合非甾体芳香酶抑制剂用于一线治疗 HR 阳性、HER2 阴性绝经后晚期乳腺癌的双盲、随机 III 期的 MONARCH-3 研究中，共纳入 493 名晚期乳腺癌，Abemaciclib 组的中位 PFS 显着延长（Abemaciclib 组未达到，安慰剂组为 14.7 月）。Abemaciclib 组较安慰剂组的客观缓解率显著提高（59％ & 44％）。Abemaciclib 组，最常见的不良反应为腹泻，余常见的 3 级或 4 级不良事件是中性粒细胞减少、白细胞减少 [11]。综上，目前已经上市的 CDK4/6 抑制（Palbociclib、Ribociclib

及 Abemaciclib）联合非甾体芳香酶抑制剂显著改善无进展生存期和客观反应率，在 HR 阳性、HER2 阴性晚期乳腺癌患者中具有可耐受的安全性。

达尔西利（SHR6390）是江苏恒瑞医药股份有限公司研发的口服、高效、选择性的小分子 CDK4/6 抑制剂。[redacted]

Shom Goel 等人研究表明，CDK4/6 抑制剂可通过增加肿瘤细胞抗原的表达以及抑制免疫抑制调节性 T 细胞的增殖从而激活抗肿瘤免疫功能 [12]。另研究表明，CDK4 /6 抑制剂不仅阻断 Rb 磷酸化，而且还减少 TSC2 磷酸化并因此部分减弱 mTORC1 活性，从而减弱对上游 EGFR 家族激酶的抑制，使肿瘤对 EGFR / HER2 抑制剂敏感。 因此，对 EGFR/HER2 和 CDK4/6 的双重抑制引起更有效的抑制 TSC2 磷酸化，从而抑制 mTORC1 / S6K / S6RP 通路的活性。在多种 PDX 模型中 CDK4/6 抑制剂对于 HER2 靶向治疗具有增敏作用，并显著延缓 HER2 阳性乳腺癌模型中的肿瘤复发 [13]。

目前北京大学肿瘤医院研究者发起 SHR 6390 联合吡咯替尼治疗 HER 2 阳性晚期胃癌的 Id 期试验（NCT03480256）；另外国内及国际开展针对激素受体阳性、HER2 阳性晚期乳腺癌的 CDK4/6 抑制剂联合抗 HER2 小分子抑制剂或抗体的多项研究，如 Tucatinib 联合 Palbociclib 及曲唑治疗激素受体阳性和 HER2 阳性转移性乳腺癌的安全性和疗效单臂开放 Ib/II 期临床试验（NCT03054363）；Palbociclib 联合曲妥珠单抗、帕妥珠单抗和氟维司群用于 ER 阳性 HER2 阳性浸润性乳腺癌新辅助治疗的 II 期 NA-PHER2 研究（NCT02530424）；Palbociclib 联合曲妥珠单抗，加或不加来曲唑治疗绝经后局部晚期或转移性 ER 阳性 HER2 阳性乳腺癌晚期乳腺癌治疗的 II 期 PATRICIA 研究（NCT02448420）； Abemaciclib 联合曲妥珠单抗和氟维司群，Abemaciclib 联合曲妥珠单抗，对比曲妥珠单抗联合标准化疗，针对 ER 阳性 HER2 阳性晚期乳腺癌治疗的 II 期 MonarcHER 研究（NCT02675231）。

综上，基于以上临床试验及理论基础，CDK4/6 抑制剂既可以增加内分泌治疗的疗效，也可以增强靶向 HER2 的抗肿瘤效应。复旦大学附属肿瘤医院开展了关于来曲唑、吡咯替尼联合 CDK4/6 抑制剂 SHR6390 治疗激素受体阳性、HER2 阳性晚期乳腺癌的 Ib 期临床研究。Ib 期研究的耐受性数据及初步的有效性数据如下：

1) 来曲唑 2.5 mg/d、吡咯替尼 400 mg/d 联合 SHR6390 125mg/d 剂量组：入组 5 例，2 例 DLT（III 级口腔黏膜炎），最佳疗效 ORR60%;
2) 来曲唑 2.5 mg/d、吡咯替尼 400 mg/d 联合 SHR6390 100mg/d 剂量组：入组 6 例，1 例 DLT（III 级口腔黏膜炎），最佳疗效 ORR 50%;

3) 来曲唑 2.5 mg/d、吡咯替尼 320 mg/d 联合 SHR6390 125mg/d 剂量组：入组 4 例，无 DLT；最佳疗效 4 例 PR，最佳疗效 ORR 100%。

根据 Ib 期研究的耐受性和安全性数据、并结合初步的有效性数据，推荐 II 期研究联剂量为：吡咯替尼 320 mg/d 及 SHR6390 125mg/d。后续拟在此基础上开展关于吡咯替尼、CDK4/6 抑制剂达尔西利（SHR6390）联合内分泌在治疗雌激素受体阳性、HER2 阳性晚期乳腺癌的多中心 II 期临床研究。

1.1 药品名称

a)【通用名称】：来曲唑片

江苏恒瑞医药股份有限公司的来曲唑片为已上市药物，该药物的理化性质及生物效应信息详见药品说明书。

b)【通用名称】：氟维司群注射液

正大天晴药业集团的氟维司群注射液为已上市药物，该药物的理化性质及生物效应信息详见药品说明书。

c)【通用名称】：吡咯替尼

【汉语拼音】Biluotini

【英文名称】Pyrotinib

【中文化学名称】[redacted]

【英文化学名称】[redacted]

d)【通用名称】：达尔西利

化合物代码: SHR6390

【汉语拼音】 Daerxili

【英文名称】Dalpiciclib

【中文化学名称】[redacted]

化学结构式: 如下:[redacted]

1.2 SHR6390 的药理类型和作用机制

SHR6390 为 CDK4/6 激酶抑制剂，是江苏恒瑞医药股份有限公司开发的[redacted]新药。临床前资料显示 SHR6390 选择性地抑制 CDK4/6 激酶活性，使其与 Cyclin D 组成的复合物不能磷酸化下游 Rb 蛋白，阻止细胞由 G1 期进入 S 期，从而发挥抑制细胞增殖和抗肿瘤的作用。

1.3 SHR6390 的药效学研究

[redacted]

1.4 SHR6390 的毒理学研究

[redacted]

1.5 SHR6390 的药代动力学研究

[redacted]

1.6 吡咯替尼联合 SHR6390 在 ER+、HER2+ 乳腺癌肿瘤模型体内抑瘤作用

[redacted]

2 临床研究

2.1 马来酸吡咯替尼临床研究

马来酸吡咯替尼片是由江苏恒瑞医药股份有限公司自主研制而成的原创药物，是一个不可逆性针对 EGFR、HER2 双靶点酪氨酸激酶抑制剂。其用于 HER2 表达阳性的晚期或转移性乳腺癌 II 期临床试验数据显示在针对治疗经蒽环类和紫杉类药物治疗失败，且复发/转移后化疗不超过 2 线的乳腺癌患者的 II 期临床试验中，马来酸吡咯替尼片联合卡培他滨片（简称“吡咯替尼组”）的客观缓解率为 78.5%，甲苯磺酸拉帕替尼片联合卡培他滨片（简称“拉帕替尼组”）的客观缓解率为 57.1%。吡咯替尼组的中位无进展生存期为 18.1 个月，拉帕替尼组的中位无进展生存期为 7.0 个月。吡咯替尼组的中位无进展生存期比拉帕替尼组显著延长，具有统计学显著意义（P<0.0001）。吡咯替尼组与拉帕替尼组相比，患者的疾病进展或死亡的风险下降了 63.7%（HR=0.363）。而且在患者疗效改善的同时，耐受性良好。基于目前 II 期临床试验获得的疗效和安全性数据，国家食品药品监督管理总局同意受理恒瑞医药递交的马来酸吡咯替尼片有条件上市的申请。随着 PHENIX 和 PHOEBE 两项大型 III 期研究的发布，2020 年吡咯替尼获得国家药监局的完全获批认定，获得完全批准的抗肿瘤创新药。

2.2 SHR6390 临床研究

SHR6390 目前已开展晚期实体瘤 I 期、晚期黑色素瘤 I 期晚期乳腺癌 Ib/II 及III期临床试验。

SHR6390-I-101 是一项在国内正在开展的，评价 SHR6390 对晚期实体瘤患者的耐受性及药代动力学 I 期临床研究，主要研究目的是观察 SHR6390 片单次及多次口服给

药在晚期实体瘤患者中的剂量限制性毒性（DLT）及最大耐受剂量（MTD）；次要目的是观察 SHR6390 片在晚期实体瘤患者中的药代动力学特征、安全性、耐受性并评价 SHR6390 片治疗晚期实体瘤的初步有效性。该研究为单臂、开放、单次及多次给药、剂量递增、I 期临床试验。[redacted]

SHR6390-Ib/II-201 研究是一项在国内正在开展的，SHR6390 联合来曲唑或阿那曲唑或氟维司群在激素受体阳性、HER2 阴性晚期乳腺癌患者中进行的 Ib/II 期临床研究。SHR6390-III-301 研究是一项在国内正在开展的，SHR6390 联合氟维司群对比安慰剂联合氟维司群在既往接受过内分泌治疗后疾病进展的 HR 阳性、HER2 阴性的局部晚期或晚期转移性乳腺癌患者中进行的 III 期临床研究。[redacted]2021 年 3 月 24 日，SHR6390 片被国家药品监督管理局药品审评中心纳入突破性治疗品种名单，拟定适应症为 SHR6390 片联合氟维司群治疗经内分泌治疗后进展的激素受体(HR)阳性、人表皮生长因子受体 2(HER2)阴性的复发或转移性乳腺癌。

SHR6390-III-302 研究是一项在国内正在开展的，SHR6390 联合来曲唑或阿那曲唑对比安慰剂联合曲唑或阿那曲唑在 HR 阳性、HER2 阴性的晚期乳腺癌患者中进行的 III 期临床研究。主要研究目的是评价 SHR6390 联合来曲唑或阿那曲唑治疗晚期乳腺癌的有效性[redacted]

SHR6390-III-303 研究是一项在 HR+/HER2-早期乳腺癌患者中开展的，评估 SHR6390 联合内分泌治疗对比内分泌治疗用于辅助阶段的疗效及安全性的 III 期临床试验，目前已在进行中。

2.3 目前已上市 CDK4/6 抑制剂临床研究

Palbociclib 联合来曲唑一线治疗晚期 ER+/HER2 阴性乳腺癌的双盲、随机对照的 III 期 PALOMA-2 研究，共纳入 666 例患者，主要研究终点表明中位 PFS 可达 24.8 月，而对照组来曲唑为 14.5 月，实现了晚期乳腺癌一线治疗 PFS 超过 2 年的突破。最常见的 3 级或 4 级不良事件是中性粒细胞减少（Palbociclib-来曲唑组中发生率为 66.4％，安慰剂-来曲唑组中为 1.4％），白细胞减少（24.8％&0％），贫血（5.4 ％&1.8％）和疲劳（1.8％&0.5％）[9]。

Ribociclib 联合来曲唑对比安慰剂联合来曲唑用于一线治疗 HR 阳性、HER2 阴性复发或转移性的绝经后乳腺癌患者的疗效和安全性 III 期随机对照试验，共纳入 668 例患者，其中 Ribociclib 联合来曲唑 PFS 显着长于安慰剂组（HR，0.56; 95％CI，0.43-0.72）。中位随访时间为 15.3 个月。随访 18 个月后，Ribociclib 组的无进展生存率为 63.0％（95％

CI，54.6-70.3），安慰剂组为 42.2%（95%CI，34.8-49.5）。总体反应率分别为 52.7% 和 37.1%（P <0.001）。两组中超过 10%的患者报告的常见 3 级或 4 级不良事件为中性粒细胞减少症（Ribociclib 为 59.3%，安慰剂组为 0.9%）和白细胞减少（21.0%&0.6）；由于不良事件导致的停药率分别为 7.5%和 2.1%[10]。

Abemaciclib 或安慰剂联合非甾体芳香酶抑制剂用于一线治疗 HR 阳性、HER2 阴性绝经后晚期乳腺癌的双盲、随机 III 期的 MONARCH-3 研究中，共纳入 493 名晚期乳腺癌， Abemaciclib 组的中位 PFS 显着延长（HR=0.54; 95%CI，0.41-0.72; P = .000021; 中位数：Abemaciclib 组未达到，安慰剂组为 14.7 个月）。Abemaciclib 组的客观缓解率为 59%，安慰剂组为 44%（P = 0.004）。在 Abemaciclib 组，最常见的不良反应为腹泻（81.3%，其中 1 级为 44.6%）。Abemaciclib 对比安慰剂组，最常见的 3 级或 4 级不良事件是中性粒细胞减少（21.1%vs 1.2%），腹泻（9.5%vs 1.2%）和白细胞减少（7.6% vs 0.6%）[11]。

3 研究目的与研究终点

3.1 主要研究目的

第一阶段（Ib期）：

确定非甾体类芳香化酶抑制剂（来曲唑）、马来酸吡咯替尼片联合CDK4/6抑制剂SHR6390治疗激素受体阳性、HER2阳性晚期乳腺癌的安全性和耐受性，结合初步的有效性数据，明确此联合方案的II期推荐给药剂量。

第二阶段（II期）：

评估吡咯替尼、达尔西利（SHR6390）联合内分泌治疗在雌激素受体阳性、HER2阳性晚期乳腺癌的有效性和安全性，进一步探索ctDNA作为生物标志物的价值，以及FDG-PET、HER2-PET评估抗肿瘤疗效的临床应用价值，[redacted]

3.2 主要研究终点

本试验的主要研究终点包括:

第一阶段（I期）

联合方案中SHR6390的剂量限制性毒性（DLT）和最大耐受剂量（MTD），确定II期临床研究推荐给药方案；

各剂量组的不良事件（AE）及严重不良事件（SAE）的发生率及严重程度。

第二阶段（II期）

II期临床研究推荐给药方案的客观有效率（ORR），基于RECIST1.1评价标准。

3.3 次要研究终点

第一阶段（I期）

ORR，基于RECIST1.1。

第二阶段（II期）

AE和SAE的发生率及严重程度。

I/II期试验的共同次要研究终点包括：

- 无进展生存期（PFS）；
- 疾病控制率（DCR）：疗效评价为CR/PR/SD的受试者比例；
- 临床获益率（CBR）：研究过程中出现CR、PR及SD≥24周的受试者比例；
- 缓解持续时间（DoR）；
- 探索性分析分子标志物疗效之间的关系。

4 试验药品

4.1 名称和来源

本项目研究药物来曲唑、吡咯替尼及达尔西利（SHR6390片）：均由江苏恒瑞医药股份有限公司生产并提供。

氟维司群由正大天晴药业集团提供。

4.2 药品剂型和规格

来曲唑片 规格：2.5mg；吡咯替尼片 规格：160mg、80mg；SHR6390片 规格：25mg、125mg；氟维司群注射液 规格：250mg

4.3 保存条件

保存条件：25° C以下密封保存。 有效期：暂定 24 个月。

4.4 使用方法

- 来曲唑，口服，2.5 mg，每天1次，空腹给药，连续给药28天为1个周期。

- 氟维司群：D1（第一周期D15增加一次给药），肌肉注射，28天为一周期；
- 吡咯替尼：口服，320mg，每天1次，早餐后30分钟内口服给药，连续给药28天为1个周期。
- SHR6390：口服，125mg，每日1次，空腹给药（给药期间应至少保证服药前1小时和服药后1小时禁食）。服药以28天为一个给药周期，每周期前3周（D1~21）连续服药，第4周（D22~28）停药。

4.5 试验药物的管理、发放与回收

本试验临床用药的管理、发放和回收由专人负责，研究者必须保证所有试验用药物仅用于参加该临床试验的受试者，其剂量与用法应遵照试验方案，剩余的药品退回，不得把临床用药转交任何非临床试验参加者。

药物分发时须签署药物接收单，双人签字，一式两份。研究结束收回剩余药品及空盒，签署药品回收单。每一份药物的发放及回收均应在专门记录单上及时记录。

5 研究总体设计

本研究为一项单臂、开放、剂量爬坡的Ib期临床研究和单臂、开放、多中心II期临床试验。依据《药品注册管理办法》、《药品临床试验质量管理规范》、《新药(化学药品)临床药代动力学试验指导原则》， 本试验拟在经常规标准治疗无效的或缺乏标准治疗的雌激素受体阳性、HER2阳性晚期乳腺癌患者中开展吡咯替尼、达尔西利（SHR6390）联合内分泌治疗的临床研究。

6 实验设计

6.1 药品的剂量和给药方案

在经常规标准治疗无效的或缺乏标准治疗的激素受体阳性、HER2阳性晚期乳腺癌患者进行联合给药的爬坡试验，开展耐受性观察，并初步观察疗效。每个剂量组最后一名受试者完成连续给药28天且该剂量组DLT观察结果满足剂量向上递增原则时，即可开始高一档剂量组爬坡试验。

第一阶段（Ib期）：
本研究来曲唑为 2.5 mg/d，吡咯替尼给药剂量为 400 mg/d 或 320 mg，SHR6390 设计了 3 个剂量组：

剂量组	SHR6390	吡咯替尼片	病例数
A	125 mg/d	400 mg/d	3~6

B	150 mg/d	400 mg/d	3~6
C（备用组）	100 mg/d	400 mg/d	3~6
D（备用组）	125 mg/d	320 mg/d	3~6
E（备用组）	150 mg/d	320 mg/d	3~6
F（备用组）	100 mg/d	320 mg/d	3~6

试验从 A 组开始，采用 3+3 剂量递增的原则设计方案，递增顺序为 A 组→B 组。如发现 A 组出现≥2 例 DLT 时，则启用 C 组及 D 组，如发现 C 组仍不能耐受，则启动备用 F 组；若 D 组能够耐受，则递增至 E 组，若 D 组不能耐受，则启动 F 组。若 F 组仍不能耐受则由研究 团队将共同分析试验数据判断是否终止试验。每个剂量组受试者给药 1 周期（28 天）结 束后对 DLT 进行评估。三药的具体服用方式见 4.4 使用方法。前 2 周期为核心试验阶段， 第 2 周期末疗效评估为 CR/PR/SD 的受试者，可接受该剂量组联合方案持续给药至受试者 出现疾病进展、不能耐受毒性或患者主动要求退出等情况为止。

第二阶段（II期）：

根据第一阶段确定的联合给药II期推荐剂量：吡咯替尼320 mg/d及 SHR6390 125mg/d联合内分泌治疗，进行第二阶段扩展研究，连续给药至受试者出现疾病进展、不能耐受毒性或患者主动要求退出等情况为止。

研究设计图：

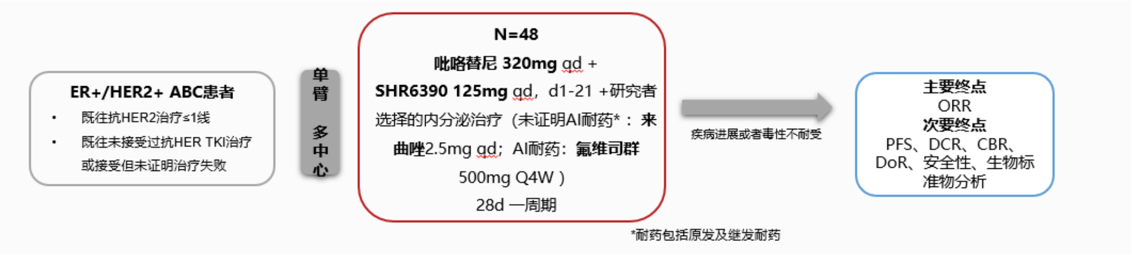

6.2 剂量限制性毒性（DLT）

本试验中 DLT 定义为在第 1 周期内出现的与药物相关或可能相关的以下不良事件（CTC-AE v4.0.3 标准）：

1. 血液学毒性：
   - 4 级中性粒细胞减少持续≥5 天；
   - 4 级血小板减少，或 3 级血小板减少伴临床显著出血；
   - ≥3 级中性粒细胞减少伴发热（≥38.0 摄氏度持续 1 小时或者>38.3 摄氏度）；
   - ≥4 级贫血。
2. 非血液学毒性：

   任何≥3 级非血液学毒性，以下情况除外：

- 3-4 级恶心/呕吐和/或腹泻和/或电解质紊乱，经最佳支持治疗后 72 小时内恢复至≤2 级者；
- 明确与肿瘤相关、与药物无关的 3-4 级的碱性磷酸酶、谷氨酰转肽酶升高。

6.3 最大耐受剂量(MTD)

在第1周期（28天）的给药观察期内，若某一剂量组最终有≥1/3的受试者出现DLT，则前一剂量组为MTD。如爬坡至I期临床试验B剂量组仍未能有1/3受试者出现DLT，则由研究者讨团队论确定是否启用备用的C剂量组，并确定II期试验的推荐剂量。

6.4 受试者替换

对于在DLT观察期内发生非DLT而导致需要退出研究的情况，则增加 1 例受试者作为替补。

DLT观察期内因非计划暂停用药时间超过 4 天，则认为受试者接受药物治疗强度不足，如未观察到DLT，亦需增加1例受试者作为替换。

6.5 样本量计算

根据Ib期研究数据分析，预计II期研究疗效达60%，half-width 0.15，考虑脱落率15%，计算样本量为48例。Ib期RP2D剂量组的4例受试者可纳入II期研究分析，II期计划纳入剩余44例受试者。

7 标本收集

Ib期收集第1周期第21天给药后1小时、3小时及24小时的血浆。II期收集8-10人第1周期第21天吡咯替尼给药前0.5h内、给药后2h±5min、4h±10min、6h±10min、12h±10min、24h±0.5h血浆；第3、第5、第8、第12周期第22天吡咯替尼给药前0.5h内，PK采血前3天服药时间相对固定。采集血液分别用2管肝素锂抗凝采血管各抽取静脉血 3 mL，轻柔颠倒3-5次使血液与抗凝剂充分混合，室温放置。准确记录实际采血时间，详细记录在采血记录表中，采血管在采集后的1小时内转移至离心机中，室温(15 °C -32 °C 下以2000g 离心 10 min，分离血浆， 分装到 2 个冻存管中，一个检测管，一个备份管，检测管血浆量不少于 0.5ml，其余血浆均转移至备份管中，注意不要将下层血细胞吸出，并贴好对应标签，置-80±10° C 低温冰箱保存待测。冻存管在寄送之前需始终保持在低温冰箱中，并每天记录冰箱温度，寄送样品时，需将检测管与备份管分开寄送：先将检测管寄出，备份管仍保存于-80±10℃冰箱内，待试验结束时统一将备份管寄出。

基线期、2周期末、疾病进展/开始新的抗肿瘤治疗前，采集受试者血样（EDTA抗凝管2管和单核细胞分离管1管），送医院组织库(组长单位及有条件的参与单位)及ctDNA血样；收集已有的石蜡包埋肿瘤组织样本或白片≥10张，尽量获取转移灶穿刺标本。

8 受试者的选择

8.1 受试者及研究例数

从伦理学和科学性方面考虑，受试者需既往至多接受过1个针对复发转移性乳腺癌的含曲妥珠单抗方案的系统性治疗；既往未接受过抗HER2 TKI治疗或接受但未证明治疗失败；既往内分泌治疗未证明芳香化酶抑制剂耐药；临床试验前必须签署知情同意书。

第一阶段入组15例；根据第一阶段研究结果，预计第二阶段入组48例。Ib期RP2D剂量组的4例受试者可纳入II期研究分析，II期计划纳入剩余44例受试者。

8.2 入选标准

患者必须满足以下所有入选标准才可入组本试验:

1. 受试者自愿加入本研究，签署知情同意书，有良好的依从性；
2. 年龄≥18 岁且≤75 岁的绝经后或绝经前/围绝经期女性患者，满足以下一条：

a) 既往进行过双侧卵巢切除术，或年龄≥60 岁；或

b) 年龄<60，自然绝经后状态（定义为连续至少 12 个月规律月

经自发性停止且无其他病理或生理原因），E2 和 FSH 在绝经后水平；或

c) 绝经前或围绝经期女性患者也可以入选，但在研究期间必须 愿意接受 LHRH 激动剂治疗；

3. 具有经组织病理确认的复发/转移性乳腺癌患者，且 ER 表达阳性，HER2 表达阳性；
   - HER2 表达阳性指标准免疫组化染色（IHC）检测显示 HER2 为 3+和/或原位杂交技术（ISH）阳性；
   - ER 阳性定义为 ER 表达阳性的细胞百分比≥1%；
   - 局部复发者需经研究者确认无法进行根治性手术切除；
4. 至少具有 1 个符合 RECIST 1.1 标准的颅外可测量病灶；
5. 既往抗肿瘤治疗的规定：
   - 既往至多接受过 1 个针对复发转移性乳腺癌的含曲妥珠单抗方案的系统性治疗【包括抗 HER2 ADC，后续含义相同】

i. 晚期阶段未经含曲妥珠单抗方案治疗，或含曲妥珠单抗方案辅助治疗结束后 1 年以上复发的，后续治疗作为抗 HER2 一线治疗入组；

ii. 一线经过含曲妥珠单抗方案治疗失败，或含曲妥珠单抗方案辅助治疗期间复发或辅助治疗结束后 1 年以内复发的，后续治疗作为抗 HER2 二线治疗入组；

- 既往未接受过抗 HER2 TKI 治疗或接受但未证明治疗失败；
- 既往内分泌治疗未证明芳香化酶抑制剂耐药（耐药定义：辅助芳香化酶抑制剂治疗过程中或结束后 1 年以内复发，复发转移阶段接受过芳香化酶抑制剂并疾病进展），后续内分泌治疗选来曲唑。既往内分泌治疗存在芳香化酶抑制剂耐药，后续内分泌治疗选氟维司群。

6. ECOG 评分：0～1；
7. 预期生存期≥12 周；
8. 重要器官的功能符合下列要求（在入组前 2 周内未使用过任何血液成分及细胞生长因子）：

- 中性粒细胞绝对计数≥1.5×10^9/L；
- 血小板≥90×10^9/L；
- 血红蛋白≥90g/L；
- 总胆红素 TBIL≤1.5 倍 ULN；
- ALT 和 AST ≤2.5 倍 ULN；
- 尿素/尿素氮（BUN）和肌酐（Cr）≤1.5×ULN；
- 左室射血分数（LVEF）≥50%；
- Fridericia 法校正的 QT 间期（QTcF）＜470 毫秒。
- INR≤1.5×ULN，APTT≤1.5×ULN。

8.3 排除标准

具有以下任何一项的患者不能入组本研究:

1. 脑膜转移或活动性脑实质转移。临床稳定脑实质转移患者可以入组，包括未接受过局部治疗的无症状的脑转移；或既往接受过中枢神经系统转移治疗（放疗或手术）的患者，如影像学证实稳定已维持至少 4 周、且已停止对症治疗（含激素和甘露醇等）大于 2 周才可以入组；
2. 既往接受过任何 CDK4/6 抑制剂治疗；
3. 基线期存在伴有临床症状的腹水、胸腔积液、心包积液，需要引流者，或首次

用药前 4 周内进行过浆膜腔积液引流者；

4. 无法吞咽、肠梗阻或存在影响药物服用和吸收的其他因素；
5. 入组前 4 周内接受过化疗、分子靶向治疗或其他临床试验药物等系统治疗；入组前 2 周内接受过内分泌治疗；
6. 受试者既往 5 年内或同时患有其它恶性肿瘤（已治愈的皮肤基底细胞癌和宫颈原位癌除外）；
7. 在首次用药前 4 周内接受过重大手术操作或明显的创伤，或预计患者将要接受重大手术治疗；
8. 妊娠期、哺乳期女性受试者，或有生育能力女性的基线妊娠试验检测阳性，或不愿意采取有效避孕措施的育龄期受试者；
9. 已知对本方案药物组分有过敏史者；
10. 有活动性 HBV、HCV 感染者；经药物治疗后稳定的乙肝（HBV 病毒拷贝数不高于参考值上限）及已治愈的丙肝患者（HCV 病毒拷贝数低于测定法的检测下限）除外；
11. 有免疫缺陷病史，包括 HIV 检测阳性，或患有其他获得性、先天性免疫缺陷疾病，或有器官移植史；
12. 曾患有任何心脏疾病，包括：（1）心绞痛；（2）需药物治疗的或有临床意义的心率失常；（3）心肌梗死；（4）心力衰竭；（5）任何被研究者判断为不适于参加本试验的其他心脏疾病等；筛选期检查发现心脏功能或肾功能异常严重程度≥II 度；
13. 根据研究者的判断，有严重的危害患者安全、或影响患者完成研究的伴随疾病（如：严重的高血压、糖尿病、甲状腺疾病等）；
14. 既往有明确的神经或精神障碍史，包括癫痫或痴呆；
15. 首次用药前 4 周内并发重度感染(如:根据临床诊疗规范需要静脉滴注抗生素、抗真菌或抗病毒药物)，或在筛选期间/首次给药前出现不明原因的发热>38.3°C。

8.4 受试者识别

本试验中所有签署了知情同意书的受试者将获得一个受试者代码，该代码为唯一的编号，如 II-01-01；编号规则为：前 2 位数为试验阶段，如 II；中间2位数为中心编号，依次为01、02、03类推；后 2 位按该中心筛选顺序为 01、02、03 类推。

8.5 脱落标准

所有签署了书面知情同意书并筛选合格进入试验的患者，均有权随时退出临床试验。无论何时何因退出，只要没有完成多次给药第 1 个周期且无法进行安全性评价的受试者，视为脱落病例。

8.6 受试者治疗终止标准

如出现以下一种及一种以上情况，该受试者须退出试验:

1. 受试者撤回知情同意，要求退出；
2. 经影像学检查显示病情进展；
3. 无法耐受毒性者；
4. 严重违背试验方案，研究者评估认为应该终止治疗者；
5. 受试者失访或发生妊娠事件；
6. 研究者认为其他有必要退出研究的情况。

8.7 研究终止标准

本研究终止标准，包括但不限于：

1. 发现对受试者有非预期的、意义重大的或不可接受的风险；
2. 研究药物/试验治疗无效，或继续试验是无意义的；
3. 由于诸如受试者入选严重滞后或重大违背方案等原因，研究者决定终止研究。

9 剂量调整与伴随用药

9.1 DLT 观察期

Ib阶段DLT观察期内，出现非DLT的不良事件时，原则上不予医学处理，以便观察试验药物可能的不良反应及其程度和可逆性。但一旦出现方案中规定的DLT毒性时，须立即停止使用研究药物，并积极处理，在 CRF 上记录使用的药物。

9.2 连续给药第 2 周期及后续周期

药物调整标准（II 期研究全部用药周期调整用药参照此标准）

发生毒性反应后，可由医生根据情况判断，给予相应的处理，具体处理原则如下:

表6

不良事件	严重程度

	I 级	II 级	III 级	IV 级
**血液学毒性**	维持原剂量	维持原剂量	暂停用药，对症处理，恢复至 I 级及以下，根据研究者判断，本周期及后续周期可维持原剂量或下调一个剂量	暂停用药，对症处理，恢复至 I 级及以下，根据研究者判断，本周期及后续周期下调一个剂量
**非血液学毒性**	维持原剂量	维持原剂量或暂停用药，对症处理，恢复至 I 级及以下，根据研究者判断，本周期及后续周期可维持原剂量或下调一个剂量	暂停用药，对症处理，恢复至 I 级及以下，根据研究者判断，本周期及后续周期下调一个剂量	永久终止用药，退出研究
**发热性中性粒细胞减少**	-	-	暂停用药，对症处理，恢复至 I 级及以下，根据研究者判断，本周期及后续周期下调一个剂量	永久终止用药，退出研究

出现明确与研究药物相关的毒性时，由研究者根据临床表现具体处理。待恢复至≤I 级（或研究者判断≤II 级的不良事件对受试者可耐受且无明显的安全风险）后按再次给药。若相同不良事件再次复发，根据研究者判断进行暂停与剂量调整，或要求受试者退出研究；研究者将以最大程度保护受试者安全性。

如因暂停用药后毒性在 2 周内仍无法恢复，原则上受试者应退出研究。暂停给药时间需计入给药周期。

9.3 研究期间不可使用的药物

治疗期间应停用其他抗肿瘤药物和肿瘤治疗相关的辅助性药物，包括抗肿瘤中药、抗肿瘤内分泌治疗药物、免疫治疗或其他抗肿瘤治疗药物等。

9.4 研究期间可酌情使用的药物

受试者如果出现不良反应，应进行密切观察，在必要时予以积极对症治疗，并在CRF表上记录和说明所使用的治疗药物。以上治疗所使用的药物应记录使用时间，使用药物名称，用法与用量。

10 研究步骤

开始研究前，患者必须阅读并且签署目前伦理委员会(EC)批准的知情同意书。所有研究步骤均需在研究计划表所指明的时间窗内进行。

10.1 筛选期

以下筛选步骤在开始研究药物治疗前的 28 天内完成:

- 签署知情同意书
- 采集病史和人口统计学资料，包括患者身份证、通讯地址、联系电话；详细的询问肿瘤病史/其他疾病史：病理结果、ER/PR/HER2检测报告；肿瘤手术、化疗、放疗史以及其他疾病治疗史；除乳腺癌以外肿瘤病史
- 乙肝、丙肝及HIV检查：乙肝五项检查，若检查结果异常，应进行病毒复制（HBV DNA）检测；丙肝病毒抗体（抗HCV）、HIV抗体检查
- 影像学检查：筛选期影像学检查包括胸部、腹部及颅脑的增强CT或MRI，以及研究者怀疑存在病灶的其他部位（如颈部或盆腔）CT/MRI。肿瘤基线评估可放宽至首次给药前4周内，在签署知情同意前获得CT/MRI扫描结果只要符合要求可以用于筛选期肿瘤评估；临床怀疑有骨转移时需进行骨扫描检查（FDG-PET、HER2-PET检查在组长单位及有条件的参与单位进行）
- [redacted]
- 生物标记物收集：[redacted]及ctDNA血样；收集已有的石蜡包埋肿瘤组织样本或白片≥10张，尽量获取转移灶穿刺标本
- 超声心动图：随访LVEF值变化，如出现LVEF下降至＜50%且较基线下降≥10%时，或出现胸痛、心悸等症状时，可增加计划外检查
- 评估当前使用的伴随用药和治疗
- 从签署知情起收集不良事件

以下筛选步骤在开始研究药物治疗前的 7 天内完成:

- 生命体征检查：体温、呼吸、脉搏、血压，血压测量时，测量前 30 分钟内禁止吸烟和饮咖啡，至少安静休息 10 分钟，测量时取坐位，肘部置于与心脏同一水平，每次血压测量均取同侧测量

- 体格检查：主要身体系统的检查（头面部、皮肤系统、淋巴结、眼部、耳鼻喉部、口腔、呼吸系统、心血管系统、腹部、生殖泌尿系统、肌肉骨骼、神经系统和精神状态）
- ECOG评分
- 血常规：血红蛋白、红细胞、白细胞、中性粒细胞计数、淋巴细胞计数和血小板计数
- 尿常规：尿蛋白、尿糖、尿潜血（尿红细胞、白细胞）。如果半定量方法显示蛋白2+，则进行24小时尿蛋白定量检查
- 大便常规：包括大便潜血
- 血生化：总胆红素、结合胆红素、ALT、AST、AKP、γ-GT、LDH、总蛋白、白蛋白、尿素/尿素氮、肌酐、尿酸、空腹血糖、甘油三酯、胆固醇、钾、钠、氯、钙、磷、镁；必要时增加心肌酶谱检查
- 妊娠检查：育龄期女性首次用药前1周内进行血清妊娠检测
- 12导联心电图：若发现心电图异常有临床意义，研究者认为必要时可再次确认

10.2 不良事件的收集/试验期

以下事项应根据试验流程表所列的时间完成（注意：第 1 周期起时间窗为±3 天）。

- ECOG评分、生命体征、体格检查、12 导联心电图：第1周期第15、28天，第2周期第28天（C1D28/C2D1 均可，后同），之后每个周期第 28 天进行；
- 血常规、血生化：第1周期第15、28天，第2周期第28天（C1D28/C2D1 均可，后同），之后每个周期第 28 天进行（受试者如为外地患者，第 2 周期起可在当地医院每周增加一次血常规检查并将结果告知研究者，由研究者判断是否需要返回研究中心进行进一步检查）；
- 尿常规、大便常规：每3周期一次，第1周期以筛选期检查替代，第2周期第1天（C1D28/C2D1 均可，后同），之后每个周期第 28 天进行；
- 超声心动图：每3周期一次，第1周期以筛选期检查替代，第4周期第1天（C3D28/C4D1 均可，后同），之后每3个周期第 28 天进行；治疗结束或受试者退出时（若之前 4 周内未做）；
- 生物标记物收集：基线、2周期末、疾病进展/开始新的抗肿瘤治疗前，采集[redacted]及ctDNA血样。
- 影像学检查：肿瘤影像学检查允许的时间窗为±7天，治疗期影像学检查应在与基线检查相同的条件（扫描的层厚、造影剂的使用等）下，具体评估时间点如下：

- ✧ 用药前 6 周期，第 2 周期末进行首次评估，此后每 2 个周期评估 1 次，6 周期之后每 3 周期进行一次，直至受试者疾病进展、不可耐受的毒性或开始新的肿瘤治疗；
- ✧ 首次评估为 CR、PR 者建议在 4~6 周后进行确认，确认后的肿瘤评估不能改变之前固定的两个周期检查时间点；
- ✧ 当怀疑疾病进展（如症状恶化）可进行计划外的影像学检查；
- ✧ 除了由于影像学证实的疾病进展外，因其它原因结束试验治疗的受试者，若试验结束前 4 周内未进行影像学评价，须在治疗结束时进行影像学评价，同时，在结束试验后继续按照方案规定的随访频率随访肿瘤疗效，直至有记录证实疾病进展或开始新的肿瘤治疗。

- ➢ [redacted]
- ➢ 不良事件：从签署知情同意书起至开始用药前只记录严重不良事件，直至末次用药后 28 天，并且随访直至不良事件消失、缓解至基线水平或≤1 级、稳定。
- ➢ 记录不良反应：自研究治疗第1天开始，直至最后1次治疗后至少28天，或直至所有严重的或药物相关毒性安全恢复至≤ NCI-CTC AE 4.0.3 标准I 度。此外，观察用药期间各种临床表现并予以记录；
- ➢ 记录伴随用药或治疗：自研究治疗前28天开始，直至最后一次治疗后的28天。

注：研究者可根据实际情况，增加受试者访视频率（如每周一次）与检查内容，最大程度保证受试者安全性最大化。

10.3 研究治疗结束/退出研究

研究结束：末例受试者入组后24个月或研究者认为需提前结束试验时。

10.4 治疗结束后随访

- ➢ 疾病进展时间：除了由于影像学证实的疾病进展外，因其它原因结束试验治疗的受试者，若试验结束前4周内未进行影像学，须在治疗结束时进行影像学评价，同时，在结束试验后继续按照方案规定的随访频率随访肿瘤疗效，直至有记录证实疾病进展或开始新的肿瘤治疗。
- ➢ 生存随访：试验治疗终止后，每3个月可通过临床随访或者电话随访收集生存状态和后续抗肿瘤治疗情况，直至死亡。

10.4.1 不良事件严重程度判断标准

参照 NCI-CTC AE 4.0.3 版关于药物不良反应的分级标准。如果出现表中未列出的不良反应可参照下列标准：

- I 级：轻度，无临床症状或有轻微临床症状；仅有临床或实验室检查异常；不需治疗。
- II 级：中度，需要微量的、局部的或非侵害性的治疗；与年龄相符的使用工具的日常生活活动(Activities of Daily Living，ADL)受限，使用工具的日常生活指做饭、购物、打电话、数钱等。
- III 级：病情重或有医学上严重的症状但是暂时不会危及生命；导致住院或住院时间延长；导致残疾；日常生活自理(Self care ADL)受限。日常生活自理指：洗澡、穿衣、脱衣、吃饭、去卫生间、吃药等，非卧床不起。
- IV 级：危及生命，需要紧急治疗。
- V 级：因不良事件致死。

11 安全性评价

11.1 不良事件(AE)

11.1.1 不良事件的定义

不良事件是指临床试验受试者从签署知情同意书后出现的不良医学事件。本试验从受试者接受研究药物治疗开始，直至最后一次使用研究药物后 28 天发生的任何不良医疗事件，无论与试验药物是否有因果关系，均判定为不良事件。

研究人员应详细记录受试者所发生的任何不良事件，包括：不良事件及所有相关症状的描述、发生时间、严重程度、持续时间、采取措施及最终结果和转归。

关注的不良事件

发生毒性反应后，原则上不予以停药或下调剂量，可由医生根据情况判断，给予相应的处理，具体处理原则如下：

- 血液学支持：出现血液学毒性时，研究者可根据临床表现等进行对症处理。血液学毒性达到≥III 度时，需暂停用药，待恢复至≤I 度后按原剂量水平给药。若出现 III 度或 IV 度贫血，可不暂停剂量，根据研究者的判断可进行输血治疗。暂停给药不影响肿瘤评价的时间点。若暂停用药时间超过 14 天，患者需退出研究。
- 腹泻：根据试验方案中“给药暂停和剂量调整”的相关规定，先给予对症治疗，

密切随访或观察（≤ 14 天）。临床建议在腹泻当天开始给予口服蒙脱石散（3 g/袋，3 次/日）或洛哌丁胺。对于仍不能缓解的 III 级腹泻则予药物暂停；待不良事件恢复至 I 级以内后再恢复原剂量给药或降低一个剂量给药。

- 肝功能异常：由研究者根据受试者及不良事件情况给予对症治疗或观察（≤14 天），治疗或观察后仍存在，密切随访，由研究者判断，必要时可予剂量调整和/或增加血生化的检查频率。对于有肝转移的受试者，入组时如 ALT/AST 超出 1.5×UNL，需对肝功能进行密切监控，综合考虑决定该受试者是否适合入组本研究。
- 其他风险：由于研究药物是研究性的，单独用药或与其他药物联合用药时，可能会存在未知的其他风险。所有药物都有过敏反应的潜在风险，如果不及时治疗可能会危及生命。出现了任何一种下述严重过敏反应症状：活动后呼吸困难或面部、口唇、牙龈、舌头或颈部肿胀，应立即获得医疗帮助并联系研究医生。其他过敏反应可能包括皮疹、荨麻疹或水疱。患者尽快报告发生的所有症状和副作用非常重要，不论患者是否认为这些是由研究药物引起的。

本实验导致的不适可能不限于上述事件，但我们会密切随访、积极处理，最大程度保证患者的安全及利益，以上治疗所使用的药物要记录在 CRF 表上。

11.1.2 不良事件与试验药物关系的分类

不良事件包括所有非预期的临床表现，只要这些事件发生于签署知情同意书后，不管是否与试验药物有关系，甚至不管是否应用药物，均应按不良事件报告，所有不良事件必须以临床报告的形式呈报。凡受试病例治疗期间患者主诉的任何不适反应或客观实验室检查指标有异常改变，应如实记录，同时注明不良事件表现严重程度、持续时间、处理措施及转归等，临床医师尚应综合判定不良事件与试验药物关系，不良事件与试验药物关系按5级判定：即肯定有关、可能有关、可能无关、肯定无关、无法评定。前两者计为不良反应，统计不良反应的发生率。

- 肯定有关：反应出现符合用药后合理的时间顺序，反应符合所疑药物已知的反应类型；停药后改善，重复给药再出现该反应。
- 可能有关：反应出现符合用药后合理的时间顺序，反应符合所疑药物已知的反应类型；病人的临床状态或其它治疗方式也可能产生该反应。
- 可能无关：反应出现不太符合用药后合理的时间顺序，反应不太符合所疑药物已知的反应类型；病人的临床状态或其它治疗方式有可能产生该反应。

- 肯定无关：反应出现不符合用药后合理的时间顺序，反应有符合非试验药物已知的反应类型；病人的临床状态或其它治疗方式可能产生该反应，疾病状态改善或停止其它治疗方式反应消除，重复使用其它治疗方法反应出现。
- 无法评定：反应出现与用药后的时间无明确关系，与该药品已知的反应类型相似，同时使用的其它药物也可能引起相同的反应。

11.2 严重不良事件（SAE）

11.2.1 严重不良事件的定义

严重不良事件是指临床试验过程中发生需要住院治疗或延长住院时间、伤残、影响工作能力、危及生命或死亡、导致先天畸形等医学事件。包括以下非预期医学事件：

- 导致死亡的事件；
- 危及生命的事件（定义为受试者在事件发生时有死亡危险）；
- 需要住院治疗或延长住院时间的事件；
- 可导致永久性或严重残疾/功能不全的事件；
- 先天异常或出生缺陷；
- 其他重要医学事件。

11.2.2 妊娠

临床试验期间女性受试者怀孕，则受试者出组，若男性受试者的伴侣怀孕，则受试者继续临床试验，并按照严重不良事件报告。

11.2.3 疾病进展

疾病进展（包括进展的症状和体征）不应作为严重不良事件报告，但如果在试验或安全报告期限内因疾病进展死亡应报告为严重不良事件。因疾病进展的症状和体征而住院不应作为严重不良事件报告。在试验或安全报告期限内，如果癌症的最终结果为死亡，那么导致死亡的事件必须作为严重不良事件报告。

11.2.4 进行其他抗肿瘤治疗

不良事件的记录从签署知情同意书开始至最后一次使用研究药物后 28 天。若 28 天后受试者开始进行其他抗肿瘤治疗，对于非死亡的不良事件，不需要再进行不良事件的记录跟踪。若死亡发生在研究治疗结束后的严重不良事件报告期限内，无论患者是否接受其他治疗，均必须报告。

11.2.5 住院治疗

临床研究中导致住院治疗或住院时间延长的不良事件应视为严重不良事件。任何初次被医疗机构收住院（即使短于 24 小时）的情况均符合此标准。

住院不包括以下情况：

- 康复机构
- 疗养院
- 常规急诊室收治
- 当日手术（如门诊/当日/非卧床的手术）与不良事件恶化无关的住院治疗或住院时间延长本身不是严重不良事件。例如：因原有疾病入院，并没有新的不良事件的发生，也没有原有疾病的加重（如：为了检查试验前至今持续存在的实验室检查异常）；
- 管理原因的住院（如：每年例行的体检）；
- 临床试验期间试验方案规定的住院（如：按试验方案的要求进行操作）；
- 与不良事件恶化无关的择期住院（如：择期整容手术）；
- 已预定的治疗或外科手术应在整个试验方案和/或受试者个人的基线资料中予以记录；
- 仅因为血液品使用而入院。

诊断性或治疗性的侵入性（如手术）、非侵入性操作不应作为不良事件报告。但导致此项操作的疾病状况符合不良事件的定义时，应予以报告，如不良事件报告期间发病的急性阑尾炎应报告为不良事件，而因此进行的阑尾切除术应记录为该不良事件的治疗方法。

12 严重不良事件的报告制度

严重不良事件的报告应自受试者签署知情同意书始，直至最后一次使用研究药物后的 28 个公历日（含 28 天）。试验期间，若发生严重不良事件，无论是首次报告还是随访报告，研究者都必须立即填写《新药临床研究严重不良事件（SAE）报告表》，签名及注明日期，在研究者获知 SAE 的 24 小时内立即通知申办者，并立即停止该受试者试验，采取保护受试者的相应措施。研究者应对严重不良事件追踪至解决。

严重不良事件应详细记录症状、严重程度、发生时间、处理时间、采取措施、随访时间和方式以及转归情况。如果研究者认为某严重不良事件与试验药物无关，而与研究

条件（例如终止原治疗，或试验过程中的合并症）潜在相关，则这种关系应在病历报告表的严重不良事件页的叙述部分详细说明。

13 SAE 的报告程序

临床研究过程中及停药后28天内发生的任何严重不良事件，必须立即书面报告申办者。获得主要疗效指标结果后，在观察次要疗效指标结果的试验期间，发生的SAE应立即上报申办者。同时，研究者必须填写严重不良事件报告表（SAE），对严重不良事件的发生时间、严重程度、与试验药的关系及采取的措施等进行详细说明，并在报告上签名。

14 疗效评价

疗效评价采用RECIST 1.1标准，包括：①评价每一例受试者的疗效，包括完全缓解（CR），部分缓解（PR），病情稳定（SD）以及疾病进展（PD）。② 记录无进展生存期（PFS），指患者开始研究治疗之日至任何有记录的肿瘤进展或任何原因的死亡之间的时间，以先出现者为准。首次被评价为CR、PR的受试者需在4-6周后进行确认。对一般状况的好转或恶化，用治疗前后ECOG评分的变化来表示。

15 研究结束

末例受试者入组后 24 个月或研究者认为需提前结束试验时。

16 安全控制措施

① 特殊处理

- 停止给药。
- 监测生命体征：心电图、血压、呼吸、体温。
- 洗胃：1%～2%氯化钠溶液或 1:5000 高锰酸钾溶液。
- 导泻：硫酸钠 15～30 g，加水 200 mL 给药。
- 灌肠：1%微温肥皂水（约 5000 mL）高位连续清洗。

② 支持疗法

- 镇静，给氧。
- 建立静脉输液通道，开放呼吸通道，必要时予气管插管、心外按摩以及呼吸机辅助呼吸支持。
- 充分补液，维持循环血量：静脉注射生理盐水或葡萄糖氯化钠注射液，根据病

情补充胶体，保证循环渗透压。

- 强心，升压，维持血压稳定，保证重要脏器的血供：可首先予肾上腺皮质激素 20～60 mg 加 5%葡萄糖注射液 50～250 mL 静脉滴注，血压稳定后可给予多巴胺泵入维持。
- 利尿，根据尿量给予利尿药如速尿等，适量给予碳酸氢钠，碱化尿液。
- 维持水电解质、酸碱平衡。
- 抗心律失常。
- 对症治疗，维持氮平衡。

③ 抗过敏

- 扑尔敏 10 mg 给药或异丙嗪 25～50 mg，平卧、吸氧、保证呼吸道通畅。
- 0.1%肾上腺素 0.1～0.2 mL 加 5%葡萄糖静脉滴注。
- 应用糖皮质激素如地塞米松。

④ 预防性应用胃酸抑制剂

⑤ 纠正呼吸循环衰竭

给氧或人工呼吸，用可拉明 0.375 g，洛贝林 3～6 mg 交替注射，每 15～30 分钟 1 次，必要时静注 1～2 次，以及强心甙药物洋地黄制剂等。

17 推荐Ⅱ期临床试验给药方案

根据连续给药研究的耐受性、初步疗效以及药物暴露的结果，综合分析并判断最佳的给药方案为吡咯替尼为320 mg/d、 SHR6390 125mg/d联合内分泌治疗，进入Ⅱ期临床研究。

18 伦理规范及知情同意

18.1 伦理规范

国内相应法规本临床试验必须遵循赫尔辛基宣言（1996 年版）、CFDA 颁布的《药品临床试验管理规范》（GCP）以及相关的法规。

18.2 知情同意

受试者在接受本试验治疗前必须对参加本试验知情同意，以保障受试者的合法权益。研究者向受试者或其指定代表人完整、全面地介绍本研究的目的、药物的作用、可能出现的毒副反应和可能的风险，应让受试者知道他们的权利，所要承担的风险和受益。谈话是十分重要的知情同意过程。如受试者和其合法代表无识字能力，知情同意过程应

有见证人参加，由受试者或其合法代表口头同意后，在知情同意书上签名，见证人的签名应与受试者的签名在同一天。知情同意书应注明版本和制订日期或修改日期。

19 临床试验的质量保证

为了确保本试验能够严格按照临床研究方案进行，在临床试验的整个过程中，临床研究者、申办者双方均应严格按照《药品临床试验质量管理规范》（GCP）的要求进行操作，务必做到试验程序规范，试验数据准确，研究结论可靠。

20 数据处理

20.1 研究者填写数据要求

- 对所有填写了知情同意书并筛选合格进入试验的患者，均须认真而详细地记录病例报告表中的任何项目，不得空项或漏项（无记录的空格划横线）；
- 病例报告表中所有数据需与受试者病历数据核对，保证无误；
- 病例报告表作为原始数据，做任何更正时只能划线，旁注改后数据，并有研究者签名标注日期；
- 化验单复印件粘贴在病例报告表后的化验单粘贴处；
- 对显著偏高或在临床接受范围以外的数据，须加以核实，由研究者做必要说明，

请参照病例报告表填写说明。

20.2 数据的可溯源性、病例报告表（CRF）的填写

最原始记录为研究病历以便妥善保存。病例报告表来自研究病历，由研究者填写，每个入选病例必须完成病例报告表。

20.3 统计分析数据的选择

① 全分析集（Full Analysis Set）

按照意向性分析（ITT）原则，对所有入组并至少使用一次药物的全部病例进行疗效分析。对于未能观察到全部治疗过程的病例资料，用最后一次观察数据结转到试验最终结果（LOCF）。

② 符合方案集（Per-protocol Set）

所有符合试验方案、依从性好、试验期间未用禁止用药、完成病例报告表规定填写内容的病例。对缺失数据不进行任何填补（imputation）。本试验药物的疗效分析对PPS进行。

③ 安全性分析集（Safety Analysis Set）

所有入组病例，至少使用过一次试验用药，并有用药后安全性记录的全部患者，均属于安全性分析集。该数据集用于安全性分析。

20.4 统计分析计划

本试验结果主要采用统计描述方法。计量资料列出均数、标准差、中位数、最大值、最小值，计数资料和等级资料列出频数（构成比）、率、可信区间。

所有统计分析将采用SAS 9.2统计分析软件编程计算。所有的统计学检验均采用双侧检验，P值小于或等于0.05将被认为所检验的差别有统计意义，可信区间采用95%的可信度。

① 患者基本特征

计算年龄、身高、体重等定量资料的均数、标准差、中位数、最大值、最小值，性别、ECOG 评分等定性资料列出频数及百分比。

② 耐受性评价

以描述性统计分析为主，列表描述本次试验各剂量组所发生的不良事件及不良反应（其中不良反应定义为“与研究药物关系为‘肯定有关/很可能有关/可能有关’的不良事件”）。实验室检验结果描述试验前正常但治疗后异常的情况以及发生异常改变时与试验药物的关系。分别计算单次给药各剂量组用药前后生命体征（血压、心率、体温、呼吸频率）及实验室指标的均数、标准差、中位数、最小值、最大值，必要时采用配对t检验前后比较；各剂量组间生命体征及实验室指标的变化进行趋势性检验。多次给药组用药后各时间点均和用药前进行比较。

③ 有效性分析

列表描述本次试验临床疗效。

参考文献

1. Siegel, R.L., Miller, K.D. & Jemal, A. Cancer statistics, 2018. *CA Cancer J Clin* **68**, 7-30 (2018).
2. Cardoso, F., *et al.* 1st International consensus guidelines for advanced breast cancer (ABC 1). *Breast* **21**, 242-252 (2012).
3. Goldhirsch, A., *et al.* Strategies for subtypes--dealing with the diversity of breast cancer: highlights of the St. Gallen International Expert Consensus on the Primary Therapy of Early Breast Cancer 2011. *Ann Oncol* **22**, 1736-1747 (2011).
4. Slamon, D.J., *et al.* Studies of the HER-2/neu proto-oncogene in human breast and ovarian cancer. *Science* **244**, 707-712 (1989).
5. Slamon, D.J., *et al.* Human breast cancer: correlation of relapse and survival with amplification of the HER-2/neu oncogene. *Science* **235**, 177-182 (1987).
6. Ponde, N., Brandao, M., El-Hachem, G., Werbrouck, E. & Piccart, M. Treatment of advanced HER2-positive breast cancer: 2018 and beyond. *Cancer Treat Rev* **67**, 10-20 (2018).
7. Velasco-Velazquez, M.A., *et al.* Examining the role of cyclin D1 in breast cancer. *Future Oncol* **7**, 753-765 (2011).
8. Casimiro, M.C., Velasco-Velazquez, M., Aguirre-Alvarado, C. & Pestell, R.G. Overview of cyclins D1 function in cancer and the CDK inhibitor landscape: past and present. *Expert Opin Investig Drugs* **23**, 295-304 (2014).
9. Finn, R.S., *et al.* Palbociclib and Letrozole in Advanced Breast Cancer. *N Engl J Med* **375**, 1925-1936 (2016).
10. Hortobagyi, G.N., *et al.* Ribociclib as First-Line Therapy for HR-Positive, Advanced Breast Cancer. *N Engl J Med* **375**, 1738-1748 (2016).
11. Goetz, M.P., *et al.* MONARCH 3: Abemaciclib As Initial Therapy for Advanced Breast Cancer. *J Clin Oncol* **35**, 3638-3646 (2017).
12. Goel, S., *et al.* CDK4/6 inhibition triggers anti-tumour immunity. *Nature* **548**, 471-475 (2017).
13. Goel, S., *et al.* Overcoming Therapeutic Resistance in HER2-Positive Breast Cancers with CDK4/6 Inhibitors. *Cancer Cell* **29**, 255-269 (2016).

附件一　身体状况评分标准（ECOG）

（东部肿瘤协作组）

活动评分	描述
0	无症状，完全主动活动，及能够进行无限制的活动。
1	有症状，完全能行走，但重体力活动受限，能从事轻的或以坐为主的工作，如轻微家务、办公室工作。
2	有症状，能行走，生活可自理，但不能进行任何的体力活动，约有 50%以上的时间清醒（白天卧床时间<50%）。
3	有症状，有限的生活自理能力，清醒时间卧床或坐椅>50%，但尚未卧床不起。
4	完全失去功能，生活完全不能自理，卧床不起。
5	死亡。

附件二　肌酐清除率计算

Cockcroft-Gault 公式计算肌酐清除率

血清肌酐浓度（mg/dL）:

$$\text{男性肌酐清除率（mL/min）} = \frac{\text{（140－年龄）×（体重）}^{a}}{72 \times \text{血清肌酐}}$$

$$\text{女性肌酐清除率（mL/min）} = \frac{0.85 \times \text{（140－年龄）×（体重）}^{a}}{72 \times \text{血清肌酐}}$$

血清肌酐浓度（μmol/L）:

$$\text{男性肌酐清除率（mL/min）} = \frac{\text{（140－年龄）×（体重）}^{a}}{0.81 \times \text{血清肌酐}}$$

$$\text{女性肌酐清除率（mL/min）} = \frac{0.85 \times \text{（140－年龄）×（体重）}^{a}}{0.81 \times \text{血清肌酐}}$$

a　年龄单位为岁，体重单位为 KG。

附件三　实体肿瘤的疗效评价标准

实体肿瘤的疗效评价标准 1.1 版（节选）

(New Response Evaluation Criteria in Solid Tumors：Revised RECIST Version 1.1)

说明：本附件为内部翻译资料，仅供参考，实际操作中请以英文版为准。

1　背景

略

2　目的

略

3　肿瘤在基线水平的可测量性

3.1　定义

在基线水平上，肿瘤病灶/淋巴结将按以下定义分为可测量和不可测量两种：

3.1.1　可测量病灶

肿瘤病灶：至少有一条可以精确测量的径线（记录为最大径），其最小长度如下：

- CT 扫描 10 mm（CT 扫描层厚不大于 5mm）
- 临床常规检查仪器 10 mm（肿瘤病灶不能用测径仪器准确测量的应记录为不可测量）
- 胸部 X-射线 20 mm
- 恶性淋巴结：病理学增大且可测量，单个淋巴结 CT 扫描短径须≥15 mm（CT 扫描层厚推荐不超过 5 mm）。基线和随访中，仅测量和随访短径。

3.1.2　不可测量病灶

所有其他病灶，包括小病灶（最长径＜10 mm 或者病理淋巴结短径≥10 mm 至＜15 mm）和无法测量的病灶。无法测量的病灶包括：脑膜疾病、腹水、胸膜或者心包积液、炎性乳腺癌、皮肤/肺的癌性淋巴管炎、影像学不能确诊和随诊的腹部包块，以及囊性病变。

3.1.3　关于病灶测量的特殊考虑

骨病灶、囊性病灶和先前接受过局部治疗的病灶需要特别注明：

骨病灶：

- 骨扫描，PET 扫描或者平片不适合于测量骨病灶，但是可用于确认骨病灶的存

在或者消失；

- 溶骨性病灶或者混合性溶骨/成骨病灶有确定的软组织成分，且软组织成分符合上述可测量性定义时，如果这些病灶可用断层影像技术如 CT 或者 MRI 进行评价，那么这些病灶可以作为可测量病灶；
- 成骨病灶属不可测量病灶。

囊性病灶：

- 符合放射影像学单纯囊肿定义标准的病灶，不应因其为定义上的单纯性囊肿，而认为是恶性病灶，既不属于可测量病灶，也不属于不可测量病灶；
- 若为囊性转移病灶，且符合上述可测量性定义的，可以作为是可测量病灶。但如果在同一病人中存在非囊性病灶，应优先选择非囊性病灶作为靶病灶。

局部治疗过的病灶：

- 位于曾放疗过或经其他局部区域性治疗的部位的病灶，一般作为不可测量病灶，除非该病灶出现明确进展。研究方案应详细描述这些病灶属于可测量病灶的条件。

3.2　测量方法说明

3.2.1　病灶测量

临床评价时，所有肿瘤测量都要以公制米制记录。所有关于肿瘤病灶大小的基线评定都应尽量在接近治疗开始前完成，且必须在治疗开始前的 28 天内（4 周）完成。

3.2.2　评价方法

对病灶基线评估和后续测量应采用同样的技术和方法。除了不能用影像学检查，而仅能用临床检查来评价的病灶之外，所有病灶必须使用影像学检查进行评价。

临床病灶：临床病灶只有位于浅表且测量时直径≥10 mm 时才能认为是可测量病灶（如皮肤结节等）。对于有皮肤病灶的患者，建议用含有标尺测量病灶大小的彩色照片作为存档。当病灶同时使用影像学和临床检查评价时，由于影像学更客观且研究结束时可重复审阅，应尽可能选用影像学评价。

胸部 X 片：当肿瘤进展作为重要研究终点时，应优先使用胸部 CT，因为 CT 比 X 线更敏感，尤其对于新发病灶。胸部 X 片检测仅当被测量病灶边界清晰且肺部通气良好时适用。

CT、MRI：CT 是目前用于疗效评价最好的可用可重复的方法。本指导原则对可测量性的定义建立在 CT 扫描层厚≤5 mm 的基础上。如果 CT 层厚大于 5 mm，可测量病灶最小应为层厚的 2 倍。MRI 在部分情况下也可接受（如全身扫描）。

超声：超声不应作为一种测量方法用于测量病灶大小。超声检查因其操作依赖性，在测量结束后不具备可重复性，不能保证不同测量间技术和测量的同一性。如果在试验期间使用超声发现新病灶，应使用 CT 或者 MRI 进行确认。如果考虑到 CT 的放射线暴露，可以使用 MRI 代替。

内窥镜，腹腔镜检查：不建议使用这些技术用于肿瘤客观评价，但这种方法在取得的活检标本时可以用于确认 CR，也可在研究终点为 CR 后复发或手术切除的试验中，用于确认复发。

肿瘤标志物：肿瘤标志物不能单独用来评价肿瘤客观缓解。但如果标志物水平在基线时超过正常值上限，用于评价完全缓解时必须回到正常水平。因为肿瘤标志物因病而异，在将测量标准写入方案中时需考虑到这个因素。有关 CA-125 缓解（复发性卵巢癌）及 PSA（复发性前列腺癌）缓解的特定标准已经发表。且国际妇科癌症组织已制定了 CA-125 进展标准，即将被加入到卵巢癌一线治疗方案的肿瘤客观评价标准中。

细胞学/组织学技术：在方案规定的特定情况下，这些技术可用于鉴定 PR 和 CR(如生殖细胞肿瘤的病灶中常存在残留的良性肿瘤组织)。当渗出可能是某种疗法潜在的副反应（如使用紫杉烷化合物或血管生成抑制剂的治疗），且可测量肿瘤符合缓解或疾病稳定标准时，在治疗过程中肿瘤相关的渗出出现或加重，可通过细胞学技术来确诊，以区分缓解（或疾病稳定）和疾病进展。

4　肿瘤缓解评估

4.1　全部肿瘤和可测量病灶的评估

为评价客观缓解或未来可能的进展，有必要对所有肿瘤病灶肿瘤的总负荷进行基线评估，为后面的测量结果作参照。在以客观缓解作为主要治疗终点的临床方案中，只有在基线时具有可测量病灶的患者才能入选。可测量病灶定义为存在至少一处可测量的病灶。而对于那些以疾病进展（疾病进展时间或固定日期进展程度）为主要治疗终点的试验，方案入选标准中必须明确是仅限于有可测量病灶的患者，还是没有可测量病灶也可以入选。

4.2　靶病灶和非靶病灶的基线记录

基线评估时有超过一个以上可测量病灶时，应记录并测量所有病灶，总数不超过 5 个（每个器官不超过 2 个），作为靶病灶代表所有累及器官（也就是说只有一个或两个累计器官的患者最多选择两个或四个靶病灶作为基线测量病灶）。

靶病灶必须基于尺寸进行选择（最长直径），能代表所有累及器官，且测量必须具有良好的重复性。有时候当最大的病灶不能重复测量时可重新选择一个可重复测量的最大病灶。

淋巴结因其为正常组织且即使没有肿瘤转移仍可为影像察觉而需要特别关注。定义为可测量结节甚至是靶病灶的病理性淋巴结必须符合以下标准：CT 测量短直径≥15 mm。基线只需要检测短直径。放射学家通常借助结节的短直径来判断该结节是否已有肿瘤转移。结节尺寸一般用影像检测的两维数据来表示（CT 用轴平面，MRI 则从轴面、矢状面或冠状面中选择一个平面）。取最小值即为短直径。例如，一个 20 mm× 30 mm 的腹部结节短直径为 20 mm，可视为恶性的、可测量的结节。在这个例子中，20 mm 即是结节的测量值。直径≥10 mm 但<15 mm 的结节不应该视为靶病灶。而<10 mm 的结节则不属于病理结节范畴，不必予以记录和进一步观察。

所有靶病灶的直径经过计算所求之和（包括非结节病灶的最长直径和结节病灶的短直径）将作为基线直径总和上报。如含有淋巴结直径，如上面提到的，只将短直径计算在内。基线直径总和将作为疾病基线水平的参考数值。

其余所有的病灶包括病理淋巴结可视为非靶病灶，无需进行测量，但应在基线评估时进行记录。如记录为“存在”，“缺失”或极少数情况下“明确进展”。广泛存在的靶病灶可与靶器官记录在一起(如大量扩增骨盆淋巴结或大规模肝转移)。

4.3 缓解标准

4.3.1 靶病灶评估

完全缓解（CR）：所有靶病灶消失，全部病理淋巴结(包括靶结节和非靶结节)短直径必须减少至＜10 mm。

部分缓解（PR）：靶病灶直径之和比基线水平减少至少 30%。

疾病进展（PD）：以整个实验研究过程中所有测量的靶病灶直径之和的最小值为参照，直径和相对增加至少 20%（如果基线测量值最小就以基线值为参照）；除此之外，必须满足直径和的绝对值增加至少 5 mm（出现一个或多个新病灶也视为疾病进展）。

疾病稳定（SD）：靶病灶减小的程度没达到 PR，增加的程度也没达到 PD 水平，介于两者之间，研究时可以直径之和的最小值作为参考。

4.3.2 靶病灶评估的注意事项

淋巴结：即使鉴定为靶病灶的淋巴结减小至 10 mm 以内，每次测量时仍需记录与

基线对应的实际短直径的值（与基线测量时的解剖平面一致）。这意味着如果淋巴结属于靶病灶，即使达到完全缓解的标准，也不能说病灶已全部消失，因为正常淋巴结的短直径就定义为＜10 mm。在 CRF 表或其他的记录方式中需在特定位置专门记录靶淋巴节病灶：对于 CR，所有淋巴节短直径必须＜10 mm；对于 PR、SD 和 PD，靶淋巴节短直径实际测量值将被包含在靶病灶直径的和之中。

小到无法测量的靶病灶：临床研究中，基线记录过的所有病灶（结节或非结节）在后面的评估中都应再次记录实际测量值，即使病灶非常小（如 2 mm）。但有时候可能太小导致 CT 扫描出的图像十分模糊，放射科医生也很难定义出确切的数值，就可能报告为“太小而测量不到”。出现这种情况时，在 CRF 表上记录上一个数值是十分重要的。如果放射科医生认为病灶可能消失了，那也应该记录为 0 mm。如果病灶确实存在但比较模糊，无法给出精确的测量值时，可默认为 5 mm。（注：淋巴结出现这种情况的可能性不大，因其正常情况下一般都具有可测量的尺寸，或者像在腹膜后腔中一样常常为脂肪组织所包绕；但是如果也出现这种无法给出测量值的情况，也默认为 5 mm）。5 mm 的默认值源于 CT 扫描的切割厚度（这个值不因 CT 不同的切割厚度值而改变）。由于同一测量值重复出现的几率不大，提供这个默认值将降低错误评估的风险。但需要重申的是，如果放射医生能给出病灶大小的确切数值，即使病灶直径小于 5 mm，也必须记录实际值。

分离或结合的病灶：当非结节性病灶分裂成碎片状时，将各分离部分的最长径加起来计算病灶的直径之和。同样，对于结合型病灶，通过各结合部分间的平面可将其区分开来，然后计算各自的最大直径。但如果结合得密不可分，最长径应取融合病灶整体的最长径。

4.3.3 非靶病灶的评估

这部分对非靶病灶肿瘤的缓解标准进行了定义。虽然一些非靶病灶实际可测量，但无需测量，只需在方案规定的时间点进行定性评估即可。

完全缓解（CR）：所有非靶病灶消失，且肿瘤标记物恢复至正常水平。所有淋巴结为非病理尺寸（短径＜10 mm）。

非完全缓解/非疾病进展：存在一个或多个非靶病灶和/或持续存在肿瘤标记物水平超出正常水平。

疾病进展：已存在的非靶病灶出现明确进展。注：出现一个或多个新病灶也被视为

疾病进展。

4.3.4 关于的非靶病灶进展评估的特别注意事项

关于非靶病灶进展的定义补充解释如下：当患者存在可测量非靶病灶时，即使靶病灶评估为稳定或部分缓解，要在非靶病灶的基础上作出明确进展的定义，必须满足非靶病灶整体的恶化程度已达到必须终止治疗的程度。而一个或多个非靶病灶尺寸的一般性增大往往不足以达到进展标准，因此，在靶病灶为稳定或部分缓解时，仅依靠非靶病灶的改变就能定义整体肿瘤进展的情况几乎是十分稀少的。

当患者的非靶病灶均不可测量时：在一些Ⅲ期试验中，当入选标准中没有规定必须存在可测量病灶时，就会出现这种情况。整体评估还是参照上文标准，但因为这种情况下没有病灶的可测量数据。非靶病灶的恶化不容易评估（根据定义：必须所有非靶病灶都确实无法测量），因此当非靶病灶改变导致整体疾病负荷增加的程度相当于靶病灶出现疾病进展时，依据非靶病灶作出明确进展的定义，需要建立一种有效的检测方法来进行评估。如描述为肿瘤负荷增加相当于体积额外增加 73%（相当于可测量病灶直径增加 20%）。又比如腹膜渗出从“微量”到“大量”；淋巴管病变从“局部”到“广泛播散”；或在方案中描述为“足够至改变治疗方法”。例子包括胸膜渗出液从痕量到大量，淋巴受累从原发部位向远处扩散，或者在方案中可能被描述为“有必要进行治疗方面的改变”。如果发现有明确的进展，该患者应该在那个时点总体上视为疾病进展。最好具有客观标准可适用于不可测量的病灶的评估，注意，增加的标准必须是可靠的。

4.3.5 新病灶

新的恶性病灶的出现预示着疾病的进展；因此针对新病变的一些评价是非常重要的。目前没有针对影像学检测病灶的具体标准，然而一种新的病灶的发现应该是明确的。比如说，进展不能归因于影像学技术的不同，成像形态的改变，或者肿瘤以外的其它病变（如：一些所谓新的骨病灶仅仅是原病灶的治愈，或原病灶的复发）。当病人的基线病灶出现部分或完全反应时，这一点非常重要的，例如：一例肝脏病灶的坏死可能在 CT 报告上定为新的囊性病变，而其实不是。

在随访中已检测到的而在基线检查中未发现的病灶将视为新的病灶，并提示疾病进展。例如一个在基线检查中发现有内脏病灶的患者，当他做 CT 或 MRI 的头颅检查时发现有转移灶，该患者的颅内转移病灶将被视为疾病进展的依据，即使他在基线检查时并未做头颅检查。

如果一个新的病灶是不明确的，比如因其形态小所致，则需要进一步的治疗和随访评价以确认其是否是一个新的病灶。如果重复的检查证实其是一个新的病灶，那么疾病进展的时间应从其最初的发现的时间算起。

病灶进行 FDG-PET 评估一般需要额外的检测进行补充确认，FDG-PET 检查和补充 CT 检查结果相结合评价进展情况是合理的（尤其是新的可疑疾病）。新的病灶可通过 FDG-PET 检查予明确的，依据以下程序执行：

基线 FDG-PET 检查结果是阴性的，接下来随访的 FDG-PET 检查是阳性的，表明疾病的进展。

没有进行基线的 FDG-PET 检查，后续的 FDG-PET 检查结果是阳性的：

如果随访的 FDG-PET 阳性检查结果发现的新的病变灶与经 CT 检查结果相符，证明是疾病进展。

如果随访的 FDG-PET 的阳性检查结果发现的新的病变灶未能得到 CT 检查结果的确认，需再行 CT 检查予以确认（如果得到确认，疾病进展时间从前期 FDG-PET 检查发现异常算起）。

如果随访的 FDG-PET 的阳性检查结果与经 CT 检查已存在的病灶相符，而该病灶在影像学检测上无进展，则疾病无进展。

4.4　最佳整体疗效评价

最佳整体疗效评价是从试验开始至试验结束的最佳疗效记录，同时要把任何必要条件考虑在内以便确认。有时疗效反应出现在治疗结束后，因此方案应该明确治疗结束后的疗效评价是否考虑在最佳整体疗效评价之内。方案必须明确任何进展前新的治疗如何影响最佳疗效反应。患者的最佳疗效反应主要依赖目标病灶和非目标病灶的结果以及新病灶的表现情况。此外，还依赖于试验性质、方案要求及结果衡量标准。具体来说，在非随机试验中，疗效反应情况是首要目标，PR 或 CR 的疗效确认是必须的，以确认哪个是最佳整体疗效反应。

4.4.1　时间点反应

假设在每个方案的具体时间点上都会有疗效反应发生。表 1 将提供一个基线水平上疾病可测量的患者人群其在每个时间点上的总体疗效反应的总结。

表 1　时间点反应：有靶病灶的受试者（包括或者不包括非靶病灶）

目标病灶	非目标病灶	新病灶	总缓解
CR	CR	非	CR

CR	非 CR/非 PD	非	PR
CR	不能评估	非	PR
PR	非进展或者不能完全评估	非	PR
SD	非进展或者不能完全评估	非	SD
不能完全评估	非进展	非	NE
PD	任何情况	是或否	PD
任何情况	PD	是或否	PD
任何情况	任何情况	是	PD

CR=完全缓解，PR=部分缓解，SD=疾病稳定，PD=疾病进展，NE=不能评估

如果患者无可测量病灶（无目标病灶），评估可参见表 2。

表 2　时间点反应-仅有非目标病灶的受试者

非目标病灶	新病灶	总缓解
CR	非	CR
非 CR 或者非 PD	非	非 CR 或非 PD[a]
不能完全评估	非	不能评估
不能明确的 PD	是或否	PD
任何情况	是	PD

a：对于非目标病灶，“非 CR/非 PD”是指优于 SD 的疗效。由于 SD 越来越多作为评价疗效的终点指标，因而制定非 CR/非 PD 的疗效，以针对未规定无病灶可测量的情况。

4.4.2　评估缺失和不可评价说明

如果在某个特定时间点上无法进行病灶成像或测量，则该患者在该时间点上无法评价。如果在一个评价中只能对部分病灶进行评价，通常这种情况视为在那个时间点无法评价，除非有证据证实缺失的病灶不会影响指定时间点的疗效反应评价。这种情况很可能发生在疾病进展的情况。例如：一个患者在基线水平有 3 个总和为 50 mm 的病灶，但是随后只有 2 个病灶可评价，总和为 80 mm，该患者将被评价为疾病进展，不管缺失的病灶影响有多大。

4.4.3　最佳总缓解：全部时间点

一旦患者的所有资料都具备，其最佳总缓解可以确定。

当研究不需要对完全或部分疗效反应进行确认时最佳总缓解的评估：试验中最佳疗效反应是所有时间点上的最佳反应（例如：一个患者在第一周期疗效评价为 SD，第二周期评价为 PR，最后一周期评价为 PD，但其最佳总缓解评价为 PR。当最佳总缓解评价为 SD 时，其必须满足方案所规定的从基线水平算起的最短时间。如果没有达到最短

时间的标准，即使最佳总缓解评价为 SD 也是不认可的，该患者的最佳总缓解将视随后的评价而定。例如：一个患者第一周期评价为 SD，第二周期为 PD，但其未达到 SD 的最短时间要求，其最佳总缓解评价为 PD。同样的患者在第一周期评价为 SD 后失访将被视为不可评价。

当研究需要对完全或部分疗效反应进行确认时最佳总缓解的评估：只有当每一个受试者符合试验规定的部分或者完全缓解标准而且在方案中特别提及的在随后的时间点（一般是四周后）再次做疗效确认后才能宣称是完全或者部分缓解。在这种情况下，最佳总缓解见表 3 的说明。

表 3　CR 和 PR 疗效需要确认的最佳总缓解

首个时间点总缓解	随后时间点总缓解	最佳总缓解
CR	CR	CR
CR	PR	SD，PD 或 PR[a]
CR	SD	如果 SD 持续足够时间则为 SD，否则应为 PD
CR	PD	如果 SD 持续足够时间则为 SD，否则应为 PD
CR	NE	如果 SD 持续足够时间则为 SD，否则应为 NE
PR	CR	PR
PR	PR	PR
PR	SD	SD
PR	PD	如果 SD 持续足够时间则为 SD，否则应为 PD
PR	NE	如果 SD 持续足够时间则为 SD，否则应为 NE
NE	NE	NE

CR=完全缓解，PR=部分缓解，SD=疾病稳定，PD=疾病进展，NE=不能评估。

a：如果在第一个时间点 CR 真正出现，在随后的时间点出现的任何疾病，那么即便相对于基线该受试者疗效达到 PR 标准，其疗效评价在之后的时间点仍然为 PD（因为在 CR 之后疾病将再次出现）。最佳缓解取决于是否在最短的治疗间隔内出现 SD。然而有时第一次评价为 CR，但随后的时间点扫描提示小病灶似乎依然出现，因而实际上受试者疗效在第一个时间点应该是 PR 而不是 CR。在这种情况下，首次 CR 判断应该被修改为 PR，同时最好的反应是 PR。

4.4.4　疗效评估的特别提示

当结节性病灶被包括在总的靶病灶评估中，同时该结节大小缩小到“正常”大小时（<10 mm），它们依然会有一个病灶大小扫描报告。为了避免过高评估基于结节大小增加所反映的情况，即便是结节正常，测量结果也将被记录。正如前面已经提及的，这就意味着疗效为完全缓解的受试者，CRF 表上也不会记录为 0。

若试验过程中需要进行疗效确认，重复的“不可测量”时间点将使最佳疗效评估变得复杂。试验的分析计划必须说明，在确定疗效时，这些缺失的数据/评估可以被解释清楚。比如，在大部分试验中，可以将某受试者 PR-NE-PR 的反应作为得到了疗效确认。

当受试者出现健康情况整体恶化要求停止给药治疗，但是没有客观证据证明时，应该被报道为症状性进展。即便在治疗终止后也应该尽量去评估客观进展的情况。症状性恶化不是客观反应的评估描述：它是停止治疗的原因。那样的受试者的客观反应情况将通过表 1 到 3 所示的目标和非目标病灶情况进行评估。

定义为早期进展，早期死亡和不可评估的情况是研究特例，且应该在每个方案中进行明确的描述（取决于治疗间期和治疗周期）。

在一些情况下，从正常组织中辨别局部病灶比较困难。当完全缓解的的评估基于这样的定义时，推荐在进行局部病灶完全缓解的疗效评估前进行活检。当一些受试者局部病灶影像学检测结果异常被认为是代表了病灶纤维化或者疤痕形成时，FDG-PET被当作与活检相似的评估标准，用来对完全缓解进行疗效确认。在此种情况下，应该在方案中对FDG-PET的应用进行前瞻性描述，同时以针对此情况专科医学文献的报告作为支持。但是必须意识到的是由于FDG-PET和活检本身的限制性（包括二者的分辨率和敏感性高低），将会导致完全缓解评估时的假阳性结果。

对于不明确的进展发现（如非常小的不确定的新病灶；原有病灶的囊性变或坏死病变）治疗可以持续到下一次评估。如果在下一次评估中，证实了疾病进展，进展日期应该是先前出现疑似进展的日期。

4.5　肿瘤重新评价的频率

治疗期间肿瘤重新评价的频率决定于治疗方案，并应与治疗的类型和日程安排相符。但是在治疗的受益效果不清楚的Ⅱ期试验中，每 6~8 周（时间设计在一个周期的结束点）进行随访是合理的，在特殊方案或情况下可调整时间间隔长度。方案应该具体指明哪些组织部位需要进行基线水平的评估（通常是那些最可能与所研究肿瘤类型的转移病变密切相关的组织部位）和评价重复的频率。正常情况下，靶病灶和非靶病灶在每次评估时都应进行评价，在一些可选择的情形下，某些非目标病灶评价频率可以小一些，例如，目标疾病的疗效评价确认为 CR 或怀疑有骨性病变进展时才需重复骨扫描。

治疗结束后，重新评价肿瘤取决于是否把缓解率或者是到出现某一事件（进展/死亡）的时间作为临床试验终点。如为出现某一事件时间（如：TTP/DFS[1]/PFS）则需要进行方案中规定的常规重复评价。特别是在随机比较试验中，预定的评价应该列在时间表内（如：治疗中的 6~8 周，或治疗后的 3~4 个月），不应受到其他因素的影响，如治疗延迟、给药间隔和任何其他在疾病评价时间选择上可能导致治疗臂不均衡的事件等。

1

4.6 疗效评估/缓解期的确认

4.6.1 确认

对于以疗效为主要研究终点的非随机临床研究，必须对 PR 和 CR 的疗效进行确认，以保证疗效不是评价失误的结果。这也允许在有历史数据的情况下，对结果进行合理的解释，但这些试验的历史数据中的疗效也应进行过确认。但在所有其他情况下，如随机试验（Ⅱ或Ⅲ期）或者以疾病稳定或者疾病进展为主要研究终点的研究中，不再需要疗效确认，因为这对于试验结果的解释没有价值。然而取消疗效确认的要求，就会使防止偏移作用的中心审查显得更加重要，特别是在非盲态实验研究中。

SD 的情况下，在试验开始后的最短时间间隔内（一般不少于 6~8 周），至少有一次测量符合方案中规定的 SD 标准。

4.6.2 总缓解期

总缓解期是从测量首次符合 CR 或 PR（无论哪个先测量到）标准的时间到首次真实记录疾病复发或进展的时间（把试验中记录的最小测量值作为疾病进展的参考）。总完全缓解时间是从测量首次符合 CR 标准的时间到首次真实记录疾病复发或进展的时间。

4.6.3 疾病稳定期

是从治疗开始到疾病进展的时间（在随机化试验中，从随机分组的时间开始），以试验中最小的总和作为参考（如果基线总和最小，则作为 PD 计算的参考）。疾病稳定期的临床相关性因不同研究和不同疾病而不同。如果在某一特定的试验中，以维持最短时间稳定期的病人比例作为研究终点，方案应特别说明 SD 定义中两个测量间的最短时间间隔。

注意：缓解期、稳定期以及 PFS 受基线评价后随访频率的影响。定义标准随访频率不属于本指导原则范围。随访频率应考虑许多因素，如疾病类型和分期、治疗周期及标准规范等。但若需进行试验间的比较，应考虑这些测量终点准确度的限制。

4.7 PFS/TTP

4.7.1 Ⅱ期临床试验

本指导原则主要关注Ⅱ期临床试验中客观缓解作为研究终点的应用。在某些情况下，缓解率可能不是评价新药/新方案潜在抗癌活性的最优选择。在这些情况下，分界时间点上的 PFS/PPF 可认为是提供新药生物活性的原始信号的合适替代指标。但是很

明显，在一个非对照试验中，这些评价会受到质疑，因为貌似有价值的观察可能与病人的筛选等生物学因素有关，而非药物干预的作用。因此，以这些作为研究终点的II期临床试验最好设计随机对照。但某些肿瘤的临床表现始终如一（通常一直状况差），非随机试验也是合理的。但是在这些情况下，因缺少阳性对照，评估预期 PFS 或 PPF[2]时，需小心记录疗效证据。

吡咯替尼、来曲唑联合 CDK4/6 抑制剂 SHR6390 治疗双受体阳性（ER+/HER2+）晚期乳腺癌的多中心 Ib/II 期临床研究

Pyrotinib, LEtrozole And SHR6390 in subjects with dUal-Receptor positive(ER+/HER2+) Advanced Breast cancer: a muLti-center phasE Ib/II study

研究方案

研究方案名称：	PLEASURABLE (LORDSHIPS 3.0/YBCSG-20-01)
研究方案版本号：	1.1
研究方案版本日期：	2021 年 2 月 5 日
临床研究负责人：	胡夕春教授
临床研究协调人：	张剑教授、孟艳春医生、陶中华医生
临床研究组长单位：	复旦大学附属肿瘤医院

目 录

方案摘要 **4**

研究流程图 **13**

缩略语表 **18**

1 研究背景 **20**

1.1 药品名称 23
1.2 SHR6390 的药理类型和作用机制 23
1.3 SHR6390 的药效学研究 23
1.4 SHR6390 的毒理学研究 24
1.5 SHR6390 的药代动力学研究 24
1.6 吡咯替尼联合 SHR6390 在 ER+、HER2+ 乳腺癌肿瘤模型体内抑瘤作用 24

2 临床研究 **24**

2.1 马来酸吡咯替尼临床研究 24
2.2 SHR6390 临床研究 24
2.3 目前已上市 CDK4/6 抑制剂临床研究 25

3 研究目的与研究终点 **25**

3.1 主要研究目的 25
3.2 主要研究终点 26
3.3 次要研究终点 26

4 试验药品 **27**

4.1 名称和来源 27
4.2 药品剂型和规格 27
4.3 保存条件 27
4.4 使用方法 27
4.5 试验药物的管理、发放与回收 27

5 研究总体设计 **27**

6 实验设计 **28**

6.1 药品的剂量和给药方案 28
6.2 剂量限制性毒性（DLT） 28
6.3 最大耐受剂量(MTD) 29
6.4 受试者替换 29
6.5 样本量计算 29

7 标本收集 **30**

8 受试者的选择 **30**

8.1 受试者及研究例数 30
8.2 入选标准 30
8.3 排除标准 32
8.4 受试者识别 33
8.5 脱落标准 33

8.6 受试者治疗终止标准 33
8.7 研究终止标准 33
9 剂量调整与伴随用药 34
9.1 DLT 观察期 34
9.2 连续给药第 2 周期及后续周期 34
9.3 研究期间不可使用的药物 35
9.4 研究期间可酌情使用的药物 35
10 研究步骤 35
10.1 筛选期 35
10.2 不良事件的收集/试验期 36
10.3 研究治疗结束/退出研究 38
10.4 治疗结束后随访 38
11 安全性评价 38
11.1 不良事件(AE) 38
11.2 严重不良事件（SAE） 40
12 严重不良事件的报告制度 42
13 SAE 的报告程序 42
14 疗效评价 42
15 研究结束 42
16 安全控制措施 42
17 推荐Ⅱ期临床试验给药方案 43
18 伦理规范及知情同意 44
18.1 伦理规范 44
18.2 知情同意 44
19 临床试验的质量保证 44
20 数据处理 44
20.1 研究者填写数据要求 44
20.2 数据的可溯源性、病例报告表（CRF）的填写 44
20.3 统计分析数据的选择 45
20.4 统计分析计划 45
附件一 身体状况评分标准（ECOG） 48
附件二 肌酐清除率计算 49
附件三 实体肿瘤的疗效评价标准 50

方案摘要

研究题目	吡咯替尼、来曲唑联合 CDK4/6 抑制剂 SHR6390 治疗双受体阳性（ER+/HER2+）晚期乳腺癌的多中心 Ib/II 期临床研究
研究方案名称	PLEASURABLE (LORDSHIPS 3.0/YBCSG-20-01))
版本号	1.1
版本日期	2021 年 2 月 5 日
组长单位	复旦大学附属肿瘤医院
主要研究者	胡夕春教授
协调研究者	张剑教授、孟艳春医生、陶中华医生
研究对象	ER受体阳性、HER2阳性晚期乳腺癌
研究目的	**第一阶段（Ib 期）：**<br>确定非甾体类芳香化酶抑制剂（来曲唑）、马来酸吡咯替尼片联合 CDK4/6 抑制剂 SHR6390 治疗激素受体阳性、HER2 阳性晚期乳腺癌的安全性和耐受性，结合初步的有效性数据，明确此联合方案的 II 期推荐给药剂量。<br>**第二阶段（II 期）：**<br>评估吡咯替尼、来曲唑联合 CDK4/6 抑制剂 SHR6390 治疗双受体阳性（ER+、HER2+）晚期乳腺癌的有效性和安全性。

<table>
<tr><td>终点指标</td><td>
<table>
<tr><th></th><th>第一阶段（I 期）</th><th>第二阶段（II 期）</th></tr>
<tr><td>主要终点指标</td><td>• 联合方案中 SHR6390 的剂量限制性毒性（DLT）和最大耐受剂量（MTD），确定 II 期临床研究推荐给药方案；
• 各剂量组的不良事件（AE）及严重不良事件（SAE）的发生率及严重程度。</td><td>II 期临床研究推荐给药方案的客观有效率（ORR），基于 RECIST1.1</td></tr>
<tr><td rowspan="2">次要终点指标
•</td><td>ORR，基于 RECIST1.1</td><td>AE 和 SAE 的发生率及严重程度</td></tr>
<tr><td colspan="2">• 无进展生存期（PFS）；
• 疾病控制率（DCR）：疗效评价为 CR/PR/SD 的受试者比例；
• 临床获益率（CBR）：研究过程中出现 CR、PR 及 SD≥24 周的受试者比例；
• 缓解持续时间（DoR）；
• 药代动力学（PK）
• 探索性分析分子标志物与疗效之间的关系。</td></tr>
</table>
</td></tr>
<tr><td>样本量</td><td>第一阶段（Ib 期）：每个剂量组 3~6 例受试者。A、B 组共 6~12 例受试者。如使用备用剂量 C、D、 E、 F 组，每组将增加 3~6 例受试者。
第二阶段（II 期）：抗 HER2 一线治疗：39 例受试者；抗 HER2 二线治疗：28 例受试者。</td></tr>
<tr><td>研究设计</td><td>第一阶段（Ib 期）：单臂、开放、剂量爬坡的 Ib 期临床研究
第二阶段（II 期）：单臂、开放、多中心 II 期临床研究</td></tr>
<tr><td>给药方案</td><td>第一阶段（Ib 期）：
本研究来曲唑给药剂量 2.5 mg/d 或阿那曲唑给药剂量 1 mg/d，吡咯替尼给药剂量为 400 mg/d 或 320 mg/d，SHR6390 设计 3 个剂量组：
<table>
<tr><td>剂量组</td><td>SHR6390</td><td>吡咯替尼片</td><td>病例数</td></tr>
<tr><td>A</td><td>125 mg/d</td><td>400 mg/d</td><td>3~6</td></tr>
<tr><td>B</td><td>150 mg/d</td><td>400 mg/d</td><td>3~6</td></tr>
</table>
</td></tr>
</table>

C（备用组）	100 mg/d	400 mg/d	3~6
D（备用组）	125 mg/d	320 mg/d	3~6
E（备用组）	150 mg/d	320 mg/d	3~6
F（备用组）	100 mg/d	320 mg/d	3~6

非甾体类芳香化酶抑制剂(NSAIs)、马来酸吡咯替尼片联合 SHR6390 方案中，SHR6390 的起始剂量为 125 mg。遵循 3+3 原则，根据起始剂量组是否出现规定频率的 DLT，以 25 mg 为剂量单位进行后续

- 如果起始剂量 A 组 125mg 没有观察到规定频率的 DLT，则剂量 递增至 150mg 组，若该组出现规定频率的 DLT，则 125mg 剂量 被确定为 MTD。
- 如果起始剂量 A 组 125mg 组出现规定频率的 DLT，由研究者讨论同时启动备选 C 组及 D 组，如 C 组仍不能耐受，则启动备 用 F 组。并同时依次启用 D 组→E 组（SHR6390 剂量调整原则 同上），如 D 组仍不能耐受，启动备用 F 组。最后由研究者团 队共同分析试验数据确定联合给药 II 期推荐剂量。
- 若 F 组仍不能耐受则由研究者团队共同分析试验数据决定是否终止试验。
- 根据来曲唑、马来酸吡咯替尼片联合 SHR6390 联合用药组推荐 II 期给药剂量，启用阿那曲唑、马来酸吡咯替尼片联合 SHR6390 联合用药组，若出现不可耐受毒性需要调整用药，由研究者讨论后决定否行药物剂量调整。

每个剂量组受试者给药 1 周期结束后对 DLT 进行评估。

三药的具体服用方式为：

- 非甾体类芳香化酶抑制剂：来曲唑，口服，2.5 mg，口服，1mg，每天 1 次，空腹给药，连续给药 28 天为 1 个周期。
- 吡咯替尼：口服，400 mg 或 320 mg，每天 1 次，早餐后 30 分钟内口服给药，连续给药 28 天为 1 个周期。
- SHR6390：口服，每日 1 次，空腹给药（给药期间应至少保证服

	药前 1 小时和服药后 1 小时禁食）。服药以 28 天为一个给药周期，每周期前 3 周（D1~21）连续服药，与来曲唑同时服用，第 4 周（D22~28）停药。<br>前 2 周期为核心试验阶段，第 2 周期末疗效评估为 CR/PR/SD 的受试者，可接受该剂量组联合方案持续给药至受试者出现疾病进展、不能耐受毒性或主动撤回知情同意书等情况为止。<br>**第二阶段（II 期）：**<br>根据第一阶段确定的联合给药 II 期推荐剂量，进行第二阶段扩组研究，吡咯替尼 320mg、来曲唑 2.5mg 联合 SHR6390 125mg，连续给 药至受试者出现疾病进展、不能耐受毒性或主动撤回知情同意书等情况为止。<br>三药的具体服用方式为：<br>• 非甾体类芳香化酶抑制剂：来曲唑，口服，2.5 mg，每天 1 次，空腹给药，连续给药 28 天为 1 个周期。<br>• 吡咯替尼：口服，320 mg，每天 1 次，早餐后 30 分钟内口服给药，连续给药 28 天为 1 个周期。<br>• SHR6390：口服，125mg，每日 1 次，空腹给药（给药期间应至少保证服药前 1 小时和服药后 1 小时禁食）。服药以 28 天为一个给药周期，每周期前 3 周（D1~21）连续服药，第 4 周（D22~28）停药。
剂量限制性毒性（DLT）	**DLT 定义为在第 1 周期内出现的与药物相关或可能相关的以下不良事件（CTC-AE v4.0.3 标准）：**<br>1. 血液学毒性：<br>● 4 级中性粒细胞减少持续≥5 天；<br>● 4 级血小板减少，或 3 级血小板减少伴临床显著出血；<br>● ≥3 级中性粒细胞减少伴发热（≥38.0 摄氏度持续 1 小时或者>38.3 摄氏度）；<br>● ≥4 级贫血。<br>2. 非血液学毒性：

	任何≥3 级非血液学毒性，以下情况除外：<br>● 3-4 级恶心/呕吐和/或腹泻和/或电解质紊乱，经最佳支持治疗后 72 小时内恢复至≤2 级者；<br>明确与肿瘤相关、与药物无关的 3-4 级的碱性磷酸酶、谷氨酰转肽酶升高。
最大耐受剂量（MTD）	**MTD 定义：**<br>在第 1 周期（28 天）的给药观察期内，若某一剂量组最终有≥1/3 的受试者出现 DLT，则前一剂量组为 MTD。如爬坡至 I 期临床试验 B 剂量组仍未能有 1/3 受试者出现 MTD，则由研究者团队讨论确定是否启用备用的 C 剂量组，并确定 II 期试验的推荐剂量。
入组标准	1. 受试者自愿加入本研究，签署知情同意书，有良好的依从性；<br>2. 年龄 18-75 岁（含界值）的女性患者；<br>3. 具有经组织病理确认的复发/转移性乳腺癌患者，且 ER 表达阳性，HER2 表达阳性；<br>•HER2 表达阳性指标准免疫组化染色（IHC）检测显示 HER2 为 3+和/或原位杂交技术（ISH）阳性；<br>•ER 阳性定义为 ER 表达阳性的细胞百分比≥1%；<br>•局部复发者需经研究者确认无法进行根治性手术切除；<br>4. 至少具有 1 个符合 RECIST 1.1 标准的颅外可测量病灶；<br>5. 自然绝经后妇女，或已接受双侧卵巢切除术或接受卵巢功能抑制的女性；<br>6. 既往抗肿瘤治疗的规定：<br>• 既往至多接受过 1 个针对复发转移性乳腺癌的含曲妥珠单抗方案的系统性治疗【包括抗 HER2 ADC，后续含义相同】<br>i. 晚期阶段未经含曲妥珠单抗方案治疗，或含曲妥珠单抗方案辅助治疗结束后 1 年以上复发的，后续治疗作为抗 HER2 一线治疗入组；<br>ii. 一线经过含曲妥珠单抗方案治疗失败，或含曲妥珠单抗方案辅助治疗期间复发或辅助治疗结束后 1 年以内复发

	的，后续治疗作为抗 HER2 二线治疗入组；<br>• 既往未接受过抗 HER2 TKI 治疗或接受但未证明治疗失败；<br>• 既往内分泌治疗未证明芳香化酶抑制剂耐药（耐药定义：辅助芳香化酶抑制剂治疗过程中或结束后 1 年以内复发，复发转移阶段接受过芳香化酶抑制剂并疾病进展）。<br>7. ECOG 评分：0～1；<br>8. 预期生存期≥12 周；<br>9. 重要器官的功能符合下列要求（在入组前 2 周内未使用过任何血液成分及细胞生长因子）：<br>•中性粒细胞绝对计数≥1.5×10^9/L；<br>•血小板≥90×10^9/L；<br>•血红蛋白≥90g/L；<br>•总胆红素 TBIL≤1.5 倍 ULN；<br>•ALT 和 AST ≤2.5 倍 ULN；<br>•尿素/尿素氮（BUN）和肌酐（Cr）≤1.5×ULN；<br>•左室射血分数（LVEF）≥50%；<br>•Fridericia 法校正的 QT 间期（QTcF）＜470 毫秒。<br>•INR≤1.5×ULN，APTT≤1.5×ULN。
排除标准	1. 受试者有未经治疗的中枢神经系统转移；<br>2. 既往接受过系统性、根治性脑或脑膜转移治疗（放疗或手术），但影像学证实稳定已维持至少 4 周，且已停止全身性激素治疗大于 4 周、无临床症状的患者可以纳入；<br>3. 既往接受过任何 CDK4/6 抑制剂治疗；<br>4. 基线期存在伴有临床症状的腹水、胸腔积液、心包积液，需要引流者，或首次用药前 4 周内进行过浆膜腔积液引流者；<br>5. 无法吞咽、肠梗阻或存在影响药物服用和吸收的其他因素；<br>6. 入组前 4 周内接受过化疗、分子靶向治疗或其他临床试验药物等系统治疗；入组前 2 周内接受过内分泌治疗；<br>7. 受试者既往 5 年内或同时患有其它恶性肿瘤（已治愈的皮肤基底

	细胞癌和宫颈原位癌除外）；<br>8. 在首次用药前 4 周内接受过重大手术操作或明显的创伤，或预计患者将要接受重大手术治疗；<br>9. 妊娠期、哺乳期女性受试者，或有生育能力女性的基线妊娠试验检测阳性，或不愿意采取有效避孕措施的育龄期受试者；<br>10. 已知对本方案药物组分有过敏史者；<br>11. 有活动性 HBV、HCV 感染者；经药物治疗后稳定的乙肝（HBV 病毒拷贝数不高于参考值上限）及已治愈的丙肝患者（HCV 病毒拷贝数低于测定法的检测下限）除外；<br>12. 有免疫缺陷病史，包括 HIV 检测阳性，或患有其他获得性、先天性免疫缺陷疾病，或有器官移植史；<br>13. 曾患有任何心脏疾病，包括：（1）心绞痛；（2）需药物治疗的或有临床意义的心率失常；（3）心肌梗死；（4）心力衰竭；（5）任何被研究者判断为不适于参加本试验的其他心脏疾病等；筛选期检查发现心脏功能或肾功能异常严重程度≥II 度；<br>14. 根据研究者的判断，有严重的危害患者安全、或影响患者完成研究的伴随疾病（如：严重的高血压、糖尿病、甲状腺疾病等）；<br>15. 既往有明确的神经或精神障碍史，包括癫痫或痴呆；<br>16. 首次用药前 4 周内并发重度感染（如：根据临床诊疗规范需要静脉滴注抗生素、抗真菌或抗病毒药物），或在筛选期间/首次给药前出现不明原因的发热>38.3°C。
受试者治疗终止标准	如出现以下一种及以上情况，该受试者须退出/终止治疗<br>1. 受试者撤回知情同意，要求退出；<br>2. 经影像学检查显示病情进展；<br>3. 无法耐受毒性者；<br>4. 严重违背试验方案，研究者评估认为应该终止治疗者；<br>5. 受试者失访或发生妊娠事件；<br>6. 研究者认为其他有必要退出研究的情况。

研究终止标准	本研究终止标准，包括但不限于：<br>1. 发现对受试者有非预期的、意义重大的或不可接受的风险；<br>2. 研究药物/试验治疗无效，或继续试验是无意义的；<br>3. 由于诸如受试者入选严重滞后或重大违背方案等原因，研究者决定终止研究。
安全性评价指标	依据 CTCAE v4.0.3 标准判断不良事件严重程度。试验期间应如实填写不良事件记录表，包括不良事件的发生时间、严重程度、持续时间、采取的措施和转归等。
疗效评价	入组受试者前 6 周期每 2 周期末（±7 天）进行 1 次影像学评价，6 周期之后每 3 周期末（±7 天）进行 1 次影像学评价，直至疾病进展或开始新的抗肿瘤治疗。根据 RECIST 1.1 标准进行肿瘤疗效评估。疾病进展或开始新的抗肿瘤治疗后每 12 周随访一次生存状态。
II 期探索性研究（仅组长单位或有条件的参与单位）	1.患者基线、2 周期、进展后加做 FDG-PET、HER2-PET 检测，进一步探索其评估抗肿瘤疗效的临床应用价值<br>2.[redacted]<br>3. Circulating tumor DNA (ctDNA)<br>ctDNA 将在基线、周期 2 结束时、疾病进展或开始新的抗肿瘤治疗之前收集，作为评估抗肿瘤疗效的生物标志物。
统计方法	本试验结果主要采用统计描述方法。计量资料列出均数、标准差、中位数、最大值、最小值，计数资料和等级资料列出频数（构成比）、率、可信区间。<br>所有统计分析将采用 SAS 9.2 或以上版本统计分析软件编程计算。<br>**安全性分析：**<br>以描述性统计分析为主，对各剂量组所发生的不良事件、严重不良事件及与研究药物相关的不良事件等进行分析。实验室检验结果描述试验前正常但治疗后异常的情况。<br>**有效性分析：**<br>对客观缓解率（ORR）、疾病控制率（DCR）、临床获益率（CBR）

	等疗效终点进行点估计并提供其代表总体的 95%置信区间。生存率采用 Kaplan-Meier 法评估中位无进展生存时间、12 个月生存率以及其代表总体的 95%置信区间，并绘制生存图。对其它次要疗效指标均采用描述性分析。<br>**其他分析：**<br>对于可能影响疗效的分子标志物的水平与疗效之间的关系。
**研究结束**	末例受试者入组后 24 个月或研究者认为需提前结束试验时。
**研究时间**	预计 2018 年 11 月~2022 年 11 月

研究流程图

项目	筛选期		治疗期（28 天为一个治疗周期）				治疗后		生存随访
			第 1 周期			≥2 周期			
	第-28 天至第-1 天	第-7 天至第-1 天	第 15 天（±3d）	第 21 天（±3d）	第 28 天（±3d）	第 28 天（±3d）	治疗结束/退出	治疗结束后访视末次给药后 4 周	每 12 周（±7d）
**基线资料**									
签署知情同意	×								
人口学资料	×								
肿瘤病史/其他病史[1]	×								
伴随用药[2]	×		×						
**实验室检查**									
血常规[3]		×	×		×	×	如 7 天内未做	必要时	
尿常规[4]		×	每 3 周期一次				如 7 天内未做	必要时	
大便常规[5]		×	每 3 周期一次				如 7 天内未做	必要时	
血生化[6]		×	×		×	×	如 7 天内未做	必要时	
乙肝、丙肝及 HIV 检查[7]	×								
妊娠试验[8]		×						必要时	
**临床评估、检查**									
不良事件[9]	**自签署知情同意书至末次用药后 28 天**								
生命体征[10]		×	×		×	×	如 7 天内未做	×	

项目	筛选期		治疗期（28 天为一个治疗周期）				治疗后		生存随访
			第 1 周期			≥2 周期			
	第-28 天至第-1 天	第-7 天至第-1 天	第 15 天（±3d）	第 21 天（±3d）	第 28 天（±3d）	第 28 天（±3d）	治疗结束/退出	治疗结束后访视末次给药后 4 周	每 12 周（±7d）
体格检查[11]		×	×		×	×	如 7 天内未做	×	
ECOG 评分		×	×		×	×	如 7 天内未做	×	
心电图[12]		×	×		×	×	如 7 天内未做	必要时	
超声心动图[13]	×		每 3 周期一次				如 4 周内未做	必要时	
研究药物									
来曲唑给药[14]			**每日一次，空腹口服**						
吡咯替尼给药[15]			**每日一次，餐后 30 分钟内口服**						
SHR6390 给药[16]			**每日一次，空腹口服**						
疗效评估									
影像学检查[17]	×		**每 2 周期末（±7 天）进行 1 次影像学评价，6 周期后每 3 周期末（±7 天）进行 1 次影像学评价，直至疾病进展或开始新的抗肿瘤治疗**						
[redacted][18]	×		[redacted]						
HER2-PET[19]	×		**第 2 周期治疗后（±7 天）及疾病进展后**						
FDG-PET[19]	×		**第 2 周期治疗后（±7 天）及疾病进展后**						
治疗结束后随访									
疾病进展时间[20]							**直至疾病进展或开始新的抗肿瘤治疗**		
生存随访[21]									×
血样采集及肿瘤样本采集/收集									
PK 采集[22]				×					

项目	筛选期		治疗期（28 天为一个治疗周期）				治疗后		生存随访
			第 1 周期			≥2 周期			
	第-28 天至第-1 天	第-7 天至第-1 天	第 15 天（±3d）	第 21 天（±3d）	第 28 天（±3d）	第 28 天（±3d）	治疗结束/退出	治疗结束后访视末次给药后 4 周	每 12 周（±7d）
生物标志物采集/收集[23]	×					×（第 2 周期第 28 天±3 天）	×（疾病进展或开始新的抗肿瘤治疗前）		

备注：

[1] 肿瘤病史/其他疾病史：病理结果、ER/PR/HER2检测报告；肿瘤手术、化疗、放疗史以及其他疾病治疗史；除乳腺癌以外肿瘤病史。

[2] 记录开始用药前28天内以及研究期间的伴随用药和伴随治疗情况。一旦受试者中断试验治疗，仅应记录试验治疗相关的新发或未解决的不良事件所用的伴随用药和伴随治疗

[3] 血常规：血红蛋白、红细胞、白细胞、中性粒细胞计数、淋巴细胞计数和血小板计数。

[4] 尿常规：尿蛋白、尿糖、尿潜血（尿红细胞、白细胞）。如果半定量方法显示蛋白2+，则进行24小时尿蛋白定量检查。

[5] 大便常规：包括大便潜血。

[6] 血生化：总胆红素、结合胆红素、ALT、AST、AKP、γ-GT、LDH、总蛋白、白蛋白、尿素/尿素氮、肌酐、尿酸、空腹血糖、甘油三酯、胆固醇、钾、钠、氯、钙、磷、镁； 必要时增加心肌酶谱检查。

[7] 乙肝、丙肝及HIV检查：乙肝五项检查，若检查结果异常，应进行病毒复制（HBV DNA）检测；丙肝病毒抗体（抗HCV）、HIV抗体检查。

[8] 妊娠检查：育龄期女性首次用药前1周内进行血清妊娠检测。

[9] 不良事件：从签署知情同意开始记录不良事件，直至末次用药后至少28天，并且随访直至不良事件缓解或稳定，如受试者开始新的抗肿瘤治疗，随访至开始肿瘤治疗。

[10] 体征检查：体温、呼吸、脉搏、血压。

[11] 体格检查及体重测量：主要身体系统的检查（头面部、皮肤系统、淋巴结、眼部、耳鼻喉部、口腔、呼吸系统、心血管系统、腹部、生殖泌尿系统、肌肉骨骼、神经系统和精神状态），筛选期及研究结束时记录全面体格检查结果，试验期间仅须记录异常情况。

[12] 12导联心电图：若发现心电图异常有临床意义，研究者认为必要时可再次确认。

[13] 超声心动图：随访LVEF值变化，如出现LVEF下降至＜50%且较基线下降≥10%时，或出现胸痛、心悸等症状时，可增加计划外检查。

[14] 非甾体类芳香化酶抑制剂：来曲唑，口服，2.5 mg，每天 1 次，连续给药 28 天为 1 个周期。

[15] 吡咯替尼：每天1次，320 mg，早餐后30分钟内口服给药，连续给药28天为1个周期。

[16] SHR6390空腹口服，每日1次，125mg，服药3周（D1~21），停药1周（D22~28），28天为1个周期。对于SHR6390空腹服药的要求，早晨空腹口服，温水送服，连续给药期间服药前1小时和服药后1小时禁食。

[17] 影像学检查：筛选期影像学检查包括胸部、腹部的增强CT或MRI，以及研究者怀疑存在病灶的其他部位（如颈部、盆腔或颅脑）CT/MRI。肿瘤基线评估可放宽至首次给药前4周内，在签署知情同意前获得CT/MRI扫描结果只要符合要求可以用于筛选期肿瘤评估；临床怀疑有骨转移时需进行骨扫描检查。治疗期影像学检查应该在与基线检查相同的条件（扫描的层厚、造影剂的使用等）下，用药前6周期，每2周期对基线时发现的病灶检查一次（骨扫描在怀疑有骨进展或进行CR确认时进行）之后每3周期进行一次；如怀疑新发病灶可适时检查。首次PR/CR，须在4~6周后进行确认。影像学检查时间表允许的窗口期为±7天。当怀疑疾病进展（如症状恶化）可进行计划外的影像学检查。

[18] [redacted]

[19] 基线期、2周期末、疾病进展时进行HER2-PET和FDG-PET检查（组长单位及有条件的参与单位）。

[20] 除了由于影像学证实的疾病进展外，因其它原因结束试验治疗的受试者，若试验结束前4周内未进行影像学评价，须在治疗结束时进行影像学评价，同时，在结束试验后继续按照方案规定的随访频率随访肿瘤疗效，直至有记录证实疾病进展或开始新的肿瘤治疗。

[21] 生存随访：试验治疗终止后，每3个月可通过临床随访或者电话随访收集生存状态和后续抗肿瘤治疗情况，直至死亡。

[22] PK采集：Ib期收集第1周期第21天给药后1小时、3小时及24小时的血浆。II期收集8-10人第1周期第21天吡咯替尼给药前0.5h内、给药后2h±5min、4h±10min、6h±10min、12h±10min、24h±0.5h血浆，第3、第5、第8、第12周期第22天吡咯替尼给药前0.5h内，PK采血前3天服药时间相对固定。

[23] 生物标志物采集/收集：基线期、2周期末、疾病进展/开始新的抗肿瘤治疗前，采集[redacted]及ctDNA血样；收集已有的石蜡包埋肿瘤组织样本或

白片≥10张，尽量获取转移灶穿刺标本。

缩略语表

缩略语	中文
ALT	丙氨酸氨基转移酶
AST	天门冬氨酸氨基转移酶
Cr	肌酐
CR	完全缓解
CRF	病例报告表
CDK	细胞周期蛋白依赖性激酶
CYP	细胞色素
bid	每日两次
BUN	尿素氮
dL	分升
EC	伦理委员会
ECG	心电图
ECOG	东部肿瘤协作组
EGFR	表皮生长因子受体
ER	雌激素受体
g	克/离心力
GCP	临床试验规范
GGT	谷氨酰转移酶
h	小时
Hb	血红蛋白
HER2	人表皮生长因子受体-2
HR	激素受体
IB	研究者手册
ISH	原位杂交技术
INR	国际标准化值
IU	国际单位
IV	静脉注射（滴注）
kg	千克

缩略语	中文
kPa	千帕
LDH	乳酸脱氢酶
m	米
min	分钟
mg	毫克
mL	毫升
mm	毫米
MBC	转移性乳腺癌
MTD	最大耐受药物剂量
NCI-CTC	国立癌症研究所通用毒性标准
ORR	客观有效率
PDX	人源肿瘤异种移植模型
PFS	无进展生存期
PLT	血小板
PR	部分缓解
qd	每日一次
RBC	红细胞计数
SAE	严重不良事件
SAP	统计分析计划
TBIL	总胆红素
UNL	正常值上限
WBC	白细胞计数

1 研究背景

在世界范围内，乳腺癌成为女性最常见的恶性肿瘤，其约占全部女性恶性肿瘤发病的 25%[1]。早期乳腺癌可治愈，但转移性乳腺癌（Metastatic Breast Cance，MBC）患者的中位总生存期（overall survival, OS）仅为 2 至 3 年[2]。研究表明，乳腺癌是一类分子水平上具有高度异质性的疾病，不同分子亚型乳腺癌的治疗疗效和生存存在显著差异[3]。乳腺癌分型对治疗方案的选择及预后起指导作用。第 12 届 St Gallen 会议专家组将乳腺癌分为 Luminal A 型、Luminal B 型、HER2 型、三阴性四种亚型[3]。 其中 Luminal B 型根据 HER2 是否阳性又可分为 Luminal B (HER2 阴性)及 Luminal B (HER2 阳性)型。研究表明内分泌联合抗 HER2 的靶向治疗是治疗 Luminal B (HER2 阳性型)MBC 患者的一种有效选择[3]。

表皮生长因子受体（epithelial growth factor receptor，EGFR）是一类具有酪氨酸激酶活性的跨膜受体，该家族包括 HER1（erbB1，EGFR）、HER2（erbB2，NEU）、HER3（erbB3）及 HER4（erbB4）。其中 HER2 基因在 30%以上的人类肿瘤中有扩增/过表达，包括乳腺癌、卵巢癌、子宫内膜癌等等[4]。在乳腺癌的临床诊治过程中 HER2 是有别于肿瘤大小、淋巴结及激素受体外的重要预后因子，也是乳腺癌复发和生存期的独立预后因子[5]。20%~30%的原发性浸润性乳腺癌有 HER2 基因的扩增/过表达，靶向 HER2 分子的药物显著延长 HER2 阳性患者的生存期[5]。1998 年，罗氏公司曲妥珠单抗（Trastuzumab，Herceptin）在美国上市，HER2 阳性患者的临床缓解率及生存期都有了较大的改善[6]；十数年后罗氏公司开发的帕妥珠单抗（Pertuzumab，Perjeta）及 T -DM1（ado-trastuzumab emtansine）分别于 2012 年和 2013 年在美国被批准上市，用于曲妥珠单抗耐药后的二三线治疗。在 HER2 抗体大分子的研发快速进展的同时，葛兰素史克（GlaxoSmithKline）公司研发的拉帕替尼（Lapatinib，TYKERB），作为靶向 HER2 的首个小分子抑制剂，于 2007 年在美国上市，并于 2013 年在中国上市，与卡培他滨（Capecitabine）联用于 HER2 阳性晚期乳腺癌的二线治疗[6]。同时，由 Puma 生物科技公司研发的用于乳腺癌治疗的 EGFR/HER2 小分子抑制剂 Neratinib（Nerlynx），2017 年被批准上市。

马来酸吡咯替尼片（Pyrotinib）是由江苏恒瑞医药股份有限公司自主研发药物，是一个不可逆性针对 EGFR、HER2 双靶点酪氨酸激酶抑制剂。一项对于 HER2 阳性的晚期或转移性乳腺癌 II 期临床试验数据显示，对于经蒽环类和紫杉类药物治疗失败复发/转移后且化疗不超过 2 线的患者，马来酸吡咯替尼片联合卡培他滨片组的较甲苯磺酸

拉帕替尼片联合卡培他滨片组显著提高客观缓解率（78.5% & 57.1%），显著延长无进展生存期（18.1 月 & 7.0 月），使得患者的疾病进展或死亡的风险下降了 63.7%（HR=0.363），且耐受性良好。基于目前 II 期临床试验获得的疗效和安全性数据，国家食品药品监督管理总局同意受理恒瑞医药递交的马来酸吡咯替尼片有条件上市的申请。

细胞周期蛋白依赖性激酶（cyclin denpendent kinase，CDK）为细胞周期调节的关键激酶，参与细胞增殖、存活等生理过程。在细胞增殖过程中，CDK4/6 与细胞 cyclin D 形成的复合物能够磷酸化视网膜母细胞瘤蛋白（Rb）[7]。Rb 磷酸化后，可释放其在未被磷酸化状态下紧密结合的转录因子 E2F，E2F 的激活进一步转录推动细胞周期通过 R 点并由生长期（G1 期）向 DNA 复制期（S1 期）转变，进入了细胞增殖期。CDK4/6 抑制剂可将细胞增殖阻滞于 G1 期，从而达到抑制肿瘤增殖的目的[8]。目前全球已经有三个 CDK4/6 抑制剂上市，辉瑞公司研发的 Palbociclib、诺华公司研发的 Ribociclib，和礼来公司研发的 Abemaciclib，用于治疗 HR（hormone-receptor [HR]）阳性（ER+和/或 PR+），HER2 阴性的晚期或者转移性乳腺癌患者。

Palbociclib 联合来曲唑一线治疗晚期 ER+/HER2 阴性乳腺癌的双盲、随机对照的 III 期 PALOMA-2 研究，共纳入 666 例患者，主要研究终点表明 Palbociclib 联合来曲唑组较来曲唑对照组显著延长中位无进展生存时间(progression-free survival,PFS)（24.8 月 & 14.5 月），实现了晚期乳腺癌一线治疗 PFS 超过 2 年的突破。最常见的 3 级或 4 级不良事件是中性粒细胞减少，白细胞减少，贫血和疲劳[9]。Ribociclib 联合来曲唑对比安慰剂联合来曲唑用于一线治疗 HR 阳性、HER2 阴性复发或转移性的绝经后乳腺癌患者的疗效和安全性 III 期随机对照试验，共纳入 668 例患者， Ribociclib 联合来曲唑较安慰剂组显著延长 PFS（HR，0.56; 95％CI，0.43-0.72）、无进展生存率（63.0％ & 42.2％），总体反应率分别为 52.7％和 37.1％（P <0.001）。两组中超过 10％的患者报告的常见 3 级或 4 级不良事件为中性粒细胞减少症和白细胞减少；由于不良事件导致的停药率分别为 7.5％和 2.1％[10]。Abemaciclib 或安慰剂联合非甾体芳香酶抑制剂用于一线治疗 HR 阳性、HER2 阴性绝经后晚期乳腺癌的双盲、随机 III 期的 MONARCH-3 研究中，共纳入 493 名晚期乳腺癌，Abemaciclib 组的中位 PFS 显着延长（Abemaciclib 组未达到，安慰剂组为 14.7 月）。Abemaciclib 组较安慰剂组的客观缓解率显著提高（59％ & 44％）。Abemaciclib 组，最常见的不良反应为腹泻，余常见的 3 级或 4 级不良事件是中性粒细胞减少、白细胞减少[11]。综上，目前已经上市的 CDK4/6 抑制（Palbociclib、Ribociclib

及 Abemaciclib）联合非甾体芳香酶抑制剂显著改善无进展生存期和客观反应率，在 HR 阳性、HER2 阴性晚期乳腺癌患者中具有可耐受的安全性。

SHR6390 是江苏恒瑞医药股份有限公司研发的口服、高效、选择性的小分子 CDK4/6 抑制剂。[redacted]

Shom Goel 等人研究表明，CDK4/6 抑制剂可通过增加肿瘤细胞抗原的表达以及抑制免疫抑制调节性 T 细胞的增殖从而激活抗肿瘤免疫功能[12]。另研究表明，CDK4 /6 抑制剂不仅阻断 Rb 磷酸化，而且还减少 TSC2 磷酸化并因此部分减弱 mTORC1 活性，从而减弱对上游 EGFR 家族激酶的抑制，使肿瘤对 EGFR / HER2 抑制剂敏感。 因此，对 EGFR/HER2 和 CDK4/6 的双重抑制引起更有效的抑制 TSC2 磷酸化，从而抑制 mTORC1 / S6K / S6RP 通路的活性。在多种 PDX 模型中 CDK4/6 抑制剂对于 HER2 靶向治疗具有增敏作用，并显著延缓 HER2 阳性乳腺癌模型中的肿瘤复发[13]。

目前北京大学肿瘤医院研究者发起 SHR 6390 联合吡咯替尼治疗 HER 2 阳性晚期胃癌的 Id 期试验（NCT03480256）；另外国内及国际开展针对激素受体阳性、HER2 阳性晚期乳腺癌的 CDK4/6 抑制剂联合抗 HER2 小分子抑制剂或抗体的多项研究，如 Tucatinib 联合 Palbociclib 及曲唑治疗激素受体阳性和 HER2 阳性转移性乳腺癌的安全性和疗效单臂开放 Ib/II 期临床试验（NCT03054363）；Palbociclib 联合曲妥珠单抗、帕妥珠单抗和氟维司群用于 ER 阳性 HER2 阳性浸润性乳腺癌新辅助治疗的 II 期 NA-PHER2 研究（NCT02530424）；Palbociclib 联合曲妥珠单抗，加或不加来曲唑治疗绝经后局部晚期或转移性 ER 阳性 HER2 阳性乳腺癌晚期乳腺癌治疗的 II 期 PATRICIA 研究（NCT02448420）； Abemaciclib 联合曲妥珠单抗和氟维司群，Abemaciclib 联合曲妥珠单抗，对比曲妥珠单抗联合标准化疗，针对 ER 阳性 HER2 阳性晚期乳腺癌治疗的 II 期 MonarcHER 研究（NCT02675231）。

综上，基于以上临床试验及理论基础，CDK4/6 抑制剂既可以增加内分泌治疗的疗效，也可以增强靶向 HER2 的抗肿瘤效应。复旦大学附属肿瘤医院开展了关于来曲唑、吡咯替尼联合 CDK4/6 抑制剂 SHR6390 治疗激素受体阳性、HER2 阳性晚期乳腺癌的 Ib 期临床研究。Ib 期研究的耐受性数据及初步的有效性数据如下：

1) 来曲唑 2.5 mg/d、吡咯替尼 400 mg/d 联合 SHR6390 125mg/d 剂量组：入组 5 例，2 例 DLT（III 级口腔黏膜炎），最佳疗效 ORR60%;
2) 来曲唑 2.5 mg/d、吡咯替尼 400 mg/d 联合 SHR6390 100mg/d 剂量组：入组 6 例，1 例 DLT（III 级口腔黏膜炎），最佳疗效 ORR 50%;

3) 来曲唑 2.5 mg/d、吡咯替尼 320 mg/d 联合 SHR6390 125mg/d 剂量组：入组 4 例，无 DLT；最佳疗效 3 例 PR，最佳疗效 ORR 75%。

根据 Ib 期研究的耐受性和安全性数据、并结合初步的有效性数据，推荐 II 期研究 联剂量为：来曲唑 2.5 mg/d、吡咯替尼 320 mg/d 联合 SHR6390 125mg/d。后续拟在此 基础上开展关于来曲唑、吡咯替尼联合 CDK4/6 抑制剂 SHR6390 治疗雌激素受体阳性、 HER2 阳性晚期乳腺癌的多中心 II 期临床研究。

1.1 药品名称

a)【通用名称】：来曲唑片

江苏恒瑞医药股份有限公司的来曲唑片为已上市药物，该药物的理化性质及生物效应信息详见药品说明书。

b)【通用名称】：吡咯替尼

【汉语拼音】Biluotini

【英文名称】Pyrotinib

【中文化学名称】[redacted]

【英文化学名称】[redacted]

c)【通用名称】：暂无

化合物代码: SHR6390

【汉语拼音】SHR6390 Pian

【英文名称】SHR6390 Tablets

【中文化学名称】

化学结构式: [redacted]

1.2 SHR6390 的药理类型和作用机制

SHR6390 为 CDK4/6 激酶抑制剂，是江苏恒瑞医药股份有限公司开发的[redacted]新药。临床前资料显示 SHR6390 选择性地抑制 CDK4/6 激酶活性，使其与 Cyclin D 组成的复合物不能磷酸化下游 Rb 蛋白，阻止细胞由 G1 期进入 S 期，从而发挥抑制细胞增殖和抗肿瘤的作用。

1.3 SHR6390 的药效学研究

[redacted]

1.4 SHR6390 的毒理学研究

[redacted]

1.5 SHR6390 的药代动力学研究

[redacted]

1.6 吡咯替尼联合 SHR6390 在 ER+、HER2+ 乳腺癌肿瘤模型体内抑瘤作用

[redacted]

2 临床研究

2.1 马来酸吡咯替尼临床研究

马来酸吡咯替尼片是由江苏恒瑞医药股份有限公司自主研制而成的原创药物，是一个不可逆性针对 EGFR、HER2 双靶点酪氨酸激酶抑制剂。其用于 HER2 表达阳性的晚期或转移性乳腺癌 II 期临床试验数据显示在针对治疗经蒽环类和紫杉类药物治疗失败，且复发/转移后化疗不超过 2 线的乳腺癌患者的 II 期临床试验中，马来酸吡咯替尼片联合卡培他滨片（简称“吡咯替尼组”）的客观缓解率为 78.5%，甲苯磺酸拉帕替尼片联合卡培他滨片（简称“拉帕替尼组”）的客观缓解率为 57.1%。吡咯替尼组的中位无进展生存期为 18.1 个月，拉帕替尼组的中位无进展生存期为 7.0 个月。吡咯替尼组的中位无进展生存期比拉帕替尼组显著延长，具有统计学显著意义（P<0.0001）。吡咯替尼组与拉帕替尼组相比，患者的疾病进展或死亡的风险下降了 63.7%（HR=0.363）。而且在患者疗效改善的同时，耐受性良好。基于目前 II 期临床试验获得的疗效和安全性数据，国家食品药品监督管理总局同意受理恒瑞医药递交的马来酸吡咯替尼片有条件上市的申请。随着 PHENIX 和 PHOEBE 两项大型 III 期研究的发布，2020 年吡咯替尼获得国家药监局的完全获批认定，获得完全批准的抗肿瘤创新药。

2.2 SHR6390 临床研究

SHR6390 目前已开展晚期实体瘤 I 期、晚期黑色素瘤 I 期晚期乳腺癌 Ib/II 及III期临床试验。

SHR6390-I-101 是一项在国内正在开展的，评价 SHR6390 对晚期实体瘤患者的耐受性及药代动力学 I 期临床研究，主要研究目的是观察 SHR6390 片单次及多次口服给药在晚期实体瘤患者中的剂量限制性毒性（DLT）及最大耐受剂量（MTD）；次要目的是观察 SHR6390 片在晚期实体瘤患者中的药代动力学特征、安全性、耐受性并评价

SHR6390 片治疗晚期实体瘤的初步有效性。该研究为单臂、开放、单次及多次给药、剂量递增、I 期临床试验。[redacted]

2.3 目前已上市 CDK4/6 抑制剂临床研究

Palbociclib 联合来曲唑一线治疗晚期 ER+/HER2 阴性乳腺癌的双盲、随机对照的 III 期 PALOMA-2 研究，共纳入 666 例患者，主要研究终点表明中位 PFS 可达 24.8 月，而对照组来曲唑为 14.5 月，实现了晚期乳腺癌一线治疗 PFS 超过 2 年的突破。最常见的 3 级或 4 级不良事件是中性粒细胞减少（Palbociclib-来曲唑组中发生率为 66.4％，安慰剂-来曲唑组中为 1.4％），白细胞减少（24.8％&0％），贫血（5.4 ％&1.8％）和疲劳（1.8％&0.5％）[9]。

Ribociclib 联合来曲唑对比安慰剂联合来曲唑用于一线治疗 HR 阳性、HER2 阴性复发或转移性的绝经后乳腺癌患者的疗效和安全性 III 期随机对照试验，共纳入 668 例患者，其中 Ribociclib 联合来曲唑 PFS 显着长于安慰剂组（HR，0.56; 95％CI，0.43-0.72）。中位随访时间为 15.3 个月。随访 18 个月后，Ribociclib 组的无进展生存率为 63.0％（95％CI，54.6-70.3），安慰剂组为 42.2％（95％CI，34.8-49.5）。总体反应率分别为 52.7％和 37.1％（P <0.001）。两组中超过 10％的患者报告的常见 3 级或 4 级不良事件为中性粒细胞减少症（Ribociclib 为 59.3％，安慰剂组为 0.9％）和白细胞减少（21.0％&0.6）；由于不良事件导致的停药率分别为 7.5％和 2.1％[10]。

Abemaciclib 或安慰剂联合非甾体芳香酶抑制剂用于一线治疗 HR 阳性、HER2 阴性绝经后晚期乳腺癌的双盲、随机 III 期的 MONARCH-3 研究中，共纳入 493 名晚期乳腺癌， Abemaciclib 组的中位 PFS 显着延长（HR=0.54; 95％CI，0.41-0.72; P = .000021; 中位数：Abemaciclib 组未达到，安慰剂组为 14.7 个月）。Abemaciclib 组的客观缓解率为 59％，安慰剂组为 44％（P = 0.004）。在 Abemaciclib 组，最常见的不良反应为腹泻（81.3％，其中 1 级为 44.6％）。Abemaciclib 对比安慰剂组，最常见的 3 级或 4 级不良事件是中性粒细胞减少（21.1％vs 1.2％），腹泻（9.5％vs 1.2％）和白细胞减少（7.6％ vs 0.6％）[11]。

3 研究目的与研究终点

3.1 主要研究目的

第一阶段（Ib期）：

确定非甾体类芳香化酶抑制剂（来曲唑）、马来酸吡咯替尼片联合CDK4/6抑制剂SHR6390治疗激素受体阳性、HER2阳性晚期乳腺癌的安全性和耐受性，结合初步的有效性数据，明确此联合方案的II期推荐给药剂量。

第二阶段（II期）：

评估来曲唑、吡咯替尼联合CDK4/6抑制剂SHR6390治疗雌激素受体阳性、HER2阳性晚期乳腺癌的有效性和安全性，进一步探索ctDNA作为生物标志物的价值，以及FDG-PET、HER2-PET评估抗肿瘤疗效的临床应用价值，[redacted]

3.2 主要研究终点

本试验的主要研究终点包括:

第一阶段（I期）

联合方案中SHR6390的剂量限制性毒性（DLT）和最大耐受剂量（MTD），确定II期临床研究推荐给药方案；

各剂量组的不良事件（AE）及严重不良事件（SAE）的发生率及严重程度。

第二阶段（II期）

II期临床研究推荐给药方案的客观有效率（ORR），基于RECIST1.1评价标准。

3.3 次要研究终点

第一阶段（I期）

ORR，基于RECIST1.1。

第二阶段（II期）

AE和SAE的发生率及严重程度。

I/II期试验的共同次要研究终点包括：

- 无进展生存期（PFS）；
- 疾病控制率（DCR）：疗效评价为CR/PR/SD的受试者比例；
- 临床获益率（CBR）：研究过程中出现CR、PR及SD≥24周的受试者比例；
- 缓解持续时间（DoR）；
- 探索性分析分子标志物疗效之间的关系。

4 试验药品

4.1 名称和来源

本项目研究药物来曲唑、吡咯替尼及SHR6390片：均由江苏恒瑞医药股份有限公司生产并提供。

4.2 药品剂型和规格

来曲唑片 规格：2.5mg；吡咯替尼片 规格：160mg、80mg；SHR6390片 规格：25mg、125mg。

4.3 保存条件

保存条件：25°C以下密封保存。 有效期：暂定 24 个月。

4.4 使用方法

- 非甾体类芳香化酶抑制剂：来曲唑，口服，2.5 mg，每天1次，空腹给药，连续给药28天为1个周期。
- 吡咯替尼：口服，320mg，每天1次，早餐后30分钟内口服给药，连续给药28天为1个周期。
- SHR6390：口服，125mg，每日1次，空腹给药（给药期间应至少保证服药前1小时和服药后1小时禁食）。服药以28天为一个给药周期，每周期前3周（D1~21）连续服药，第4周（D22~28）停药。

4.5 试验药物的管理、发放与回收

本试验临床用药的管理、发放和回收由专人负责，研究者必须保证所有试验用药物仅用于参加该临床试验的受试者，其剂量与用法应遵照试验方案，剩余的药品退回，不得把临床用药转交任何非临床试验参加者。

药物分发时须签署药物接收单，双人签字，一式两份。研究结束收回剩余药品及空盒，签署药品回收单。每一份药物的发放及回收均应在专门记录单上及时记录。

5 研究总体设计

本研究为一项单臂、开放、剂量爬坡的Ib期临床研究和单臂、开放、多中心II期临床试验。依据《药品注册管理办法》、《药品临床试验质量管理规范》、《新药(化学药品)临床药代动力学试验指导原则》，本试验拟在经常规标准治疗无效的或缺乏标准

治疗的雌激素受体阳性、HER2阳性晚期乳腺癌患者中开展来曲唑、吡咯替尼联合CDK4/6抑制剂SHR6390治疗的有效性和安全性。

6 实验设计

6.1 药品的剂量和给药方案

在经常规标准治疗无效的或缺乏标准治疗的激素受体阳性、HER2阳性晚期乳腺癌患者进行联合给药的爬坡试验，开展耐受性观察，并初步观察疗效。每个剂量组最后一名受试者完成连续给药28天且该剂量组DLT观察结果满足剂量向上递增原则时，即可开始高一档剂量组爬坡试验。

第一阶段（Ib期）：

本研究来曲唑为 2.5 mg/d，吡咯替尼给药剂量为 400 mg/d 或 320 mg，SHR6390 设计了 3 个剂量组：

剂量组	SHR6390	吡咯替尼片	病例数
A	125 mg/d	400 mg/d	3~6
B	150 mg/d	400 mg/d	3~6
C（备用组）	100 mg/d	400 mg/d	3~6
D（备用组）	125 mg/d	320 mg/d	3~6
E（备用组）	150 mg/d	320 mg/d	3~6
F（备用组）	100 mg/d	320 mg/d	3~6

试验从 A 组开始，采用 3+3 剂量递增的原则设计方案，递增顺序为 A 组→B 组。如发现 A 组出现≥2 例 DLT 时，则启用 C 组及 D 组，如发现 C 组仍不能耐受，则启动备用 F 组；若 D 组能够耐受，则递增至 E 组，若 D 组不能耐受，则启动 F 组。若 F 组仍不能耐受则由研究 团队将共同分析试验数据判断是否终止试验。每个剂量组受试者给药 1 周期（28 天）结 束后对 DLT 进行评估。三药的具体服用方式见 4.4 使用方法。前 2 周期为核心试验阶段， 第 2 周期末疗效评估为 CR/PR/SD 的受试者，可接受该剂量组联合方案持续给药至受试者 出现疾病进展、不能耐受毒性或患者主动要求退出等情况为止。

第二阶段（II期）：

根据第一阶段确定的联合给药II期推荐剂量：吡咯替尼320 mg/d联合SHR6390 125mg/d联合来曲唑2.5 mg/d，进行第二阶段扩组研究，连续给药至受试者出现疾病进 展、不能耐受毒性或患者主动要求退出等情况为止。

6.2 剂量限制性毒性（DLT）

本试验中 DLT 定义为在第 1 周期内出现的与药物相关或可能相关的以下不良事件（CTC-AE v4.0.3 标准）：

1. 血液学毒性：

- 4 级中性粒细胞减少持续≥5 天；
- 4 级血小板减少，或 3 级血小板减少伴临床显著出血；
- ≥3 级中性粒细胞减少伴发热（≥38.0 摄氏度持续 1 小时或者>38.3 摄氏度）；
- ≥4 级贫血。

2. 非血液学毒性：

任何≥3 级非血液学毒性，以下情况除外：

- 3-4 级恶心/呕吐和/或腹泻和/或电解质紊乱，经最佳支持治疗后 72 小时内恢复至≤2 级者；
- 明确与肿瘤相关、与药物无关的 3-4 级的碱性磷酸酶、谷氨酰转肽酶升高。

6.3 最大耐受剂量(MTD)

在第1周期（28天）的给药观察期内，若某一剂量组最终有≥1/3的受试者出现DLT，则前一剂量组为MTD。如爬坡至I期临床试验B剂量组仍未能有1/3受试者出现DLT，则由研究者讨团队论确定是否启用备用的C剂量组，并确定II期试验的推荐剂量。

6.4 受试者替换

对于在DLT观察期内发生非DLT而导致需要退出研究的情况，则增加 1 例受试者作为替补。

DLT观察期内因非计划暂停用药时间超过 4 天，则认为受试者接受药物治疗强度不足，如未观察到DLT，亦需增加1例受试者作为替换。

6.5 样本量计算

根据Ib期研究及II期研究首例受试者的数据分析，预计II期研究抗HER2一线治疗受试者ORR为50%，二线治疗受试者ORR为43%。

以既往研究 ORR 28% 作历史对照，在α为0.05、把握度80%的情况下，计算一线受试者样本量为35例，考虑10%失访率后样本量为39例。历史对照依据：EGF30008研究中，HER2+HR+MBC受试者1线接受拉帕替尼联合来曲唑治疗的ORR为28%。

以既往研究 ORR 19% 作历史对照，在α为0.05、把握度80%的情况下，计算二线受试者样本量为25例，考虑10%失访率后样本量为28例。历史对照依据：ALTERNATIVE研究中，HER2+HR+MBC受试者1-2线接受拉帕替尼联合来曲唑治疗的ORR为18.6%。

7 标本收集

Ib期收集第1周期第21天给药后1小时、3小时及24小时的血浆。II期收集8-10人第1周期第21天吡咯替尼给药前0.5h内、给药后2h±5min、4h±10min、6h±10min、12h±10min、24h±0.5h血浆；第3、第5、第8、第12周期第22天吡咯替尼给药前0.5h内，PK采血前3天服药时间相对固定。采集血液分别用2管肝素锂抗凝采血管各抽取静脉血 3 mL，轻柔颠倒3-5次使血液与抗凝剂充分混合，室温放置。准确记录实际采血时间，详细记录在采血记录表中，采血管在采集后的1小时内转移至离心机中，室温(15 °C -32 °C下以2000g 离心 10 min，分离血浆， 分装到 2 个冻存管中，一个检测管，一个备份管，检测管血浆量不少于 0.5ml，其余血浆均转移至备份管中，注意不要将下层血细胞吸出，并贴好对应标签，置-80±10° C 低温冰箱保存待测。冻存管在寄送之前需始终保持在低温冰箱中，并每天记录冰箱温度，寄送样品时，需将检测管与备份管分开寄送：先将检测管寄出，备份管仍保存于-80±10℃冰箱内，待试验结束时统一将备份管寄出。

基线期、2周期末、疾病进展/开始新的抗肿瘤治疗前，采集受试者血样（EDTA抗凝管2管和单核细胞分离管1管），送医院组织库(组长单位及有条件的参与单位)及ctDNA血样；收集已有的石蜡包埋肿瘤组织样本或白片≥10张，尽量获取转移灶穿刺标本。

8 受试者的选择

8.1 受试者及研究例数

从伦理学和科学性方面考虑，受试者需既往至多接受过1个针对复发转移性乳腺癌的含曲妥珠单抗方案的系统性治疗；既往未接受过抗HER2 TKI治疗或接受但未证明治疗失败；既往内分泌治疗未证明芳香化酶抑制剂耐药；临床试验前必须签署知情同意书。

第一阶段入组15例；根据第一阶段研究结果，预计第二阶段入组抗HER2一线治疗39例受试者，入组抗HER2二线治疗28例受试者。

8.2 入选标准

患者必须满足以下所有入选标准才可入组本试验:

1. 受试者自愿加入本研究，签署知情同意书，有良好的依从性；
2. 年龄 18-75 岁（含界值）的女性患者；
3. 具有经组织病理确认的复发/转移性乳腺癌患者，且 ER 表达阳性，HER2 表达阳性；

- HER2 表达阳性指标准免疫组化染色（IHC）检测显示 HER2 为 3+和/或原位杂交技术（ISH）阳性；
- ER 阳性定义为 ER 表达阳性的细胞百分比≥1%；
- 局部复发者需经研究者确认无法进行根治性手术切除；

4. 至少具有 1 个符合 RECIST 1.1 标准的颅外可测量病灶；
5. 自然绝经后妇女，或已接受双侧卵巢切除术或接受卵巢功能抑制的女性；
6. 既往抗肿瘤治疗的规定：
   - 既往至多接受过 1 个针对复发转移性乳腺癌的含曲妥珠单抗方案的系统性治疗【包括抗 HER2 ADC，后续含义相同】
     i. 晚期阶段未经含曲妥珠单抗方案治疗，或含曲妥珠单抗方案辅助治疗结束后 1 年以上复发的，后续治疗作为抗 HER2 一线治疗入组；
     ii. 一线经过含曲妥珠单抗方案治疗失败，或含曲妥珠单抗方案辅助治疗期间复发或辅助治疗结束后 1 年以内复发的，后续治疗作为抗 HER2 二线治疗入组；
   - 既往未接受过抗 HER2 TKI 治疗或接受但未证明治疗失败；
   - 既往内分泌治疗未证明芳香化酶抑制剂耐药（耐药定义：辅 助芳香化酶抑制剂治疗过程中或结束后 1 年以内复发，复发转移阶段接受过芳香化酶抑制剂并疾病进展）。
7. ECOG 评分：0～1；
8. 预期生存期≥12 周；
9. 重要器官的功能符合下列要求（在入组前 2 周内未使用过任何血液成分及细胞生长因子）：

- 中性粒细胞绝对计数≥1.5×10^9/L；
- 血小板≥90×10^9/L；
- 血红蛋白≥90g/L；
- 总胆红素 TBIL≤1.5 倍 ULN；
- ALT 和 AST ≤2.5 倍 ULN；
- 尿素/尿素氮（BUN）和肌酐（Cr）≤1.5×ULN；
- 左室射血分数（LVEF）≥50%；
- Fridericia 法校正的 QT 间期（QTcF）< 470 毫秒。
- INR≤1.5×ULN，APTT≤1.5×ULN。

8.3 排除标准

具有以下任何一项的患者不能入组本研究：

1. 受试者有未经治疗的中枢神经系统转移；
2. 既往接受过系统性、根治性脑或脑膜转移治疗（放疗或手术），但影像学证实稳定已维持至少 4 周，且已停止全身性激素治疗大于 4 周、无临床症状的患者可以纳入；
3. 既往接受过任何 CDK4/6 抑制剂治疗；
4. 基线期存在伴有临床症状的腹水、胸腔积液、心包积液，需要引流者，或首次用药前 4 周内进行过浆膜腔积液引流者；
5. 无法吞咽、肠梗阻或存在影响药物服用和吸收的其他因素；
6. 入组前 4 周内接受过化疗、分子靶向治疗或其他临床试验药物等系统治疗；入组前 2 周内接受过内分泌治疗；
7. 受试者既往 5 年内或同时患有其它恶性肿瘤（已治愈的皮肤基底细胞癌和宫颈原位癌除外）；
8. 在首次用药前 4 周内接受过重大手术操作或明显的创伤，或预计患者将要接受重大手术治疗；
9. 妊娠期、哺乳期女性受试者，或有生育能力女性的基线妊娠试验检测阳性，或不愿意采取有效避孕措施的育龄期受试者；
10. 已知对本方案药物组分有过敏史者；
11. 有活动性 HBV、HCV 感染者；经药物治疗后稳定的乙肝（HBV 病毒拷贝数不高于参考值上限）及已治愈的丙肝患者（HCV 病毒拷贝数低于测定法的检测下限）除外；
12. 有免疫缺陷病史，包括 HIV 检测阳性，或患有其他获得性、先天性免疫缺陷疾病，或有器官移植史；
13. 曾患有任何心脏疾病，包括：（1）心绞痛；（2）需药物治疗的或有临床意义的心率失常；（3）心肌梗死；（4）心力衰竭；（5）任何被研究者判断为不适于参加本试验的其他心脏疾病等；筛选期检查发现心脏功能或肾功能异常严重程度≥II 度；
14. 根据研究者的判断，有严重的危害患者安全、或影响患者完成研究的伴随疾病（如：严重的高血压、糖尿病、甲状腺疾病等）；
15. 既往有明确的神经或精神障碍史，包括癫痫或痴呆；

16. 首次用药前 4 周内并发重度感染（如：根据临床诊疗规范需要静脉滴注抗生素、抗真菌或抗病毒药物），或在筛选期间/首次给药前出现不明原因的发热>38.3°C。

8.4 受试者识别

本试验中所有签署了知情同意书的受试者将获得一个受试者代码，该代码为唯一的编号，如 II-01-01；编号规则为：前 2 位数为试验阶段，如 II；中间2位数为中心编号，依次为01、02、03类推；后 2 位按该中心筛选顺序为 01、02、03 类推。

8.5 脱落标准

所有签署了书面知情同意书并筛选合格进入试验的患者，均有权随时退出临床试验。无论何时何因退出，只要没有完成多次给药第 1 个周期且无法进行安全性评价的受试者，视为脱落病例。

8.6 受试者治疗终止标准

如出现以下一种及一种以上情况，该受试者须退出试验：

1. 受试者撤回知情同意，要求退出；
2. 经影像学检查显示病情进展；
3. 无法耐受毒性者；
4. 严重违背试验方案，研究者评估认为应该终止治疗者；
5. 受试者失访或发生妊娠事件；
6. 研究者认为其他有必要退出研究的情况。

8.7 研究终止标准

本研究终止标准，包括但不限于：

1. 发现对受试者有非预期的、意义重大的或不可接受的风险；
2. 研究药物/试验治疗无效，或继续试验是无意义的；
3. 由于诸如受试者入选严重滞后或重大违背方案等原因，研究者决定终止研究。

9 剂量调整与伴随用药

9.1 DLT 观察期

Ib阶段DLT观察期内，出现非DLT的不良事件时，原则上不予医学处理，以便观察试验药物可能的不良反应及其程度和可逆性。但一旦出现方案中规定的DLT毒性时，须立即停止使用研究药物，并积极处理，在 CRF 上记录使用的药物。

9.2 连续给药第 2 周期及后续周期

药物调整标准（II 期研究全部用药周期调整用药参照此标准）

发生毒性反应后，可由医生根据情况判断，给予相应的处理，具体处理原则如下:

表6

不良事件	严重程度			
	I 级	II 级	III 级	IV 级
**血液学毒性**	维持原剂量	维持原剂量	暂停用药，对症处理，恢复至 I 级及以下，根据研究者判断，本周期及后续周期可维持原剂量或下调一个剂量	暂停用药,对症处理，恢复至 I 级及以下,根据研究者判断,本周期及后续周期下调一个剂量
**非血液学毒性**	维持原剂量	维持原剂量或暂停用药，对症处理，恢复至 I 级及以下,根据研究者判断,本周期及后续周期可维持原剂量或下调一个剂量	暂停用药，对症处理，恢复至 I 级及以下，根据研究者判断，本周期及后续周期下调一个剂量	永久终止用药,退出研究
**发热性中性粒细胞减少**	-	-	暂停用药，对症处理，恢复至 I 级及以下，根据研究者判断，本周期及后续周期下调一个剂量	永久终止用药,退出研究

出现明确与研究药物相关的毒性时，由研究者根据临床表现具体处理。待恢复至≤I 级（或研究者判断≤II 级的不良事件对受试者可耐受且无明显的安全风险）后按再次

给药。若相同不良事件再次复发，根据研究者判断进行暂停与剂量调整，或要求受试者退出研究；研究者将以最大程度保护受试者安全性。

如因暂停用药后毒性在 2 周内仍无法恢复，原则上受试者应退出研究。暂停给药时间需计入给药周期。

9.3 研究期间不可使用的药物

治疗期间应停用其他抗肿瘤药物和肿瘤治疗相关的辅助性药物，包括抗肿瘤中药、抗肿瘤内分泌治疗药物、免疫治疗或其他抗肿瘤治疗药物等。

9.4 研究期间可酌情使用的药物

受试者如果出现不良反应，应进行密切观察，在必要时予以积极对症治疗，并在CRF表上记录和说明所使用的治疗药物。以上治疗所使用的药物应记录使用时间，使用药物名称，用法与用量。

10 研究步骤

开始研究前，患者必须阅读并且签署目前伦理委员会(EC)批准的知情同意书。所有研究步骤均需在研究计划表所指明的时间窗内进行。

10.1 筛选期

以下筛选步骤在开始研究药物治疗前的 28 天内完成:

- 签署知情同意书
- 采集病史和人口统计学资料，包括患者身份证、通讯地址、联系电话；详细的询问肿瘤病史/其他疾病史：病理结果、ER/PR/HER2检测报告；肿瘤手术、化疗、放疗史以及其他疾病治疗史；除乳腺癌以外肿瘤病史
- 乙肝、丙肝及HIV检查：乙肝五项检查，若检查结果异常，应进行病毒复制（HBV DNA）检测；丙肝病毒抗体（抗HCV）、HIV抗体检查
- 影像学检查：筛选期影像学检查包括胸部、腹部及颅脑的增强CT或MRI，以及研究者怀疑存在病灶的其他部位（如颈部或盆腔）CT/MRI。肿瘤基线评估可放宽至首次给药前4周内，在签署知情同意前获得CT/MRI扫描结果只要符合要求可以用于筛选期肿瘤评估；临床怀疑有骨转移时需进行骨扫描检查（FDG-PET、HER2-PET检查在组长单位及有条件的参与单位进行）
- [redacted]

- 生物标记物收集：[redacted]及ctDNA血样；收集已有的石蜡包埋肿瘤组织样本或白片≥10张，尽量获取转移灶穿刺标本
- 超声心动图：随访LVEF值变化，如出现LVEF下降至＜50%且较基线下降≥10%时，或出现胸痛、心悸等症状时，可增加计划外检查
- 评估当前使用的伴随用药和治疗
- 从签署知情起收集不良事件

以下筛选步骤在开始研究药物治疗前的 7 天内完成:

- 生命体征检查：体温、呼吸、脉搏、血压，血压测量时，测量前 30 分钟内禁止吸烟和饮咖啡，至少安静休息 10 分钟，测量时取坐位，肘部置于与心脏同一水平，每次血压测量均取同侧测量
- 体格检查：主要身体系统的检查（头面部、皮肤系统、淋巴结、眼部、耳鼻喉部、口腔、呼吸系统、心血管系统、腹部、生殖泌尿系统、肌肉骨骼、神经系统和精神状态）
- ECOG评分
- 血常规：血红蛋白、红细胞、白细胞、中性粒细胞计数、淋巴细胞计数和血小板计数
- 尿常规：尿蛋白、尿糖、尿潜血（尿红细胞、白细胞）。如果半定量方法显示蛋白2+，则进行24小时尿蛋白定量检查
- 大便常规：包括大便潜血
- 血生化：总胆红素、结合胆红素、ALT、AST、AKP、γ-GT、LDH、总蛋白、白蛋白、尿素/尿素氮、肌酐、尿酸、空腹血糖、甘油三酯、胆固醇、钾、钠、氯、钙、磷、镁；必要时增加心肌酶谱检查
- 妊娠检查：育龄期女性首次用药前1周内进行血清妊娠检测
- 12导联心电图：若发现心电图异常有临床意义，研究者认为必要时可再次确认

10.2 不良事件的收集/试验期

以下事项应根据试验流程表所列的时间完成（注意：第 1 周期起时间窗为±3 天）。

- ECOG评分、生命体征、体格检查、12 导联心电图：第1周期第15、28天，第2周期第28天（C1D28/C2D1 均可，后同），之后每个周期第 28 天进行；
- 血常规、血生化：第1周期第15、28天，第2周期第28天（C1D28/C2D1 均可，后同），之后每个周期第 28 天进行（受试者如为外地患者，第 2 周期起可在当

地医院每周增加一次血常规检查并将结果告知研究者，由研究者判断是否需要返回研究中心进行进一步检查）；

- 尿常规、大便常规：每3周期一次，第1周期以筛选期检查替代，第2周期第1天（C1D28/C2D1 均可，后同），之后每个周期第 28 天进行；
- 超声心动图：每3周期一次，第1周期以筛选期检查替代，第4周期第1天（C3D28/C4D1 均可，后同），之后每3个周期第 28 天进行；治疗结束或受试者退出时（若之前 4 周内未做）；
- 生物标记物收集：基线、2周期末、疾病进展/开始新的抗肿瘤治疗前，采集[redacted]及ctDNA血样。
- 影像学检查：肿瘤影像学检查允许的时间窗为±7天，治疗期影像学检查应在与基线检查相同的条件（扫描的层厚、造影剂的使用等）下，具体评估时间点如下：
  - 用药前 6 周期，第 2 周期末进行首次评估，此后每 2 个周期评估 1 次，6 周期之后每 3 周期进行一次，直至受试者疾病进展、不可耐受的毒性或开始新的肿瘤治疗；
  - 首次评估为 CR、PR 者建议在 4~6 周后进行确认，确认后的肿瘤评估不能改变之前固定的两个周期检查时间点；
  - 当怀疑疾病进展（如症状恶化）可进行计划外的影像学检查；
  - 除了由于影像学证实的疾病进展外，因其它原因结束试验治疗的受试者，若试验结束前 4 周内未进行影像学评价，须在治疗结束时进行影像学评价，同时，在结束试验后继续按照方案规定的随访频率随访肿瘤疗效，直至有记录证实疾病进展或开始新的肿瘤治疗。
- [redacted]
- 不良事件：从签署知情同意书起至开始用药前只记录严重不良事件，直至末次用药后 28 天，并且随访直至不良事件消失、缓解至基线水平或≤1 级、稳定。
- 记录不良反应：自研究治疗第1天开始，直至最后1次治疗后至少28天，或直至所有严重的或药物相关毒性安全恢复至≤ NCI-CTC AE 4.0.3 标准I 度。此外，观察用药期间各种临床表现并予以记录；
- 记录伴随用药或治疗：自研究治疗前28天开始，直至最后一次治疗后的28天。

注：研究者可根据实际情况，增加受试者访视频率（如每周一次）与检查内容，最大程度保证受试者安全性最大化。

10.3 研究治疗结束/退出研究

研究结束：末例受试者入组后24个月或研究者认为需提前结束试验时。

10.4 治疗结束后随访

- 疾病进展时间：除了由于影像学证实的疾病进展外，因其它原因结束试验治疗的受试者，若试验结束前4周内未进行影像学，须在治疗结束时进行影像学评价，同时，在结束试验后继续按照方案规定的随访频率随访肿瘤疗效，直至有记录证实疾病进展或开始新的肿瘤治疗。
- 生存随访：试验治疗终止后，每3个月可通过临床随访或者电话随访收集生存状态和后续抗肿瘤治疗情况，直至死亡。

10.4.1 不良事件严重程度判断标准

参照 NCI-CTC AE 4.0.3 版关于药物不良反应的分级标准。如果出现表中未列出的不良反应可参照下列标准：

- I 级：轻度，无临床症状或有轻微临床症状；仅有临床或实验室检查异常；不需治疗。
- II 级：中度，需要微量的、局部的或非侵害性的治疗；与年龄相符的使用工具的日常生活活动(Activities of Daily Living，ADL)受限，使用工具的日常生活指做饭、购物、打电话、数钱等。
- III 级：病情重或有医学上严重的症状但是暂时不会危及生命；导致住院或住院时间延长；导致残疾；日常生活自理(Self care ADL)受限。日常生活自理指：洗澡、穿衣、脱衣、吃饭、去卫生间、吃药等，非卧床不起。
- IV 级：危及生命，需要紧急治疗。
- V 级：因不良事件致死。

11 安全性评价

11.1 不良事件(AE)

11.1.1 不良事件的定义

不良事件是指临床试验受试者从签署知情同意书后出现的不良医学事件。本试验从受试者接受研究药物治疗开始，直至最后一次使用研究药物后 28 天发生的任何不良医疗事件，无论与试验药物是否有因果关系，均判定为不良事件。

研究人员应详细记录受试者所发生的任何不良事件，包括：不良事件及所有相关症状的描述、发生时间、严重程度、持续时间、采取措施及最终结果和转归。

关注的不良事件

发生毒性反应后，原则上不予以停药或下调剂量，可由医生根据情况判断，给予相应的处理，具体处理原则如下：

- 血液学支持：出现血液学毒性时，研究者可根据临床表现等进行对症处理。血液学毒性达到≥III 度时，需暂停用药，待恢复至≤I 度后按原剂量水平给药。若出现 III 度或 IV 度贫血，可不暂停剂量，根据研究者的判断可进行输血治疗。暂停给药不影响肿瘤评价的时间点。若暂停用药时间超过 14 天，患者需退出研究。
- 腹泻：根据试验方案中“给药暂停和剂量调整”的相关规定，先给予对症治疗，密切随访或观察（≤ 14 天）。临床建议在腹泻当天开始给予口服蒙脱石散（3 g/袋，3 次/日）或洛哌丁胺。对于仍不能缓解的 III 级腹泻则予药物暂停；待不良事件恢复至 I 级以内后再恢复原剂量给药或降低一个剂量给药。
- 肝功能异常：由研究者根据受试者及不良事件情况给予对症治疗或观察（≤14 天），治疗或观察后仍存在，密切随访，由研究者判断，必要时可予剂量调整和/或增加血生化的检查频率。对于有肝转移的受试者，入组时如 ALT/AST 超出 1.5×UNL，需对肝功能进行密切监控，综合考虑决定该受试者是否适合入组本研究。
- 其他风险：由于研究药物是研究性的，单独用药或与其他药物联合用药时，可能会存在未知的其他风险。所有药物都有过敏反应的潜在风险，如果不及时治疗可能会危及生命。出现了任何一种下述严重过敏反应症状：活动后呼吸困难或面部、口唇、牙龈、舌头或颈部肿胀，应立即获得医疗帮助并联系研究医生。其他过敏反应可能包括皮疹、荨麻疹或水疱。患者尽快报告发生的所有症状和副作用非常重要，不论患者是否认为这些是由研究药物引起的。

本实验导致的不适可能不限于上述事件，但我们会密切随访、积极处理，最大程度保证患者的安全及利益，以上治疗所使用的药物要记录在 CRF 表上。

11.1.2 不良事件与试验药物关系的分类

不良事件包括所有非预期的临床表现，只要这些事件发生于签署知情同意书后，不管是否与试验药物有关系，甚至不管是否应用药物，均应按不良事件报告，所有不良事

件必须以临床报告的形式呈报。凡受试病例治疗期间患者主诉的任何不适反应或客观实验室检查指标有异常改变，应如实记录，同时注明不良事件表现严重程度、持续时间、处理措施及转归等，临床医师尚应综合判定不良事件与试验药物关系，不良事件与试验药物关系按5级判定：即肯定有关、可能有关、可能无关、肯定无关、无法评定。前两者计为不良反应，统计不良反应的发生率。

- 肯定有关：反应出现符合用药后合理的时间顺序，反应符合所疑药物已知的反应类型；停药后改善，重复给药再出现该反应。
- 可能有关：反应出现符合用药后合理的时间顺序，反应符合所疑药物已知的反应类型；病人的临床状态或其它治疗方式也可能产生该反应。
- 可能无关：反应出现不太符合用药后合理的时间顺序，反应不太符合所疑药物已知的反应类型；病人的临床状态或其它治疗方式有可能产生该反应。
- 肯定无关：反应出现不符合用药后合理的时间顺序，反应有符合非试验药物已知的反应类型；病人的临床状态或其它治疗方式可能产生该反应，疾病状态改善或停止其它治疗方式反应消除，重复使用其它治疗方法反应出现。
- 无法评定：反应出现与用药后的时间无明确关系，与该药品已知的反应类型相似，同时使用的其它药物也可能引起相同的反应。

11.2 严重不良事件（SAE）

11.2.1 严重不良事件的定义

严重不良事件是指临床试验过程中发生需要住院治疗或延长住院时间、伤残、影响工作能力、危及生命或死亡、导致先天畸形等医学事件。包括以下非预期医学事件：

- 导致死亡的事件；
- 危及生命的事件（定义为受试者在事件发生时有死亡危险）；
- 需要住院治疗或延长住院时间的事件；
- 可导致永久性或严重残疾/功能不全的事件；
- 先天异常或出生缺陷；
- 其他重要医学事件。

11.2.2 妊娠

临床试验期间女性受试者怀孕，则受试者出组，若男性受试者的伴侣怀孕，则受试者继续临床试验，并按照严重不良事件报告。

11.2.3 疾病进展

疾病进展（包括进展的症状和体征）不应作为严重不良事件报告，但如果在试验或安全报告期限内因疾病进展死亡应报告为严重不良事件。因疾病进展的症状和体征而住院不应作为严重不良事件报告。在试验或安全报告期限内，如果癌症的最终结果为死亡，那么导致死亡的事件必须作为严重不良事件报告。

11.2.4 进行其他抗肿瘤治疗

不良事件的记录从签署知情同意书开始至最后一次使用研究药物后 28 天。若 28 天后受试者开始进行其他抗肿瘤治疗，对于非死亡的不良事件，不需要再进行不良事件的记录跟踪。若死亡发生在研究治疗结束后的严重不良事件报告期限内，无论患者是否接受其他治疗，均必须报告。

11.2.5 住院治疗

临床研究中导致住院治疗或住院时间延长的不良事件应视为严重不良事件。任何初次被医疗机构收住院（即使短于 24 小时）的情况均符合此标准。

住院不包括以下情况：

- 康复机构
- 疗养院
- 常规急诊室收治
- 当日手术（如门诊/当日/非卧床的手术）与不良事件恶化无关的住院治疗或住院时间延长本身不是严重不良事件。例如：因原有疾病入院，并没有新的不良事件的发生，也没有原有疾病的加重（如：为了检查试验前至今持续存在的实验室检查异常）；
- 管理原因的住院（如：每年例行的体检）；
- 临床试验期间试验方案规定的住院（如：按试验方案的要求进行操作）；
- 与不良事件恶化无关的择期住院（如：择期整容手术）；
- 已预定的治疗或外科手术应在整个试验方案和/或受试者个人的基线资料中予以记录；
- 仅因为血液品使用而入院。

诊断性或治疗性的侵入性（如手术）、非侵入性操作不应作为不良事件报告。但导致此项操作的疾病状况符合不良事件的定义时，应予以报告，如不良事件报告期间发病

的急性阑尾炎应报告为不良事件，而因此进行的阑尾切除术应记录为该不良事件的治疗方法。

12 严重不良事件的报告制度

严重不良事件的报告应自受试者签署知情同意书始，直至最后一次使用研究药物后的 28 个公历日（含 28 天）。试验期间，若发生严重不良事件，无论是首次报告还是随访报告，研究者都必须立即填写《新药临床研究严重不良事件（SAE）报告表》，签名及注明日期，在研究者获知 SAE 的 24 小时内立即通知申办者，并立即停止该受试者试验，采取保护受试者的相应措施。研究者应对严重不良事件追踪至解决。

严重不良事件应详细记录症状、严重程度、发生时间、处理时间、采取措施、随访时间和方式以及转归情况。如果研究者认为某严重不良事件与试验药物无关，而与研究条件（例如终止原治疗，或试验过程中的合并症）潜在相关，则这种关系应在病历报告表的严重不良事件页的叙述部分详细说明。

13 SAE 的报告程序

临床研究过程中及停药后28天内发生的任何严重不良事件，必须立即书面报告申办者。获得主要疗效指标结果后，在观察次要疗效指标结果的试验期间，发生的SAE应立即上报申办者。同时，研究者必须填写严重不良事件报告表（SAE），对严重不良事件的发生时间、严重程度、与试验药的关系及采取的措施等进行详细说明，并在报告上签名。

14 疗效评价

疗效评价采用RECIST 1.1标准，包括：①评价每一例受试者的疗效，包括完全缓解（CR），部分缓解（PR），病情稳定（SD）以及疾病进展（PD）。② 记录无进展生存期（PFS），指患者开始研究治疗之日至任何有记录的肿瘤进展或任何原因的死亡之间的时间，以先出现者为准。首次被评价为CR、PR的受试者需在4-6周后进行确认。对一般状况的好转或恶化，用治疗前后ECOG评分的变化来表示。

15 研究结束

末例受试者入组后 24 个月或研究者认为需提前结束试验时。

16 安全控制措施

① 特殊处理

- 停止给药。
- 监测生命体征：心电图、血压、呼吸、体温。
- 洗胃：1%～2%氯化钠溶液或 1:5000 高锰酸钾溶液。
- 导泻：硫酸钠 15～30 g，加水 200 mL 给药。
- 灌肠：1%微温肥皂水（约 5000 mL）高位连续清洗。

② 支持疗法

- 镇静，给氧。
- 建立静脉输液通道，开放呼吸通道，必要时予气管插管、心外按摩以及呼吸机辅助呼吸支持。
- 充分补液，维持循环血量：静脉注射生理盐水或葡萄糖氯化钠注射液，根据病情补充胶体，保证循环渗透压。
- 强心，升压，维持血压稳定，保证重要脏器的血供：可首先予肾上腺皮质激素 20～60 mg 加 5%葡萄糖注射液 50～250 mL 静脉滴注，血压稳定后可给予多巴胺泵入维持。
- 利尿，根据尿量给予利尿药如速尿等，适量给予碳酸氢钠，碱化尿液。
- 维持水电解质、酸碱平衡。
- 抗心律失常。
- 对症治疗，维持氮平衡。

③ 抗过敏

- 扑尔敏 10 mg 给药或异丙嗪 25～50 mg，平卧、吸氧、保证呼吸道通畅。
- 0.1%肾上腺素 0.1～0.2 mL 加 5%葡萄糖静脉滴注。
- 应用糖皮质激素如地塞米松。

④ 预防性应用胃酸抑制剂

⑤ 纠正呼吸循环衰竭

给氧或人工呼吸，用可拉明 0.375 g，洛贝林 3～6 mg 交替注射，每 15～30 分钟 1 次，必要时静注 1～2 次，以及强心甙药物洋地黄制剂等。

17 推荐Ⅱ期临床试验给药方案

根据连续给药研究的耐受性、初步疗效以及药物暴露的结果，综合分析并判断最佳的给药方案为吡咯替尼为320 mg/d、 SHR6390 125mg/d联合内分泌治疗，进入Ⅱ期临床研究。

18 伦理规范及知情同意

18.1 伦理规范

国内相应法规本临床试验必须遵循赫尔辛基宣言（1996 年版）、CFDA 颁布的《药品临床试验管理规范》（GCP）以及相关的法规。

18.2 知情同意

受试者在接受本试验治疗前必须对参加本试验知情同意，以保障受试者的合法权益。研究者向受试者或其指定代表人完整、全面地介绍本研究的目的、药物的作用、可能出现的毒副反应和可能的风险，应让受试者知道他们的权利，所要承担的风险和受益。谈话是十分重要的知情同意过程。如受试者和其合法代表无识字能力，知情同意过程应有见证人参加，由受试者或其合法代表口头同意后，在知情同意书上签名，见证人的签名应与受试者的签名在同一天。知情同意书应注明版本和制订日期或修改日期。

19 临床试验的质量保证

为了确保本试验能够严格按照临床研究方案进行，在临床试验的整个过程中，临床研究者、申办者双方均应严格按照《药品临床试验质量管理规范》（GCP）的要求进行操作，务必做到试验程序规范，试验数据准确，研究结论可靠。

20 数据处理

20.1 研究者填写数据要求

- 对所有填写了知情同意书并筛选合格进入试验的患者，均须认真而详细地记录病例报告表中的任何项目，不得空项或漏项（无记录的空格划横线）；
- 病例报告表中所有数据需与受试者病历数据核对，保证无误；
- 病例报告表作为原始数据，做任何更正时只能划线，旁注改后数据，并有研究者签名标注日期；
- 化验单复印件粘贴在病例报告表后的化验单粘贴处；
- 对显著偏高或在临床接受范围以外的数据，须加以核实，由研究者做必要说明，请参照病例报告表填写说明。

20.2 数据的可溯源性、病例报告表（CRF）的填写

最原始记录为研究病历以便妥善保存。病例报告表来自研究病历，由研究者填写，每个入选病例必须完成病例报告表。

20.3 统计分析数据的选择

① 全分析集（Full Analysis Set）

按照意向性分析（ITT）原则，对所有入组并至少使用一次药物的全部病例进行疗效分析。对于未能观察到全部治疗过程的病例资料，用最后一次观察数据结转到试验最终结果（LOCF）。

② 符合方案集（Per-protocol Set）

所有符合试验方案、依从性好、试验期间未用禁止用药、完成病例报告表规定填写内容的病例。对缺失数据不进行任何填补（imputation）。本试验药物的疗效分析对PPS进行。

③ 安全性分析集（Safety Analysis Set）

所有入组病例，至少使用过一次试验用药，并有用药后安全性记录的全部患者，均属于安全性分析集。该数据集用于安全性分析。

20.4 统计分析计划

本试验结果主要采用统计描述方法。计量资料列出均数、标准差、中位数、最大值、最小值，计数资料和等级资料列出频数（构成比）、率、可信区间。

所有统计分析将采用SAS 9.2统计分析软件编程计算。所有的统计学检验均采用双侧检验，P值小于或等于0.05将被认为所检验的差别有统计意义，可信区间采用95%的可信度。

① 患者基本特征

计算年龄、身高、体重等定量资料的均数、标准差、中位数、最大值、最小值，性别、ECOG 评分等定性资料列出频数及百分比。

② 耐受性评价

以描述性统计分析为主，列表描述本次试验各剂量组所发生的不良事件及不良反应（其中不良反应定义为“与研究药物关系为‘肯定有关/很可能有关/可能有关’的不良事件”）。实验室检验结果描述试验前正常但治疗后异常的情况以及发生异常改变时与试验药物的关系。分别计算单次给药各剂量组用药前后生命体征（血压、心率、体温、呼吸频率）及实验室指标的均数、标准差、中位数、最小值、最大值，必要时采用配对t检验前后比较；各剂量组间生命体征及实验室指标的变化进行趋势性检验。多次给药组用药后各时间点均和用药前进行比较。

③ 有效性分析

列表描述本次试验临床疗效。

参考文献

1. Siegel, R.L., Miller, K.D. & Jemal, A. Cancer statistics, 2018. *CA Cancer J Clin* **68**, 7-30 (2018).
2. Cardoso, F., *et al.* 1st International consensus guidelines for advanced breast cancer (ABC 1). *Breast* **21**, 242-252 (2012).
3. Goldhirsch, A., *et al.* Strategies for subtypes--dealing with the diversity of breast cancer: highlights of the St. Gallen International Expert Consensus on the Primary Therapy of Early Breast Cancer 2011. *Ann Oncol* **22**, 1736-1747 (2011).
4. Slamon, D.J., *et al.* Studies of the HER-2/neu proto-oncogene in human breast and ovarian cancer. *Science* **244**, 707-712 (1989).
5. Slamon, D.J., *et al.* Human breast cancer: correlation of relapse and survival with amplification of the HER-2/neu oncogene. *Science* **235**, 177-182 (1987).
6. Ponde, N., Brandao, M., El-Hachem, G., Werbrouck, E. & Piccart, M. Treatment of advanced HER2-positive breast cancer: 2018 and beyond. *Cancer Treat Rev* **67**, 10-20 (2018).
7. Velasco-Velazquez, M.A., *et al.* Examining the role of cyclin D1 in breast cancer. *Future Oncol* **7**, 753-765 (2011).
8. Casimiro, M.C., Velasco-Velazquez, M., Aguirre-Alvarado, C. & Pestell, R.G. Overview of cyclins D1 function in cancer and the CDK inhibitor landscape: past and present. *Expert Opin Investig Drugs* **23**, 295-304 (2014).
9. Finn, R.S., *et al.* Palbociclib and Letrozole in Advanced Breast Cancer. *N Engl J Med* **375**, 1925-1936 (2016).
10. Hortobagyi, G.N., *et al.* Ribociclib as First-Line Therapy for HR-Positive, Advanced Breast Cancer. *N Engl J Med* **375**, 1738-1748 (2016).
11. Goetz, M.P., *et al.* MONARCH 3: Abemaciclib As Initial Therapy for Advanced Breast Cancer. *J Clin Oncol* **35**, 3638-3646 (2017).
12. Goel, S., *et al.* CDK4/6 inhibition triggers anti-tumour immunity. *Nature* **548**, 471-475 (2017).
13. Goel, S., *et al.* Overcoming Therapeutic Resistance in HER2-Positive Breast Cancers with CDK4/6 Inhibitors. *Cancer Cell* **29**, 255-269 (2016).

附件一　身体状况评分标准（ECOG）

（东部肿瘤协作组）

活动评分	描述
0	无症状，完全主动活动，及能够进行无限制的活动。
1	有症状，完全能行走，但重体力活动受限，能从事轻的或以坐为主的工作，如轻微家务、办公室工作。
2	有症状，能行走，生活可自理，但不能进行任何的体力活动，约有 50%以上的时间清醒（白天卧床时间<50%）。
3	有症状，有限的生活自理能力，清醒时间卧床或坐椅>50%，但尚未卧床不起。
4	完全失去功能，生活完全不能自理，卧床不起。
5	死亡。

附件二　肌酐清除率计算

Cockcroft-Gault 公式计算肌酐清除率

血清肌酐浓度（mg/dL）:

男性肌酐清除率（mL/min）＝ $\frac{（140－年龄）×（体重）^{a}}{72×血清肌酐}$

女性肌酐清除率（mL/min）＝ $\frac{0.85×（140－年龄）×（体重）^{a}}{72×血清肌酐}$

血清肌酐浓度（μmol/L）:

男性肌酐清除率（mL/min）＝ $\frac{（140－年龄）×（体重）^{a}}{0.81×血清肌酐}$

女性肌酐清除率（mL/min）＝ $\frac{0.85×（140－年龄）×（体重）^{a}}{0.81×血清肌酐}$

a　年龄单位为岁，体重单位为 KG。

附件三　实体肿瘤的疗效评价标准

实体肿瘤的疗效评价标准 1.1 版（节选）

(New Response Evaluation Criteria in Solid Tumors：Revised RECIST Version 1.1)

说明：本附件为内部翻译资料，仅供参考，实际操作中请以英文版为准。

1　背景

略

2　目的

略

3　肿瘤在基线水平的可测量性

3.1　定义

在基线水平上，肿瘤病灶/淋巴结将按以下定义分为可测量和不可测量两种：

3.1.1　可测量病灶

肿瘤病灶：至少有一条可以精确测量的径线（记录为最大径），其最小长度如下：

- CT 扫描 10 mm（CT 扫描层厚不大于 5mm）
- 临床常规检查仪器 10 mm（肿瘤病灶不能用测径仪器准确测量的应记录为不可测量）
- 胸部 X-射线 20 mm
- 恶性淋巴结：病理学增大且可测量，单个淋巴结 CT 扫描短径须≥15 mm（CT 扫描层厚推荐不超过 5 mm）。基线和随访中，仅测量和随访短径。

3.1.2　不可测量病灶

所有其他病灶，包括小病灶（最长径＜10 mm 或者病理淋巴结短径≥10 mm 至＜15 mm）和无法测量的病灶。无法测量的病灶包括：脑膜疾病、腹水、胸膜或者心包积液、炎性乳腺癌、皮肤/肺的癌性淋巴管炎、影像学不能确诊和随诊的腹部包块，以及囊性病变。

3.1.3　关于病灶测量的特殊考虑

骨病灶、囊性病灶和先前接受过局部治疗的病灶需要特别注明：

骨病灶：

- 骨扫描，PET 扫描或者平片不适合于测量骨病灶，但是可用于确认骨病灶的存

在或者消失；

- 溶骨性病灶或者混合性溶骨/成骨病灶有确定的软组织成分，且软组织成分符合上述可测量性定义时，如果这些病灶可用断层影像技术如 CT 或者 MRI 进行评价，那么这些病灶可以作为可测量病灶；
- 成骨病灶属不可测量病灶。

囊性病灶：

- 符合放射影像学单纯囊肿定义标准的病灶，不应因其为定义上的单纯性囊肿，而认为是恶性病灶，既不属于可测量病灶，也不属于不可测量病灶；
- 若为囊性转移病灶，且符合上述可测量性定义的，可以作为是可测量病灶。但如果在同一病人中存在非囊性病灶，应优先选择非囊性病灶作为靶病灶。

局部治疗过的病灶：

- 位于曾放疗过或经其他局部区域性治疗的部位的病灶，一般作为不可测量病灶，除非该病灶出现明确进展。研究方案应详细描述这些病灶属于可测量病灶的条件。

3.2　测量方法说明

3.2.1　病灶测量

临床评价时，所有肿瘤测量都要以公制米制记录。所有关于肿瘤病灶大小的基线评定都应尽量在接近治疗开始前完成，且必须在治疗开始前的 28 天内（4 周）完成。

3.2.2　评价方法

对病灶基线评估和后续测量应采用同样的技术和方法。除了不能用影像学检查，而仅能用临床检查来评价的病灶之外，所有病灶必须使用影像学检查进行评价。

临床病灶：临床病灶只有位于浅表且测量时直径≥10 mm 时才能认为是可测量病灶（如皮肤结节等）。对于有皮肤病灶的患者，建议用含有标尺测量病灶大小的彩色照片作为存档。当病灶同时使用影像学和临床检查评价时，由于影像学更客观且研究结束时可重复审阅，应尽可能选用影像学评价。

胸部 X 片：当肿瘤进展作为重要研究终点时，应优先使用胸部 CT，因为 CT 比 X 线更敏感，尤其对于新发病灶。胸部 X 片检测仅当被测量病灶边界清晰且肺部通气良好时适用。

CT、MRI：CT 是目前用于疗效评价最好的可用可重复的方法。本指导原则对可测量性的定义建立在 CT 扫描层厚≤5 mm 的基础上。如果 CT 层厚大于 5 mm，可测量病灶最小应为层厚的 2 倍。MRI 在部分情况下也可接受（如全身扫描）。

超声：超声不应作为一种测量方法用于测量病灶大小。超声检查因其操作依赖性，在测量结束后不具备可重复性，不能保证不同测量间技术和测量的同一性。如果在试验期间使用超声发现新病灶，应使用 CT 或者 MRI 进行确认。如果考虑到 CT 的放射线暴露，可以使用 MRI 代替。

内窥镜，腹腔镜检查：不建议使用这些技术用于肿瘤客观评价，但这种方法在取得的活检标本时可以用于确认 CR，也可在研究终点为 CR 后复发或手术切除的试验中，用于确认复发。

肿瘤标志物：肿瘤标志物不能单独用来评价肿瘤客观缓解。但如果标志物水平在基线时超过正常值上限，用于评价完全缓解时必须回到正常水平。因为肿瘤标志物因病而异，在将测量标准写入方案中时需考虑到这个因素。有关 CA-125 缓解（复发性卵巢癌）及 PSA（复发性前列腺癌）缓解的特定标准已经发表。且国际妇科癌症组织已制定了 CA-125 进展标准，即将被加入到卵巢癌一线治疗方案的肿瘤客观评价标准中。

细胞学/组织学技术：在方案规定的特定情况下，这些技术可用于鉴定 PR 和 CR(如生殖细胞肿瘤的病灶中常存在残留的良性肿瘤组织)。当渗出可能是某种疗法潜在的副反应（如使用紫杉烷化合物或血管生成抑制剂的治疗），且可测量肿瘤符合缓解或疾病稳定标准时，在治疗过程中肿瘤相关的渗出出现或加重，可通过细胞学技术来确诊，以区分缓解（或疾病稳定）和疾病进展。

4 肿瘤缓解评估

4.1 全部肿瘤和可测量病灶的评估

为评价客观缓解或未来可能的进展，有必要对所有肿瘤病灶肿瘤的总负荷进行基线评估，为后面的测量结果作参照。在以客观缓解作为主要治疗终点的临床方案中，只有在基线时具有可测量病灶的患者才能入选。可测量病灶定义为存在至少一处可测量的病灶。而对于那些以疾病进展（疾病进展时间或固定日期进展程度）为主要治疗终点的试验，方案入选标准中必须明确是仅限于有可测量病灶的患者，还是没有可测量病灶也可以入选。

4.2 靶病灶和非靶病灶的基线记录

基线评估时有超过一个以上可测量病灶时，应记录并测量所有病灶，总数不超过 5 个（每个器官不超过 2 个），作为靶病灶代表所有累及器官（也就是说只有一个或两个累计器官的患者最多选择两个或四个靶病灶作为基线测量病灶）。

靶病灶必须基于尺寸进行选择（最长直径），能代表所有累及器官，且测量必须具有良好的重复性。有时候当最大的病灶不能重复测量时可重新选择一个可重复测量的最大病灶。

淋巴结因其为正常组织且即使没有肿瘤转移仍可为影像察觉而需要特别关注。定义为可测量结节甚至是靶病灶的病理性淋巴结必须符合以下标准：CT 测量短直径≥15 mm。基线只需要检测短直径。放射学家通常借助结节的短直径来判断该结节是否已有肿瘤转移。结节尺寸一般用影像检测的两维数据来表示（CT 用轴平面，MRI 则从轴面、矢状面或冠状面中选择一个平面）。取最小值即为短直径。例如，一个 20 mm× 30 mm 的腹部结节短直径为 20 mm，可视为恶性的、可测量的结节。在这个例子中，20 mm 即是结节的测量值。直径≥10 mm 但<15 mm 的结节不应该视为靶病灶。而<10 mm 的结节则不属于病理结节范畴，不必予以记录和进一步观察。

所有靶病灶的直径经过计算所求之和（包括非结节病灶的最长直径和结节病灶的短直径）将作为基线直径总和上报。如含有淋巴结直径，如上面提到的，只将短直径计算在内。基线直径总和将作为疾病基线水平的参考数值。

其余所有的病灶包括病理淋巴结可视为非靶病灶，无需进行测量，但应在基线评估时进行记录。如记录为“存在”，“缺失”或极少数情况下“明确进展”。广泛存在的靶病灶可与靶器官记录在一起(如大量扩增骨盆淋巴结或大规模肝转移)。

4.3 缓解标准

4.3.1 靶病灶评估

完全缓解（CR）：所有靶病灶消失，全部病理淋巴结(包括靶结节和非靶结节)短直径必须减少至＜10 mm。

部分缓解（PR）：靶病灶直径之和比基线水平减少至少 30%。

疾病进展（PD）：以整个实验研究过程中所有测量的靶病灶直径之和的最小值为参照，直径和相对增加至少 20%（如果基线测量值最小就以基线值为参照）；除此之外，必须满足直径和的绝对值增加至少 5 mm（出现一个或多个新病灶也视为疾病进展）。

疾病稳定（SD）：靶病灶减小的程度没达到 PR，增加的程度也没达到 PD 水平，介于两者之间，研究时可以直径之和的最小值作为参考。

4.3.2 靶病灶评估的注意事项

淋巴结：即使鉴定为靶病灶的淋巴结减小至 10 mm 以内，每次测量时仍需记录与

基线对应的实际短直径的值（与基线测量时的解剖平面一致）。这意味着如果淋巴结属于靶病灶，即使达到完全缓解的标准，也不能说病灶已全部消失，因为正常淋巴结的短直径就定义为＜10 mm。在 CRF 表或其他的记录方式中需在特定位置专门记录靶淋巴节病灶：对于 CR，所有淋巴节短直径必须＜10 mm；对于 PR、SD 和 PD，靶淋巴节短直径实际测量值将被包含在靶病灶直径的和之中。

小到无法测量的靶病灶：临床研究中，基线记录过的所有病灶（结节或非结节）在后面的评估中都应再次记录实际测量值，即使病灶非常小（如 2 mm）。但有时候可能太小导致 CT 扫描出的图像十分模糊，放射科医生也很难定义出确切的数值，就可能报告为“太小而测量不到”。出现这种情况时，在 CRF 表上记录上一个数值是十分重要的。如果放射科医生认为病灶可能消失了，那也应该记录为 0 mm。如果病灶确实存在但比较模糊，无法给出精确的测量值时，可默认为 5 mm。（注：淋巴结出现这种情况的可能性不大，因其正常情况下一般都具有可测量的尺寸，或者像在腹膜后腔中一样常常为脂肪组织所包绕;但是如果也出现这种无法给出测量值的情况,也默认为 5 mm）。5 mm 的默认值源于 CT 扫描的切割厚度（这个值不因 CT 不同的切割厚度值而改变）。由于同一测量值重复出现的几率不大，提供这个默认值将降低错误评估的风险。但需要重申的是，如果放射医生能给出病灶大小的确切数值，即使病灶直径小于 5 mm，也必须记录实际值。

分离或结合的病灶：当非结节性病灶分裂成碎片状时，将各分离部分的最长径加起来计算病灶的直径之和。同样，对于结合型病灶，通过各结合部分间的平面可将其区分开来，然后计算各自的最大直径。但如果结合得密不可分，最长径应取融合病灶整体的最长径。

4.3.3　非靶病灶的评估

这部分对非靶病灶肿瘤的缓解标准进行了定义。虽然一些非靶病灶实际可测量，但无需测量，只需在方案规定的时间点进行定性评估即可。

完全缓解（CR）：所有非靶病灶消失，且肿瘤标记物恢复至正常水平。所有淋巴结为非病理尺寸（短径＜10 mm）。

非完全缓解/非疾病进展：存在一个或多个非靶病灶和/或持续存在肿瘤标记物水平超出正常水平。

疾病进展：已存在的非靶病灶出现明确进展。注：出现一个或多个新病灶也被视为

疾病进展。

4.3.4　关于的非靶病灶进展评估的特别注意事项

关于非靶病灶进展的定义补充解释如下：当患者存在可测量非靶病灶时，即使靶病灶评估为稳定或部分缓解，要在非靶病灶的基础上作出明确进展的定义，必须满足非靶病灶整体的恶化程度已达到必须终止治疗的程度。而一个或多个非靶病灶尺寸的一般性增大往往不足以达到进展标准，因此，在靶病灶为稳定或部分缓解时，仅依靠非靶病灶的改变就能定义整体肿瘤进展的情况几乎是十分稀少的。

当患者的非靶病灶均不可测量时：在一些III期试验中，当入选标准中没有规定必须存在可测量病灶时，就会出现这种情况。整体评估还是参照上文标准，但因为这种情况下没有病灶的可测量数据。非靶病灶的恶化不容易评估（根据定义：必须所有非靶病灶都确实无法测量），因此当非靶病灶改变导致整体疾病负荷增加的程度相当于靶病灶出现疾病进展时，依据非靶病灶作出明确进展的定义，需要建立一种有效的检测方法来进行评估。如描述为肿瘤负荷增加相当于体积额外增加 73%（相当于可测量病灶直径增加 20%）。又比如腹膜渗出从“微量”到“大量”；淋巴管病变从“局部”到“广泛播散”；或在方案中描述为“足够至改变治疗方法”。例子包括胸膜渗出液从痕量到大量，淋巴受累从原发部位向远处扩散，或者在方案中可能被描述为“有必要进行治疗方面的改变”。如果发现有明确的进展，该患者应该在那个时点总体上视为疾病进展。最好具有客观标准可适用于不可测量的病灶的评估，注意，增加的标准必须是可靠的。

4.3.5　新病灶

新的恶性病灶的出现预示着疾病的进展；因此针对新病变的一些评价是非常重要的。目前没有针对影像学检测病灶的具体标准，然而一种新的病灶的发现应该是明确的。比如说，进展不能归因于影像学技术的不同，成像形态的改变，或者肿瘤以外的其它病变（如：一些所谓新的骨病灶仅仅是原病灶的治愈，或原病灶的复发）。当病人的基线病灶出现部分或完全反应时，这一点非常重要的，例如：一例肝脏病灶的坏死可能在 CT 报告上定为新的囊性病变，而其实不是。

在随访中已检测到的而在基线检查中未发现的病灶将视为新的病灶，并提示疾病进展。例如一个在基线检查中发现有内脏病灶的患者，当他做 CT 或 MRI 的头颅检查时发现有转移灶，该患者的颅内转移病灶将被视为疾病进展的依据，即使他在基线检查时并未做头颅检查。

如果一个新的病灶是不明确的，比如因其形态小所致，则需要进一步的治疗和随访评价以确认其是否是一个新的病灶。如果重复的检查证实其是一个新的病灶，那么疾病进展的时间应从其最初的发现的时间算起。

病灶进行 FDG-PET 评估一般需要额外的检测进行补充确认，FDG-PET 检查和补充 CT 检查结果相结合评价进展情况是合理的（尤其是新的可疑疾病）。新的病灶可通过 FDG-PET 检查予明确的，依据以下程序执行：

基线 FDG-PET 检查结果是阴性的，接下来随访的 FDG-PET 检查是阳性的，表明疾病的进展。

没有进行基线的 FDG-PET 检查，后续的 FDG-PET 检查结果是阳性的：

如果随访的 FDG-PET 阳性检查结果发现的新的病变灶与经 CT 检查结果相符，证明是疾病进展。

如果随访的 FDG-PET 的阳性检查结果发现的新的病变灶未能得到 CT 检查结果的确认，需再行 CT 检查予以确认（如果得到确认，疾病进展时间从前期 FDG-PET 检查发现异常算起）。

如果随访的 FDG-PET 的阳性检查结果与经 CT 检查已存在的病灶相符，而该病灶在影像学检测上无进展，则疾病无进展。

4.4　最佳整体疗效评价

最佳整体疗效评价是从试验开始至试验结束的最佳疗效记录，同时要把任何必要条件考虑在内以便确认。有时疗效反应出现在治疗结束后，因此方案应该明确治疗结束后的疗效评价是否考虑在最佳整体疗效评价之内。方案必须明确任何进展前新的治疗如何影响最佳疗效反应。患者的最佳疗效反应主要依赖目标病灶和非目标病灶的结果以及新病灶的表现情况。此外，还依赖于试验性质、方案要求及结果衡量标准。具体来说，在非随机试验中，疗效反应情况是首要目标，PR 或 CR 的疗效确认是必须的，以确认哪个是最佳整体疗效反应。

4.4.1　时间点反应

假设在每个方案的具体时间点上都会有疗效反应发生。表 1 将提供一个基线水平上疾病可测量的患者人群其在每个时间点上的总体疗效反应的总结。

表 1　时间点反应：有靶病灶的受试者（包括或者不包括非靶病灶）

目标病灶	非目标病灶	新病灶	总缓解
CR	CR	非	CR

CR	非 CR/非 PD	非	PR
CR	不能评估	非	PR
PR	非进展或者不能完全评估	非	PR
SD	非进展或者不能完全评估	非	SD
不能完全评估	非进展	非	NE
PD	任何情况	是或否	PD
任何情况	PD	是或否	PD
任何情况	任何情况	是	PD

CR=完全缓解，PR=部分缓解，SD=疾病稳定，PD=疾病进展，NE=不能评估

如果患者无可测量病灶（无目标病灶），评估可参见表 2。

表 2　时间点反应-仅有非目标病灶的受试者

非目标病灶	新病灶	总缓解
CR	非	CR
非 CR 或者非 PD	非	非 CR 或非 PD[a]
不能完全评估	非	不能评估
不能明确的 PD	是或否	PD
任何情况	是	PD

a：对于非目标病灶，"非 CR/非 PD"是指优于 SD 的疗效。由于 SD 越来越多作为评价疗效的终点指标，因而制定非 CR/非 PD 的疗效，以针对未规定无病灶可测量的情况。

4.4.2　评估缺失和不可评价说明

如果在某个特定时间点上无法进行病灶成像或测量，则该患者在该时间点上无法评价。如果在一个评价中只能对部分病灶进行评价，通常这种情况视为在那个时间点无法评价，除非有证据证实缺失的病灶不会影响指定时间点的疗效反应评价。这种情况很可能发生在疾病进展的情况。例如：一个患者在基线水平有 3 个总和为 50 mm 的病灶，但是随后只有 2 个病灶可评价，总和为 80 mm，该患者将被评价为疾病进展，不管缺失的病灶影响有多大。

4.4.3　最佳总缓解：全部时间点

一旦患者的所有资料都具备，其最佳总缓解可以确定。

当研究不需要对完全或部分疗效反应进行确认时最佳总缓解的评估：试验中最佳疗效反应是所有时间点上的最佳反应（例如：一个患者在第一周期疗效评价为 SD，第二周期评价为 PR，最后一周期评价为 PD，但其最佳总缓解评价为 PR。当最佳总缓解评价为 SD 时，其必须满足方案所规定的从基线水平算起的最短时间。如果没有达到最短

时间的标准，即使最佳总缓解评价为 SD 也是不认可的，该患者的最佳总缓解将视随后的评价而定。例如：一个患者第一周期评价为 SD，第二周期为 PD，但其未达到 SD 的最短时间要求，其最佳总缓解评价为 PD。同样的患者在第一周期评价为 SD 后失访将被视为不可评价。

当研究需要对完全或部分疗效反应进行确认时最佳总缓解的评估：只有当每一个受试者符合试验规定的部分或者完全缓解标准而且在方案中特别提及的在随后的时间点（一般是四周后）再次做疗效确认后才能宣称是完全或者部分缓解。在这种情况下，最佳总缓解见表 3 的说明。

表 3　CR 和 PR 疗效需要确认的最佳总缓解

首个时间点总缓解	随后时间点总缓解	最佳总缓解
CR	CR	CR
CR	PR	SD，PD 或 PR[a]
CR	SD	如果 SD 持续足够时间则为 SD，否则应为 PD
CR	PD	如果 SD 持续足够时间则为 SD，否则应为 PD
CR	NE	如果 SD 持续足够时间则为 SD，否则应为 NE
PR	CR	PR
PR	PR	PR
PR	SD	SD
PR	PD	如果 SD 持续足够时间则为 SD，否则应为 PD
PR	NE	如果 SD 持续足够时间则为 SD，否则应为 NE
NE	NE	NE

CR=完全缓解，PR=部分缓解，SD=疾病稳定，PD=疾病进展，NE=不能评估。

a：如果在第一个时间点 CR 真正出现，在随后的时间点出现的任何疾病，那么即便相对于基线该受试者疗效达到 PR 标准，其疗效评价在之后的时间点仍然为 PD（因为在 CR 之后疾病将再次出现）。最佳缓解取决于是否在最短的治疗间隔内出现 SD。然而有时第一次评价为 CR，但随后的时间点扫描提示小病灶似乎依然出现，因而实际上受试者疗效在第一个时间点应该是 PR 而不是 CR。在这种情况下，首次 CR 判断应该被修改为 PR，同时最好的反应是 PR。

4.4.4　疗效评估的特别提示

当结节性病灶被包括在总的靶病灶评估中，同时该结节大小缩小到“正常”大小时（<10 mm），它们依然会有一个病灶大小扫描报告。为了避免过高评估基于结节大小增加所反映的情况，即便是结节正常，测量结果也将被记录。正如前面已经提及的，这就意味着疗效为完全缓解的受试者，CRF 表上也不会记录为 0。

若试验过程中需要进行疗效确认，重复的“不可测量”时间点将使最佳疗效评估变得复杂。试验的分析计划必须说明，在确定疗效时，这些缺失的数据/评估可以被解释清楚。比如，在大部分试验中，可以将某受试者 PR-NE-PR 的反应作为得到了疗效确认。

当受试者出现健康情况整体恶化要求停止给药治疗，但是没有客观证据证明时，应该被报道为症状性进展。即便在治疗终止后也应该尽量去评估客观进展的情况。症状性恶化不是客观反应的评估描述：它是停止治疗的原因。那样的受试者的客观反应情况将通过表 1 到 3 所示的目标和非目标病灶情况进行评估。

定义为早期进展，早期死亡和不可评估的情况是研究特例，且应该在每个方案中进行明确的描述（取决于治疗间期和治疗周期）。

在一些情况下，从正常组织中辨别局部病灶比较困难。当完全缓解的的评估基于这样的定义时，推荐在进行局部病灶完全缓解的疗效评估前进行活检。当一些受试者局部病灶影像学检测结果异常被认为是代表了病灶纤维化或者疤痕形成时，FDG-PET被当作与活检相似的评估标准，用来对完全缓解进行疗效确认。在此种情况下，应该在方案中对FDG-PET的应用进行前瞻性描述，同时以针对此情况专科医学文献的报告作为支持。但是必须意识到的是由于FDG-PET和活检本身的限制性（包括二者的分辨率和敏感性高低），将会导致完全缓解评估时的假阳性结果。

对于不明确的进展发现（如非常小的不确定的新病灶；原有病灶的囊性变或坏死病变）治疗可以持续到下一次评估。如果在下一次评估中，证实了疾病进展，进展日期应该是先前出现疑似进展的日期。

4.5　肿瘤重新评价的频率

治疗期间肿瘤重新评价的频率决定于治疗方案，并应与治疗的类型和日程安排相符。但是在治疗的受益效果不清楚的Ⅱ期试验中，每 6~8 周（时间设计在一个周期的结束点）进行随访是合理的，在特殊方案或情况下可调整时间间隔长度。方案应该具体指明哪些组织部位需要进行基线水平的评估（通常是那些最可能与所研究肿瘤类型的转移病变密切相关的组织部位）和评价重复的频率。正常情况下，靶病灶和非靶病灶在每次评估时都应进行评价，在一些可选择的情形下，某些非目标病灶评价频率可以小一些，例如，目标疾病的疗效评价确认为 CR 或怀疑有骨性病变进展时才需重复骨扫描。

治疗结束后，重新评价肿瘤取决于是否把缓解率或者是到出现某一事件（进展/死亡）的时间作为临床试验终点。如为出现某一事件时间（如：TTP/DFS[1]/PFS）则需要进行方案中规定的常规重复评价。特别是在随机比较试验中，预定的评价应该列在时间表内（如：治疗中的 6~8 周，或治疗后的 3~4 个月），不应受到其他因素的影响，如治疗延迟、给药间隔和任何其他在疾病评价时间选择上可能导致治疗臂不均衡的事件等。

1

4.6 疗效评估/缓解期的确认

4.6.1 确认

对于以疗效为主要研究终点的非随机临床研究，必须对 PR 和 CR 的疗效进行确认，以保证疗效不是评价失误的结果。这也允许在有历史数据的情况下，对结果进行合理的解释，但这些试验的历史数据中的疗效也应进行过确认。但在所有其他情况下，如随机试验（Ⅱ或Ⅲ期）或者以疾病稳定或者疾病进展为主要研究终点的研究中，不再需要疗效确认，因为这对于试验结果的解释没有价值。然而取消疗效确认的要求，就会使防止偏移作用的中心审查显得更加重要，特别是在非盲态实验研究中。

SD 的情况下，在试验开始后的最短时间间隔内（一般不少于 6~8 周），至少有一次测量符合方案中规定的 SD 标准。

4.6.2 总缓解期

总缓解期是从测量首次符合 CR 或 PR（无论哪个先测量到）标准的时间到首次真实记录疾病复发或进展的时间（把试验中记录的最小测量值作为疾病进展的参考）。总完全缓解时间是从测量首次符合 CR 标准的时间到首次真实记录疾病复发或进展的时间。

4.6.3 疾病稳定期

是从治疗开始到疾病进展的时间（在随机化试验中，从随机分组的时间开始），以试验中最小的总和作为参考（如果基线总和最小，则作为 PD 计算的参考）。疾病稳定期的临床相关性因不同研究和不同疾病而不同。如果在某一特定的试验中，以维持最短时间稳定期的病人比例作为研究终点，方案应特别说明 SD 定义中两个测量间的最短时间间隔。

注意：缓解期、稳定期以及 PFS 受基线评价后随访频率的影响。定义标准随访频率不属于本指导原则范围。随访频率应考虑许多因素，如疾病类型和分期、治疗周期及标准规范等。但若需进行试验间的比较，应考虑这些测量终点准确度的限制。

4.7 PFS/TTP

4.7.1 Ⅱ期临床试验

本指导原则主要关注Ⅱ期临床试验中客观缓解作为研究终点的应用。在某些情况下，缓解率可能不是评价新药/新方案潜在抗癌活性的最优选择。在这些情况下，分界时间点上的 PFS/PPF 可认为是提供新药生物活性的原始信号的合适替代指标。但是很

明显，在一个非对照试验中，这些评价会受到质疑，因为貌似有价值的观察可能与病人的筛选等生物学因素有关，而非药物干预的作用。因此，以这些作为研究终点的Ⅱ期临床试验最好设计随机对照。但某些肿瘤的临床表现始终如一（通常一直状况差），非随机试验也是合理的。但是在这些情况下，因缺少阳性对照，评估预期 PFS 或 PPF[2]时，需小心记录疗效证据。
