# Supplementary material for: Dalpiciclib combined with pyrotinib and endocrine therapy in women with ER-positive, HER2-positive advanced breast cancer: A prospective, multicenter, single-arm, phase 2 trial
Source: PLoS Med. 2025 Jul 31;22(7):e1004669. doi: 10.1371/journal.pmed.1004669 (PMC12312931; doi:10.1371/journal.pmed.1004669)
Supplement: S1 Text — (DOCX) [file pmed.1004669.s010.docx]

**S1 Text. Ethics Approval.**

The study was conducted in accordance with ethical guidelines and was approved by the respective ethics committees. The approval numbers for each committee are listed below:

| Ethics Committee Name | Approval Number |
| --- | --- |
| Fudan University Shanghai Cancer Center | 1811193-9 |
| Hubei Cancer Hospital | 2021-084 |
| The Affiliated Tumour Hospital of Harbin Medical University | 2021-176-R |
| Nanchang People's Hospital | 2021-014 |
| Affiliated Hospital of Nantong University | 2022-K008 |
| Tumour Hospital of Mudanjiang City | 2022-001-001 |
