# Supplementary material for: Dalpiciclib combined with pyrotinib and endocrine therapy in women with ER-positive, HER2-positive advanced breast cancer: A prospective, multicenter, single-arm, phase 2 trial
Source: PLoS Med. 2025 Jul 31;22(7):e1004669. doi: 10.1371/journal.pmed.1004669 (PMC12312931; doi:10.1371/journal.pmed.1004669)
Supplement: S2 Text — (DOCX) [file pmed.1004669.s011.docx]

**Summary of Protocol Amendments**

Please refer to the comparison document for protocol Version 3.0 (dated 21 Dec 2022) vs.

protocol Version 1.1 (dated 5 Feb 2021) for actual changes in text.

| **Section # and Title** | **Description of Change** | **Brief Rationale** |
| --- | --- | --- |
| Protocol name  Synopsis  Section 3.1 Primary Study Objective  Section 5 Overall Study Design | Changed anastrozole to endocrine therapy. | Added endocrine therapy of fulvestrant. Changed anastrozole to endocrine therapy.  This adjustment was made because some patients had previously received aromatase inhibitors in the adjuvant or first-line setting and experienced disease progression, indicating endocrine resistance. As a result, continued use of letrozole as per the original protocol was no longer appropriate for these patients. Fulvestrant was therefore introduced as an alternative endocrine partner. |
| Synopsis  Schedule of event  Background  Section 1.1 Investigational Product  Section 3.1 Primary Study Objective  Section 4.1 Name and Source  Section 5 Overall Study Design | Updated the English name of SHR6390 to dalpiciclib. | Updated the English name. |
| Synopsis  Section 6.5 Planned Sample Size  Section 8.1 Subjects and Sample Size | Adjusted the Phase II sample size. | Modifications were made for the addition of fulvestrant, recalculated the sample size based on Phase I results. |
| Synopsis  Section 8.2 Inclusion Criteria | Updated inclusion criterion #5 and, merged with inclusion criterion #2，Added the premenopausal/perimenopausal women and specify treatment for premenopausal or perimenopausal women. | Modifications are for  Clarification. |
| Synopsis  Dosage and Administration | Added Study Design Schema | Modifications are for  clarification. |
| Synopsis  Section 8.2 Inclusion Criteria | Updated inclusion criterion #6 (final #5)，Subjects who were resistant to aromatase inhibitors were added to the inclusion criteria, and fluvestrant was specified as the endocrine therapy. | Modifications were made for the addition of fulvestrant. |
| Synopsis  Section 1.1 Investigational Product  Section 4.1 Name and Source  Section 4.2 Dosage Form and Specifications  Section 4.4 Administration  Section 8.2 Inclusion Criteria | Added Study drug fulvestrant | Modifications were made for the addition of fulvestrant. |
| Synopsis  Section 8.3 Exclusion Criteria | Updated exclusion criterion #1 and #2 (final #1), Patients with meningeal metastasis or active brain parenchymal metastasis could be excluded and clinically stable brain metastases could be included. | Updated based on current research progress. |
| Background | Updated phase 1 dose exploration data | Updated based on ongoing  review of data from the Phase I study. |
| Section 2.2 Clinical Studies SHR6390 | Update SHR6390 clinical research | Updated based on ongoing  study of SHR6390. |
