# Supplementary material for: Dalpiciclib combined with pyrotinib and endocrine therapy in women with ER-positive, HER2-positive advanced breast cancer: A prospective, multicenter, single-arm, phase 2 trial
Source: PLoS Med. 2025 Jul 31;22(7):e1004669. doi: 10.1371/journal.pmed.1004669 (PMC12312931; doi:10.1371/journal.pmed.1004669)
Supplement: Attachment — Submitted filename: Point to point response letter.docx [file pmed.1004669.s015.docx]

Dear Editor,

We sincerely appreciate the time and effort that you and the reviewers have dedicated to evaluating our manuscript, “*Dalpiciclib combined with pyrotinib and endocrine therapy in women with ER-positive, HER2-positive advanced breast cancer (PLEASURABLE): a multicentre, single-arm, phase 2 trial*” (PMEDICINE-D-24-03621R1). We are grateful for the insightful comments and constructive feedback, which have significantly helped us improve the clarity and impact of our study.

**Updates and Administrative Items:**

We have updated the dataset with new information collected before the submission deadline (December 11, 2024), primarily including long-term efficacy data such as PFS and DOR. These updates have been incorporated into the revised version of the manuscript.

Some of our co-authors have reported that they did not receive the emails regarding the confirmation of competing interests and author status, or the provided links have expired. We kindly request that these emails be resent to the authors who have not yet confirmed, to ensure all authors can complete the required confirmations and declarations.

**Responses to Comments:**

In response to the editor’s and reviewers’ comments, we have carefully revised our manuscript. Below, we provide detailed point-by-point responses to the comments:

**Editor’s Comment:**

At present, we feel that it is not clear what the research question and aim of your study is/was. Given that PLOS Medicine publishes articles for a wide audience, it is necessary to clearly state the need for your study in the Abstract Background section. In the Introduction, you should address previous research and explain the need for and potential importance of your study (How is it different from previous trials?). You should also indicate how your study is novel, how you have determined this, and what may be novel about the treatment regimen chosen. In addition, with a global audience in mind, we feel that it is currently not clear what the standard of care is in China and how this regimen compares to it (e.g. what are the potential advantages and disadvantages?). You should also provide details on whether the standard of care varies between institutions in China and how the standard of care differs from other settings (e.g. Europe, USA).

**Response:**

Thank you for your feedback. The research question and aim of the study have already been clearly stated in the Abstract Background section. As stated in the Abstract Background, HER2-targeted therapy combined with endocrine therapy offers modest efficacy for ER-positive, HER2-positive breast cancer. Adding CDK4/6 inhibitors may improve treatment durability, providing a potential chemotherapy-sparing option, though its role in frontline treatment is still unclear. We studied dalpiciclib with pyrotinib and endocrine therapy in patients receiving first- or second-line treatment for ER-positive, HER2-positive advanced breast cancer (ABC). (P2, L24-31)

The introduction has been revised based on your suggestions, addressing standard treatments, prior research, unmet clinical needs, and highlighting the innovation of this study. A brief summary is provided below: The combination of HER2-targeted therapies, trastuzumab and pertuzumab, with chemotherapy has made significant advances in HER2+ advanced breast cancer, improving survival and establishing itself as the standard of treatment worldwide, irrespective of ER status. However, not all patients are suitable for chemotherapy. Considering the bidirectional crosstalk between the HER2 and ER pathways in ER+/HER2+ breast cancer, chemotherapy-free regimens combining anti-HER2 with endocrine therapy have been actively explored in this subtype. Previous studies have modestly improved outcomes with HER2 monotherapy or HER2 dual-target therapy with endocrine therapy. Further exploration in this area is still warranted. The addition of cyclin-dependent kinase 4/6 (CDK4/6) inhibition to HER2-targeted therapy and endocrine therapy has shown efficacy in ER-positive/HER2-positive later-line ABC, providing a potential chemotherapy-free alternative. The benefit of the triple-target combination in advanced front-line treatment is still unknown and warrants further exploration. This study is the first to explore the combination of CDK4/6 inhibitor dalpiciclib with HER2-targeted pyrotinib and endocrine therapy in frontline ER+/HER2+ advanced breast cancer. (P6-P7, L87-127)

Regarding potential concerns about treatment strategies, we would like to clarify the standard treatment approach for ER+/HER2+ ABC. In China, the treatment of ER+/HER2+ ABC follows the treatment principles for HER2+ breast cancer as outlined in the CSCO guidelines(1). First-line standard therapy primarily includes the THP regimen (docetaxel, trastuzumab, and pertuzumab) based on the CLEOPATRA, with trastuzumab plus chemotherapy as an alternative. Second-line standard treatment includes pyrotinib plus capecitabine as the preferred option, with T-DM1 as an alternative. Following the approval of the PHILA and DESTINY-Breast03 studies in China (which occurred after the study enrollment phase), the first-line standard of care now includes the TH + pyrotinib regimen (docetaxel, trastuzumab, and pyrotinib), while T-DXd has become one of the preferred second-line treatments. However, its limited availability and high cost make it unaffordable for many patients in China. Anti-HER2 combined with endocrine therapy is recommended for cases where chemotherapy is not tolerated, or as a maintenance therapy following chemotherapy. Referring to the NCCN(2) and ESMO(3) guidelines, treatment for ER+/HER2+ ABC in China is similar to other settings (e.g., Europe, USA), where anti-HER2 therapy combined with chemotherapy is the standard treatment. Due to the varying approval status of HER2 TKIs in different regions, treatments differ. For example, pyrotinib combined with capecitabine, pyrotinib combined with trastuzumab, and docetaxel have only been approved in China. Regarding endocrine therapy, the combination of anti-HER2 therapy with endocrine treatment is recommended in ER+/HER2+ ABC when chemotherapy is not suitable or as maintenance therapy following chemotherapy. However, combining endocrine therapy with anti-HER2 therapy offers better safety compared to chemotherapy, and the potential for improved efficacy addresses an unmet clinical need, warranting further exploration of this combination in targeted therapies. In this study, the efficacy of the treatment regimen was similar to the standard, as mentioned in the discussion (P16-P17, L313-334). The safety profile is manageable, with a lower incidence of gastrointestinal and nervous system disorders compared to HER2-targeted therapy combined with chemotherapy. The regimen offers the convenience of non-intravenous administration, presenting a potential chemotherapy-free treatment option for ER+/HER2+ advanced breast cancer. However, the main limitation is that these results are based on a small sample size, and further validation with a larger cohort is needed (P16-P19, L313-388).

**Editor’s Comment 1:**

1) The regimen is used in both first and second line therapy - what did the women who received other therapy receive, was this also endocrine/HER2 targeted?

**Response:**

Thank you for the valuable comment. As stated in the previous response, in China, the treatment of ER+/HER2+ ABC follows the treatment principles for HER2+ breast cancer as outlined in the CSCO guidelines, typically consists of HER2-targeted therapies combined with chemotherapy. Anti-HER2 combined with endocrine therapy is recommended for cases where chemotherapy is not tolerated, or as a maintenance therapy following chemotherapy (P6 L87-90, L94-96).

**Editor’s Comment 2:**

2) What is the standard of care and how does this regimen differ?

**Response:**

Thank you for the valuable comment. As stated in the previous response, the treatment of ER+/HER2+ ABC follows the treatment principles for HER2+ breast cancer. First-line standard therapy primarily includes docetaxel, trastuzumab, and pertuzumab (ORR 79%, PFS 16.5 months), with trastuzumab plus chemotherapy as an alternative. Second-line standard treatment includes pyrotinib plus capecitabine as the preferred option (ORR 67%, PFS 12.5 months), with T-DM1(ORR 43.6%, PFS 9.6 months) as an alternative. Following the approval of the PHILA and DESTINY-Breast03 studies in China (which occurred after the study enrollment phase), the first-line standard of care now includes docetaxel, trastuzumab, and pyrotinib (ORR 83% and PFS 24.3 months), while T-DXd has become one of the preferred second-line treatments. however, the limited availability of T-Dxd (ORR 79%, PFS 28.8 months) and its high cost make it unaffordable for many patients in China. If chemotherapy is not tolerated, it is recommended to use anti-HER2 therapy in combination with endocrine treatment. In China, the combination of trastuzumab plus endocrine therapy in the SYSUCC-002 study resulted in PFS of 19.2 months, with an ORR of 37.2%. As mentioned in the discussion, the efficacy of the study treatment regimen (ORR 70.2% and PFS 22.0 months in front line) was similar to the standard and the safety profile appeared better than chemotherapy. It offers the convenience of non-intravenous administration, presenting a potential chemotherapy-free treatment option for ER+/HER2+ advanced breast cancer (P16-P19, L313-388).

**Editor’s Comment 3:**

3) Why was this particular endocrine therapy regimen chosen? How much influence did physician choice have on the treatment protocol?

**Response:**

Thank you for your valuable question. Considering the patient's previous use of aromatase inhibitor treatment during the adjuvant phase, the choice of endocrine therapy for advanced disease was made based on the agents the patient had already received and/or progressed on. According to the definition of aromatase inhibitor resistance outlined in the protocol, if aromatase inhibitor resistance was determined, fulvestrant was used, whereas if the patient was not resistant to aromatase inhibitors, letrozole was chosen. The researchers did not make a free choice in this regard but followed the protocol's guidelines to determine which endocrine therapy was needed for the patient (P9 L168-172).

**Editor’s Comment 4:**

4) The research question needs to be clarified and why it might be relevant in this treatment context.

**Response:**

Thank you for your insightful comment. The research question in this study focuses on evaluating the efficacy and safety of a chemotherapy-free regimen in patients with ER-positive, HER2-positive advanced breast cancer. This is particularly relevant in the current treatment context, as new therapeutic strategies are needed to improve outcomes for patients who are not candidates for traditional chemotherapy or for those who wish to avoid chemotherapy. By investigating the combination of oral agents and targeted therapies, this study aims to address the growing need for less toxic, yet effective, treatment options for this patient population (P7 L111-113, L125-127).

**Editor’s Comment 5:**

5) How does the choice of treatment regimen in your study compare with other settings worldwide?

**Response:**

Thank you for your insightful comment. Referring to current international guidelines, the treatment for ER+/HER2+ ABC in China is similar to other settings (e.g., Europe, USA), except for regimens containing pyrotinib, which are specific to China. Current international guidelines primarily recommend chemotherapy-based regimens for ER+/HER2+ ABC, including taxane plus trastuzumab and pertuzumab (ORR 80.2%, PFS 18.7 months) as first-line treatment, followed by T-Dxd (ORR 79%, PFS 28.8 months) in second lines. Patients unsuitable for chemotherapy, it is recommended to use anti-HER2 therapy in combination with endocrine treatment (4.8 to 14.1 months). The efficacy of the study treatment regimen (ORR 70.2% and PFS 22.0 months in front line) was similar to the standard, except for T-Dxd, and the safety profile appeared better than chemotherapy. It offers the convenience of non-intravenous administration, presenting a potential chemotherapy-free treatment option for ER+/HER2+ advanced breast cancer(P16-P19, L313-388).

-----------------------------------------------------------

**Comments from the reviewers:**

**Reviewer #1:** The author evaluated the non-intravenous (also chemo-free) regimen for HR+/HER2+ breast cancer. The regimen has a very good response rate, long PFS, with clinical managable toxicity, which could provide a good clinical option in further clincial practice or research. This paper is well prepared. I would recommend "ACCEPT" for this paper after revision with 2 additional questions.

**Reviewer #1, Comment 1:**

1. The explonary analysis of predictive factor for this regimen should be with caution due to its limited number of patients, especially for these patients with certain gene mutations (subgroup of a subgroup analysis). The mutaion rate of ERBB2 is very high for these patients receiving ctDNA sequecing analysis, is this HER2 amplification or mutation?

**Response:**

Thank you for your thoughtful comments. We acknowledge that the exploratory analysis was limited by the small sample size, particularly in the subgroups with specific gene mutations. The mutation status and efficacy data of the subjects are provided in eTable 3 in the Supplement. Regarding the mutation rate of ERBB2, among the 16 patients tested, 11 (68.8%) had ERBB2 mutations. Of these, 10 cases (62.5%) showed HER2 amplification, while 1 case (6.3%) exhibited a missense mutation. We have updated the manuscript to specify the high mutation rate of ERBB2 (P15, L299-300) and clarified that, due to the limited sample size, no definitive conclusions can be drawn (P15, L301-302).

**Reviewer #1, Comment 2:**

2. Any pre-medication for this regimen, if has, the author needs to add this point in the manscript.

**Response:**

Thank you for your valuable comment. Regarding the pre-medication for this regimen, we would like to clarify that there were no pre-medications used for this treatment protocol. We have added a note in the manuscript to clearly indicate this information (P10, L175-176).

**Reviewer #2:** In this study, Zhang et al. aimed to evaluate the efficacy and safety of a chemotherapy-free regimen that combines dalpiciclib with pyrotinib and endocrine therapy for patients undergoing first- or second-line treatment for ER-positive and HER2-positive advanced breast cancer. Additionally, they conducted a biomarker analysis using 68Ga-HER2 PET/CT imaging and genomic testing. The study demonstrated promising results for a more convenient, purely outpatient treatment regimen. If validated in a phase III trial, it might become the new standard of care.

**Reviewer #2, Comment 1:**

1.My main concern is the varying efficacy observed in similar yet distinct treatment regimens. In Yan Min's study, the median progression-free survival (PFS) for the dalpiciclib and pyrotinib combination is a modest 11 months, with the HR+ subgroup faring even worse at just 9.1 months. In Ouyang Quchang's study, however, the median PFS for the pyrotinib and endocrine therapy combination is more promising at 13.7 months. Moreover, in Wang Shusen's study focusing on the HER2+ brain metastasis patient population, the median PFS for the regimen comprising trastuzumab, pyrotinib, palbociclib, and endocrine therapy is a relatively lower 10.6 months. These treatment strategies, while different, exhibit a significant disparity in their efficacy.

**Response:**

Thank you for your comment and for highlighting the varying efficacy observed in different treatment regimens. We acknowledge your concern regarding the discrepancies in progression-free survival (PFS) across similar yet distinct treatment strategies (P18, L350). In the discussion section (line), we address the varying efficacy levels observed with different treatment strategies. As pointed out by the reviewer, "These treatment strategies, while different, exhibit a significant disparity in their efficacy." This disparity is evident across studies with distinct regimens, such as those using two-drug combinations (pyrotinib and dalpiciclib or pyrotinib and endocrine therapy) with PFS ranging from 9.1 months to 13.7 months, versus our three-drug combination (pyrotinib, dalpiciclib, and endocrine therapy), which showed a PFS of 20.3 months. We hypothesize that triple targeting of ER, HER2, and CDK4/6 in ER-positive and HER2-positive breast cancer could be a more effective chemotherapy-free treatment strategy than a two-agent combination alone (P18, L355-358).

Regarding Wang Shusen’s study, while it involves a four-drug combination (trastuzumab, pyrotinib, palbociclib, and endocrine therapy), it specifically targets the brain metastasis patient population. In contrast, our study includes a limited number of brain metastasis cases (8/48), and the PFS data for this subgroup remains immature, preventing us from drawing definitive conclusions. Consequently, we have not discussed this subgroup in detail (P18, L358-362).

**Reviewer #2, Comment 2:**

2.The study screened 51 participants and enrolled 48 patients, of whom 3 were excluded: two because they did not meet the inclusion criteria, but the rest one the investigators did not specify why she were excluded.

**Response:**

Thank you for your comment. One of the three excluded participants was due to patient withdrawal, which was not previously mentioned. We have now updated the Fig 1 to reflect this reason for exclusion.

**Reviewer #2, Comment 3:**

3.In the Figure 1, is the "^" redundant?

**Response:**

Thank you for your helpful reminder regarding the “^” symbol in Fig 1. We have reviewed the figure and agree that the symbol is redundant. We have removed it and made the necessary adjustments to the figure.

**Reviewer #3:** Thanks for the opportunity to read your manuscript. My role is statistical reviewer, so I have focused on the design, data, and analysis that are presented. I have put general comments first, followed by questions relevant to a specific section of the manuscript (with a page/line reference).

This manuscript presents a Phase II, single-arm study of patients with advanced HR+/HER2+ breast cancer, examining if the addition of CDK4/6 inhibitors to current treatment with anti-HER2/endocrine therapies shows evidence of efficacy. Patients were recruited from 6 large sites and includes patients with an ER2 and HER2 positive breast cancer. Treatment was based on data from a phase Ib study. Main study outcome was objective response rate (RECIST 1.1 guidelines), with a range of a secondary endpoints. The analysis was simple but appropriate for a single-arm design, with the estimates of the endpoints estimated with 95% confidence intervals (Clopper-Pearson for binary outcomes). ORR was 70.2%, with most patients achieving a partial response. Key components of the study match between the manuscript, protocol and study registration.

**Reviewer #3, Comment 1:**

I suspect it is unlikely to make a difference here, but simulation research suggests a liberal bias in the Clopper-Pearson binomial confidence intervals. For the main study outcome, could you confirm the interval is similar when calculated with a Wilson Score Interval calculation?

**Response:**

Thank you for your thoughtful suggestion. We have computed the confidence intervals for the key endpoints using both the Clopper-Pearson and Wilson Score methods, as you recommended. The results from both methods showed minimal differences, confirming the robustness of our findings. We have provided the comparison of the intervals in the table for your reference. Based on this verification, we conclude that both methods yield consistent results for the observed proportions.

| 95% confidence interval | Clopper-Pearson | Wilson Score |
| --- | --- | --- |
| ORR | 0.55-0.83 | 0.56-0.81 |
| CBR | 0.74-0.95 | 0.75-0.94 |

**Reviewer #3, Comment 2:**

Could a summary in the appendix be provided about the changes to the study protocol and the rationale for these from the first version to the final version? It looks like in the study registration there are less details about the age-based inclusion criteria (e.g. participants under <60 having additional inclusion criteria).

**Response:**

Thank you for your insightful comment. We will provide a summary in S1 Protocol detailing the changes made to the study protocol, along with the rationale behind these revisions from the first version to the final version.

Regarding the age-based inclusion criteria, the details are less explicitly stated in the manuscript. This is because both premenopausal and postmenopausal women, as well as those in the perimenopausal period, were eligible for inclusion. The key distinction lies in whether concurrent LHRH agonist therapy is required, as outlined in the protocol. Specifically, premenopausal and perimenopausal patients are required to receive LHRH agonists. For patients under 60 years of age, the differentiation between menopausal and perimenopausal status is important, as it determines whether LHRH agonist therapy is needed. Natural menopause is defined by the cessation of menstruation for at least 12 consecutive months without other pathological or physiological causes. E2 and FSH levels are also assessed to confirm postmenopausal status. Once confirmed, LHRH agonist therapy is not required for postmenopausal patients.

The manuscript describes that female patients aged 18-75 years, regardless of menopausal status, were eligible for inclusion, with premenopausal or perimenopausal patients receiving LHRH agonists as per the protocol. This provides a concise overview of the inclusion criteria, and more detailed information can be found in the study protocol.

**Reviewer #3, Comment 3:**

P3, L46. 'hypothesis generating trend' is a vague term, I think you could rephrase this to simply state these results instead.

**Response:**

Thank you for your valuable suggestion. In response to your comment, we have simplified the phrasing as recommended. Specifically, we removed the vague term “hypothesis-generating trend” and directly stated the observed findings. The corresponding changes have been made in the manuscript (P3, L48).

**Reviewer #3, Comment 4:**

P12, L227. I think it's ok to present the results by subgroup, but this should be carefully worded so that there is no direct comparison between them, as this study doesn't have the sample size to achieve this.

**Response:**

Thank you for your thoughtful comment. As the subgroups are based on post-hoc analysis and the study does not have the sample size to support direct comparisons, we agree with your suggestion to avoid such description. In response, we have made the necessary revisions to the manuscript. Specifically, we have removed the comparative language and now present the data for each subgroup separately (P13, L243-246, L248-251).

**Reviewer #4:** The manuscript presents a Phase II study of an antiestrogen, an investigational CDK 4/6inhibitor and an investigational HER2 targeting TKI used as first or second line therapy in metastatic breast cancer that is hormone receptor positive and HER2 positive.

The following comments are made in an attempt to improve the manuscript

**Reviewer #4, Comment 1:**

The introduction and the discussion should address that in early line therapy of metastatic breast cancer the use of an AI and CDK 4/6 inhibitor has shown an overall survival improvement and the regimen of docetaxel, trastuzumab, and pertuzumab has shown an overall survival improvement.

**Response:**

Thank you for your valuable comments. We have revised the Introduction and Discussion to address the overall survival improvement observed with AI plus CDK4/6 inhibitors in early-line therapy for HR+/HER2−ABC, as well as with the docetaxel, trastuzumab, and pertuzumab regimen in HER2+ metastatic breast cancer. The relevant updates have been incorporated into the manuscript (P6, L87-90, L100-103; P16, L314-316).

**Reviewer #4, Comment 2:**

It would be of interest to know what standard of care practice is for patients with hormone receptor positive, HER2 positive metastatic breast cancer at the Institutions where patients were enrolled.

**Response:**

Regarding the standard of care for ER+/HER2+ ABC at the institutions where patients were enrolled, this study was conducted across six tertiary hospitals in China, where treatment follows the CSCO guidelines. As a subset of HER2+ breast cancer, ER+/HER2+ ABC adheres to the treatment principles of HER2+ breast cancer. The first-line standard therapy primarily includes the THP regimen (docetaxel, trastuzumab, and pertuzumab (based on the CLEOPATRA and PUFFIN studies), with trastuzumab plus chemotherapy as an alternative. The second-line standard treatment includes pyrotinib plus capecitabine (based on the PHEOBE and PHENIX studies) as the preferred option, with T-DM1 as an alternative. Following the approval of the PHILA and DESTINY-Breast03 studies in China (which occurred after the study enrollment phase), the first-line standard of care now includes the TH + pyrotinib regimen (docetaxel, trastuzumab and pyrotinib), while T-DXd has become one of the preferred second-line treatments (P16-P17, L313-344).

**Reviewer #4, Comment 3:**

The manuscript is overly enthusiastic over this 48 patient study used in a population that is expected to have somewhat similar outcomes with standard of care. The tone can be revised.

**Response:**

Thank you for your comment. We agree with your observation regarding the tone of the manuscript. Given the small sample size, overly enthusiastic descriptions may not be appropriate. While the sample size is limited, the study does provide some promising indications. We haverevised the manuscript to adopt a more balanced tone, acknowledging the small sample size while emphasizing the positive insights the study offers (P16, L322; P17, L332).

**Reviewer #4, Comment 4:**

The manuscript is inconsistent with use of ER Positive and Hormone Receptor Positive. It is suggested that a definition is provided and only one term is used throughout.

**Response:**

Thank you for raising this critical point. We agree with your observation regarding the inconsistency in the use of “ER Positive” and “Hormone Receptor Positive”. To address this, we have revised the manuscript to consistently use “ER Positive” throughout, as this term more precisely reflects the inclusion criteria outlined in the study protocol. When citing literature that mentions Hormone Receptor (HR) in the article, its definition also be included (P6, L98). This revision aims to enhance clarity and ensure consistency across the manuscript.

**Reviewer #4, Comment 5:**

The manuscript uses the term "resistance" in manners that does not apply to this patient population (first or second line therapy). Please modify the text.

**Response:**

Thank you for your comment. This study includes first and second-line ER+/HER2+ advanced breast cancer patients, primarily focusing on evaluating the synergistic effects of the three-drug combination. In the subgroup analysis, for first-line HER2-targeted therapy, the original manuscript considered whether the combination could delay the onset of resistance by simultaneously targeting HER2, ER, and CDK4/6, thereby preventing the tumor from bypassing individual blockade mechanisms and improving treatment efficacy. For second-line therapy, the original manuscript considered whether the combination could reverse trastuzumab resistance (in patients with progression after trastuzumab treatment) and improve efficacy. The concept of "resistance" is more relevant in later-line therapy and requires a clear definition. Since this study predominantly enrolls early-line patients, the main objective is to explore whether the three-drug combination can provide synergistic effects and offer a chemotherapy-free alternative. To avoid any ambiguity, we have revised the manuscript to modify the description of “resistance” accordingly (P4, L60; P6, L87-97; P7, L121;P17, L346-349).

**Reviewer #4, Comment 6:**

It is suggested that the analysis of the data be revised to only use those patients who met enrollment criteria.

**Response:**

Thank you for your suggestion. Currently, only one patient who initially did not meet the enrollment criteria of hemoglobin has been included in the analysis. This patient received symptomatic treatment after enrollment, and their values recovered to meet the enrollment requirements specified in the protocol. Although this recovery occurred after the administration, the patient contributed valuable efficacy and safety data during the study, which justifies their inclusion in the analysis (P8, L144-148).

**Reviewer #4, Comment 7:**

The discussion is too long and discusses matters not directly related to the data presented in this manuscript. The discussion can be shortened and made more focused.

**Response:**

Thank you for your comment. We appreciate your feedback on the discussion section. We agree that it can be made more focused. We have revised the discussion to ensure it is more directly aligned with the data presented in the manuscript, removing any content that is not closely related to the findings. This will help improve clarity and ensure that the manuscript stays concise and focused on the key points (P16-P18, L313-368).

-----------------------------------------------------------

**General editorial requests:**

(Note: not all will apply to your paper, but please check each item carefully)

*	We ask every co-author listed on the manuscript to fill in a contributing author statement, making sure to declare all competing interests. If any of the co-authors have not filled in the statement, we will remind them to do so when the paper is revised. If all statements are not completed in a timely fashion this could hold up the re-review process. If new competing interests are declared later in the revision process, this may also hold up the submission. Should there be a problem getting one of your co-authors to fill in a statement we will be in contact. Please do not add or remove authors without first discussing this with the handling editor. You can see our competing interests policy here: http://journals.plos.org/plosmedicine/s/competing-interests.

**Response:**

Thank you for the reminder. I will ensure that all co-authors complete the contributing author statement and declare any competing interests. However, it seems that the link provided by the journal is no longer functional for some authors. Some of our co-authors have reported that they did not receive the emails regarding the confirmation of competing interests and author status. We kindly request that these emails be resent to the authors who have not yet confirmed, to ensure all authors can complete the required confirmations and declarations. Thank you again for your help. Additionally, if there are any changes to the author list, I will make sure to communicate with the editor in advance.

*	Please upload any figures associated with your paper as individual TIF or EPS files with 300dpi resolution at resubmission; please read our figure guidelines for more information on our requirements: http://journals.plos.org/plosmedicine/s/figures. While revising your submission, please upload your figure files to the PACE digital diagnostic tool, https://pacev2.apexcovantage.com/. PACE helps ensure that figures meet PLOS requirements. To use PACE, you must first register as a user. Then, login and navigate to the UPLOAD tab, where you will find detailed instructions on how to use the tool. If you encounter any issues or have any questions when using PACE, please email us at PLOSMedicine@plos.org.

**Response:**

Thank you for the reminder. I have already ensured that all figures are uploaded as individual TIF or EPS files with a resolution of 300dpi during resubmission.

*	Please ensure that the paper adheres to the PLOS Data Availability Policy (see http://journals.plos.org/plosmedicine/s/data-availability), which requires that all data underlying the study's findings be provided in a repository or as Supporting Information. For data residing with a third party, authors are required to provide instructions with contact information (web or email address) for obtaining the data. Please note that a study author cannot be the contact person for the data. PLOS journals do not allow statements supported by "data not shown" or "unpublished results." For such statements, authors must provide supporting data or cite public sources that include it.

**Response:**

Thank you for the reminder. I have ensured that the paper adheres to the PLOS Data Availability Policy.

*	We expect all researchers with submissions to PLOS in which author-generated code underpins the findings in the manuscript to make all author-generated code available without restrictions upon publication of the work. In cases where code is central to the manuscript, we may require the code to be made available as a condition of publication. Authors are responsible for ensuring that the code is reusable and well documented. Please make any custom code available, either as part of your data deposition or as a supplementary file. Please add a sentence to your data availability statement regarding any code used in the study, e.g. "The code used in the analysis is available from Github [URL] and archived in Zenodo [DOI link]" Please review our guidelines at https://journals.plos.org/plosmedicine/s/materials-software-and-code-sharing and ensure that your code is shared in a way that follows best practice and facilitates reproducibility and reuse. Because Github depositions can

be readily changed or deleted, we encourage you to make a permanent DOI'd copy (e.g. in Zenodo) and provide the URL.

**Response:**

Thank you for the reminder. This study did not involve any author-generated code.

*	FINANCIAL DISCLOSURES: The funding statement should include: specific grant numbers, initials of authors who received each award, URLs to sponsors’ websites. Also, please state whether any sponsors or funders (other than the named authors) played any role in study design, data collection and analysis, the decision to publish, or preparation of the manuscript. If they had no role in the research, include this sentence: “The funders had no role in study design, data collection and analysis, decision to publish, or preparation of the manuscript.”

**Response:**

Thank you for the reminder. I have updated the funding statement in the manuscript. Additionally, I have stated that the funders had no role in the study design, data collection and analysis, decision to publish, or preparation of the manuscript (P22, L443-445).

*	COMPETING INTERESTS: All authors must declare their relevant competing interests per the PLOS policy, which can be seen here: https://journals.plos.org/plosmedicine/s/competing-interests

For authors with ties to industry, please indicate whether any of the interests has a financial stake in the results of the current study.

**Response:**

Thank you for the reminder. I have ensured that all authors have declared their relevant competing interests as per the PLOS policy. For authors with ties to industry, I have stated that they have no financial stake in the results of the current study (P22, L455).

*	The Data Availability Statement (DAS) requires revision. For each data source used in your study:

a) If the data are freely or publicly available, note this and state the location of the data: within the paper, in Supporting Information files, or in a public repository (include the DOI or accession number).

b) If the data are owned by a third party but freely available upon request, please note this and state the owner of the data set and contact information for data requests (web or email address). Note that a study author cannot be the contact person for the data.

c) If the data are not freely available, please describe briefly the ethical, legal, or contractual restriction that prevents you from sharing it. Please also include an appropriate contact (web or email address) for inquiries (again, this cannot be a study author).

**Response:**

Thank you for your feedback. We have revised the Data Availability Statement to clarify that All data used in the manuscript can be found in the publicly available supporting files (P22, L458).

*	ETHICS STATEMENT: Please provide the approval number for each of the ethics committees.

**Response:**

Thank you for the reminder. I have provided the approval number for each of the ethics committees in the ethics statement, and the details are included in the supplementary materials in S1 Text.

FORMATTING - GENERAL

*	Abstract: Please structure your abstract using the PLOS Medicine headings (Background, Methods and Findings, Conclusions). Please combine the Methods and Findings sections into one section.

**Response:**

Thank you for the reminder. I have reviewed the abstract and confirmed that it follows the PLOS Medicine headings (Background, Methods and Findings, Conclusions). The Methods and Findings sections have been combined into one section as requested (P2-P3, L22-56).

*	At this stage, we ask that you include a short, non-technical Author Summary of your research to make findings accessible to a wide audience that includes both scientists and non-scientists. The Author Summary should immediately follow the Abstract in your revised manuscript. This text is subject to editorial change and should be distinct from the scientific abstract. Ideally each sub-heading should contain 2-3 single sentence, concise bullet points containing the most salient points from your study. In the final bullet point of 'What Do These Findings Mean?', please include the main limitations of the study in non-technical language. Please see our author guidelines for more information: https://journals.plos.org/plosmedicine/s/revising-your-manuscript#loc-author-summary.

**Response:**

Thank you for the reminder. I have added the short, non-technical Author Summary to the revised manuscript, immediately following the scientific abstract. The summary includes concise bullet points highlighting the most salient points from the study, along with the main limitations of the study in non-technical language as requested (P4-P5, L58-85).

*	Please express the main results with 95% CIs as well as p values. When reporting p values please report as p<0.001 and where higher as the exact p value p=0.002, for example. Throughout, suggest reporting statistical information as follows to improve clarity for the reader "22% (95% CI [13%,28%]; p</=)". Please be sure to define all numerical values at first use.

**Response:**

Thank you for the reminder. Since there were no data comparisons made for the main endpoint, p-values are not applicable. However, the main results are presented with 95% confidence intervals (CIs) as requested (P2-3, L42-44).

*	Please include page numbers and line numbers in the manuscript file. Use continuous line numbers (do not restart the numbering on each page).

**Response:**

Thank you for the reminder. I have added page numbers and continuous line numbers to the manuscript, as requested.

*	Please cite the reference numbers in square brackets. Citations should precede punctuation.

**Response:**

Thank you for the reminder. I have ensured that the reference numbers are cited in square brackets and placed before the punctuation, as requested.

FIGURES AND TABLES

*	Please provide titles and legends for all figures and tables (including those in Supporting Information files).

**Response:**

Thank you for the reminder. I have provided titles and legends for all figures and tables, including those in the Supporting Information files, as requested.

*	Please define all abbreviations used in each figure/table (including those in Supporting Information files).

**Response:**

Thank you for the reminder. I have defined all abbreviations used in each figure and table, including those in the Supporting Information files.

*	Please consider avoiding the use of red and green in order to make your figure more accessible to those with color blindness.

**Response:**

Thank you for the reminder. I have taken accessibility into account and have made sure to avoid using red and green in the figures to improve clarity and ensure the figures are accessible to a wider audience.

SUPPLEMENTARY MATERIAL

*	Please note that supplementary material will be posted as supplied by the authors. Therefore, please amend it according to the relevant comments outlined here.

**Response:**

Thank you for the reminder. I have noted that the supplementary material will be posted as supplied by the authors.

*	Please cite your Supporting Information as outlined here: https://journals.plos.org/plosmedicine/s/supporting-information

**Response:**

Thank you for the reminder. I have ensured that the Supporting Information has been properly cited as outlined in the provided link.

REFERENCES

*	PLOS uses the numbered citation (citation-sequence) method and first six authors, et al.

**Response:**

Thank you for the reminder. I have ensured that the manuscript follows the PLOS numbered citation method, including the first six authors followed by "et al."

*	Please ensure that journal name abbreviations match those found in the National Center for Biotechnology Information (NCBI) databases (http://www.ncbi.nlm.nih.gov/nlmcatalog/journals), and are appropriately formatted and capitalised.

**Response:**

Thank you for the reminder. I have verified that the journal name abbreviations match those found in the National Center for Biotechnology Information (NCBI) databases and have formatted and capitalized them appropriately.

*	Where website addresses are cited, please include the complete URL and specify the date of access (e.g. [accessed: 12/06/2023]).

**Response:**

Thank you for the reminder. I have ensured that all website addresses are cited with the complete URL and the date of access, as required.

*	Please also see https://journals.plos.org/plosmedicine/s/submission-guidelines#loc-references for further details on reference formatting.

**Response:**

Thank you for the reminder. I have reviewed the reference formatting guidelines provided in the link and have made the necessary adjustments.

STUDY TYPE-SPECIFIC REQUESTS

*	PLOS Medicine requires that all trials be prospectively registered in one of registries recognized by WHO. Please ensure that study registration details are included in the Methods section.

**Response:**

Thank you for the reminder. I have ensured that the study registration details are included in the Methods section as required (P8, L139).

*	Please structure the Methods section using the following sub-headings: Study design and participants, Randomization and masking, Procedures, Outcomes, Statistical analysis.

**Response:**

Thank you for the reminder. I have structured the Methods section using the following sub-headings: Study design, Participants, Interventions and procedures, Outcomes, and Statistical analysis.

*	Please ensure that all outcomes measures are reported according to the trial protocol [and/or trial registry]. Please clarify and explain all discrepancies between the paper and protocol. If the outcomes were not prespecified in the protocol, please define them in the Methods (Outcomes section) as post hoc and explain why they were added. Post-hoc comparisons should be presented as hypothesis generating rather than conclusive.

**Response:**

Thank you for the reminder. I have ensured that all outcomes measures are reported according to the trial protocol and registry. Any discrepancies between the paper and protocol have been clarified. For outcomes not prespecified in the protocol, I have defined them in the Methods section (Outcomes) as post hoc and explained why they were added. Post-hoc comparisons have been presented as hypothesis-generating rather than conclusive.

*	Please ensure that all prespecified outcomes (primary, secondary, and exploratory) are listed in the Methods/Outcomes section and indicate whether there are outcomes that are not presented in the current report.

**Response:**

Thank you for the reminder. I have ensured that all prespecified outcomes (primary, secondary, and exploratory) are listed in the Methods/Outcomes section, and I have confirmed that all outcomes are presented in the current report.

*	Please specify the dates (Month Day, Year) during which study enrollment and follow up occurred.

**Response:**

Thank you for the reminder. I have specified the dates (Month, Day, Year) during which study enrollment and follow-up occurred (P11, L217).

*	Please include absolute numbers wherever you report percentages; eg, n/N (%)

**Response:**

Thank you for the reminder. I have included absolute numbers wherever percentages are reported (e.g., n/N (%)).

*	Please present the safety data for the study including numbers of specific events and whether or not adverse events are thought to be related to treatment. AEs should be reported in the abstract, per CONSORT and CONSORT-Harms.

**Response:**

Thank you for the reminder. I have presented the safety data for the study, including the numbers of specific adverse events (AEs), and have indicated whether or not they are thought to be related to treatment. Adverse events (AEs) have been reported in the abstract, in accordance with CONSORT and CONSORT-Harms guidelines.

*	Please complete the CONSORT checklist (https://www.equator-network.org/reporting-guidelines/consort/) and ensure that all components of CONSORT are present in the manuscript, including how randomization was performed, allocation concealment, blinding of intervention, definition of lost to follow-up, power statement. When completing the checklist, please use section and paragraph numbers, rather than page numbers.

**Response:**

Thank you for the reminder. As this is a single-arm study, the full CONSORT checklist may not be fully applicable.

*	Please report your abstract according to CONSORT for abstracts, following the PLOS Medicine abstract structure (Background, Methods and Findings, Conclusions) <https://www.equator-network.org/reporting-guidelines/consort-abstracts/>

**Response:**

Thank you for the reminder. I have reported the abstract according to the CONSORT guidelines, following the structure of Background, Methods and Findings, and Conclusions as required.

*	If your trial had to undergo important modifications in response to extenuating circumstances, please complete the CONSERVE-CONSORT checklist and provide in your Supporting Information; (https://www.equator-network.org/reporting-guidelines/guidelines-for-reporting-trial-protocols-and-completed-trials-modified-due-to-the-covid-19-pandemic-and-other-extenuating-circumstances-the-conserve-2021-statement/). When completing the checklist, please use section and paragraph numbers, rather than page numbers.

**Response:**

Thank you for the reminder. As this study is not affected by extenuating circumstances, the CONSERVE-CONSORT checklist is not applicable.

*	In keeping with our commitment to Open Science, please include the study protocol document and analysis plan (including any amendments) as Supporting Information to be published with the manuscript if accepted.

**Response:**

Thank you for the reminder. In line with our commitment to Open Science, I will include the study protocol document (including any amendments) as Supporting Information to be published with the manuscript if accepted.

*	Please note that PLOS Medicine requires prospective, public registration of a data sharing plan (as part of mandatory clinical trials registration) for all clinical trials that began enrollment on or after January 1, 2019, in accordance with ICMJE requirements.

**Response:**

Thank you for the reminder. I have ensured that the study registration and data sharing plan, in accordance with ICMJE requirements, are included for this clinical trial.

*	In the main text, please clarify how the data was analysed (intention-to-treat, per protocol, etc.) and explain all discrepancies between the manuscript and protocol.

**Response:**

Thank you for the reminder. I have clarified how the data was analyzed in the main text (P11, L206-208), and I have explained any discrepancies between the manuscript and protocol(P12, L219-221).

**References**

1.	Li J, Hao C, Wang K, Zhang J, Chen J, Liu Y, et al. Chinese Society of Clinical Oncology (CSCO) Breast Cancer guidelines 2024. Translational breast cancer research : a journal focusing on translational research in breast cancer. 2024;5:18.

2.	Gradishar WJ, Anderson BO, Abraham J, Aft R, Agnese D, Allison KH, et al. Breast Cancer, Version 3.2020, NCCN Clinical Practice Guidelines in Oncology. Journal of the National Comprehensive Cancer Network : JNCCN. 2020;18(4):452-78.

3.	Gennari A, André F, Barrios CH, Cortés J, de Azambuja E, DeMichele A, et al. ESMO Clinical Practice Guideline for the diagnosis, staging and treatment of patients with metastatic breast cancer. Annals of oncology : official journal of the European Society for Medical Oncology. 2021;32(12):1475-95.

We have attached the revised manuscript and the detailed responses to each comment. We appreciate your consideration of our revisions and look forward to hearing your feedback.

Best regards,

Jian Zhang

Department of Medical Oncology, Fudan University Shanghai Cancer Center

syner2000@163.com
